# High expression of ABCF1 is an independent predictor of poor prognosis in bladder cancer

**DOI:** 10.1186/s12894-023-01211-y

**Published:** 2023-03-17

**Authors:** JiaWen Fan, Yi Ding, HaoXuan Huang, ShiDa Xiong, Liang He, Ju Guo

**Affiliations:** 1 Department of Urology, The First Affiliated Hospital of Nanchang University, Nanchang, Jiangxi China; 2 The Second Affiliated Hospital of Jiujiang University, Jiujiang, Jiangxi China

**Keywords:** Bladder cancer, ABCF1, GSEA, Prognosis

## Abstract

ABCF1, a member of the ATP-binding cassette (ABC) transporter family, is involved in the malignant progression of tumors. However, the role of ABCF1 in bladder cancer is poorly understood. In our study, we explored the differential expression of ABCF1 in bladder cancer and normal bladder tissues based on bioinformatic analysis and immunohistochemical results. GSEA was performed to ascertain the potential related signaling pathways of ABCF1. The relationship between ABCF1 expression and bladder cancer progression was analyzed using the GSE13507 dataset. In addition, the differential expression of ABCF1 in the cell lines was verified by quantitative real-time polymerase chain reaction (qRT‒PCR) and Western blotting. ABCF1 was upregulated in bladder cancer, and the high expression of ABCF1 was closely related to sex (*P* = 0.00056), grade (*P* = 0.00049), T stage (*P* = 0.00007), and N stage (*P* = 0.0076). High expression of ABCF1 was correlated with poor overall survival in bladder cancer patients (*P* < 0.001). In addition, univariate and multivariate Cox regression analyses showed that high ABCF1 expression was an independent factor for poor prognosis in bladder cancer patients. Therefore, ABCF1 expression is closely related to the progression of bladder cancer and can be used as a potential indicator of poor prognosis and a therapeutic target for bladder cancer.

**Supplementary Information:**

The online version contains supplementary material available at 10.1186/s12894-023-01211-y.

## Introduction

According to 2020 global cancer statistics, bladder cancer is a common malignant tumor of the genitourinary system and ranks 10th in global malignant tumor diagnosis. Every year, more than 570,000 patients are diagnosed with bladder cancer, and over 210,000 people die of bladder cancer globally [1]. Relevant epidemiological studies have confirmed that smoking is the main risk factor for inducing carcinogenic mutations in the bladder. However, smoking cessation strategies do not appear to have an effect on mortality in bladder cancer patients [2, 3]. Bladder cancer is reportedly one of the most frequently mutated cancers after lung and skin cancers [4]. After multiple divisions and proliferations as bladder cancer grows, bladder tumor cells often exhibit changes in molecular biology or genes, which result in differences in the growth rate, invasion ability, drug sensitivity, prognosis and other aspects of the tumor. These observations suggest that bladder tumors are characterized by high mutational heterogeneity, which is also a feature that distinguishes bladder cancer from other cancers [5, 6]. Bladder cancer is divided into nonmuscle invasive bladder cancer (NMIBC) and muscle invasive bladder cancer (MIBC). Clinical treatment is mainly endoluminal resection and radical resection of bladder cancer, and adjuvant chemotherapy is given after surgery. However, half of NMIBC patients still experience recurrence and metastasis after local infusion chemotherapy or BCG and develop resistance to a new round of chemotherapy; MIBC patients can also develop resistance to adjuvant chemotherapy after surgery, and the prognosis will be worse [7]. Therefore, to better elucidate the relevant molecular mechanisms of the occurrence and development of bladder cancer, it is crucial for us to explore novel biomarkers as potential therapeutic targets for bladder cancer.

The ATP-binding cassette (ABC) transporter is a ubiquitous membrane-bound protein present in both prokaryotes and eukaryotes [8]. ABC transporters transport various molecules through the outer and inner membranes of cells, including those involved in the expulsion of toxic endogenous molecules and exogenous substances from cells. Thus, multiple overexpressed ABC transporters can facilitate drug efflux and promote multidrug resistance in cancer cells [9]. ABC transporters, including ABCB1, ABCB5, and ABCG2, have been reported to have stem cell-related roles in multiple cancers, such as differentiation, self-renewal, and stem cell markers [10–12]. ABCF1 (also known as ABC50) is an ABC transporter family protein that was originally identified as a protein that was upregulated in synovial cells in the presence of TNF-α [13]. Research on ABCF1 is relatively rare, and only some of its functions have been discovered in cell or mouse experiments. ABCF1 is involved in the initiation of ribosome biogenesis, regulates protein synthesis [14–16] and is essential for innate immune responses that regulate cellular cytoplasmic DNA and retroviral infection [17]. In addition, ABCF1 is very important to mouse embryonic development, and knocking out the *ABCF1* gene can lead to embryonic lethality [18]. Lindqvist et al. [19] demonstrated that protein synthesis inhibitors trigger cell death. Paytubi et al. demonstrated that knockdown of ABCF1 resulted in the suppression of total protein synthesis [16]. These results suggest that the functions exerted by ABCF1 are important in the control of eukaryotic biogenesis and function or translation machinery. In cancer, related reports show that there may be some correlation between high expression of ABCF1 and the metastatic potential of soft tissue tumors [20, 21]. However, the role of ABCF1 in bladder cancer is poorly understood.

In this study, we explored the expression of ABCF1 in bladder cancer and studied the prognosis and molecular function of ABCF1. Our findings suggest that high expression of ABCF1 is a factor in poor prognosis of bladder cancer patients and may serve as a potential target for future therapeutic strategies.

## Materials and methods

### Data collection and processing

We downloaded the gene expression data of 19 normal bladder tissues and 414 bladder cancer tissues from the TCGA database (TCGA, https://portal.gdc.cancer.gov/). To investigate the role of ABCF1 in bladder cancer development, we downloaded the GSE13507 dataset from the Gene Expression Omnibus (GEO) database. Ten normal bladder mucosa samples and 165 primary bladder cancer samples were downloaded, and the 165 bladder cancer patients had detailed clinical information. We also collected immunohistochemical (IHC) data for the *ABCF1* gene in bladder cancer and normal tissues from the Human Protein Atlas (HPA, http://www.proteinatlas.org/) database.

### Patients and follow‐up

According to the established scheme authorized by the Medical Science Research Ethics Committee of the First Affiliated Hospital of Nanchang University, informed consent was obtained from all patients in the study. In this study, a total of 120 paraffinized primary specimens were collected, all of which were matching specimens of cancer tissues and adjacent tissues. The inclusion criteria were as follows: (1) all operations were performed by the same group of urologists; (2) postoperative pathology confirmed bladder cancer; and (3) patients had complete clinical data. Prior to registration, each participant provided written informed consent. The median follow-up period was 40 months (range 1–60 months). The overall survival rate (OS) of patients refers to the time between surgery and death or between surgery and the last observation for survivors.

### Gene set enrichment analysis

In the present study, to further understand the biological function of ABCF1 in bladder cancer, we performed gene set enrichment analysis (GSEA) with the phenotypic signatures of the high-ABCF1 group and low-ABCF1 group. KEGG pathway enrichment analysis was performed using GSEA 4.1.0 software, and the number of random combinations was set to 1000. An absolute value normalized enrichment score (NES) greater than 1, normal *P* value < 0.05 and false discovery rate (FDR) < 0.25 were used as enrichment criteria.

### Gene ontology (GO) enrichment and kyoto encyclopedia of genes and genomes (KEGG) pathway analysis

We screened for differentially expressed genes (DEGs) associated with ABCF1 using the criteria of log2-fold change (FC) > 0.5 or ≤ 0.5 and *P* < 0.05. In addition, the biological functions of DEGs were systematically examined by GO enrichment and KEGG pathway analysis [22–24] using R packages *DOSE*, *clusterProfiler*, *enrichplot,* and *ggplot2*. Both P and FDR values < 0.05 were considered statistically significant.

### Protein‒protein interaction (PPI) network construction and module screening

The DEGs were submitted to the STRING database (http://www.string-db.org/) for detection of protein‒protein interactions (PPIs). The PPI network was then constructed and visualized using Cytoscape 3.8.2. The Molecular Complex Detection (MCODE) plugin was used to screen out key modules with MCODE scores and node counts > 5 from the PPI network. *P* < 0.05 was considered statistically significant.

### Quantitative real-time polymerase chain reaction (qRT‒PCR)

To further confirm the high expression of ABCF1 in bladder cancer and its reliability in predicting poor prognosis, we performed qRT‒PCR. Seven different cell lines were used: T24, 5637, EJ, J82, UMUC3 and BIU-87 are bladder cancer cell lines, and SV-HUC-1 is a normal bladder epithelial cell line. The primer sequences for ABCF1 are as follows: forward primer, 5′-CCACACTCCTCAAGCACA-3′; reverse primer, 3′-GCTCACACAGCAACACATC-5′. The expression data were calculated by the 2^−△△CT^ method, and the relative expression level of the results was obtained with ACTIN as the internal reference.

### Western blotting

Cells and tissues were lysed using RIPA buffer (R0020; Solarbio, Beijing, China), to which a mixture of protease phosphatase inhibitors (P1261; Solarbio, Beijing, China) was added. We used β-actin (1:1000, GB12001; Servicebio, Wuhan, China) primary antibodies as an internal reference. All antibody information used in this study is listed in Additional file 1: Table B1. The electrophoretic protein was transferred to a PVDF membrane. We then blocked the membranes in 5% skim milk for 1 h and incubated them overnight at 4 °C with primary antibodies. Next, the membranes were washed three times and incubated with goat anti-rabbit secondary antibodies (BA1054; BOSTER, Wuhan, China) conjugated with horseradish peroxidase (HRP) for 2 h. Finally, the protein bands were detected by P2300 (NCM Biotech, Suzhou, China). Images were captured using ImageQuant LAS 500 (GE, Boston, America).

### Immunohistochemistry (IHC)

Paraffin-embedded normal bladder and bladder cancer tissue sections were deparaffinized and rehydrated, and 3% hydrogen peroxide was utilized to quench endogenous peroxidase activity. Next, sections were cultured with bovine serum albumin (G5001; Servicebio, Wuhan, China) for 30 min. Tissue sections were incubated with rabbit anti-human ABCF1 monoclonal antibody (1:100 dilution; EPR16068; ABCAM, UK) in a wet box at 4 °C overnight. The samples were then incubated at 37 °C for 50 min with a goat anti-rabbit secondary antibody (G1213; Servicebio, Wuhan, China) labeled with biotin and streptomycin. Next, the slide was stained with diaminobenzidine and counterstained with hematoxylin. We acquired images of each section using an optical microscope (Eclipse E100; Nikon, Tokyo, Japan) and imaging system (DS-U3; Nikon). IHC evaluation was carried out by two independent pathologists. To reveal positively stained areas of tissue, a brownish yellow stain was used to contrast the blue background color (hematoxylin). Signal intensity (SI) was divided into 3 grades: grade 0 indicated no positive staining, grade 1 indicated light yellow weakly positive staining, grade 2 indicated brownish yellow medium positivity, and grade 3 indicated brown strong positivity. There were four grades of PP (positive cell ratio): grade 0 indicates 0 ~ 5%, grade 1 indicates 6 ~ 25%, grade 2 indicates 26 ~ 50%, grade 3 indicates 51 ~ 75%, and grade 4 indicates > 75%.

### Statistical analysis

All statistical analyses were performed by R (4.0.4) and GraphPad Prism (version 7.0). We analyzed the expression levels of ABCF1 by the Wilcoxon signed-rank test. Survival analysis was assessed using Cox regression multivariate analysis and the Kaplan‒Meier method. The expression level of ABCF1 between bladder cancer cell lines and normal epithelial cells was verified using Student's t test. *P* < 0.05 was considered statistically significant. *P* value is denoted by *(*P < 0.05, **P < 0.01, 254 ***P < 0.001, ****P < 0.0001).

## Results

### Upregulation of ABCF1 in bladder cancer tissues

**Fig. 1:**
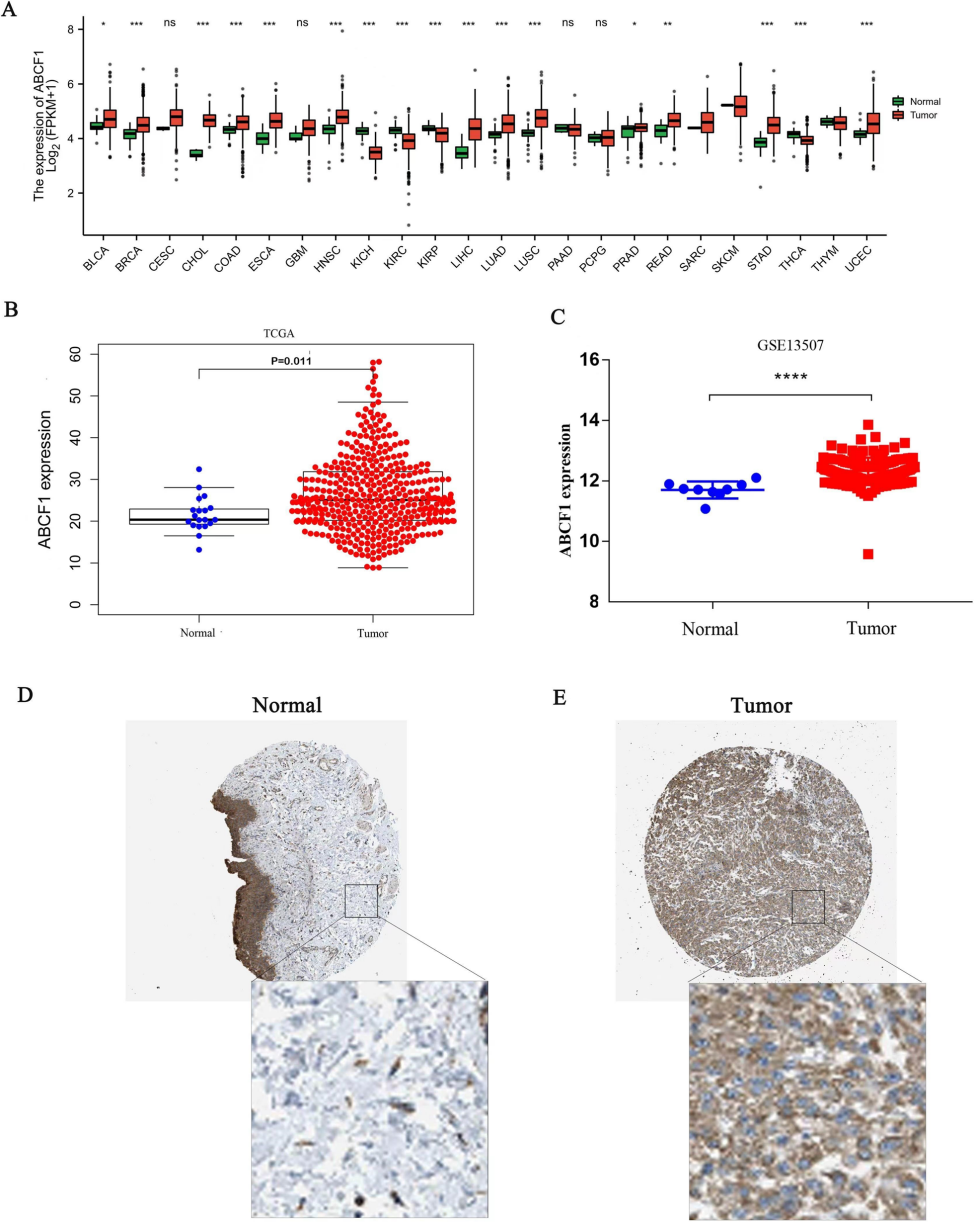
ABCF1 expression levels in bladder cancer. **A** Transcript levels of ABCF1 in different types of cancers from the TCGA database. **B**, **C** Transcript levels of ABCF1 from the TCGA and GSE13507 datasets. **D**, **E** Protein expression of ABCF1 in normal tissue and bladder cancer from HPA data. *P < 0.05; **P < 0.01; ***P < 0.001; ****P < 0.0001

We initially assessed ABCF1 transcript levels in different human tumors by analyzing RNA-seq data using the TCGA database. As shown in Fig. 1A, ABCF1 was differentially expressed in 17 cancer types, including bladder cancer. This suggests that ABCF1 is aberrantly expressed in a variety of tumors. Then, we further evaluated the expression level of ABCF1 in bladder cancer using TCGA and GSE13507 data and found that the ABCF1 mRNA expression level was significantly increased in bladder cancer tissue compared with normal bladder tissue (Fig. 1B, C). Furthermore, as shown in Fig. 1D, E, immunohistochemical staining data from the HPA showed that ABCF1 protein expression was significantly elevated in bladder cancer tissues compared to normal tissues. Overall, these results suggest that ABCF1 is elevated in bladder cancer.

### GSEA identifies ABCF1-related signaling pathways

**Fig. 2:**
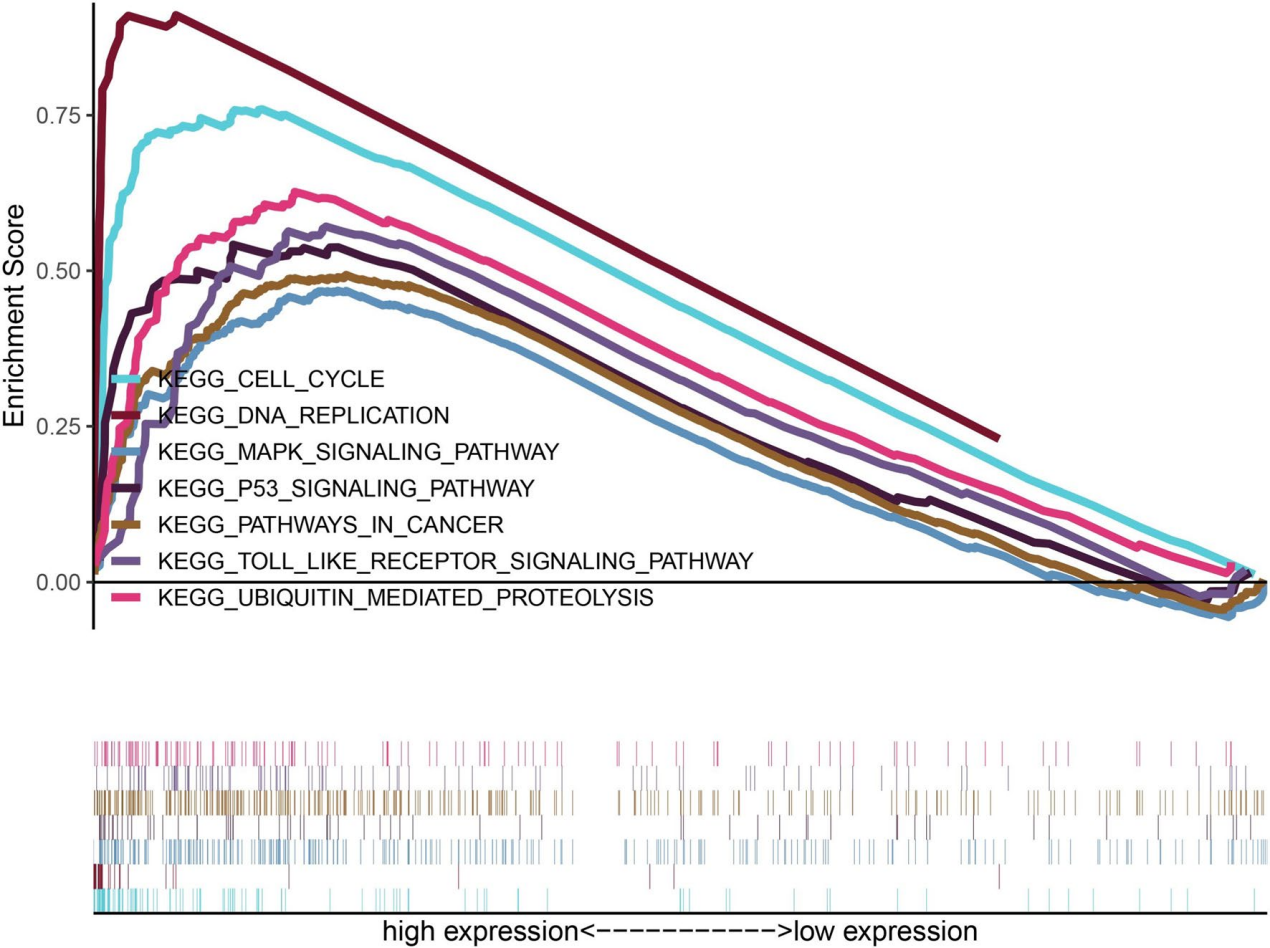
Gene set enrichment analysis (GSEA) results based on ABCF1 mRNA expression in bladder cancer from the TCGA dataset

On the basis of TCGA data, we explored ABCF1-related signal transduction pathways by GSEA [22–24]. Significantly enriched signaling pathways were selected using the criteria of NES, NOM p values and FDR q-values. In this study, the cell cycle, ubiquitin-mediated proteolysis, DNA replication, cancer pathways, Toll-like receptor pathways, p53 signaling, and MAPK signaling were differentially enriched in the high-ABCF1 phenotype (Fig. 2 and Table 1).

**Table 1:** Gene sets enriched in the high expression phenotype

MSigDB collection	Gene set name	NES	NOM *P*-value	FDR *q*-value
c2.cp.ke gg.v7.1 symbols .gmt	KEGG_CELL_CYCLE	2.43	0.000	0.000
	KEGG_UBIQUITIN_MEDIATED_PROTEOLYSIS	2.38	0.000	0.000
	KEGG_DNA_REPLICATION	2.33	0.000	0.001
	KEGG_PATHWAYS_IN_CANCER	2.09	0.000	0.001
	KEGG_TOLL_LIKE_RECEPTOR_SIGNALING_PATHWAY	2.04	0.002	0.003
	KEGG_P53_ SIGNALING_PATHWAY	2.00	0.000	0.004
	KEGG_MAPK_SIGNALING_PATHWAY	1.98	0.000	0.004

### The relationship between ABCF1 expression and the clinicopathological characteristics of bladder cancer patients

**Fig. 3:**
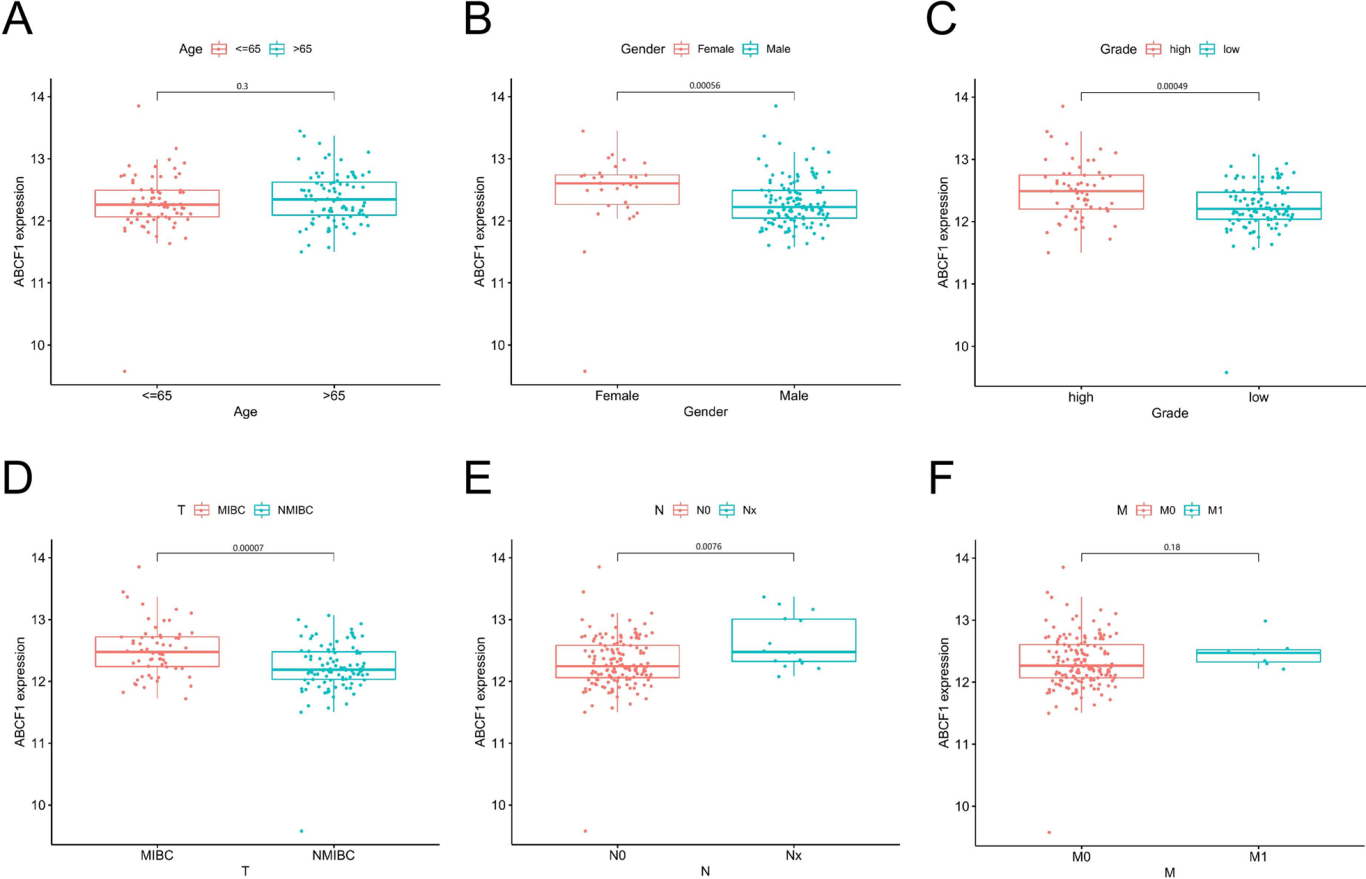
The relationships between ABCF1 expression and clinicopathological features in bladder cancer. The results show correlation between age (**A**), gender (**B**), Fuhrman grade (**C**), T classification (**D**), N classification (**E**), M classification (**F**) and ABCF1 expression

Because of the rich clinical data of 165 bladder cancer patients in the GSE13507 dataset, we used this dataset to investigate the association between ABCF1 expression and clinicopathological features, including age, sex, Fuhrman grade, depth of invasion (T classification), lymph node metastasis (N classification), and distant metastasis (M classification). As shown in Fig. 3, high expression of ABCF1 was closely related to sex (*P* = 0.00056), Fuhrman grade (*P* = 0.00049), T classification (*P* = 0.00007), and N classification (*P* = 0.0076). However, ABCF1 expression was not significantly associated with age (*P* = 0.3) or M classification (*P* = 0.18). In conclusion, the above results suggest that high expression of ABCF1 is associated with the prognosis of bladder cancer.

### ABCF1 is an independent factor of poor prognosis for bladder cancer

**Fig. 4:**
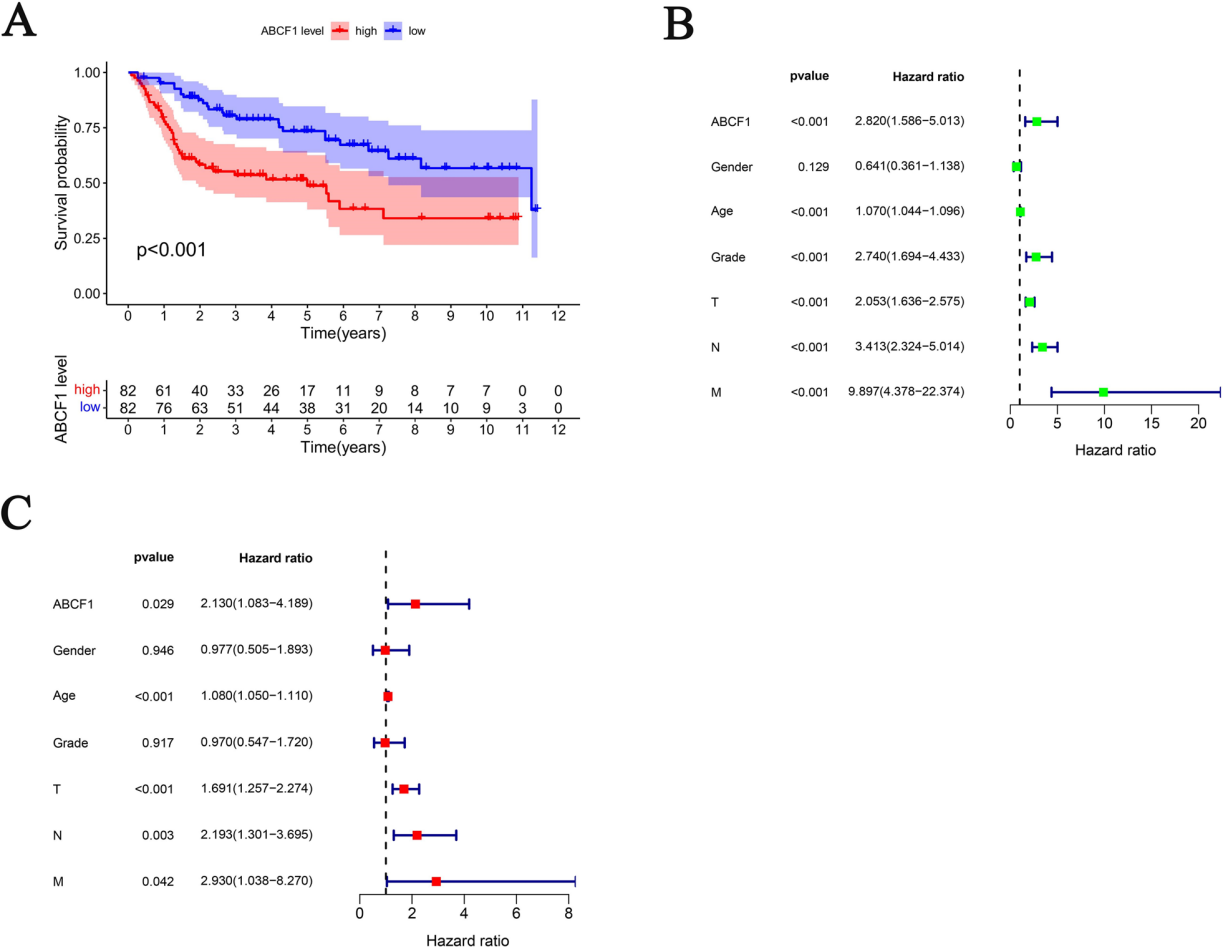
**A** Overall survival curves of bladder cancer patients with high expression versus low expression of ABCF1. **B**, **C** Univariate and multivariate Cox regression analyses of the association between clinicopathological features and the overall survival rate of patients

We analyzed the prognostic value of ABCF1 expression in bladder cancer using the GSE13507 dataset. As shown in Fig. 4A, overall survival (OS) was significantly shorter in patients with higher ABCF1 expression than in those with lower ABCF1 expression (*P* < 0.001). In addition, we also evaluated the effect of ABCF1 expression on the survival of bladder cancer patients by univariate and multivariate Cox regression analyses. In univariate analysis, high ABCF1 expression was significantly associated with poorer survival probability [HR: 2.820, 95% CI 1.586–5.013; *P* < 0.001]. In addition, age, grade, T classification, N classification, and M classification were also included (Fig. 4B). As shown in Fig. 4C, multivariate analysis had the same result [high expression of ABCF1, HR: 2.130, 95% CI 1.083–4.189, *P* = 0.029]. The above results indicate that ABCF1 is a factor of poor prognosis and an independent prognostic marker in bladder cancer.

### Differential expression and pathway analysis for DEGs of ABCF1^high^ versus ABCF1^low^

**Fig. 5:**
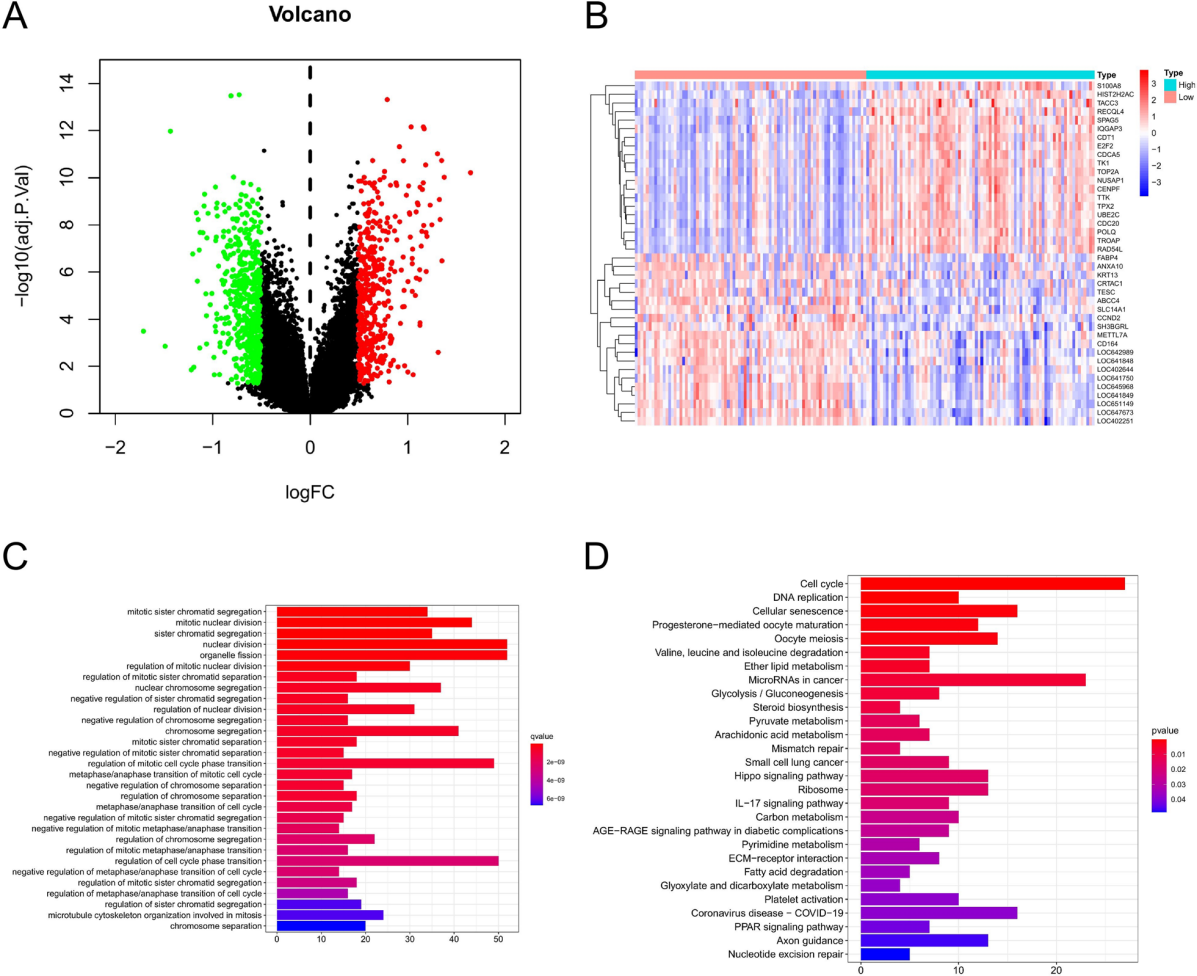
**A** Volcano plot of the DEG expression between ABCF1^high^ and ABCF1^low^ patients The red dots represent 437 upregulated genes, the green dots represent 535 downregulated genes, and the black dots denote nonsignificant genes. **B** Heatmap demonstrating the top 20 upregulated genes and the top 20 downregulated genes. Red indicates high expression, white indicates intermediate expression, and blue represents low expression. **C**, **D** GO and KEGG analyses show different enriched pathways for differentially expressed genes

To identify the genes associated with ABCF1, we compared the differential expression values of ABCF1^high^ and ABCF1^low^ patients. Among them, 437 genes were upregulated and 535 were downregulated [(FC, log2) > 0.5 or ≤ 0.5, *P* < 0.05, Fig. 5A]. The top 20 upregulated and top 20 downregulated genes are shown in the heatmap (Fig. 5B). To investigate the potential functions and mechanisms of ABCF1, we performed GO and KEGG pathway enrichment analyses on the differentially expressed genes (DEGs) associated with ABCF1. GO enrichment analysis showed that most of the DEGs were enriched in mitotic sister chromatid segregation, mitotic nuclear division, nuclear division, organelle fission, regulation of mitotic cell cycle phase transition, and regulation of cell cycle phase transition (Fig. 5C). In the KEGG analysis results, most of the DEGs were enriched in the cell cycle, DNA replication, cellular senescence, and microRNAs in cancer pathways (Fig. 5D).

### PPI network construction and key module screening

**Fig. 6:**
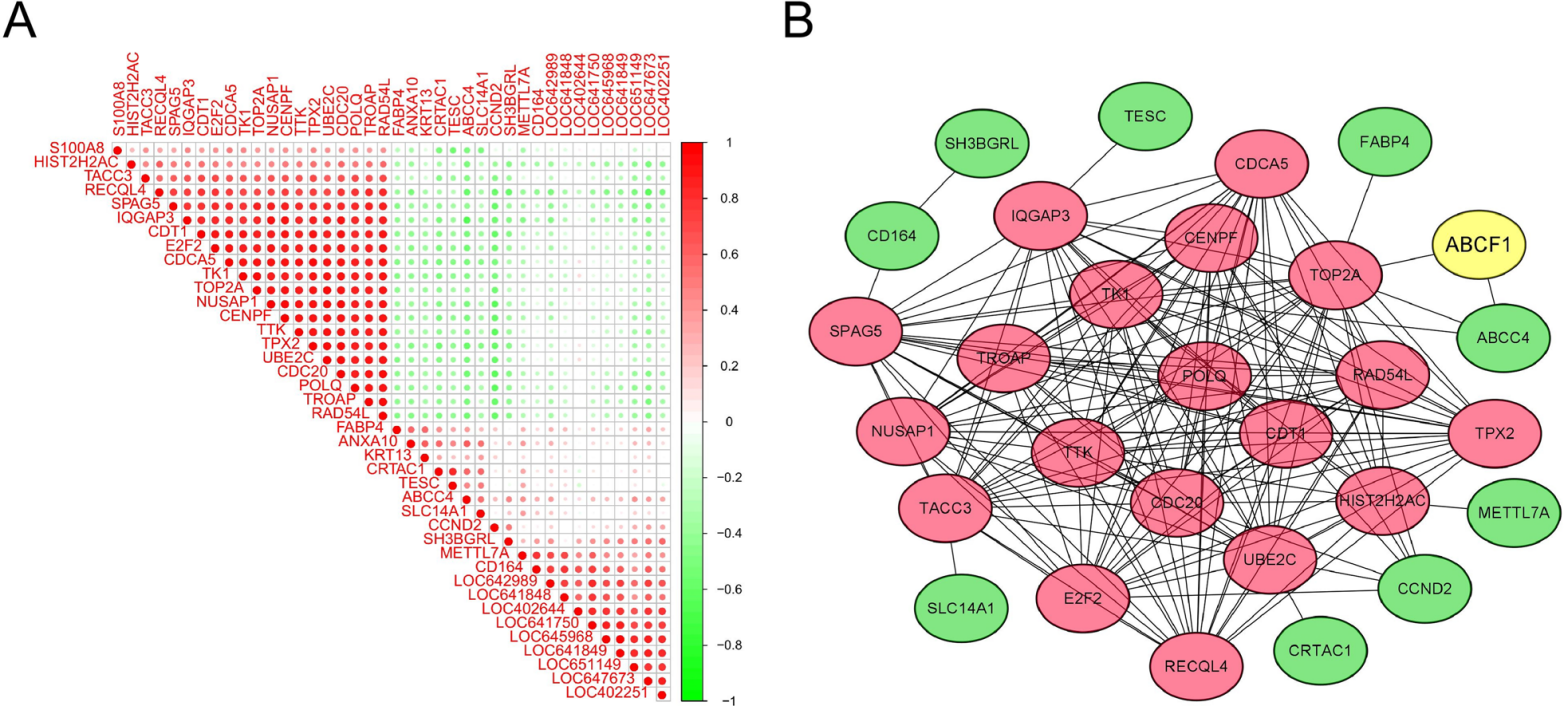
Correlation analysis and PPI results of DEGs. **A** Correlation analysis of DEGs through the Pearson correlation coefficient; the green dots indicate a negative correlation, while the red dots indicate a positive correlation. **B** PPI network of the most common DEGs; green indicates downregulated genes, while red represents upregulated genes

We calculated the correlation between the top 40 DEGs of ABCF1^high^ and ABCF1^low^ patients (Fig. 6A). In addition, the top 40 DEGs and ABCF1 were screened through the STRING database to identify the relevant PPI network. Most upregulated and downregulated genes interacted in the PPI network (Fig. 6B). *TOP2A*, *CENPF*, *TROAP*, *CDC20*, *CDCA5*, and *UBE2C* are all reported to be associated with cancer [25–30].

### Validation of ABCF1 upregulation in bladder cancer by qRT‒PCR and Western blotting

**Fig. 7:**
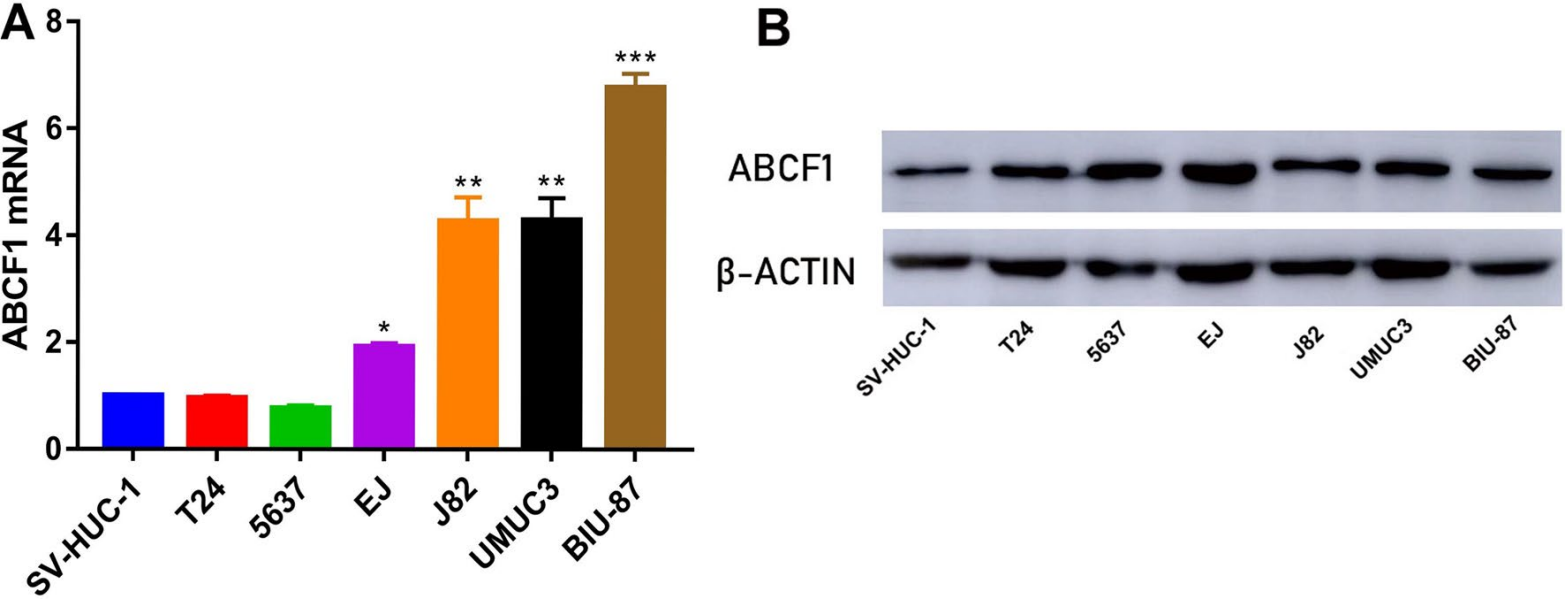
**A** The relative mRNA expression of *ABCF1* in the SV-HUC-1, T24, 5637, EJ, J82, UMUC3, and BIU-87 cell lines. *P < 0.05; **P < 0.01; ***P < 0.001. **B** WB analysis of ABCF1 expression in bladder cancer cell lines (WB bands were cropped from the same gels/blots)

To verify the differential expression of ABCF1 in the above databases, we used qRT‒PCR to detect the relative expression of *ABCF1* mRNA in bladder cancer cell lines (Fig. 7A). Western blotting was used to detect the expression of ABCF1 in bladder cancer cell lines and normal bladder epithelial cells (Fig. 7B). The results showed that the expression level of *ABCF1* mRNA was significantly higher in most bladder cancer cell lines than in the normal bladder epithelial cell line SV-HUC-1 (Additional file 3).

### IHC assay shows that ABCF1 expression was significantly higher and associated with a poor prognosis

**Fig. 8:**
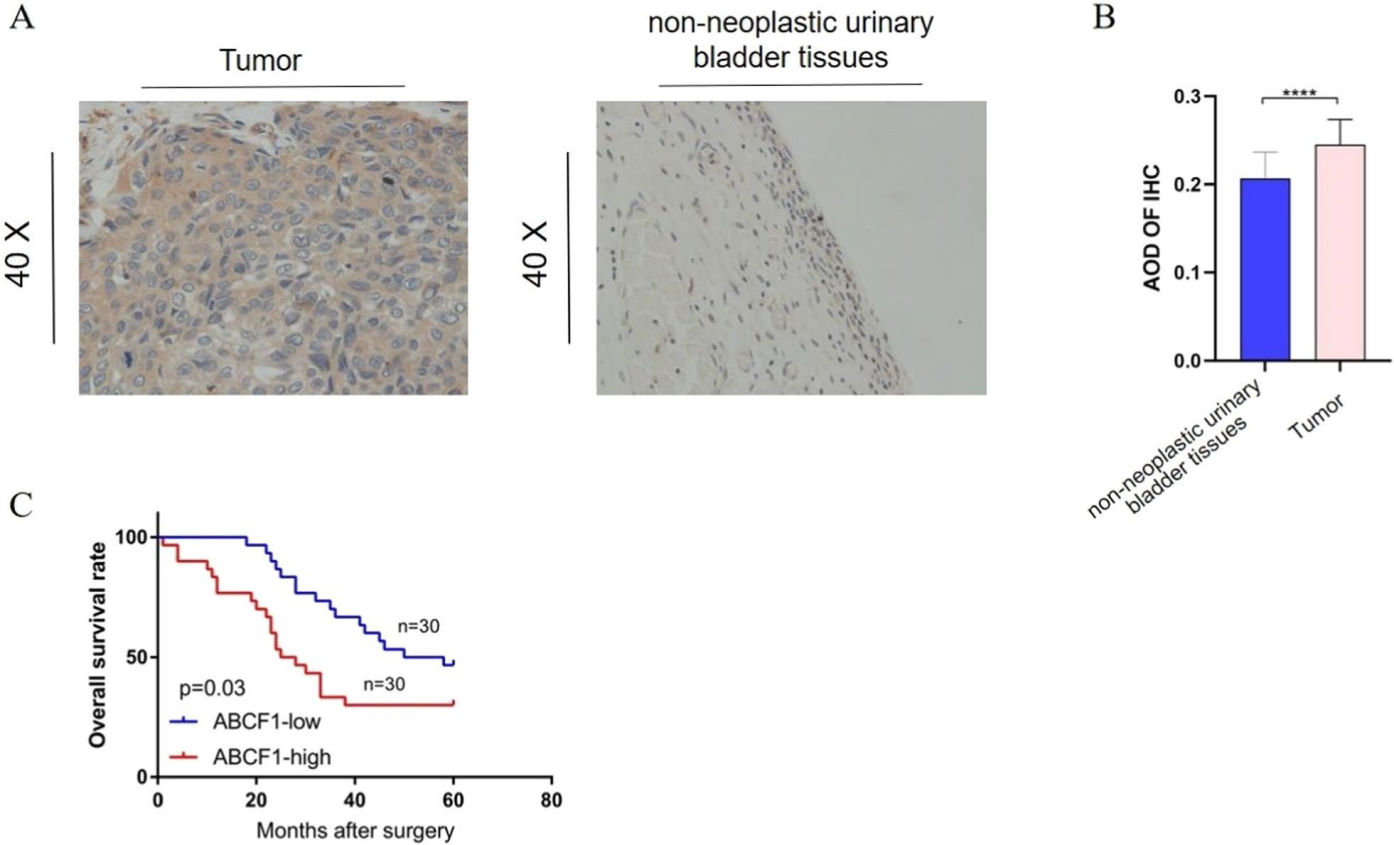
Differential expression of ABCF1 and its effect on prognosis. **A** Representative expression of ABCF1 (40×) in bladder cancer tissues and paired adjacent tissues (scale 289 bar, 100 μm) (Additional files 5, 6). **B** The AOD of tumors was significantly higher than that of nonneoplastic urinary bladder samples (*P* < 0.0001). **C** Survival analysis of 60 bladder cancer patients with respect to ABCF1 expression (ABCF1^low^, n = 30; ABCF1^high^, n = 30; *P* = 0.03; median AOD = 0.22) (Additional file 4)

To prove the differences at the organizational level, we compared the expression of ABCF1 in cancer and adjacent tissues of 60 bladder cancer patients in our hospital and found that the expression of ABCF1 was also increased in cancer tissues (Fig. 8A), and the AOD was higher in tumors than in nonneoplastic urinary bladder samples (*P* < 0.0001) (Fig. 8B). Moreover, the protein expression of ABCF1 in bladder cancer tissues was significantly higher than that in paired adjacent noncancer tissues. A survival analysis of the prognostic information of 60 bladder cancer patients using immunohistochemical results showed that the higher the expression of ABCF1, the worse the prognosis of the patient was (*P* = 0.03; Fig. 8C) (Additional file 2).

## Discussion

ABCF1, unlike other ABC transporter family members, does not have a transmembrane domain [16]. Therefore, ABCF1 may be functionally different from most ABC transporters [9]. It has been reported that ABCF1 is overexpressed in liver cancer; is a liver tumor fetal protein that promotes chemotherapy resistance, epithelial-mesenchymal transition and tumor stem cell differentiation in liver cancer cells; and has potential as a therapeutic target. However, the prognostic role of ABCF1 in bladder cancer remains unclear. Therefore, we aimed to explore whether ABCF1 could serve as a prognostic target [31] in bladder cancer patients through bioinformatics analysis.

In our study, bioinformatics analysis of ABCF1 datasets from public databases revealed that the expression of ABCF1 was significantly increased in bladder cancer compared with normal bladder tissue. This is consistent with our results using qRT‒PCR and Western blotting to validate ABCF1 mRNA expression levels in bladder cancer cell lines. Next, we showed that ABCF1 is highly expressed in bladder cancer tissue through an IHC assay, and we performed a survival analysis of the prognostic information of bladder cancer patients, which showed that the higher the expression of ABCF1, the worse the prognosis of the patients was. In addition, GSEA results showed that the ABCF1^high^ expression phenotype was associated with the cell cycle, ubiquitin-mediated proteolysis, DNA repair, cancer pathway, Toll-like receptor pathway, p53 signaling pathway and MAPK signaling pathway. Several studies have shown that the cell cycle [32, 33], ubiquitin-mediated proteolysis [34], DNA replication [35, 36], Toll-like receptor pathway [37, 38], p53 signaling pathway [39, 40] and MAPK signaling pathway [41] are closely related to cancer. Importantly, Arora et al. recently reported that ABCF1 is an E2 ubiquitination-conjugating enzyme [42]. The E2 ubiquitination-conjugating enzyme plays a central role in the ubiquitination system and is involved in various cancer-promoting processes [43]. These results suggest that ABCF1 may be an oncogene that plays an important role in bladder cancer progression.

Through analysis of the GSE13507 dataset, we also found that ABCF1 expression was closely associated with multiple clinicopathological features, including Fuhrman grade, T classification, N classification, age and sex. This suggests that bladder cancers with high ABCF1 expression may be more likely to progress to an advanced stage. Furthermore, Kaplan‒Meier survival analysis showed that high expression of ABCF1 was associated with poor prognosis in patients with bladder cancer. Univariate and multivariate Cox regression analyses indicated that ABCF1 expression was an independent prognostic factor in bladder cancer. This study suggests that ABCF1 may serve as a novel biomarker for predicting poor prognosis in bladder cancer.

GO analysis showed that most DEGs were enriched in mitotic sister chromatid segregation, mitotic nuclear division, nuclear division, organelle fission, regulation of mitotic cell cycle phase transition, and regulation of cell cycle phase transition. The KEGG pathways were mainly enriched in the cell cycle, DNA replication, cellular senescence, and microRNAs in cancer. In addition, we also found that many cancer-related genes interact with ABCF1 through the PPI network. Among them, *TOP2A*, *CDC20*, *CDCA5* and *UBE2C* all act as oncogenes in bladder cancer and promote tumor progression [44–47]. Therefore, these findings once again suggest that ABCF1 may be an oncogene in bladder cancer (Additional file 4).

## Conclusions

In conclusion, our study found that ABCF1 could serve as a prognostic marker and potential therapeutic target for bladder cancer. However, this study is mainly based on the GSE13507 dataset and immunohistochemistry (IHC), which still has certain limitations. Thus, we are planning to perform a series of experiments to reveal the prognostic value of ABCF1 in bladder cancer (Additional file 5 and Additional file 6).

## Supplementary Information

**Additional file 1**. **Table B1**:The primary antibodies for western blot and IHC

**Additional file 2**. The AOD of immunohistochemistry in 60 patients.

**Additional file 3**. **WB**：the result of Western blotting

**Additional file 4**. **PI**: prognostic information of patients

**Additional file 5**. **IHC 1**: cancer tissues Immunohistochemical result of patients

**Additional file 6**. **IHC 2**: normal tissues Immunohistochemical result of patients

## Data Availability

The RNA-seq data and clinical follow-up data associated with BC patient samples were downloaded from the TCGA database (https://cancergenome.nih.gov/). The GSE13507 dataset was downloaded from the Gene Expression Omnibus (GEO) database (https://www.ncbi.nlm.nih.gov/geo/). The immunohistochemical (IHC) data of the ABCF1 gene in bladder cancer and normal tissues were downloaded from the Human Protein Atlas (HPA, http://www.proteinatlas.org/) database. The immunohistochemical results and survival time of patients used to support the findings of this study are included within the supplementary information files. The datasets used during the current study are available from the corresponding author on request.
