# Supplementary material for: High expression of ABCF1 is an independent predictor of poor prognosis in bladder cancer
Source: BMC Urol. 2023 Mar 17;23:37. doi: 10.1186/s12894-023-01211-y (PMC10022215; doi:10.1186/s12894-023-01211-y)
Supplement: Supplementary file 1 — Additional file 1. Table B1:The primary antibodies for western blot and IHC [file 12894_2023_1211_MOESM1_ESM.docx]

| **Antibody Concentration Concentration Concentration Specificity Company**  **for WB for IHC for IF** | | | | | |
| --- | --- | --- | --- | --- | --- |
| ABCF1 | 1:1000 | 1:100 | / | Rabbit monoclonal | Abcam |
| β-Actin | 1:1000 | / | / | Monoclonal mouse | Servicebio |

**Table B1. The primary antibodies for western blot and IHC**

Abbreviations: WB, western blot; IHC, immunohistochemistry;
