# Supplementary material for: High expression of ABCF1 is an independent predictor of poor prognosis in bladder cancer
Source: BMC Urol. 2023 Mar 17;23:37. doi: 10.1186/s12894-023-01211-y (PMC10022215; doi:10.1186/s12894-023-01211-y)
Supplement: Supplementary file 2 — Additional file 2. The AOD of immunohistochemistry in 60 patients. [file 12894_2023_1211_MOESM2_ESM.docx]

**The AOD of immunohistochemistry in 60 patients**


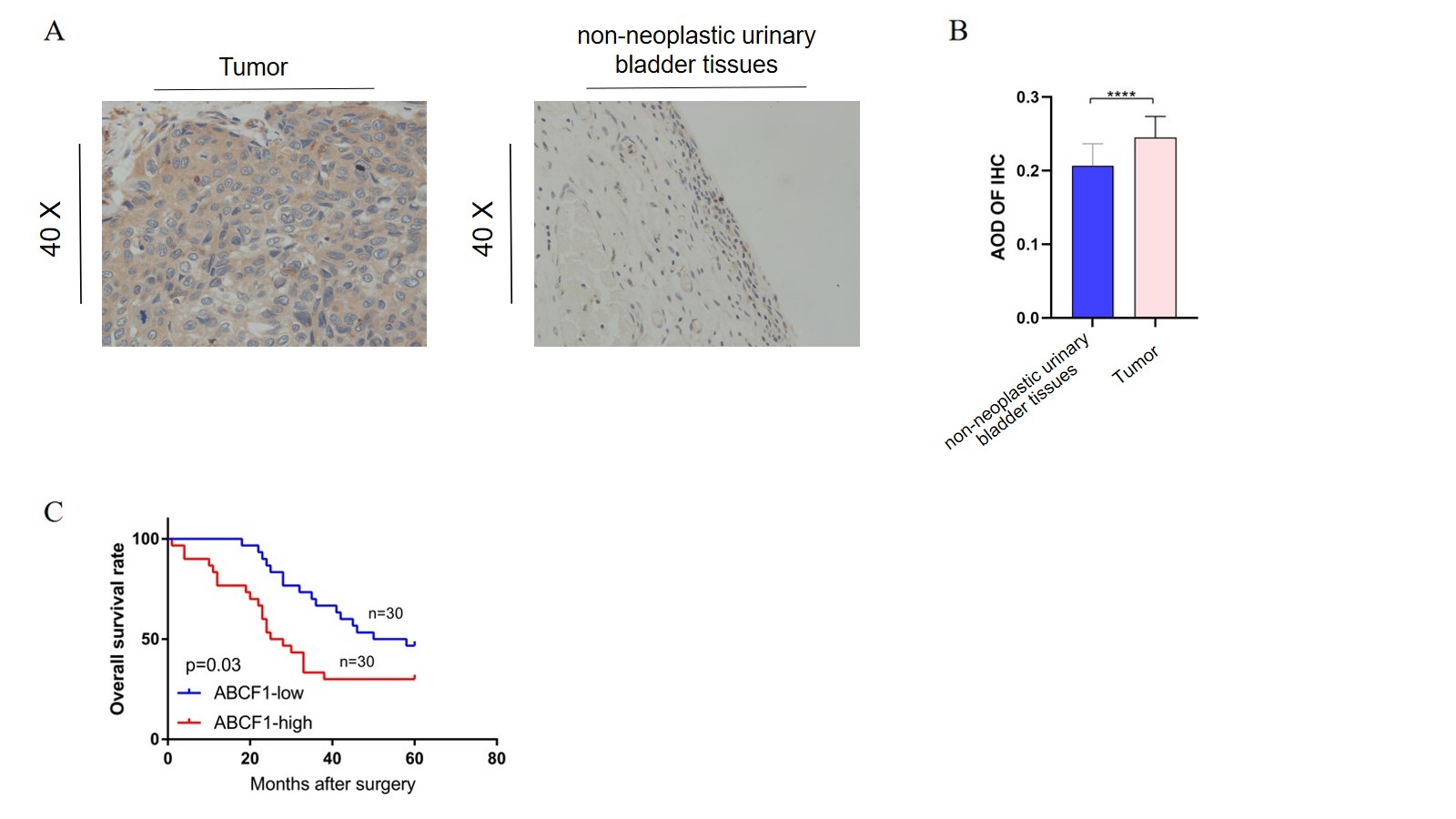


**Figure S8B:The AOD of tumors was significantly higher than that of nonneoplastic urinary bladder samples (P<0.0001).**

| **Table S1:** | | |
| --- | --- | --- |
| Patient number | the AOD of Tumor | The AOD of nonneoplastic urinary bladder samples |
| 1 | 0.242 | 0.18 |
| 2 | 0.225 | 0.186 |
| 3 | 0.323 | 0.202 |
| 4 | 0.299 | 0.189 |
| 5 | 0.314 | 0.194 |
| 6 | 0.244 | 0.213 |
| 7 | 0.248 | 0.192 |
| 8 | 0.263 | 0.2 |
| 9 | 0.271 | 0.202 |
| 10 | 0.237 | 0.184 |
| 11 | 0.242 | 0.17 |
| 12 | 0.25 | 0.218 |
| 13 | 0.276 | 0.209 |
| 14 | 0.235 | 0.188 |
| 15 | 0.24 | 0.215 |
| 16 | 0.253 | 0.245 |
| 17 | 0.259 | 0.204 |
| 18 | 0.237 | 0.2 |
| 19 | 0.226 | 0.19 |
| 20 | 0.241 | 0.238 |
| 21 | 0.225 | 0.205 |
| 22 | 0.286 | 0.196 |
| 23 | 0.269 | 0.192 |
| 24 | 0.256 | 0.196 |
| 25 | 0.237 | 0.232 |
| 26 | 0.249 | 0.206 |
| 27 | 0.243 | 0.206 |
| 28 | 0.245 | 0.198 |
| 29 | 0.269 | 0.209 |
| 30 | 0.274 | 0.207 |
| 31 | 0.218 | 0.195 |
| 32 | 0.258 | 0.208 |
| 33 | 0.231 | 0.216 |
| 34 | 0.254 | 0.208 |
| 35 | 0.218 | 0.195 |
| 36 | 0.208 | 0.22 |
| 37 | 0.223 | 0.199 |
| 38 | 0.228 | 0.176 |
| 39 | 0.218 | 0.17 |
| 40 | 0.218 | 0.215 |
| 41 | 0.258 | 0.188 |
| 42 | 0.214 | 0.198 |
| 43 | 0.194 | 0.194 |
| 44 | 0.215 | 0.225 |
| 45 | 0.277 | 0.196 |
| 46 | 0.23 | 0.217 |
| 47 | 0.236 | 0.218 |
| 48 | 0.294 | 0.225 |
| 49 | 0.309 | 0.199 |
| 50 | 0.25 | 0.184 |
| 51 | 0.217 | 0.163 |
| 52 | 0.248 | 0.162 |
| 53 | 0.224 | 0.21 |
| 54 | 0.212 | 0.205 |
| 55 | 0.243 | 0.181 |
| 56 | 0.24 | 0.225 |
| 57 | 0.207 | 0.356 |
| 58 | 0.218 | 0.245 |
| 59 | 0.201 | 0.314 |
| 60 | 0.197 | 0.253 |

**Table S1: The AOD of immunohistochemical**
