# Supplementary figures and images for: High expression of ABCF1 is an independent predictor of poor prognosis in bladder cancer
Source: BMC Urol. 2023 Mar 17;23:37. doi: 10.1186/s12894-023-01211-y (PMC10022215; doi:10.1186/s12894-023-01211-y)

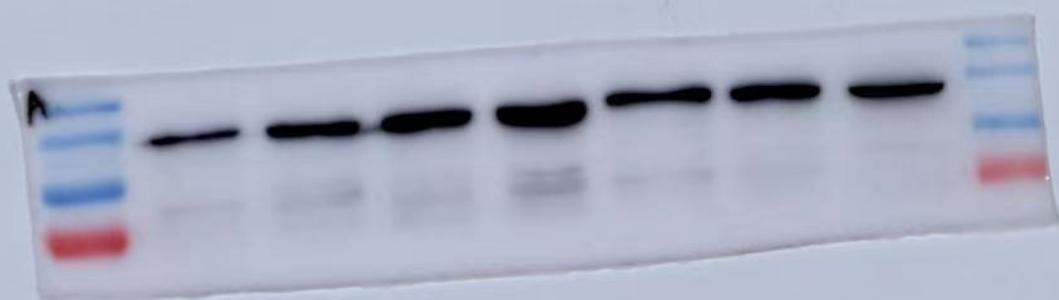

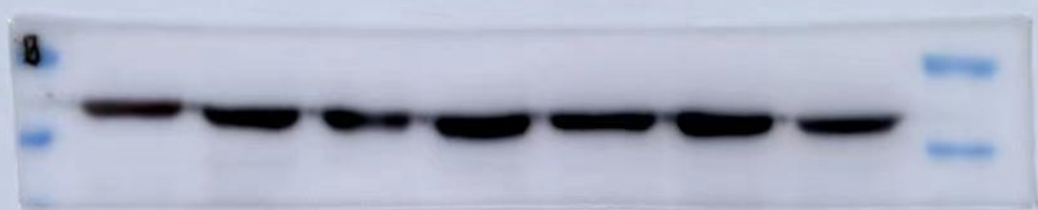

Supplement: Supplementary file 3 — Additional file 3. WB：the result of Western blotting [file 12894_2023_1211_MOESM3_ESM.pdf]

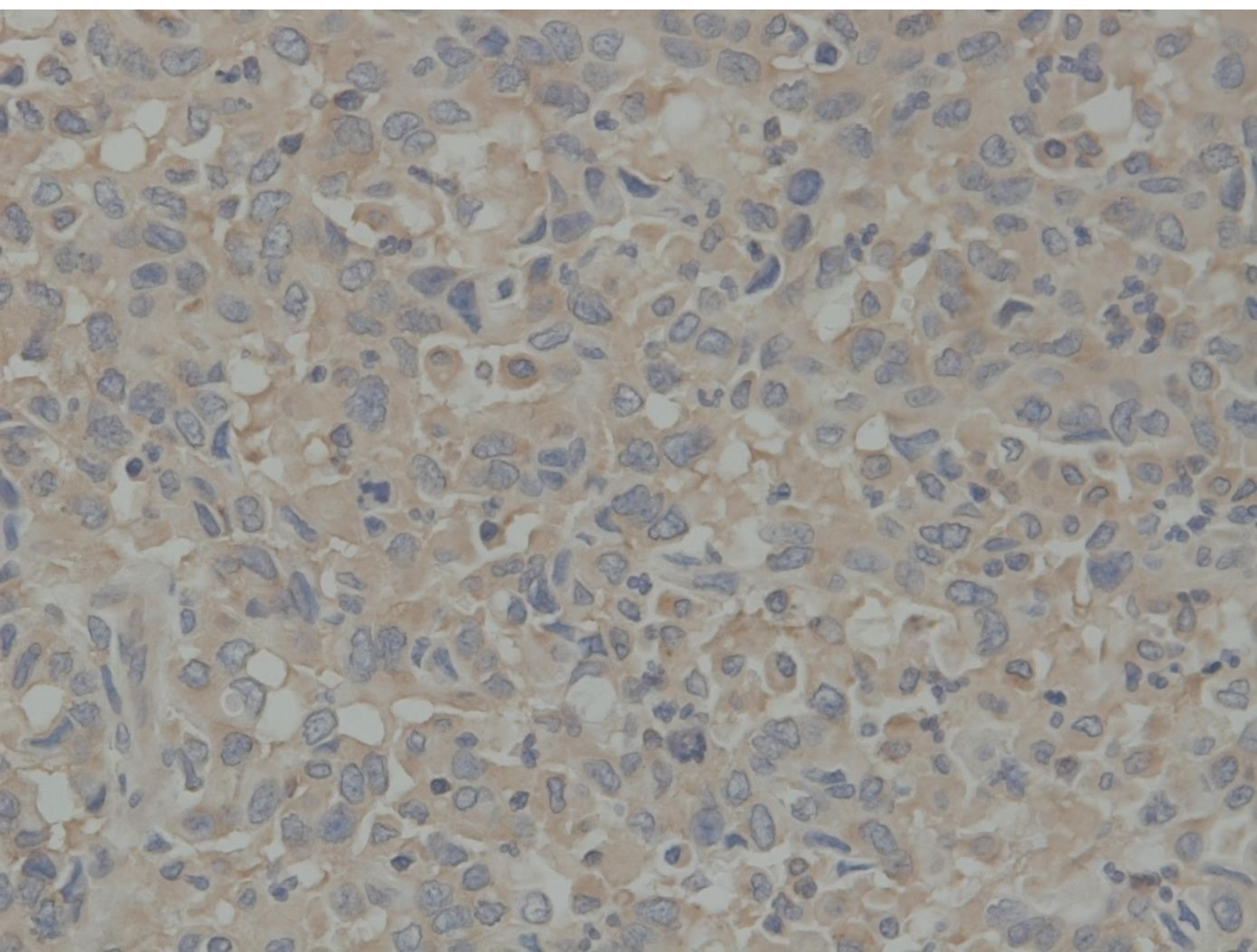

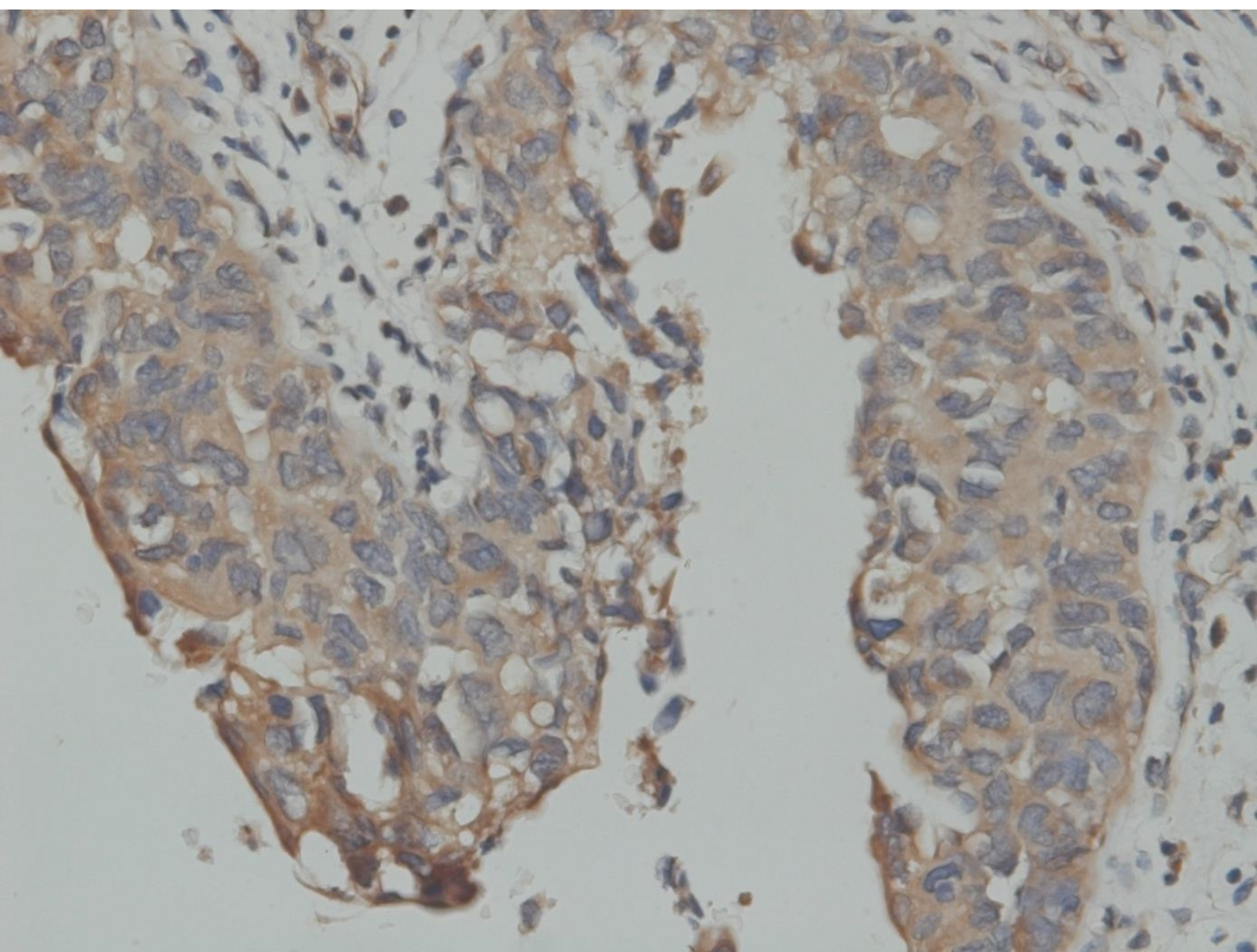

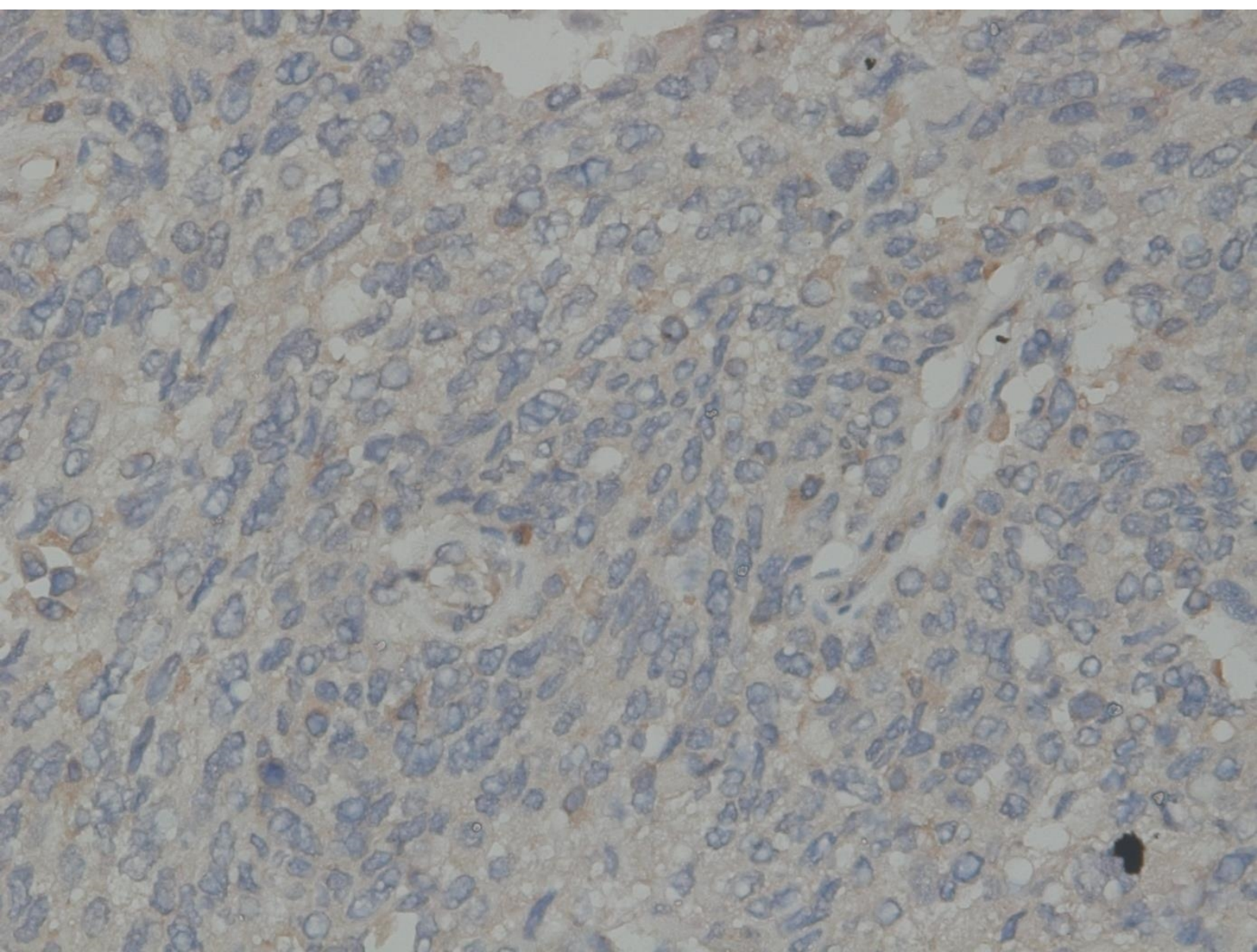

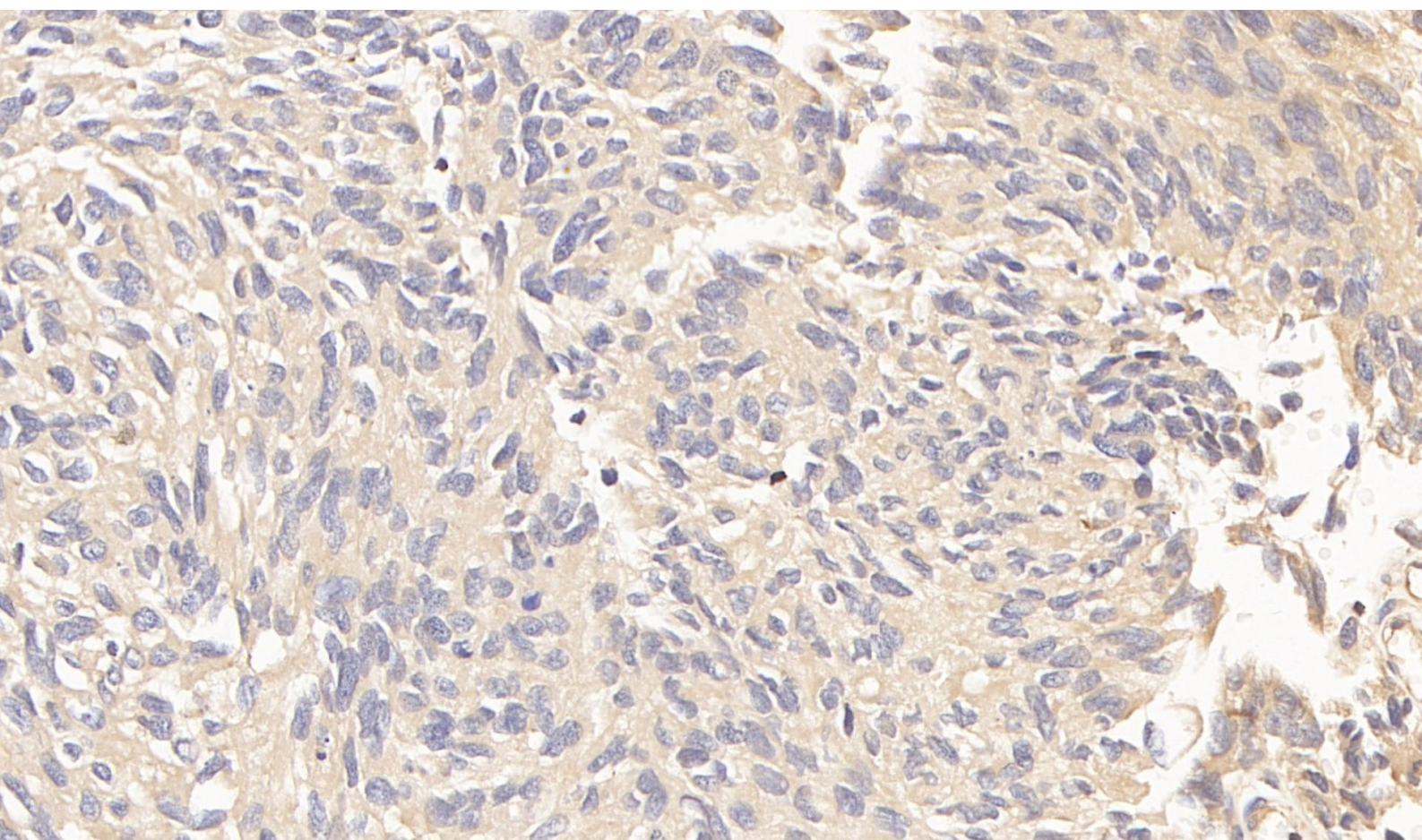

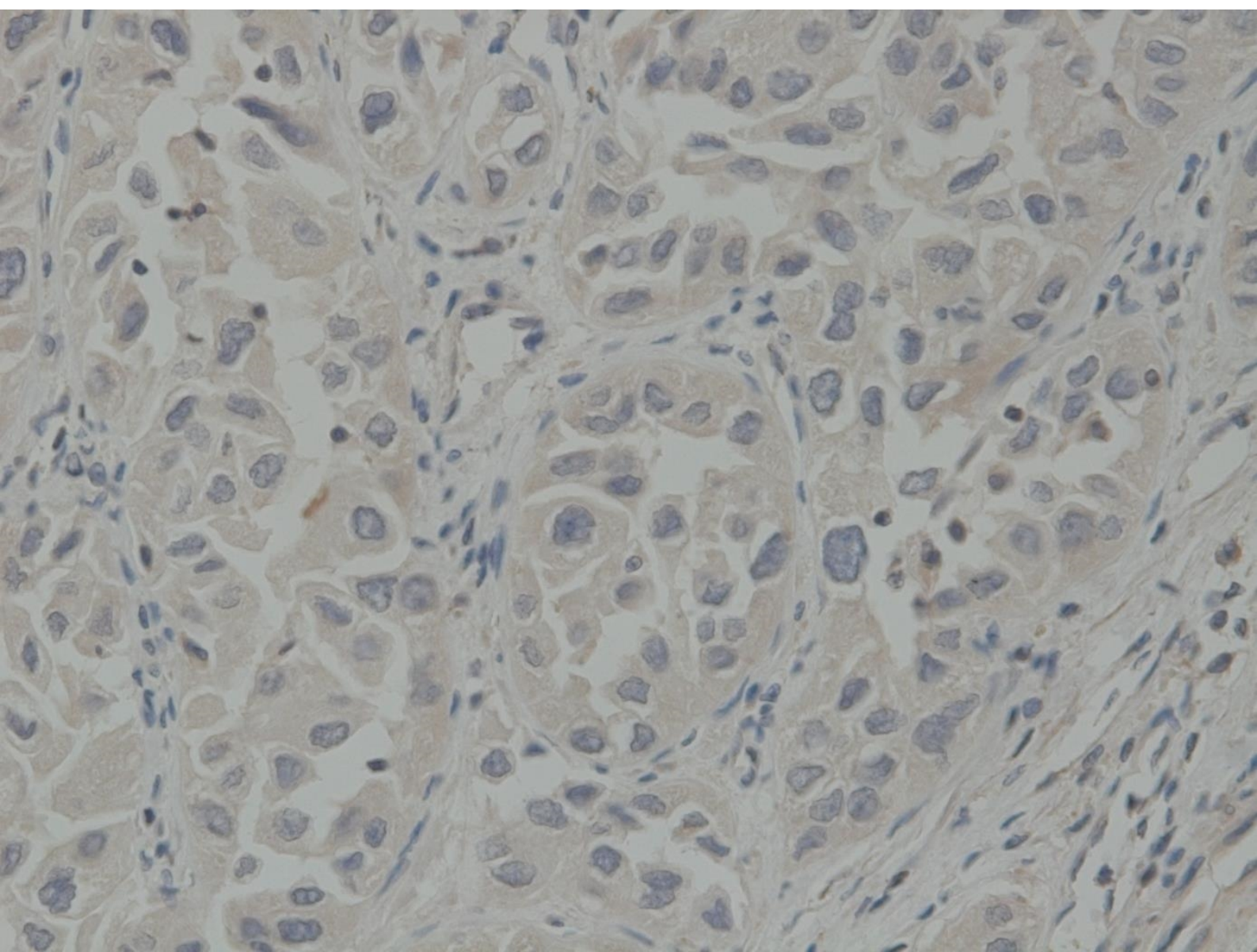

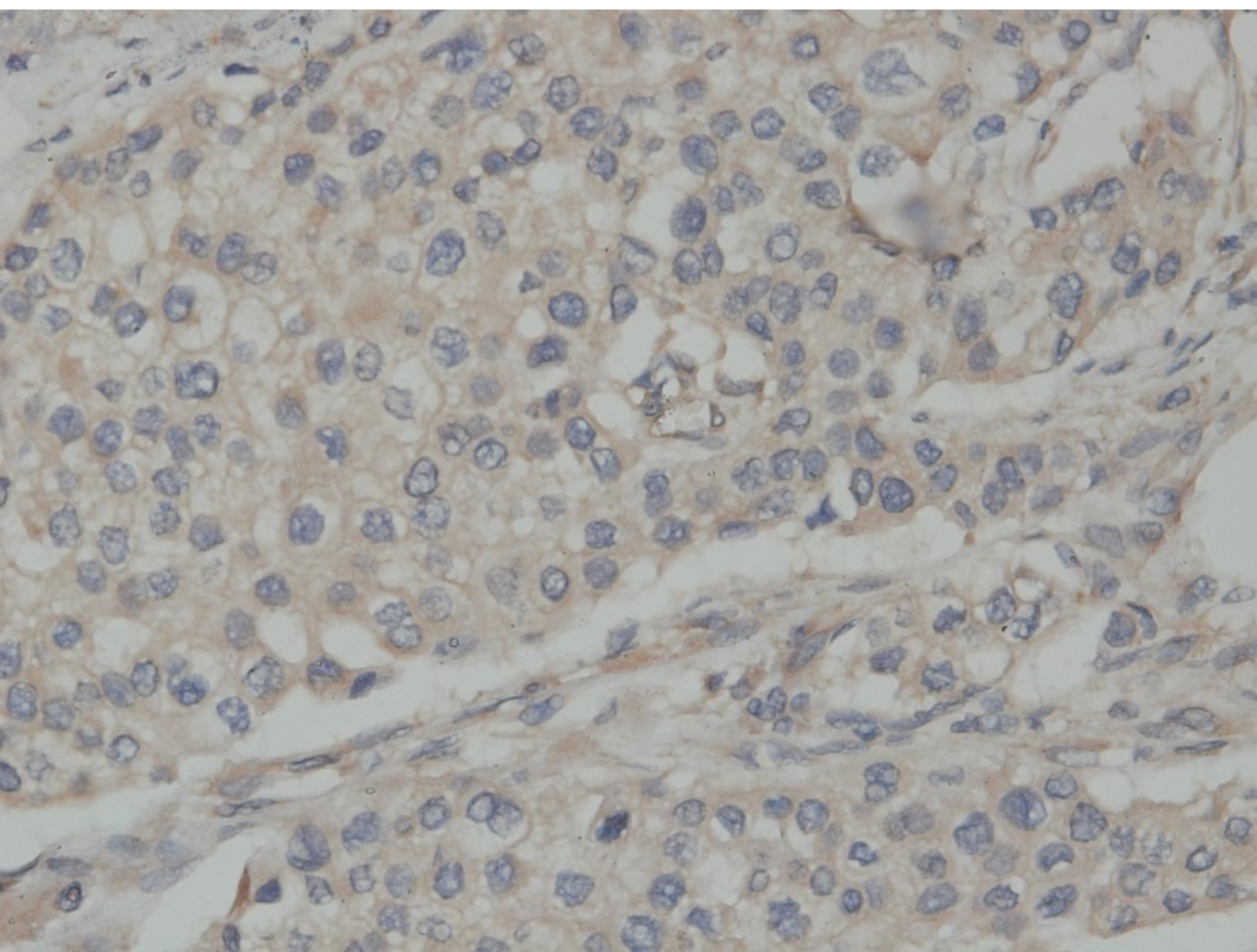

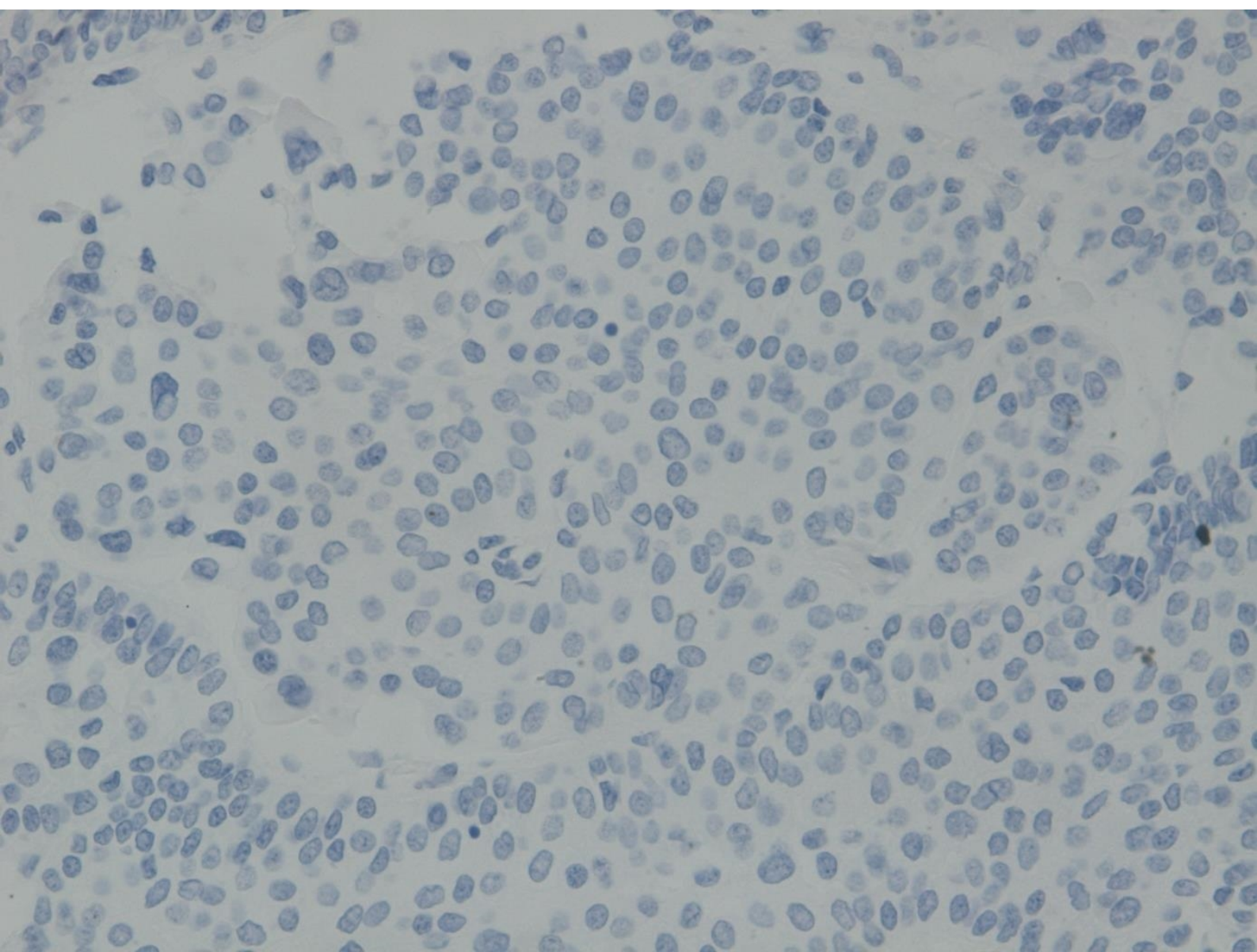

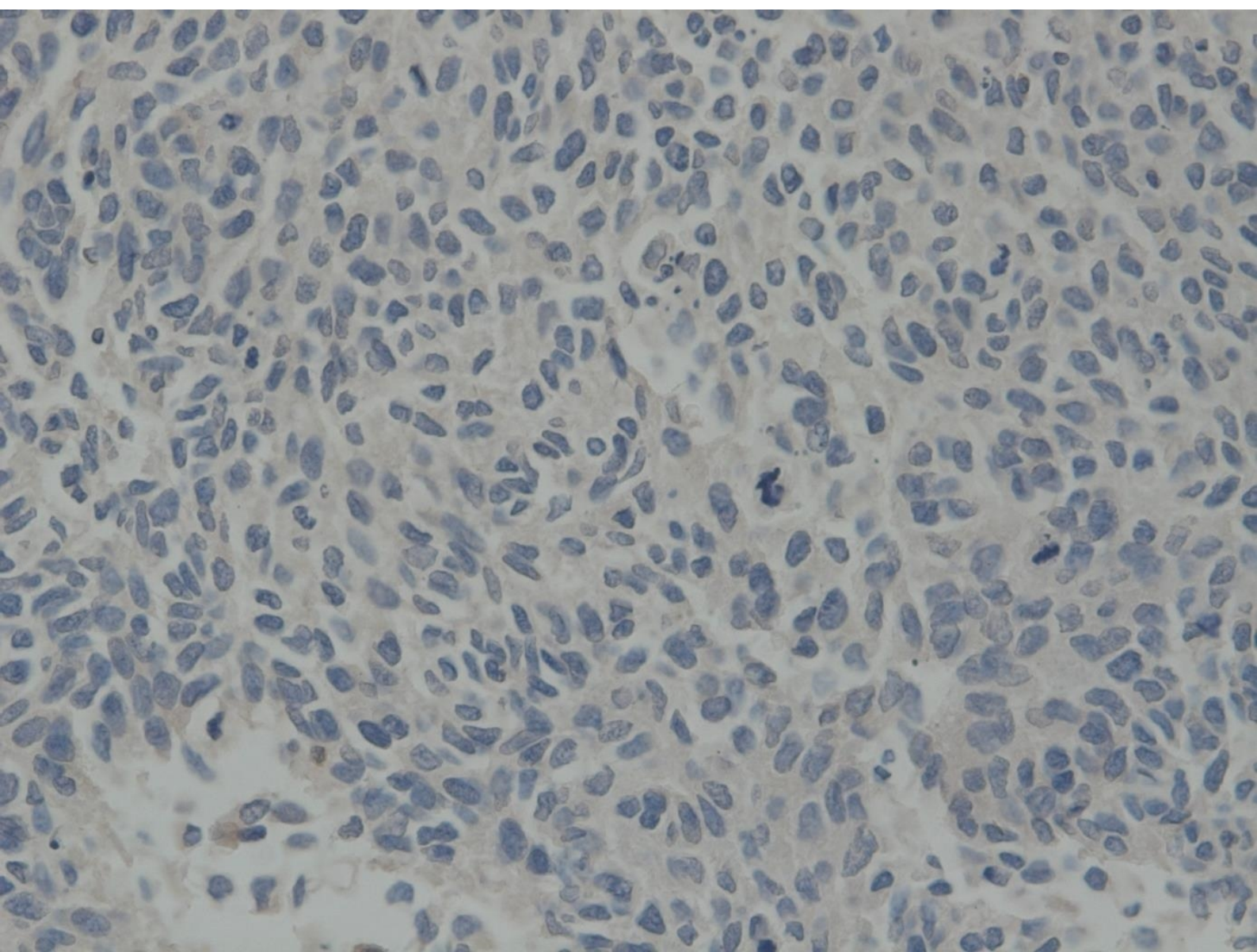

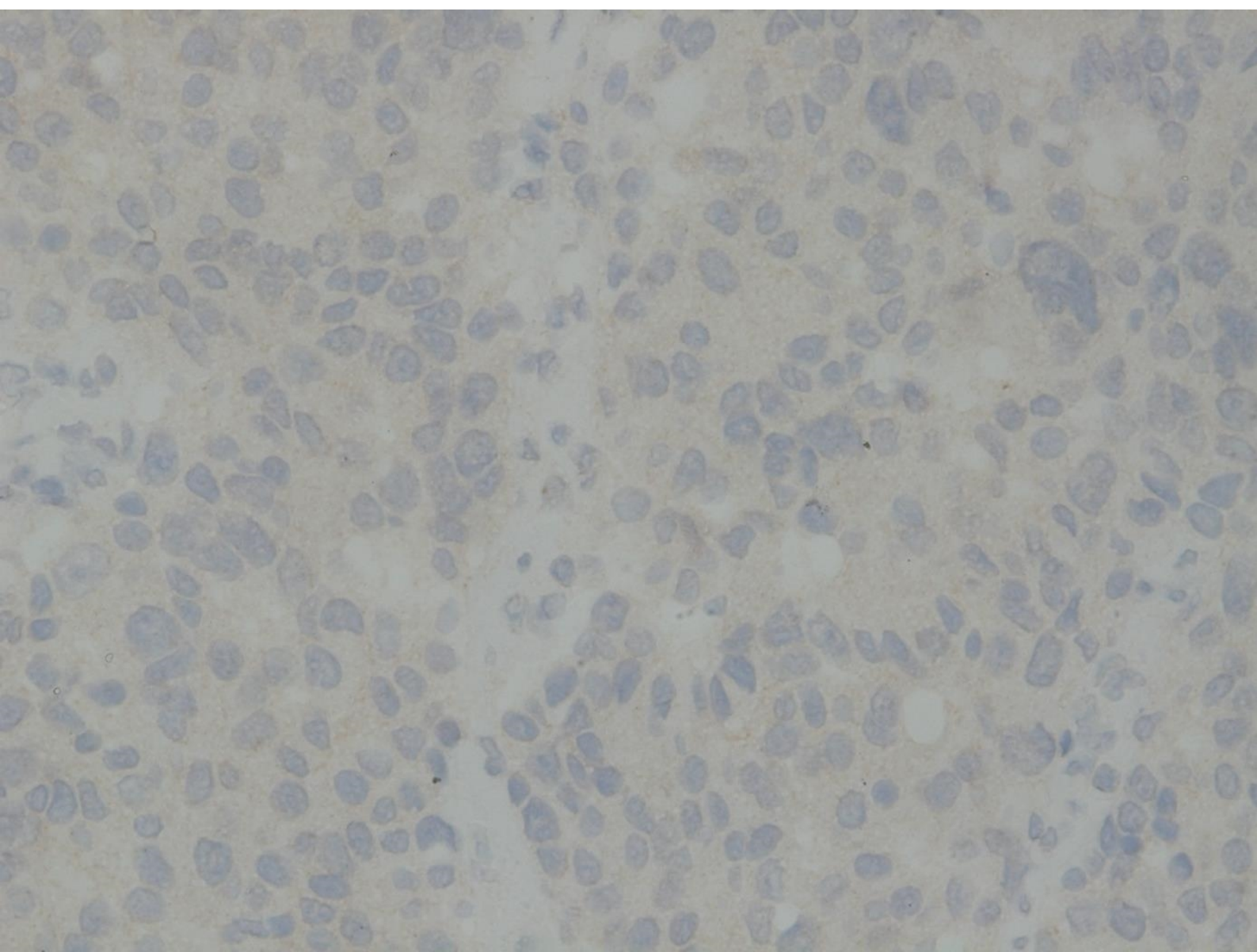

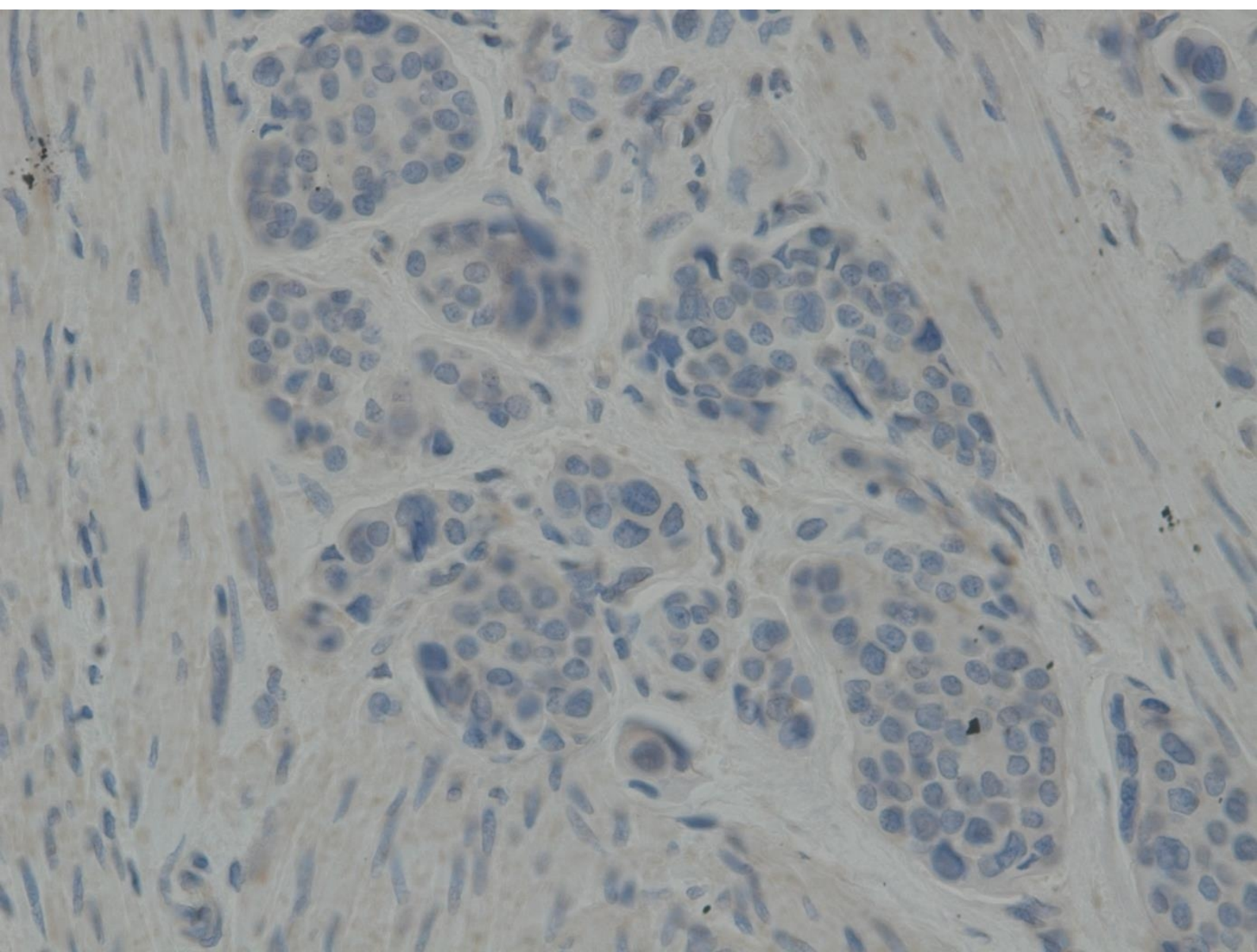

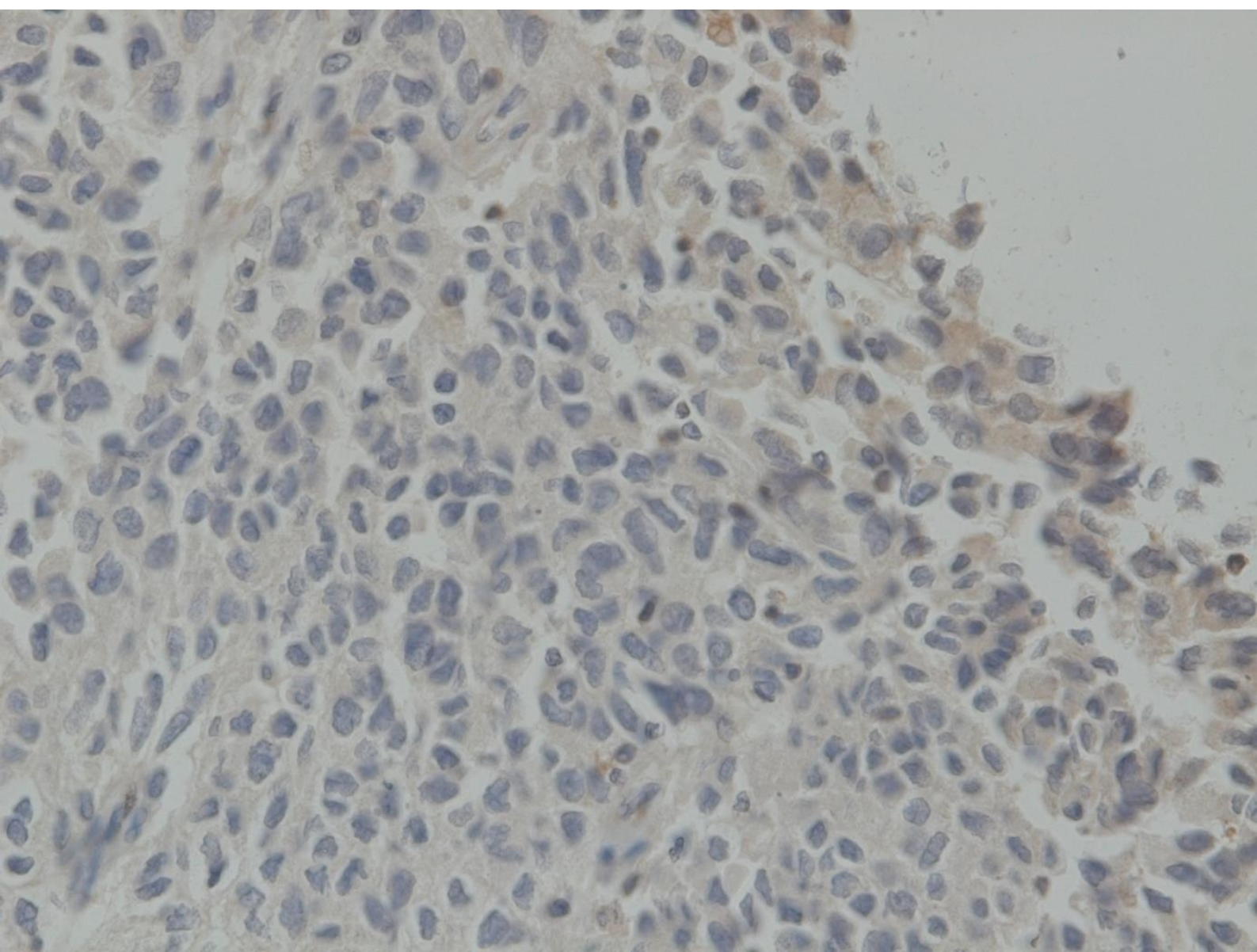

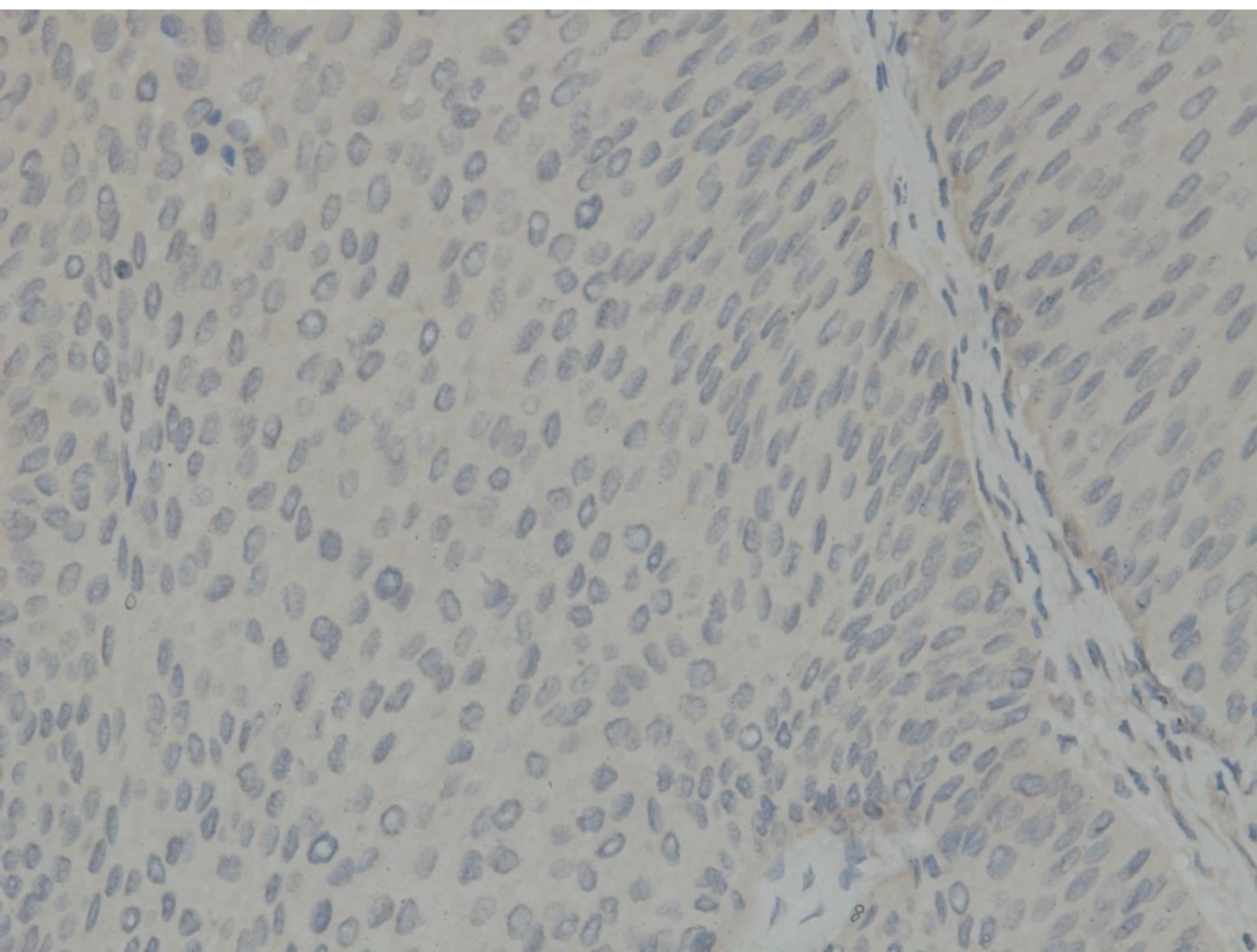

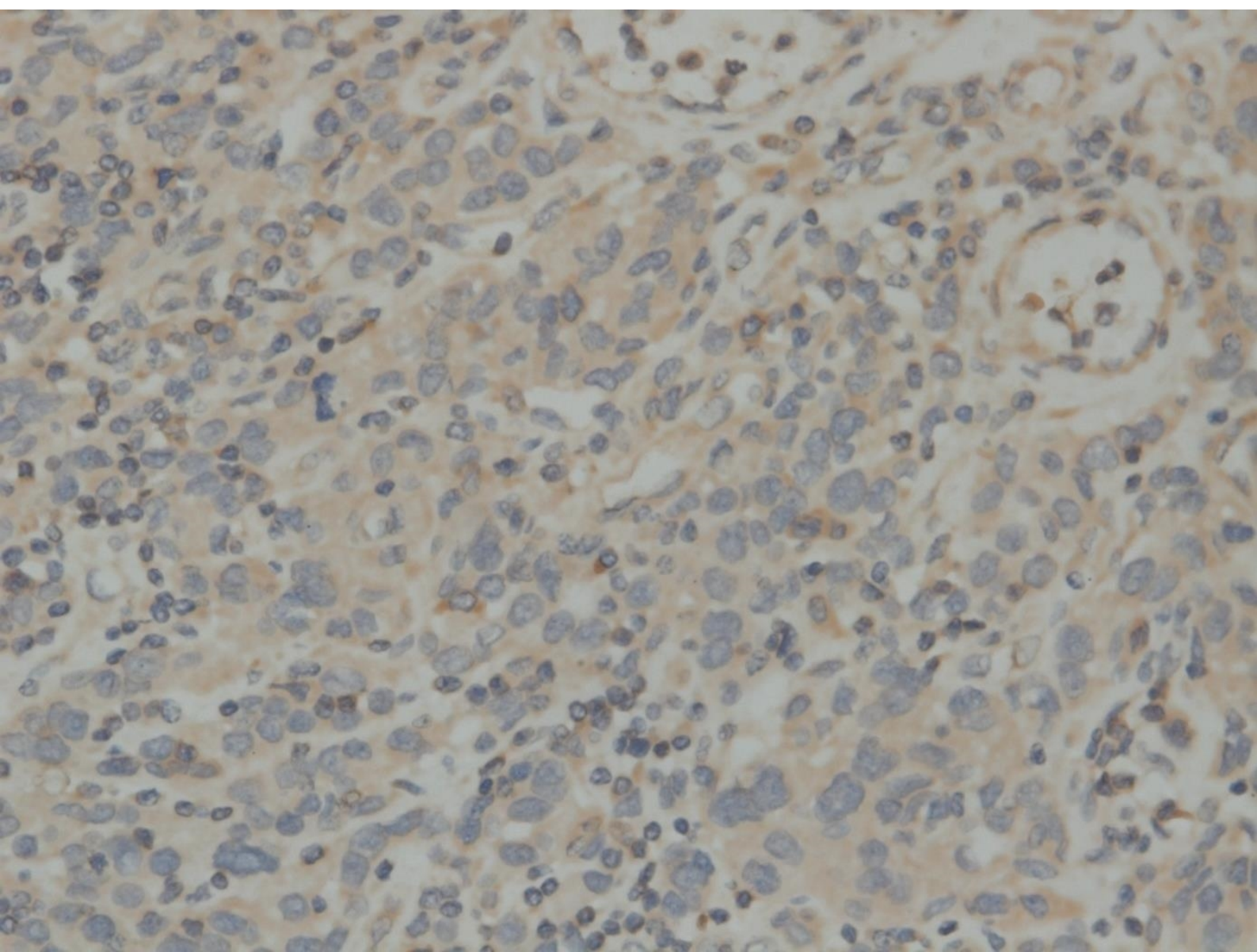

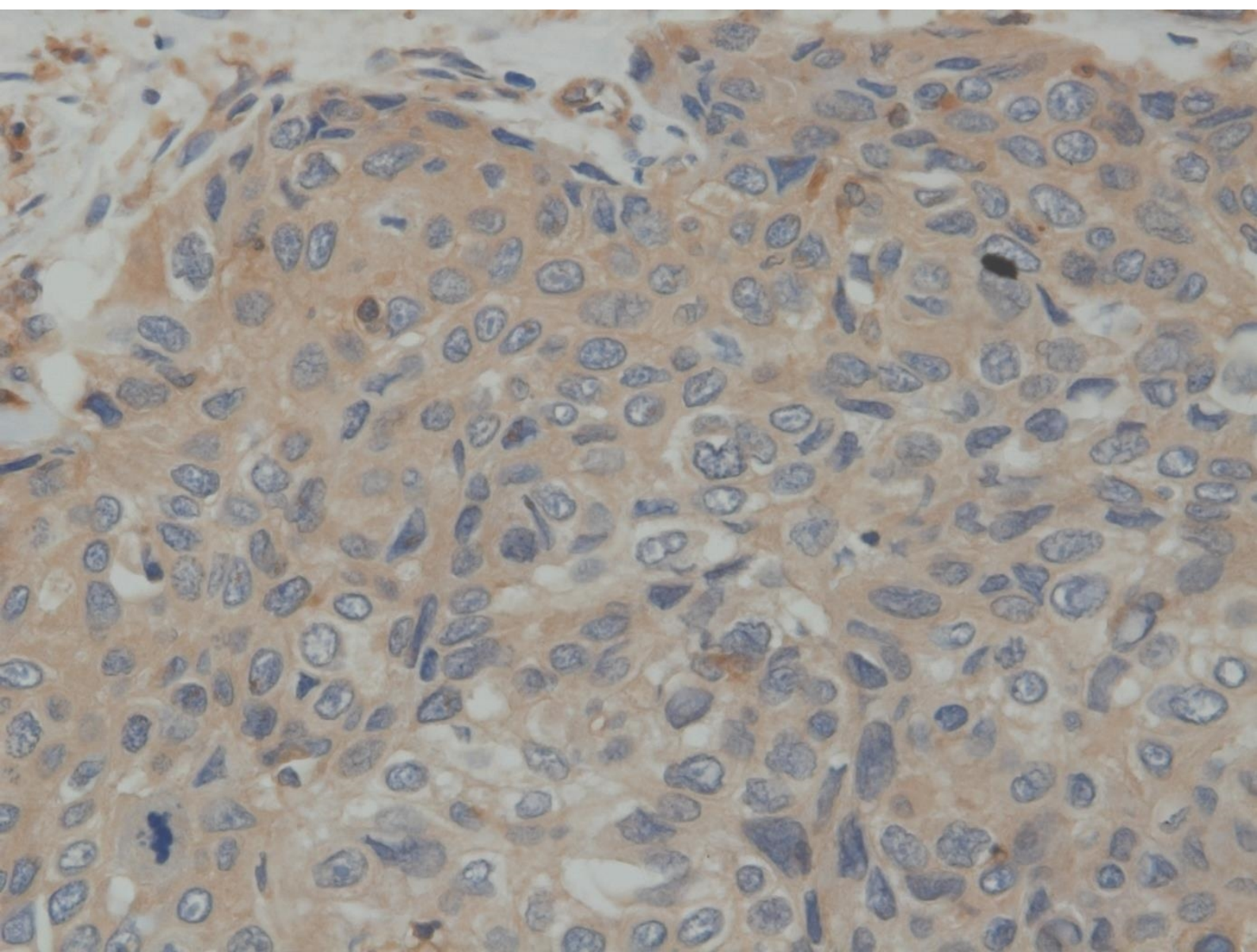

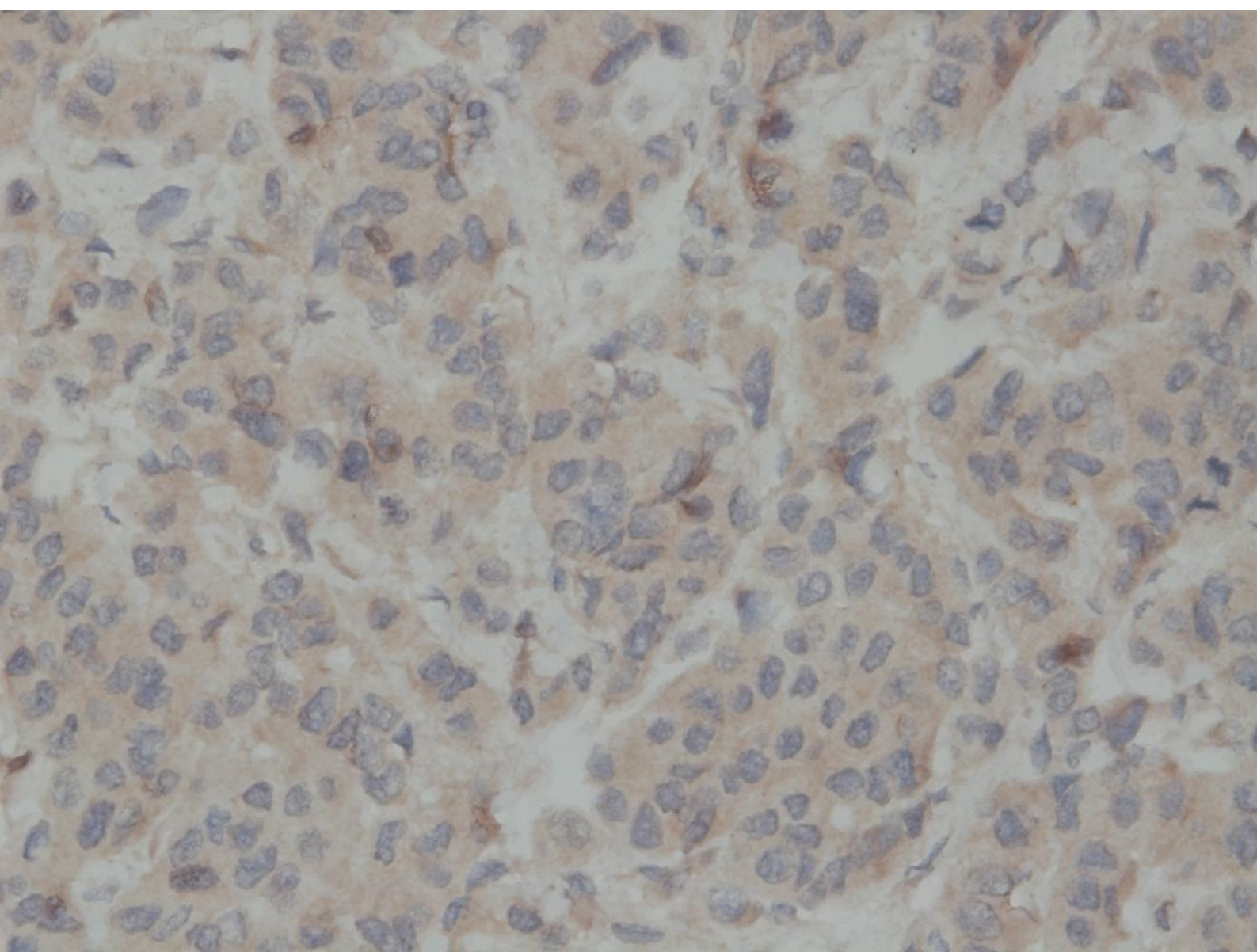

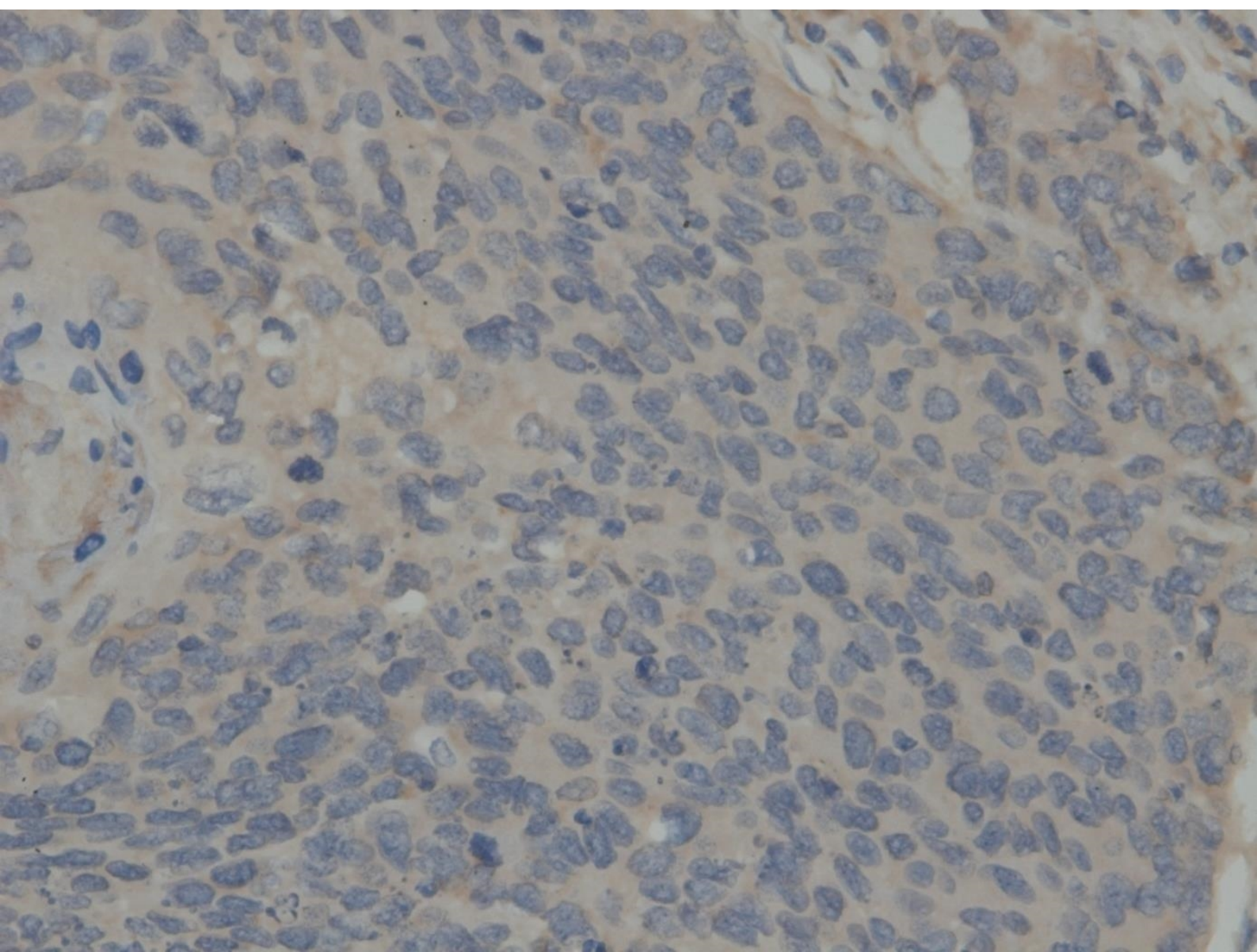

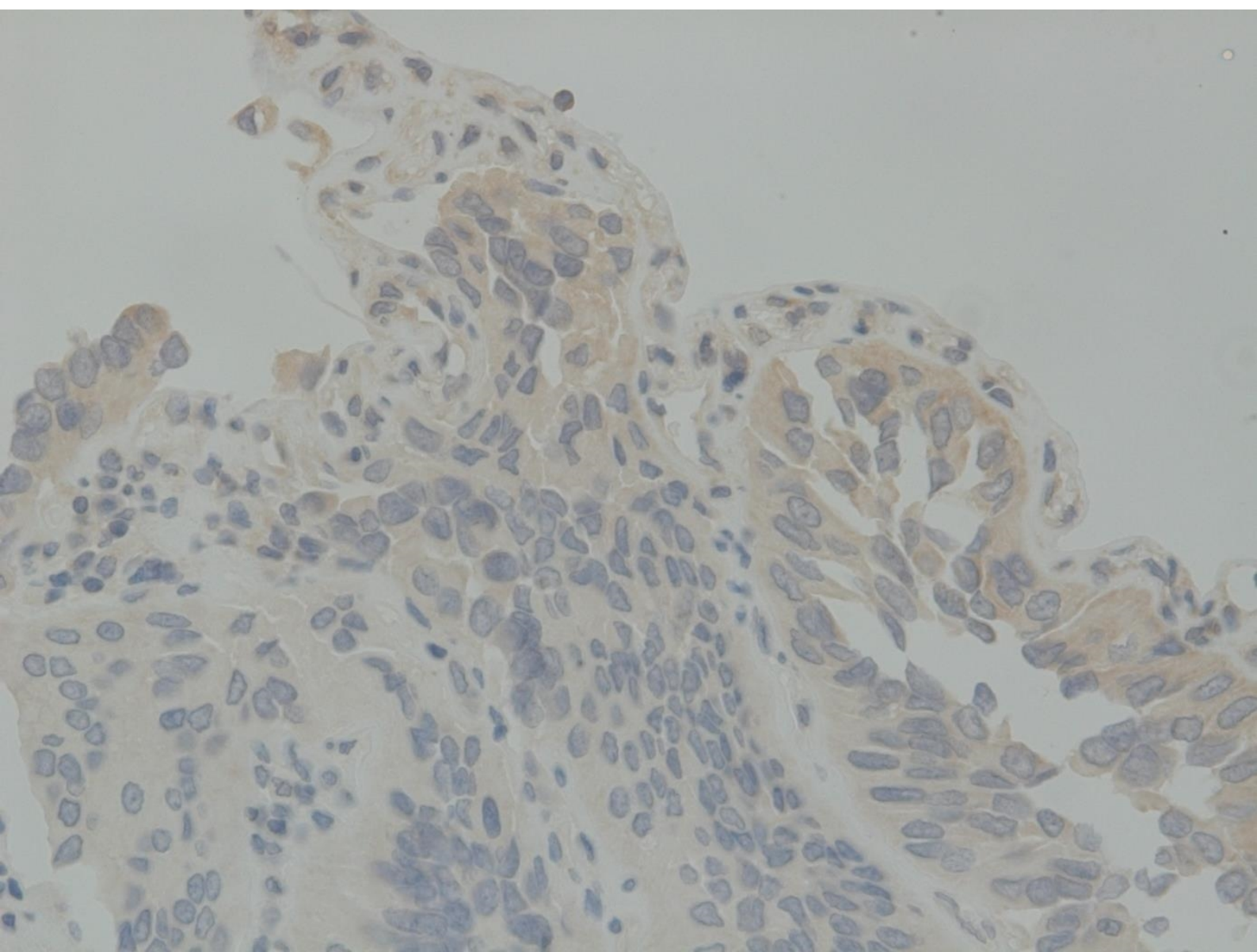

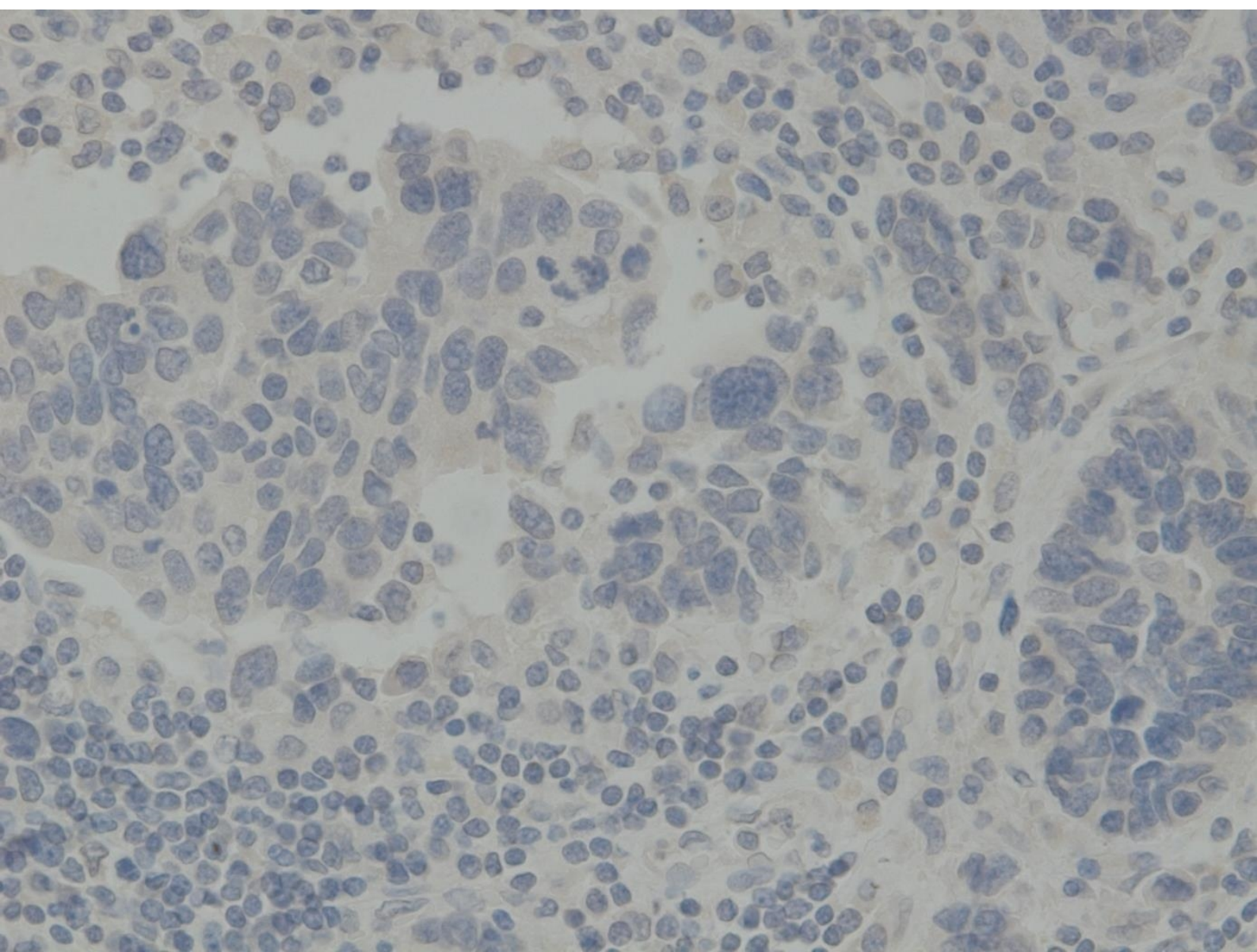

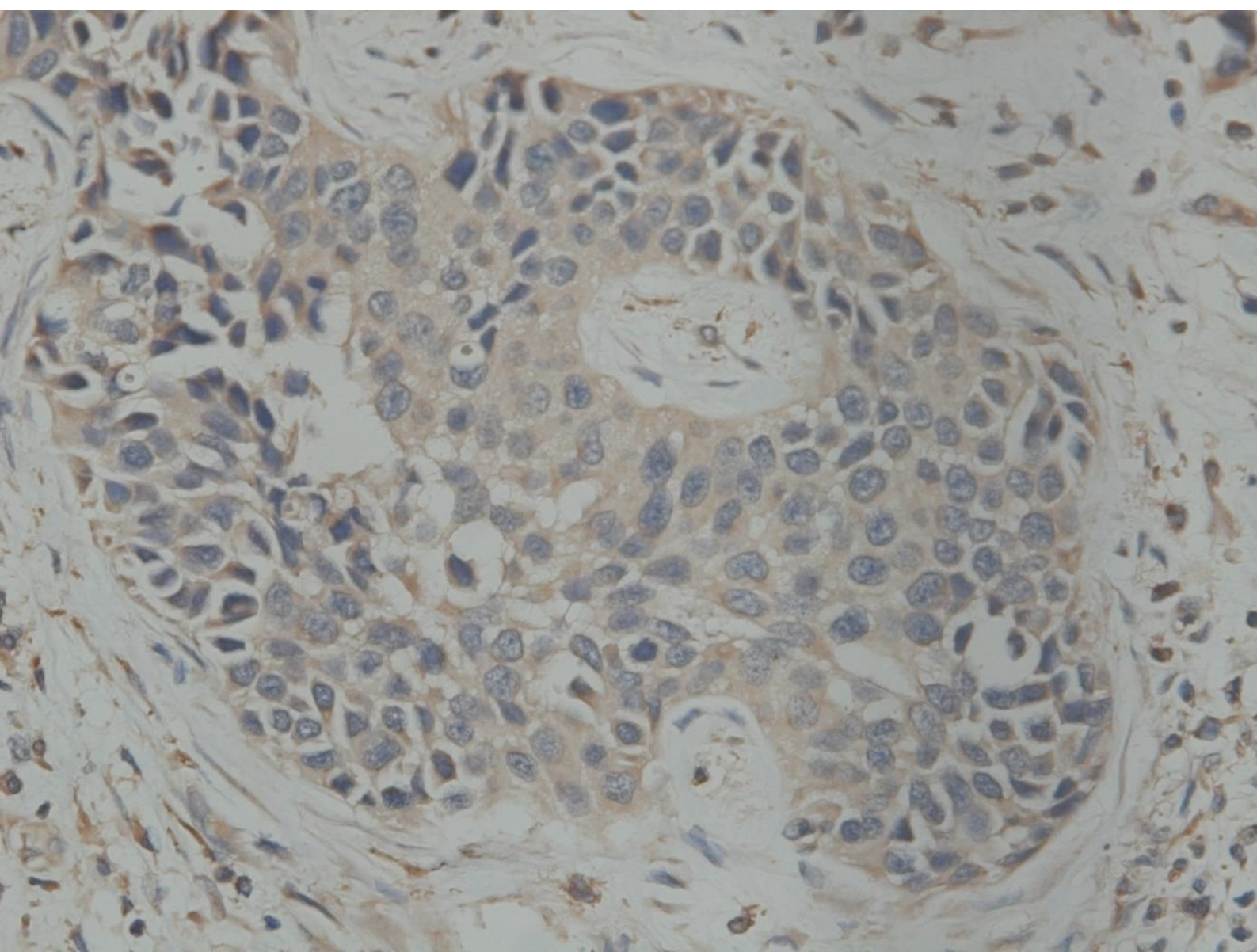

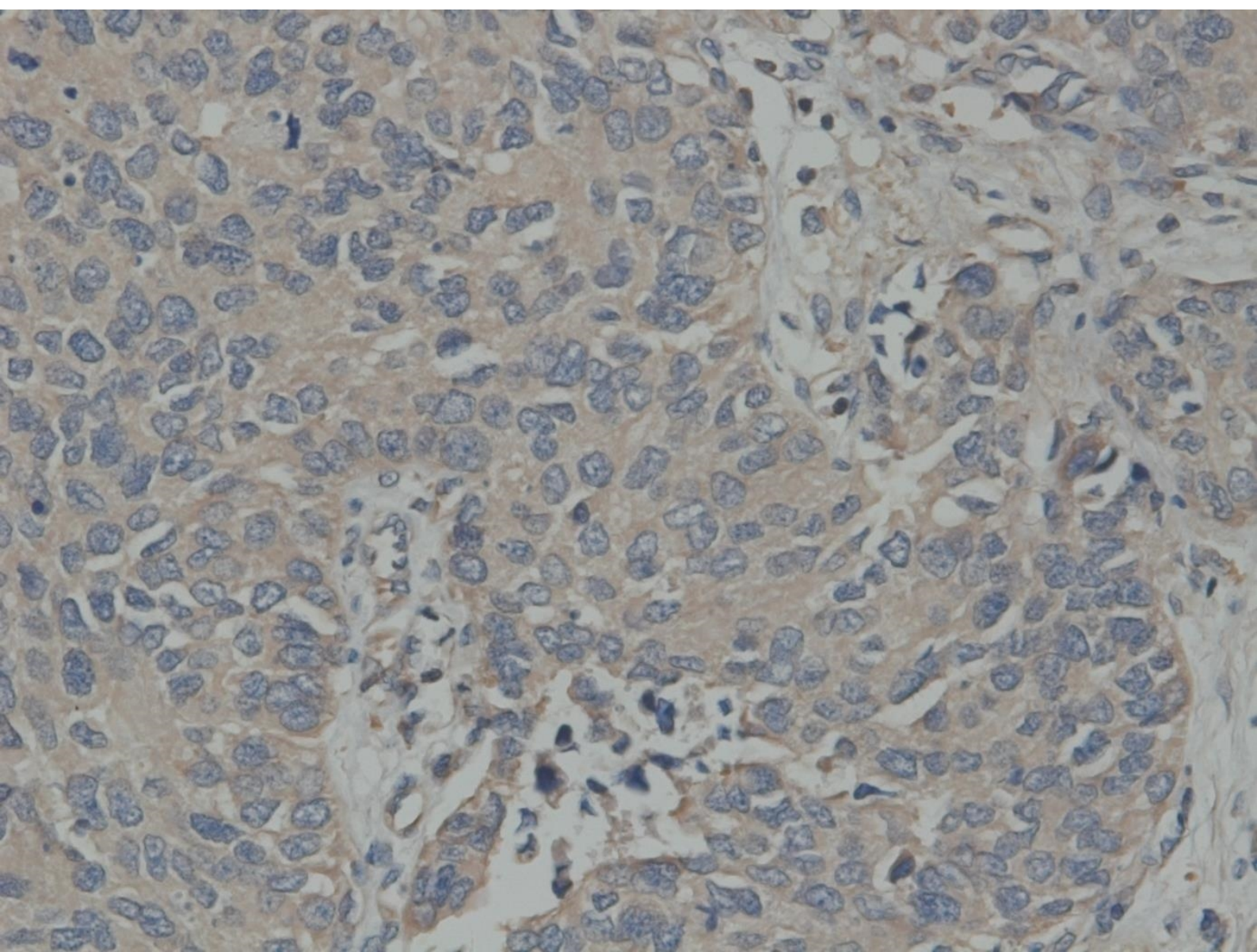

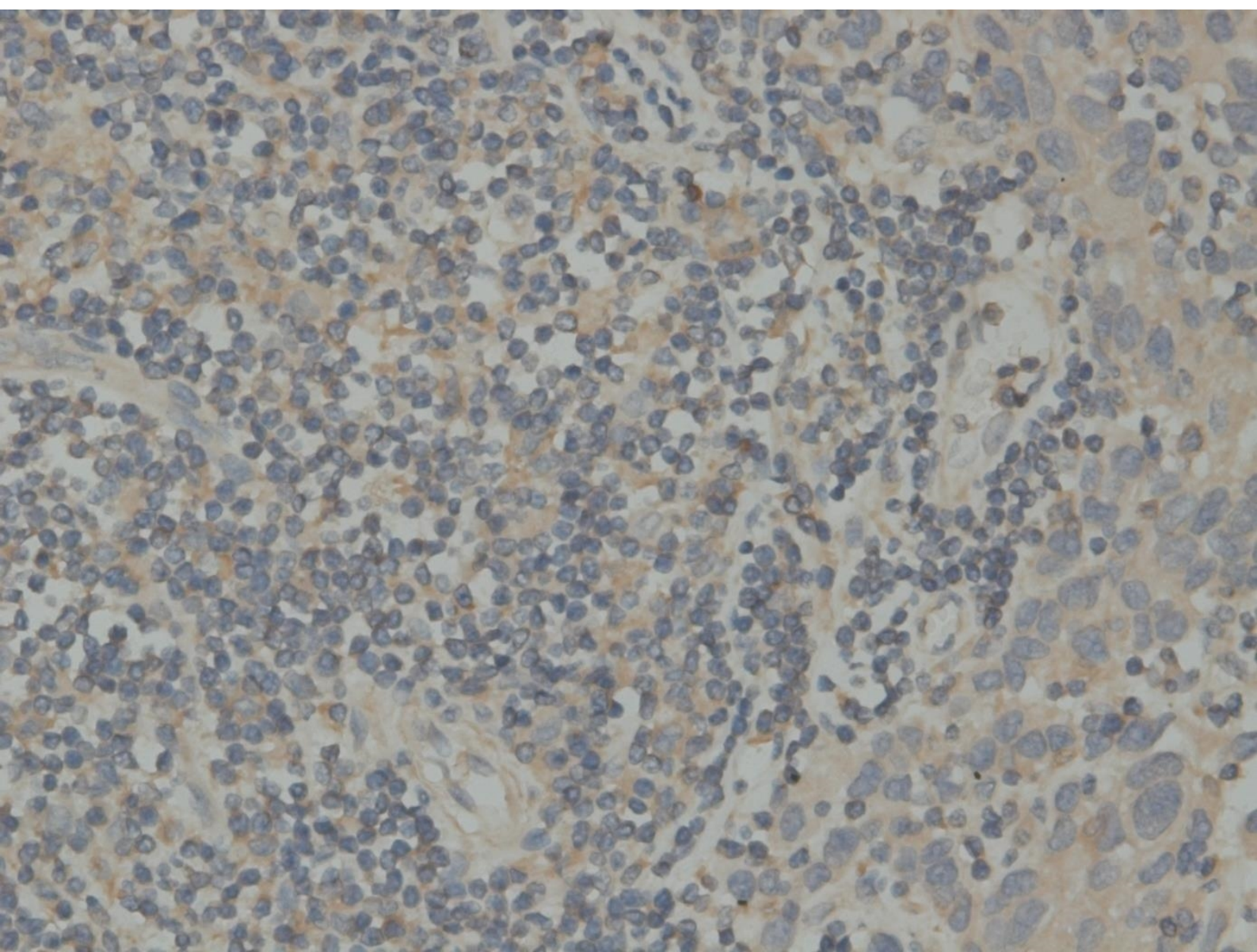

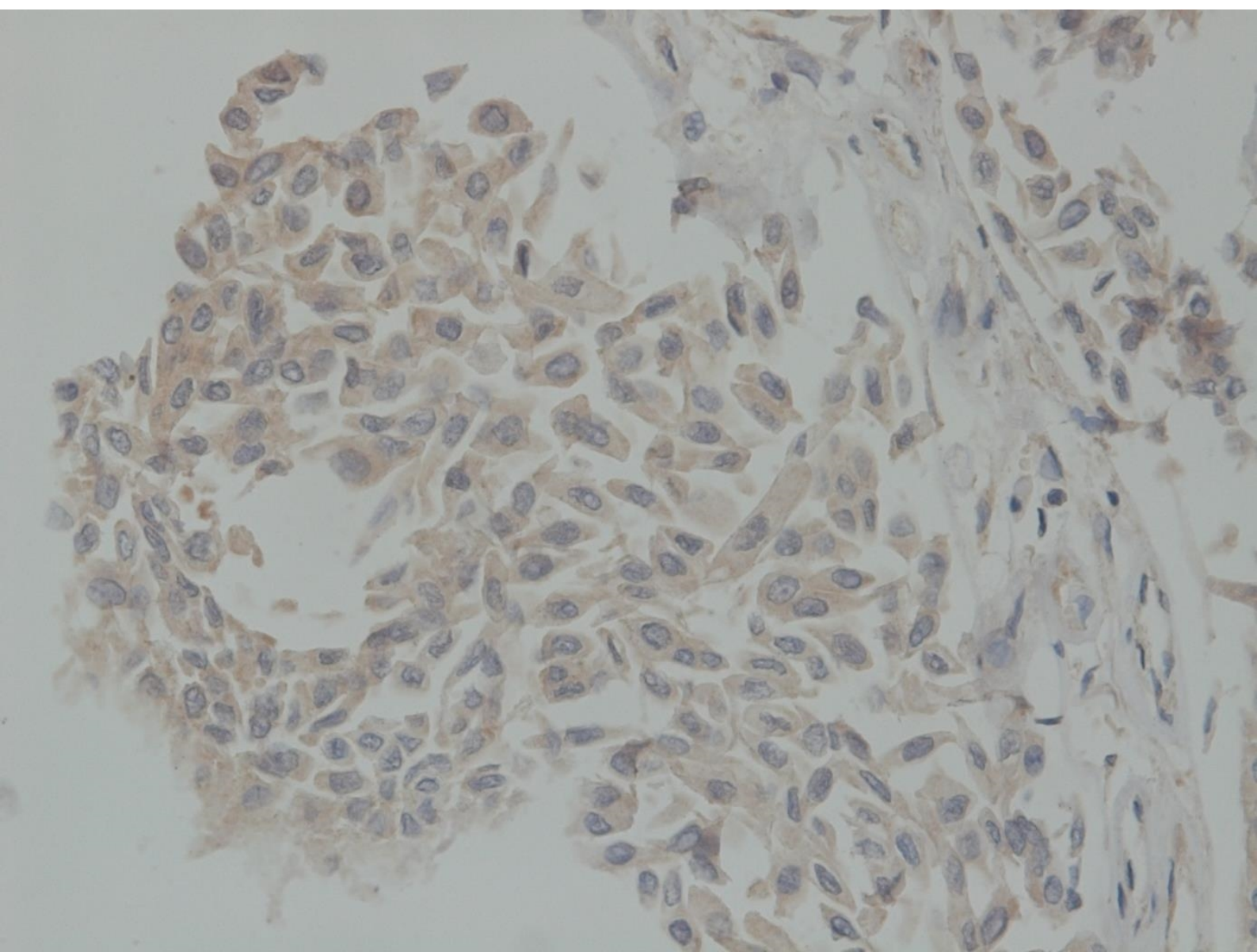

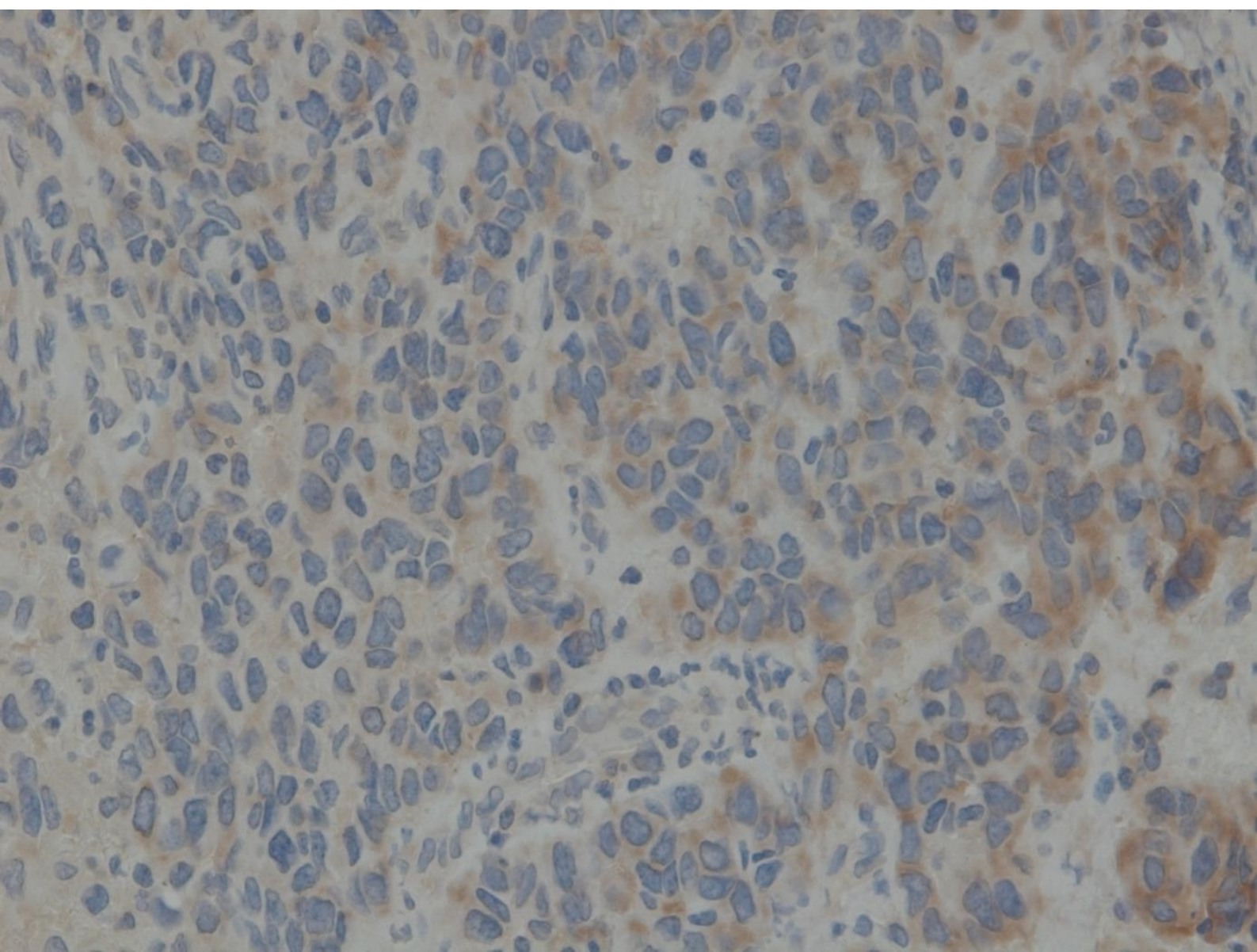

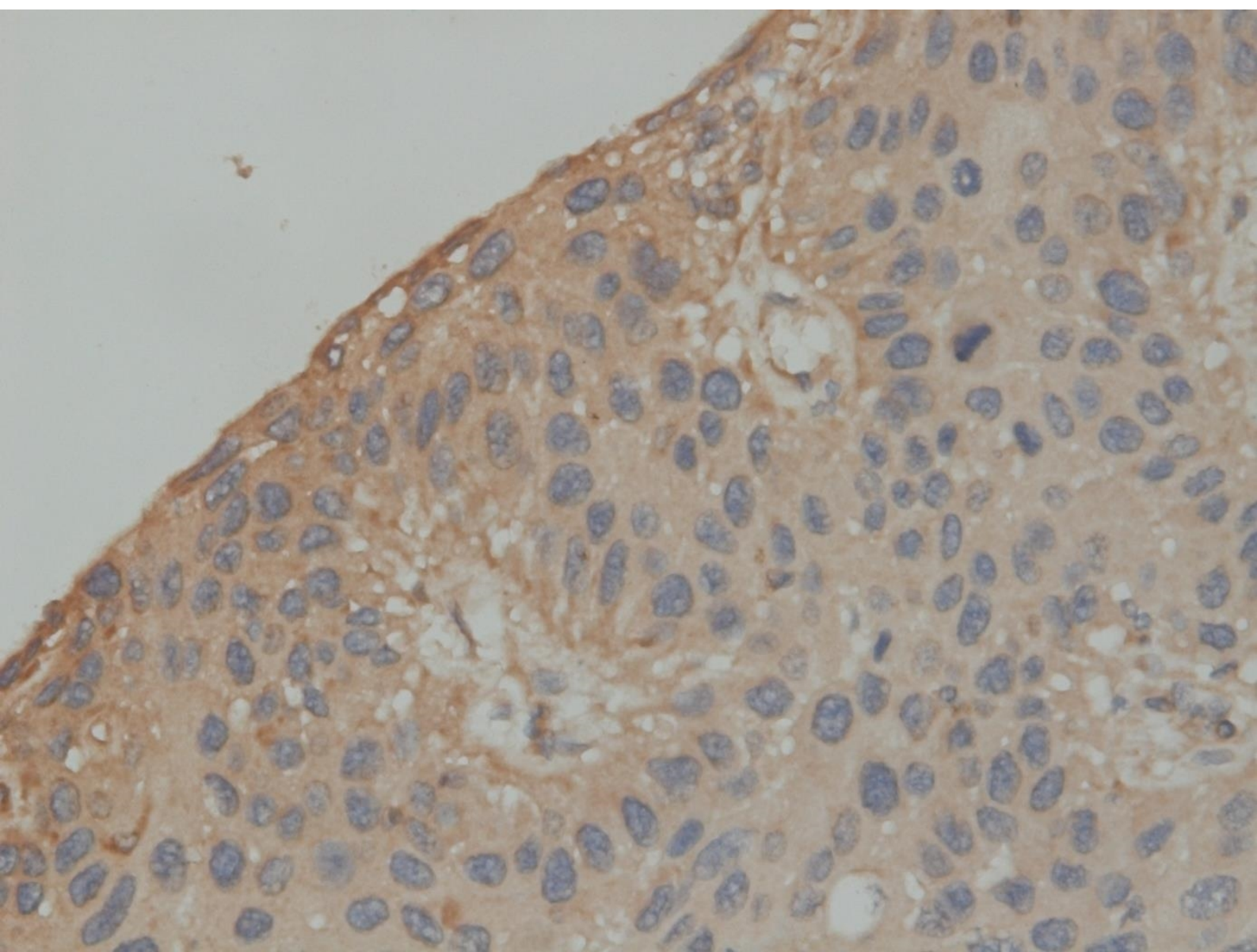

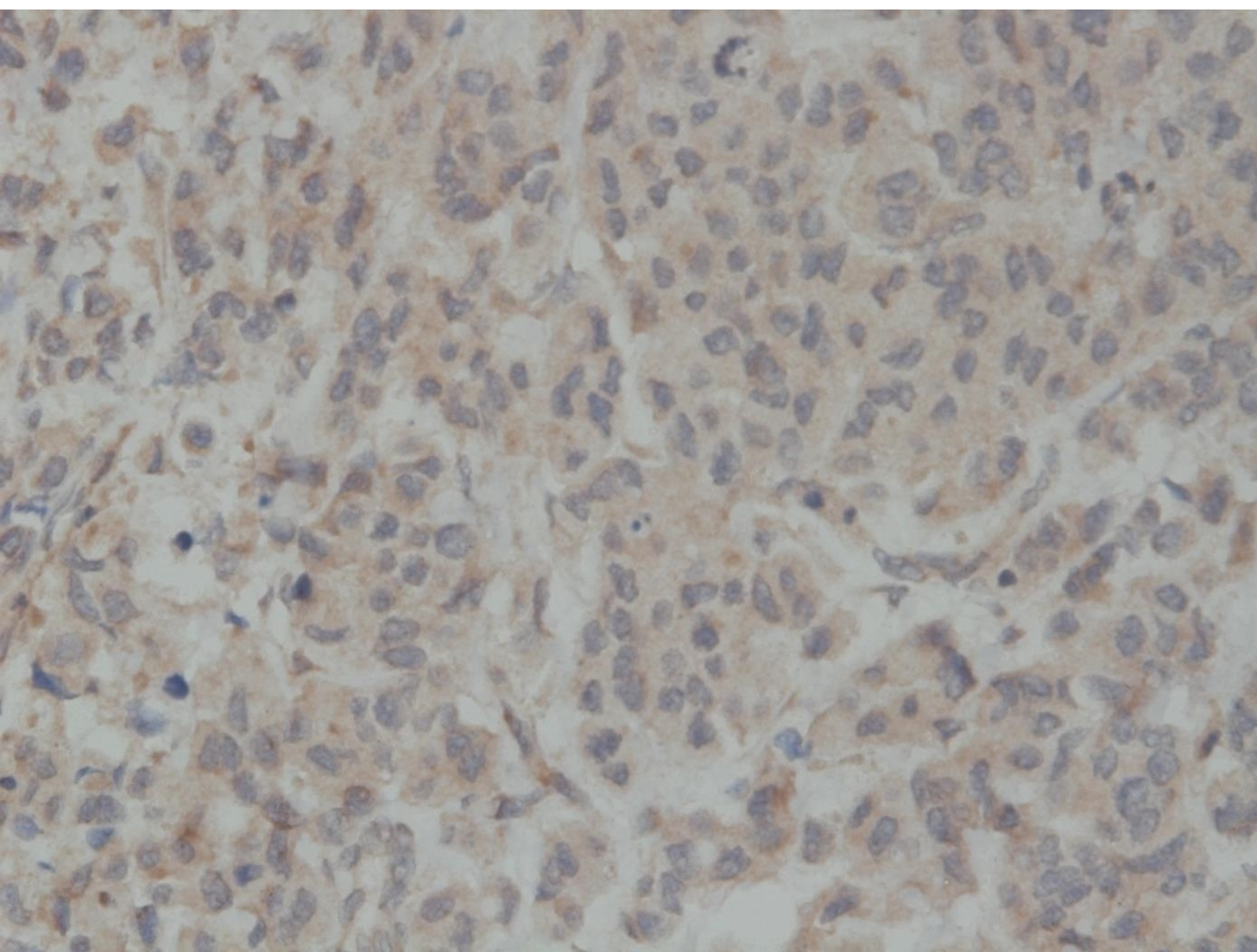

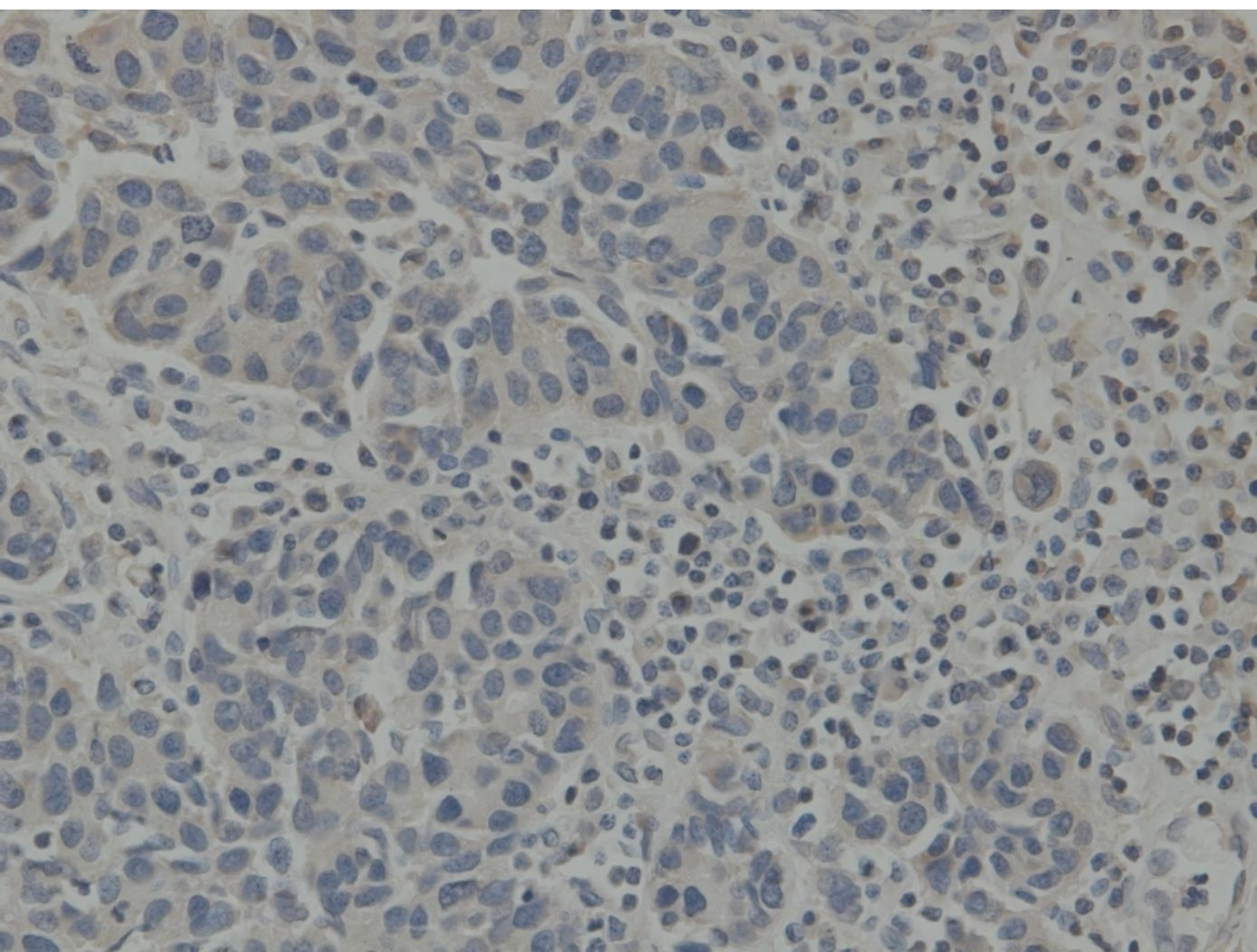

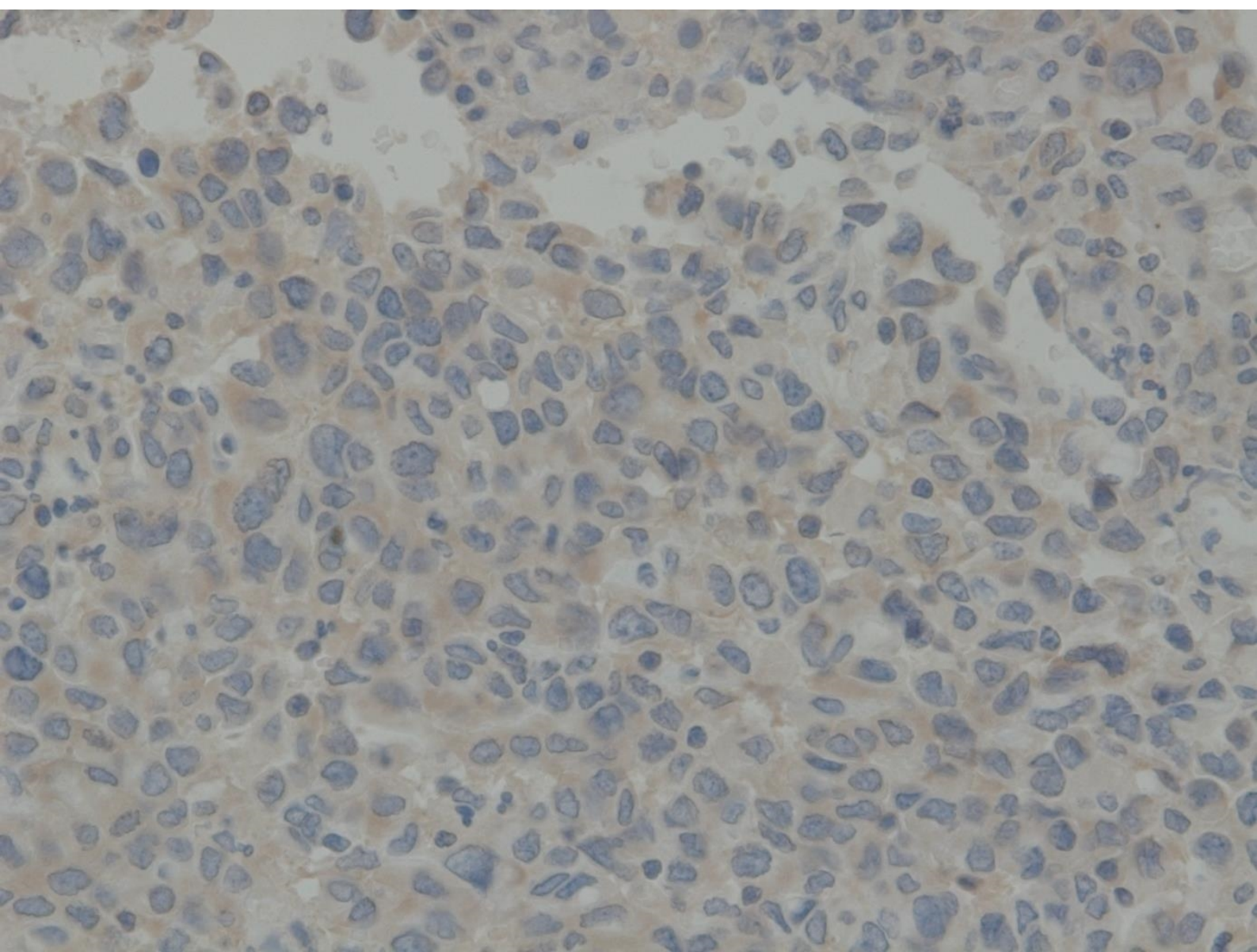

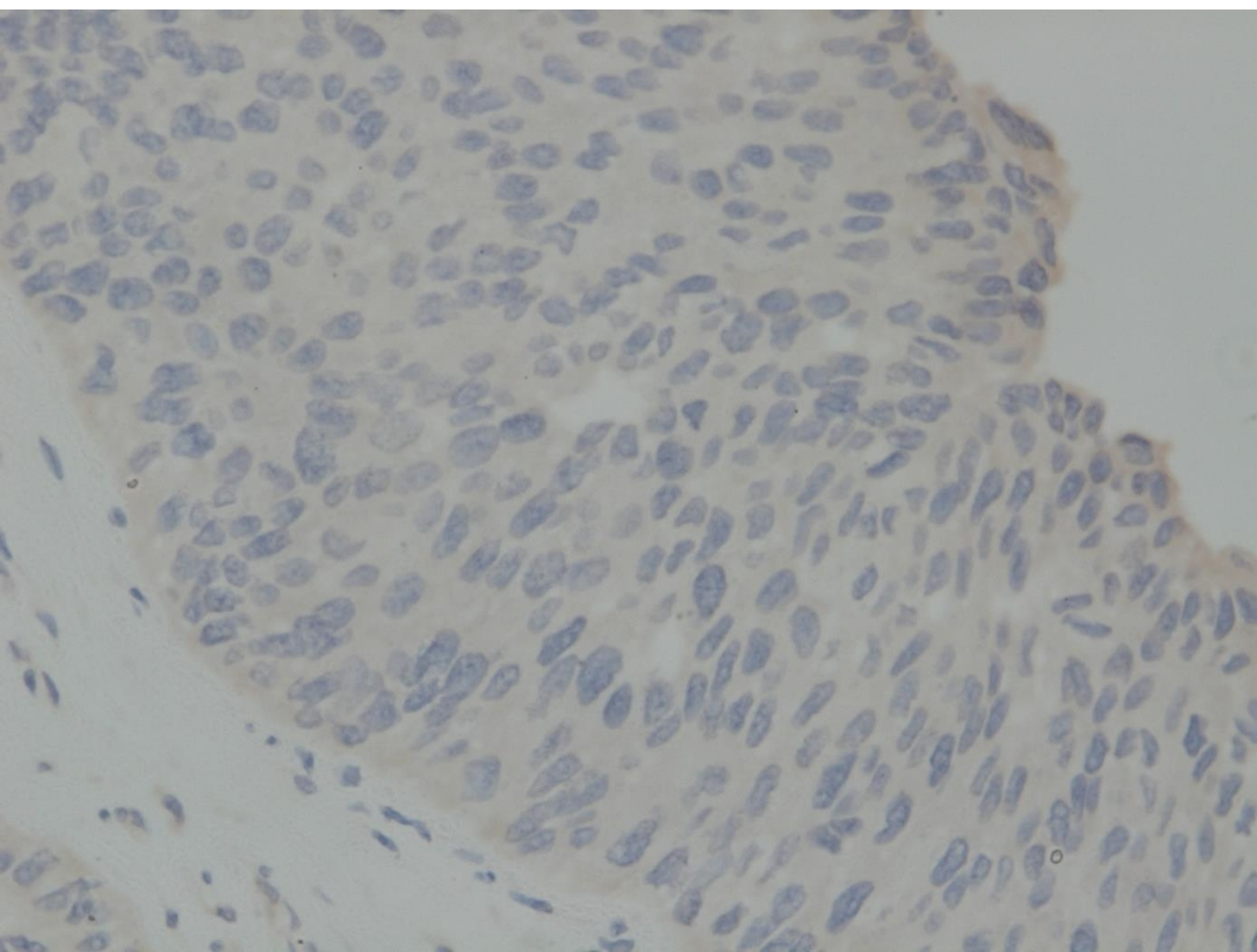

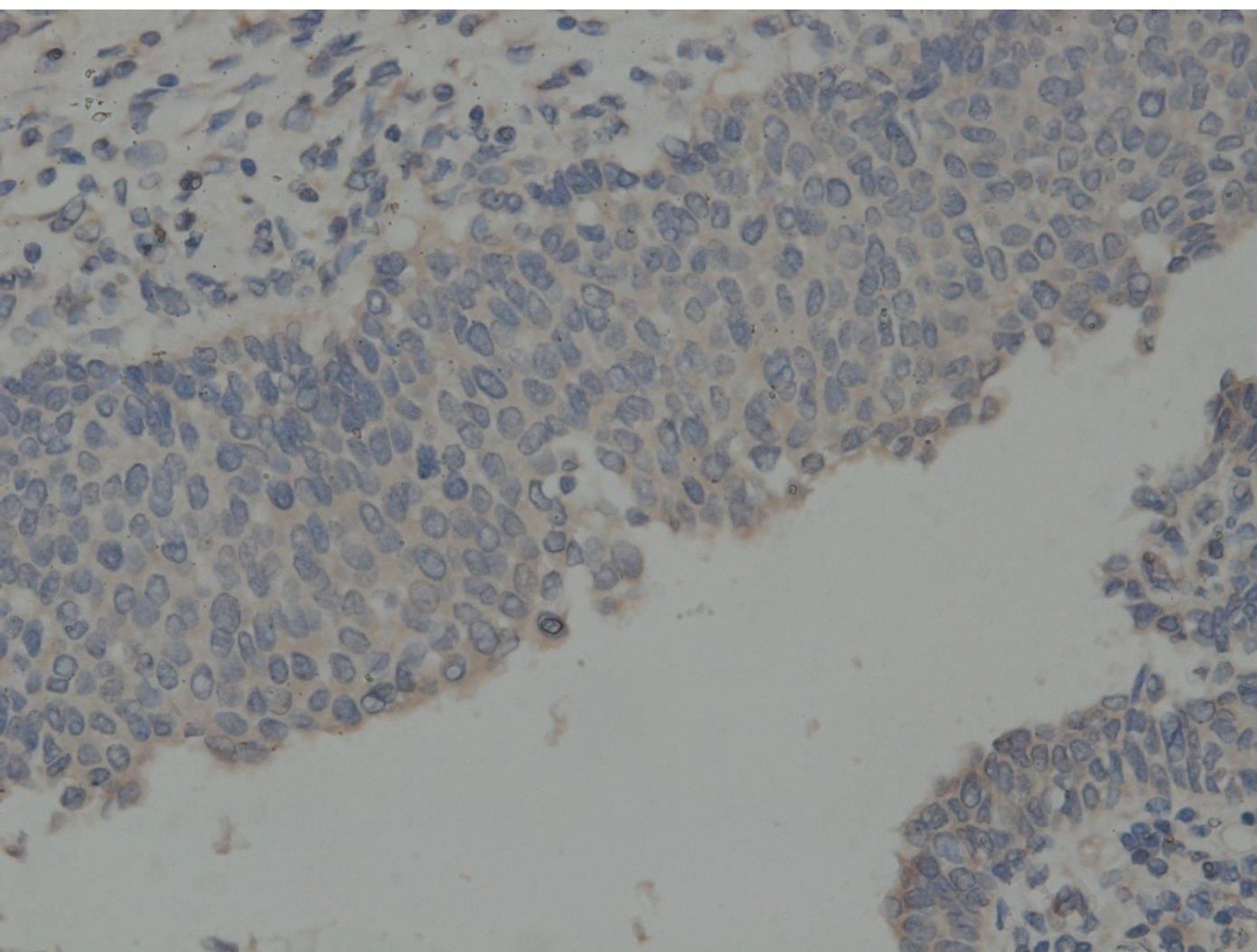

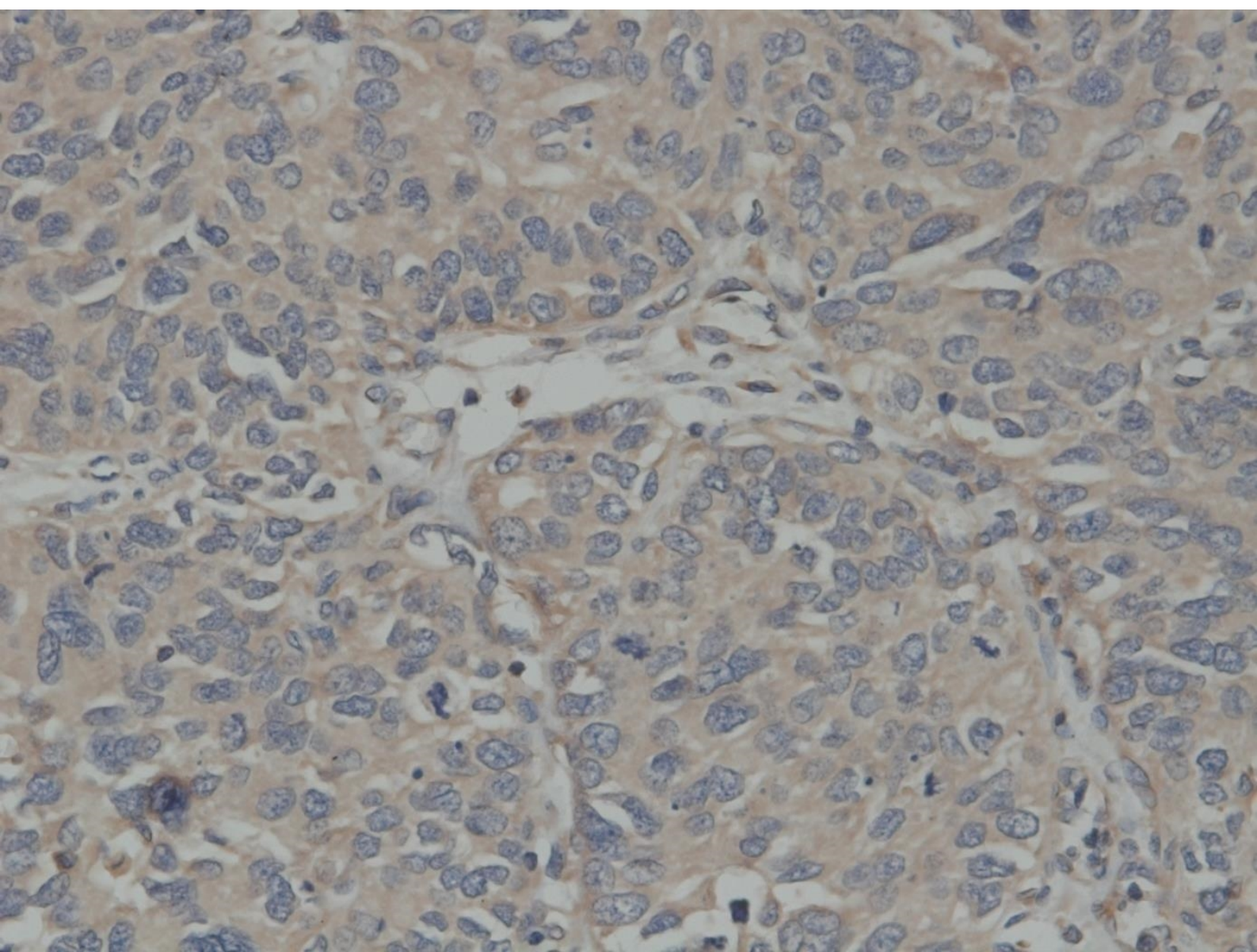

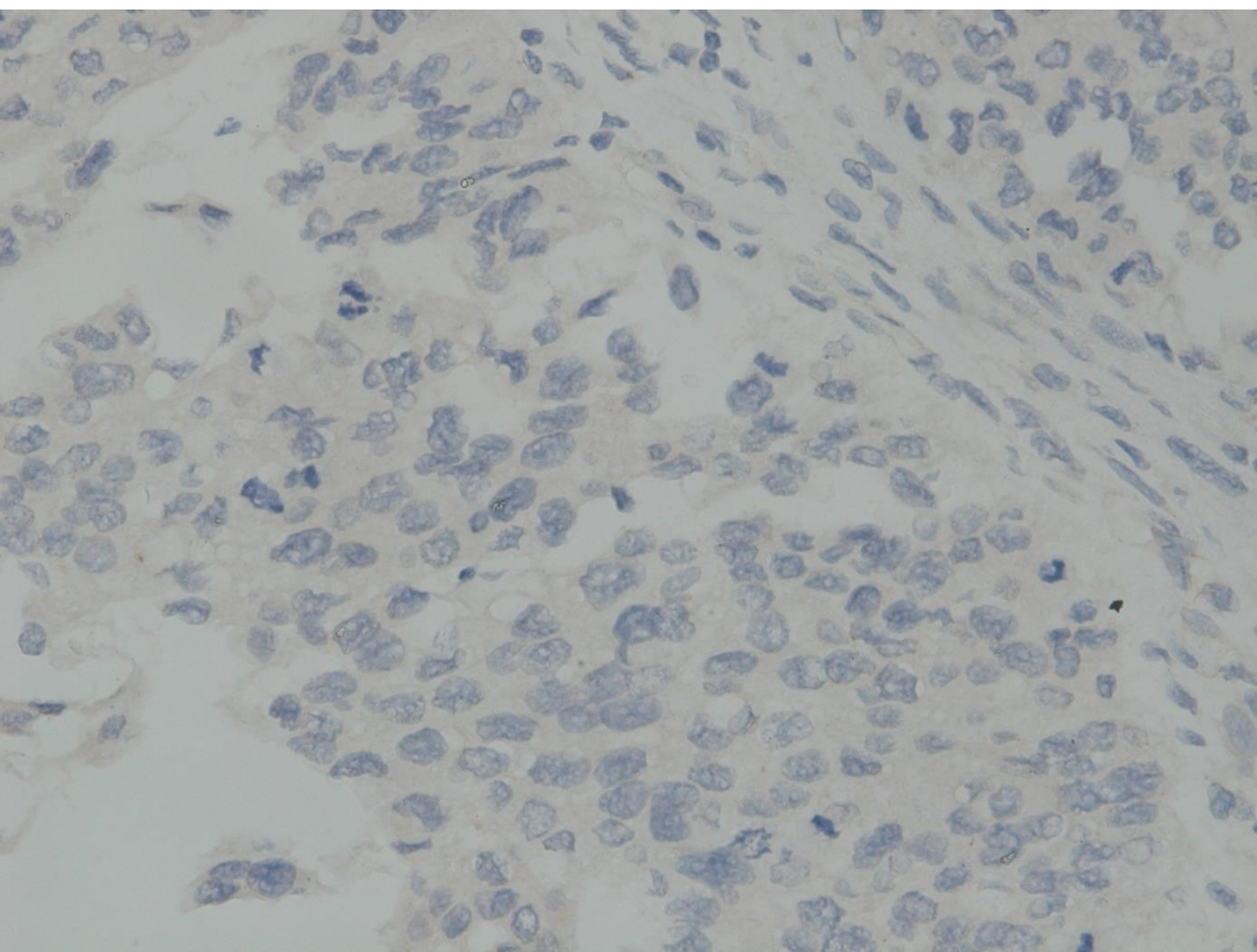

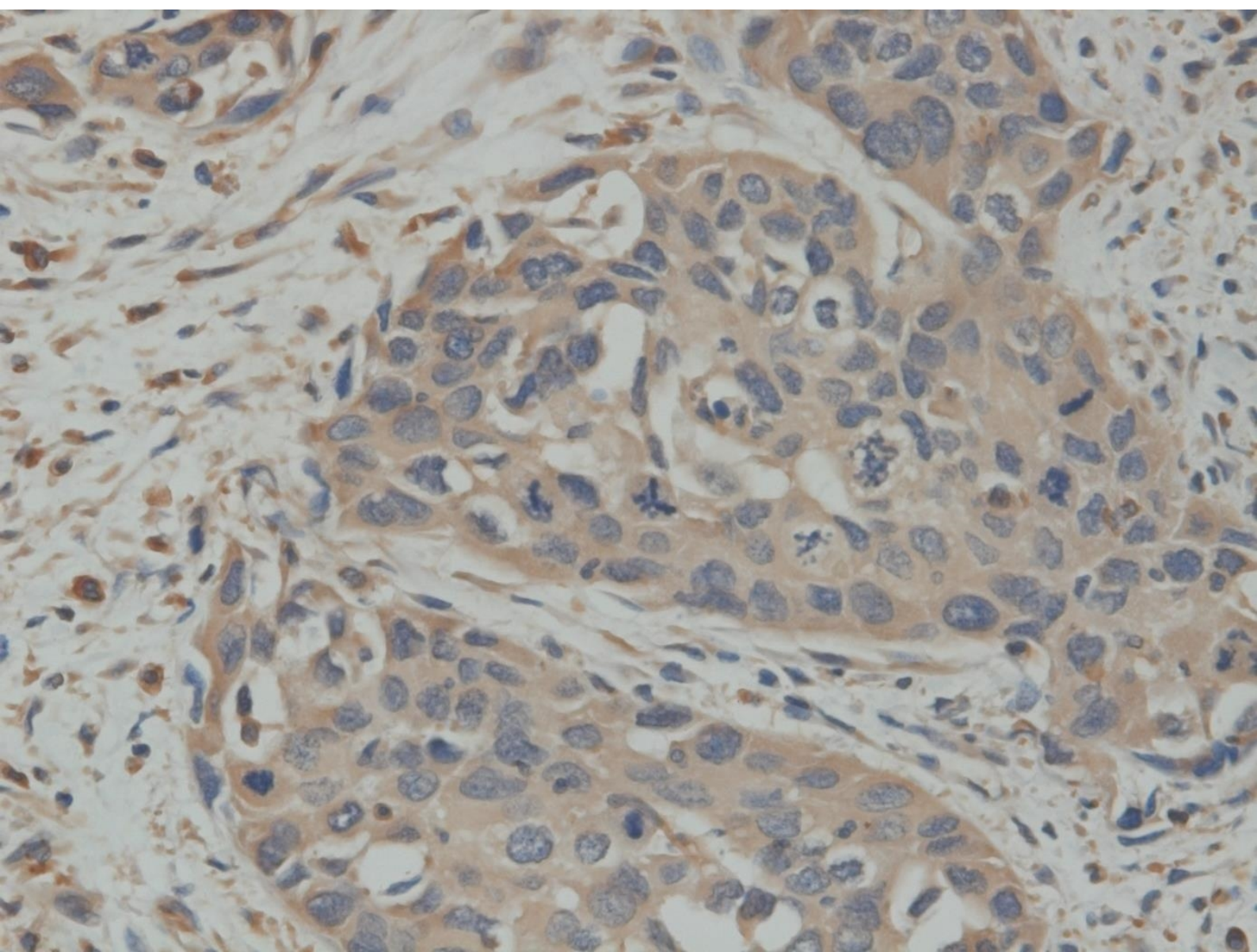

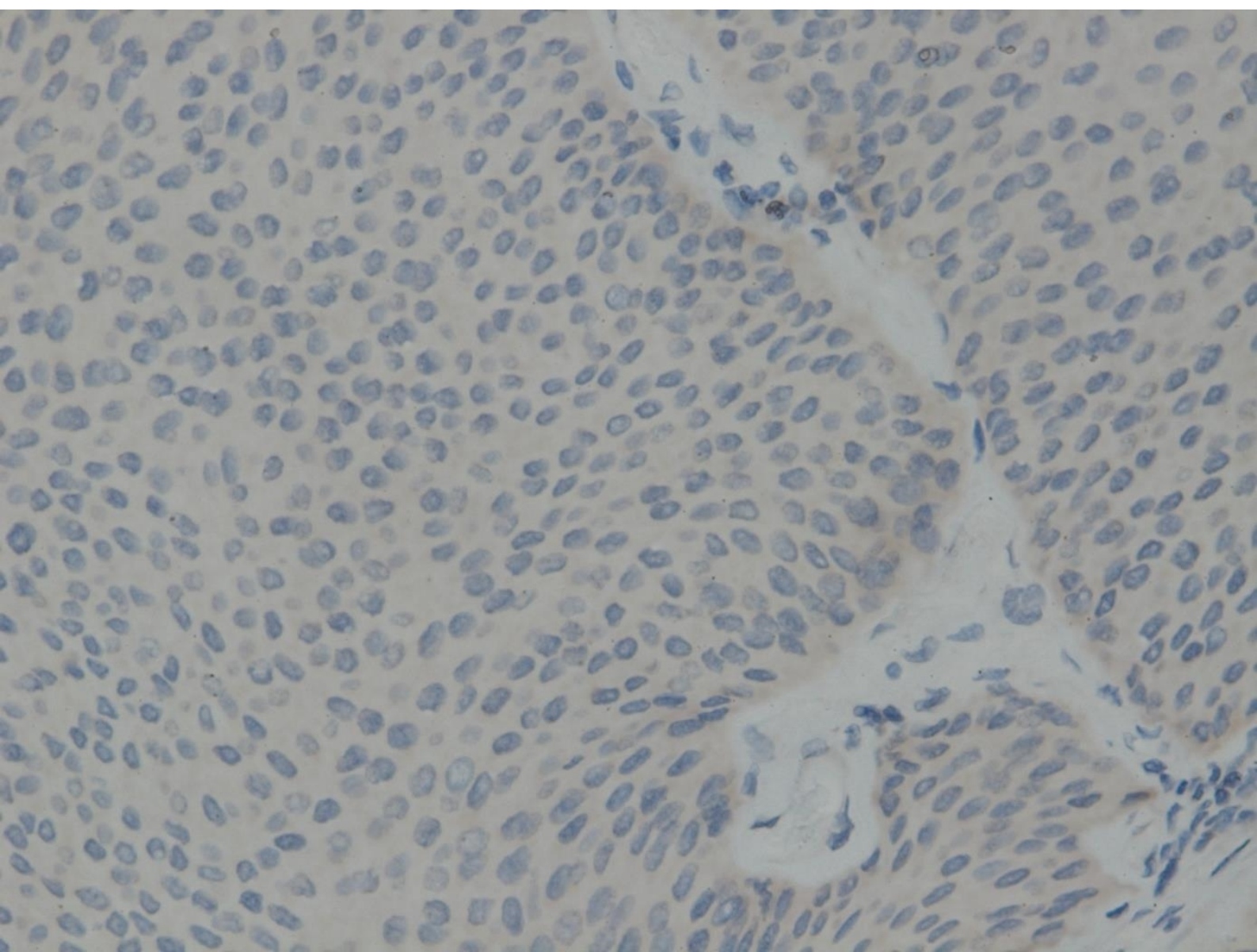

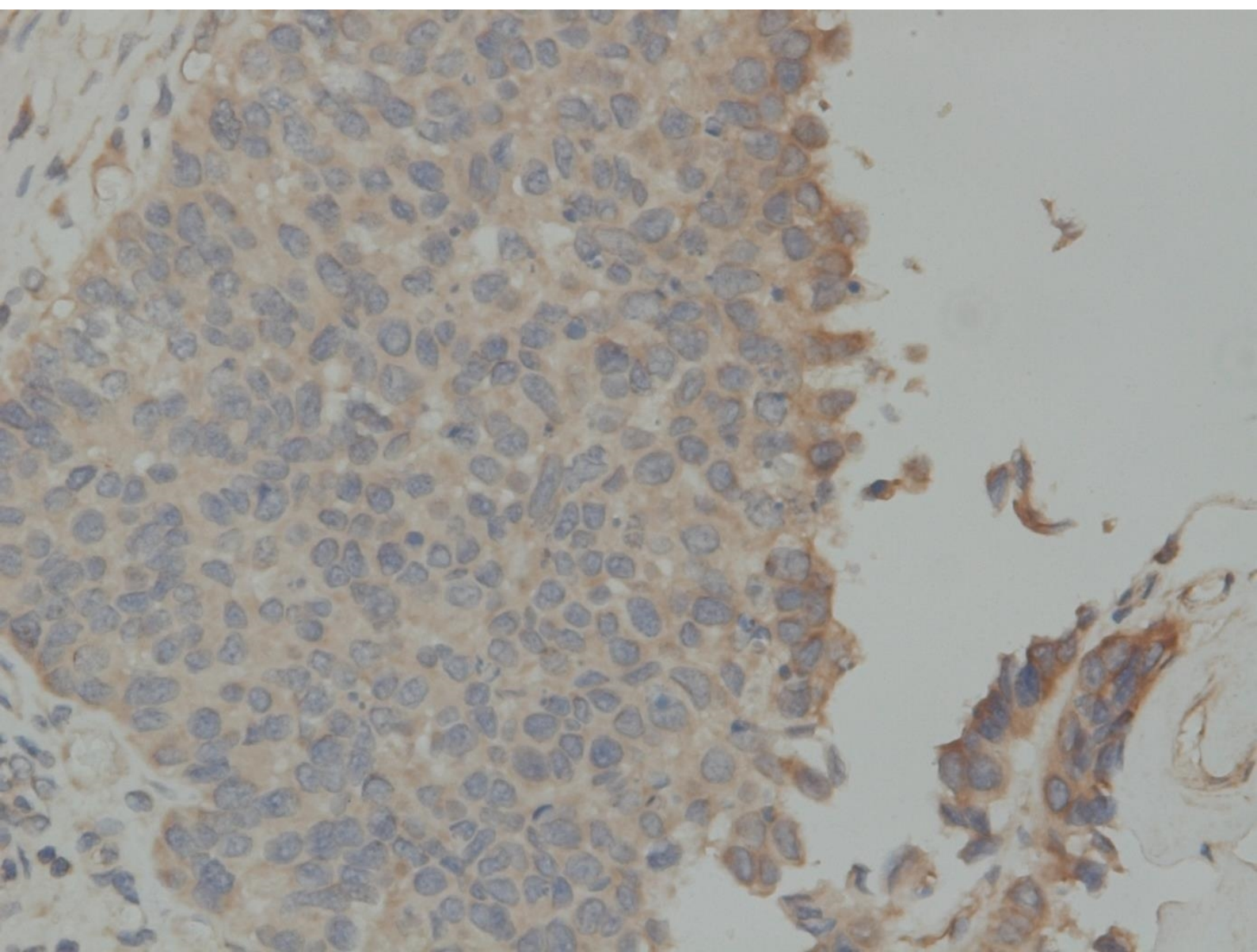

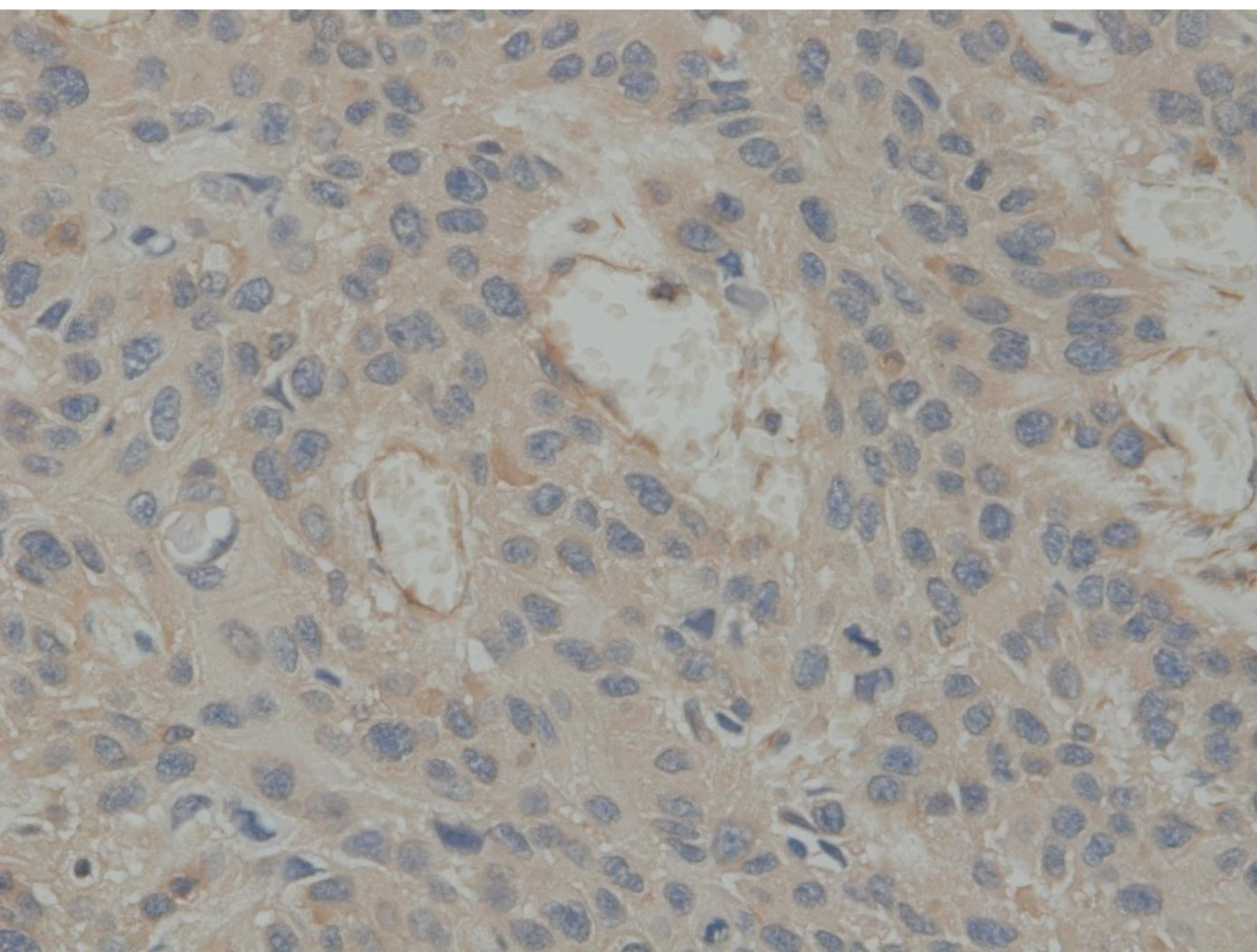

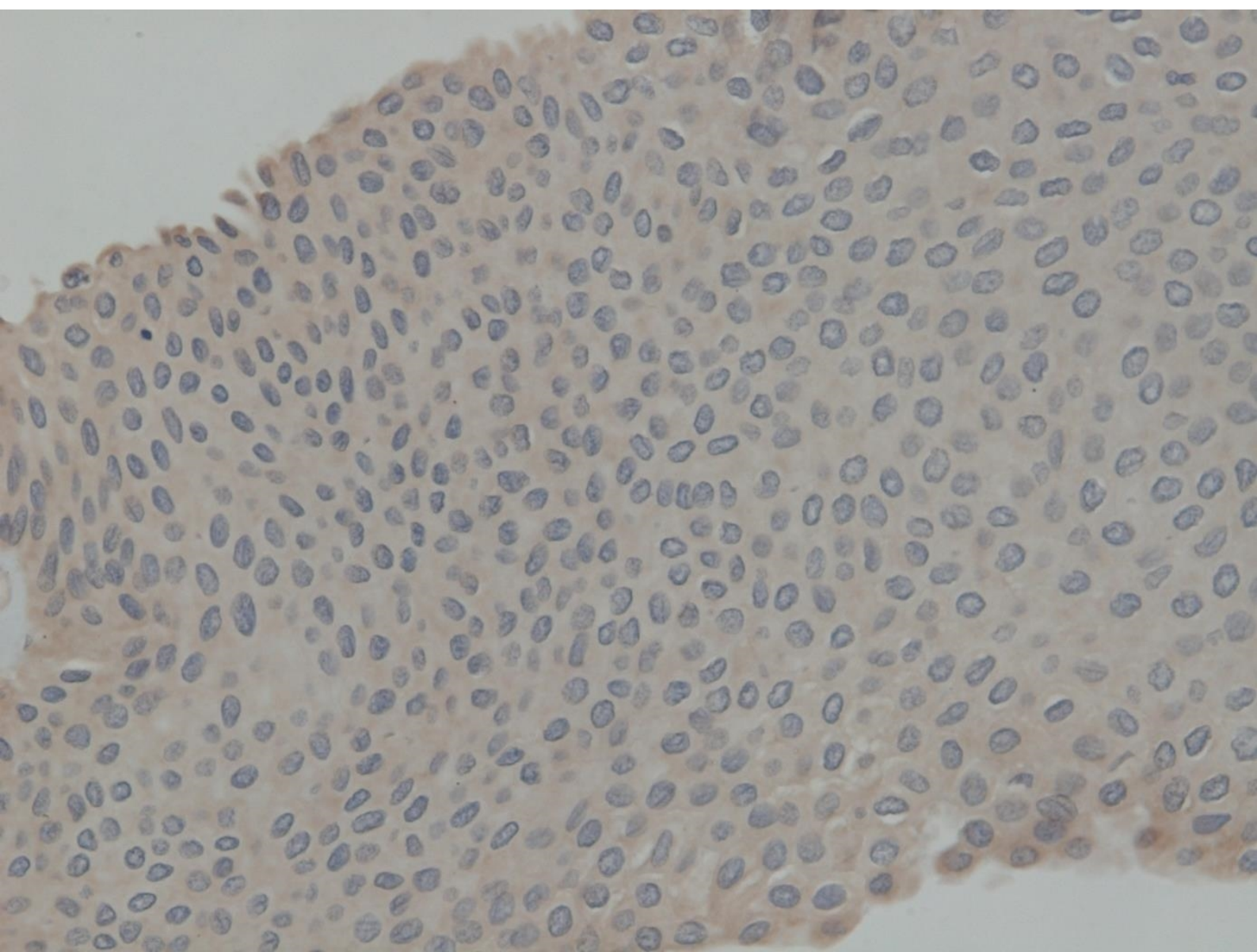

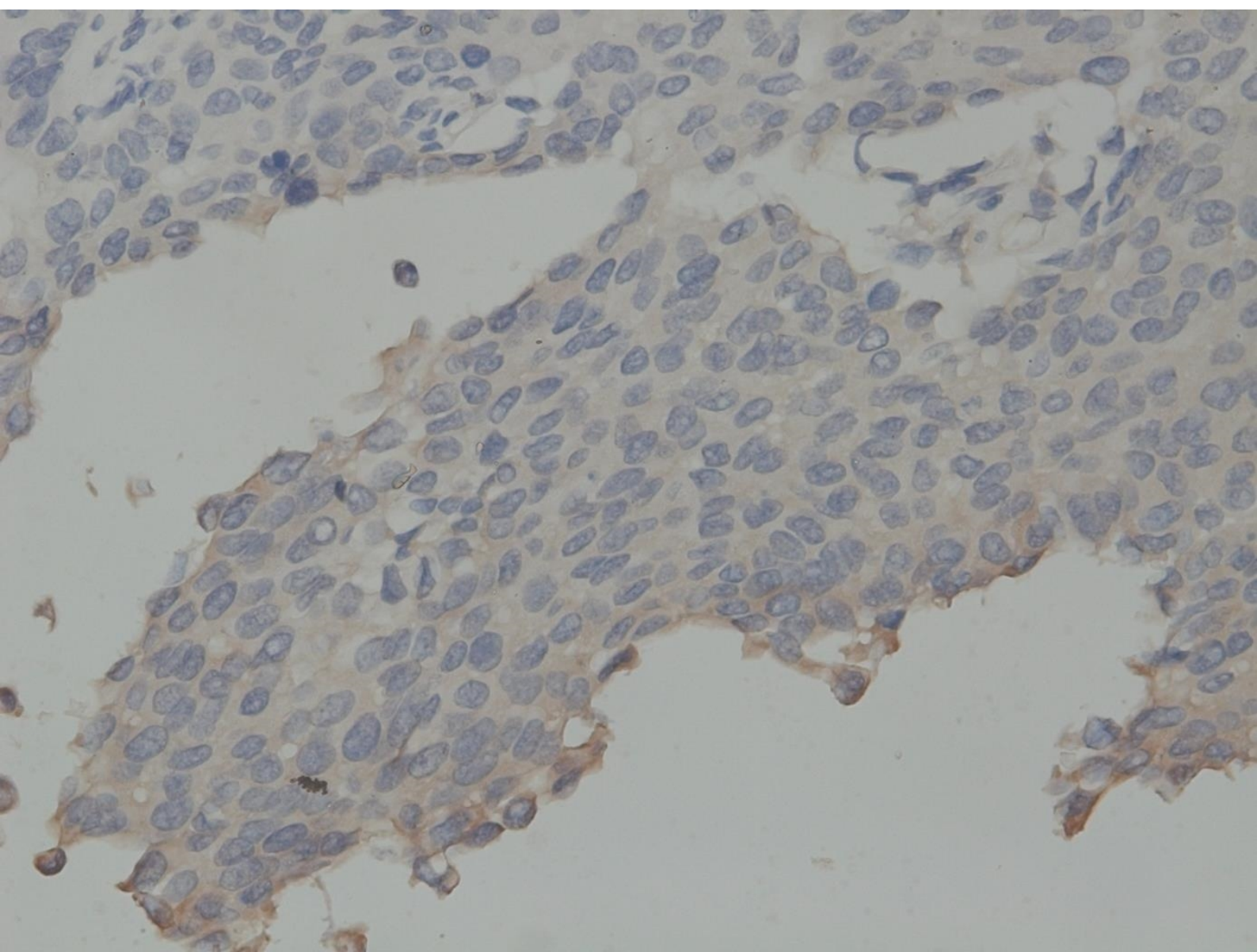

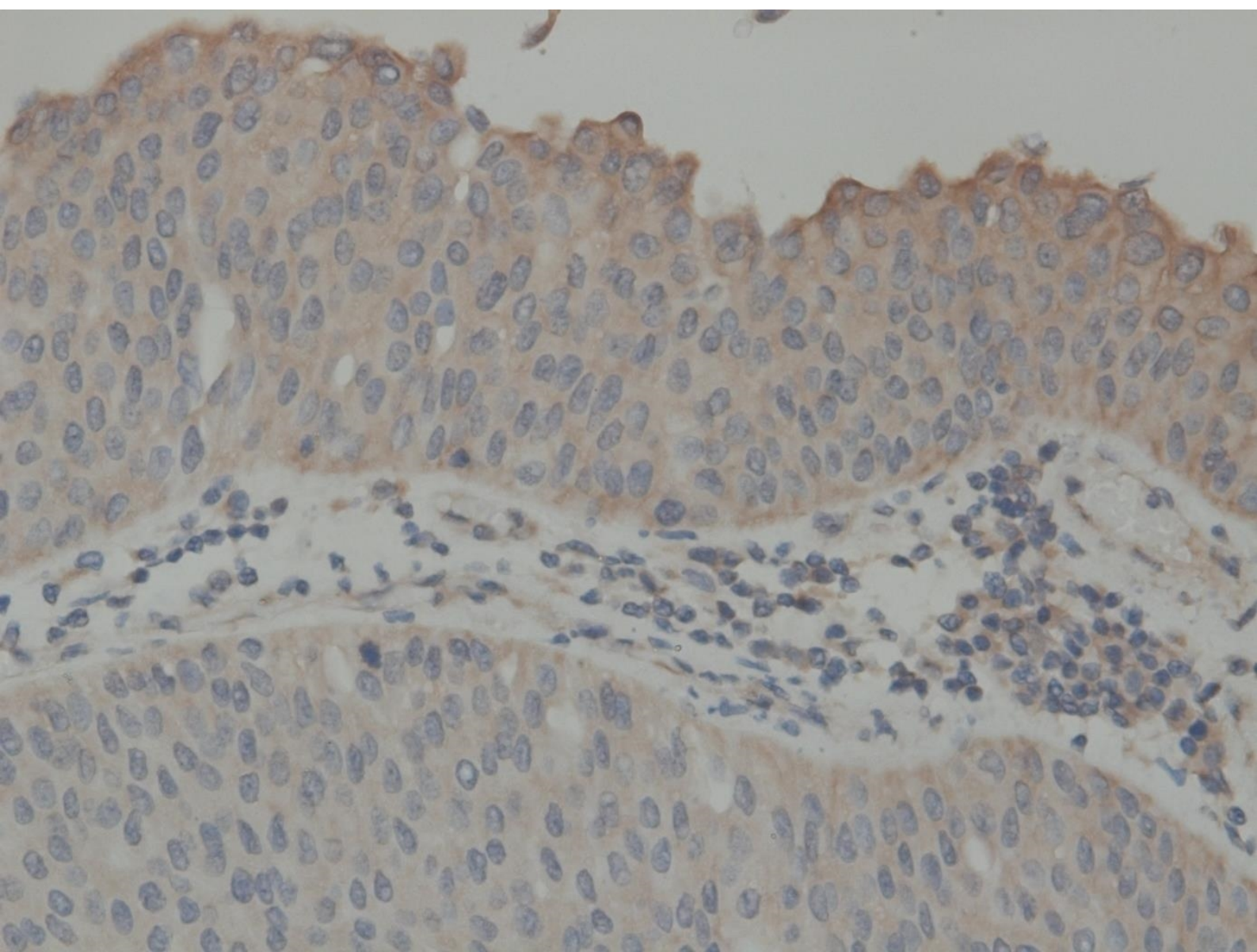

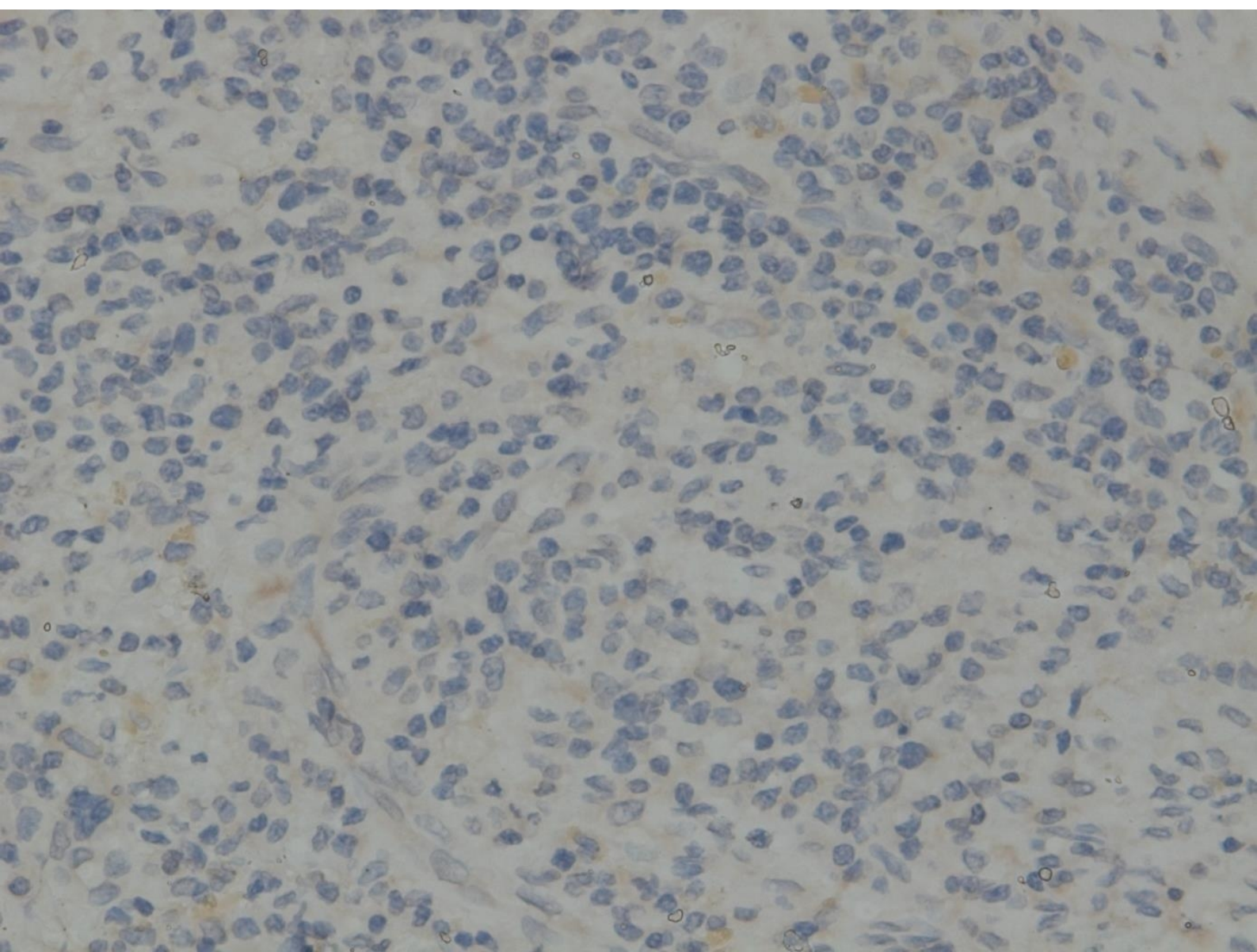

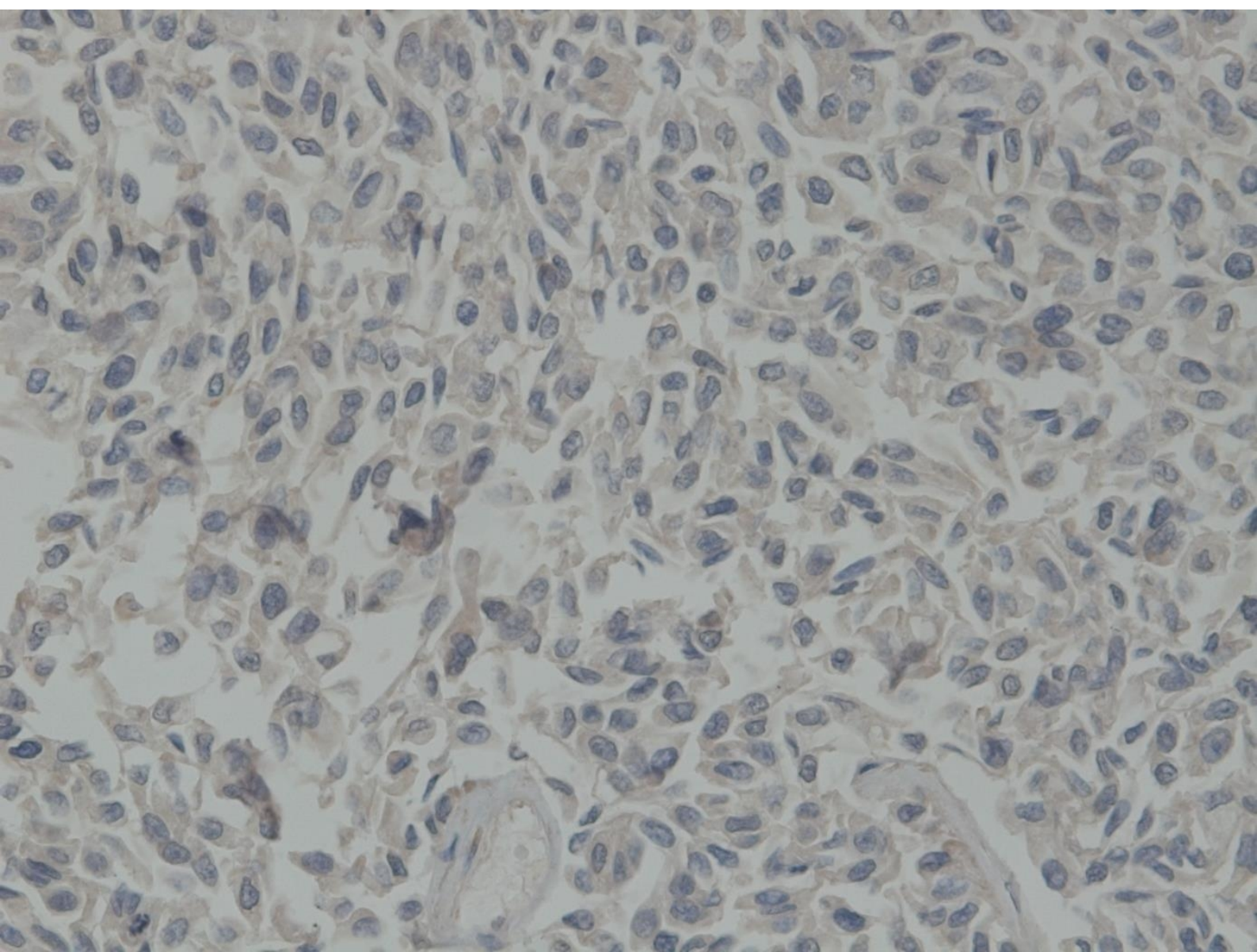

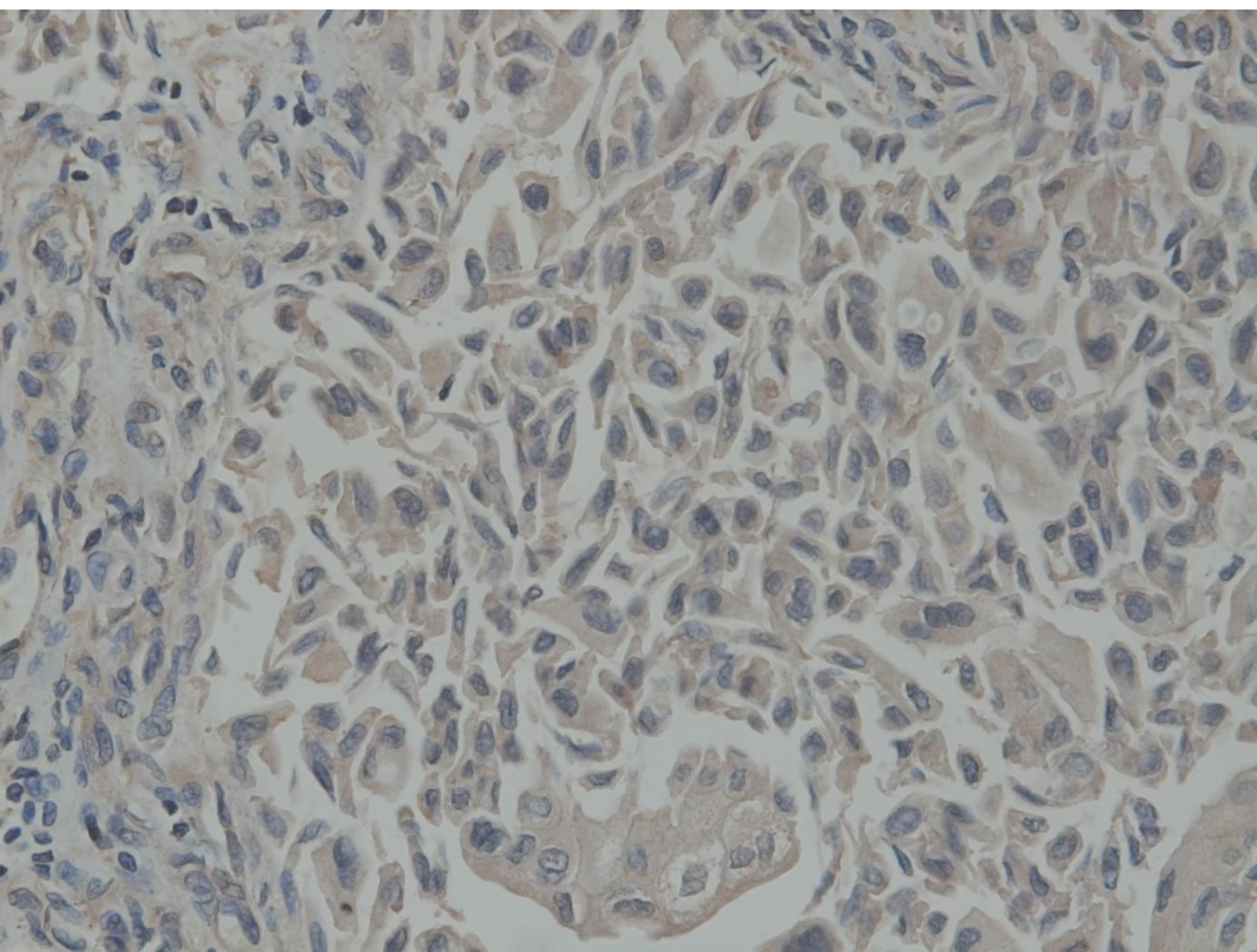

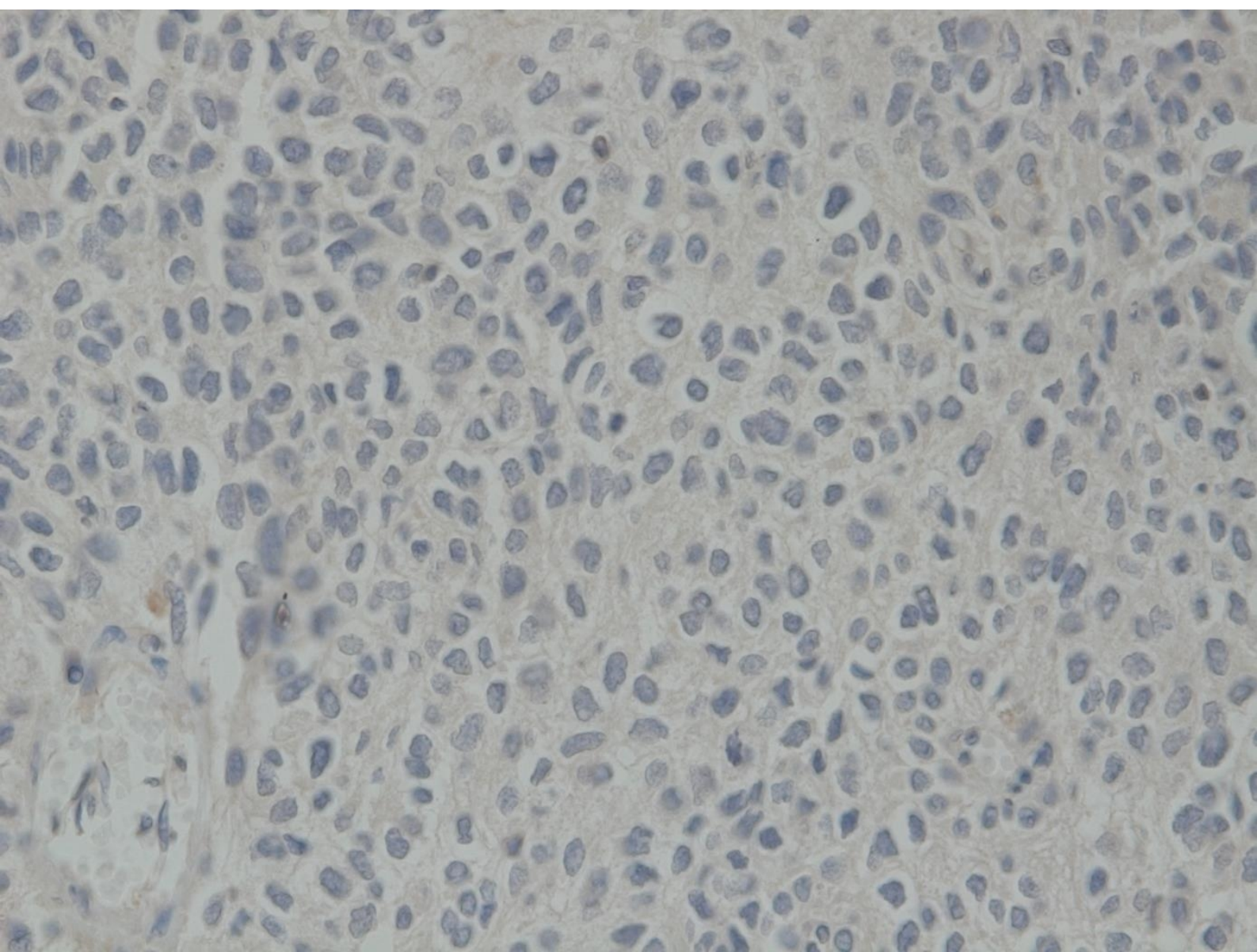

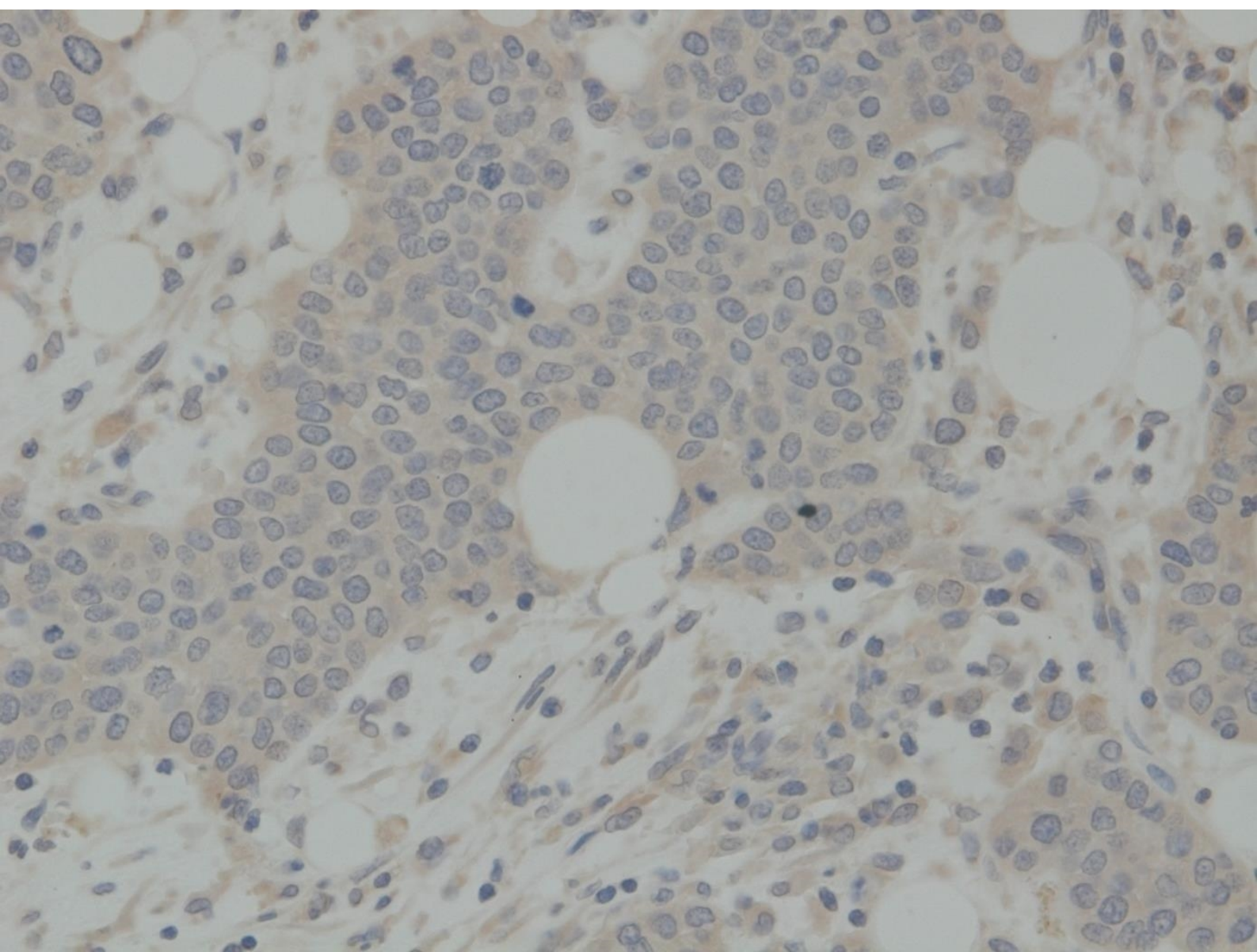

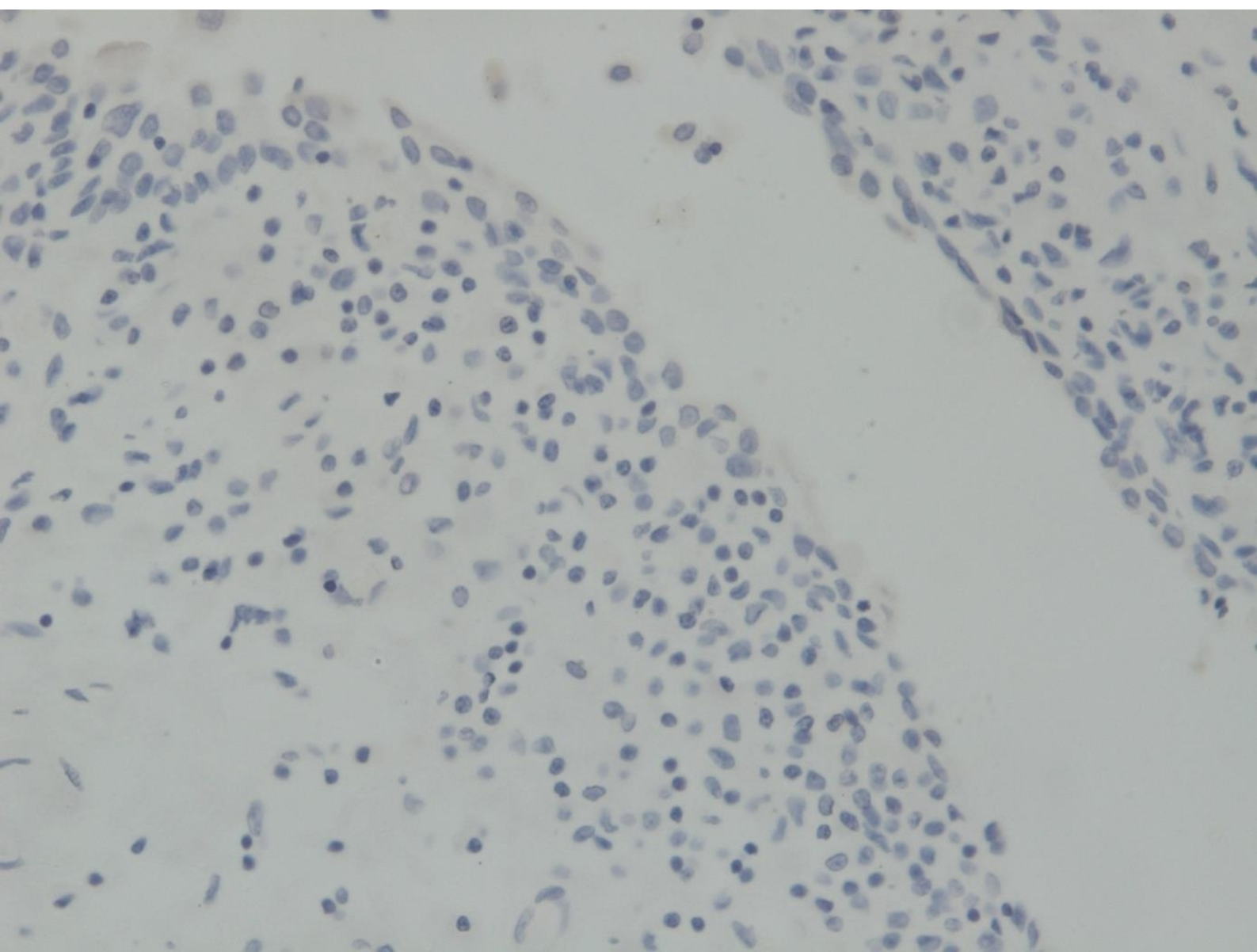

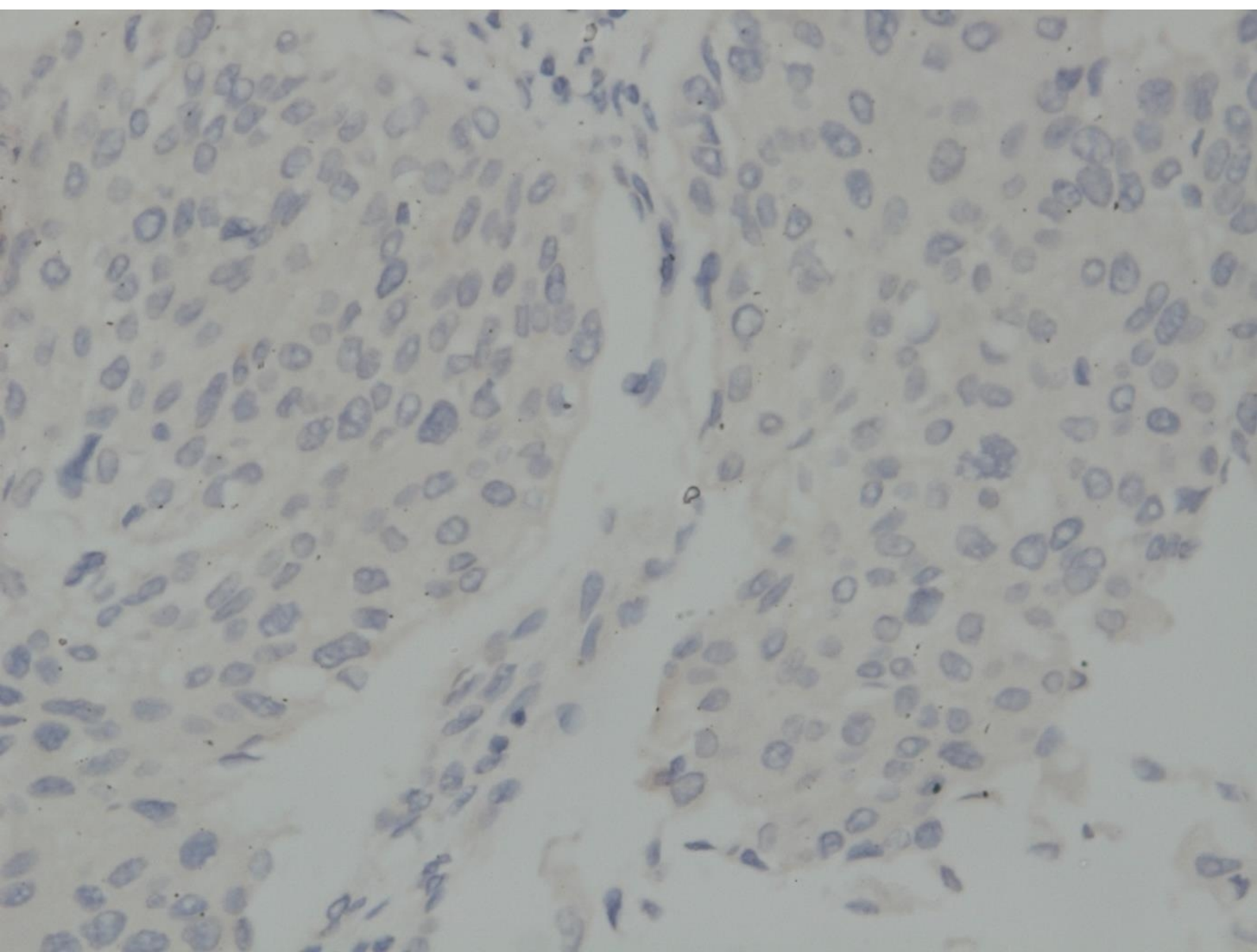

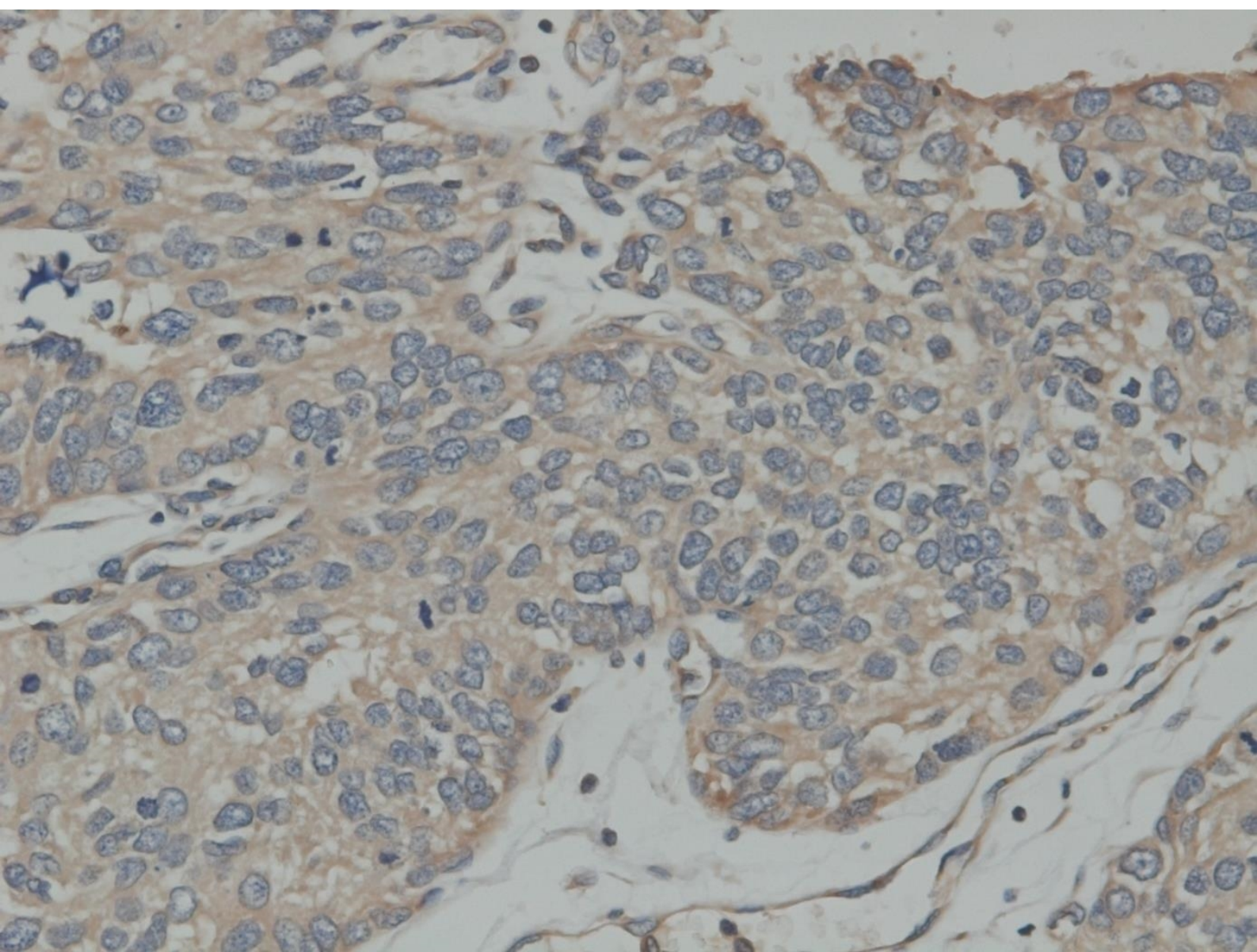

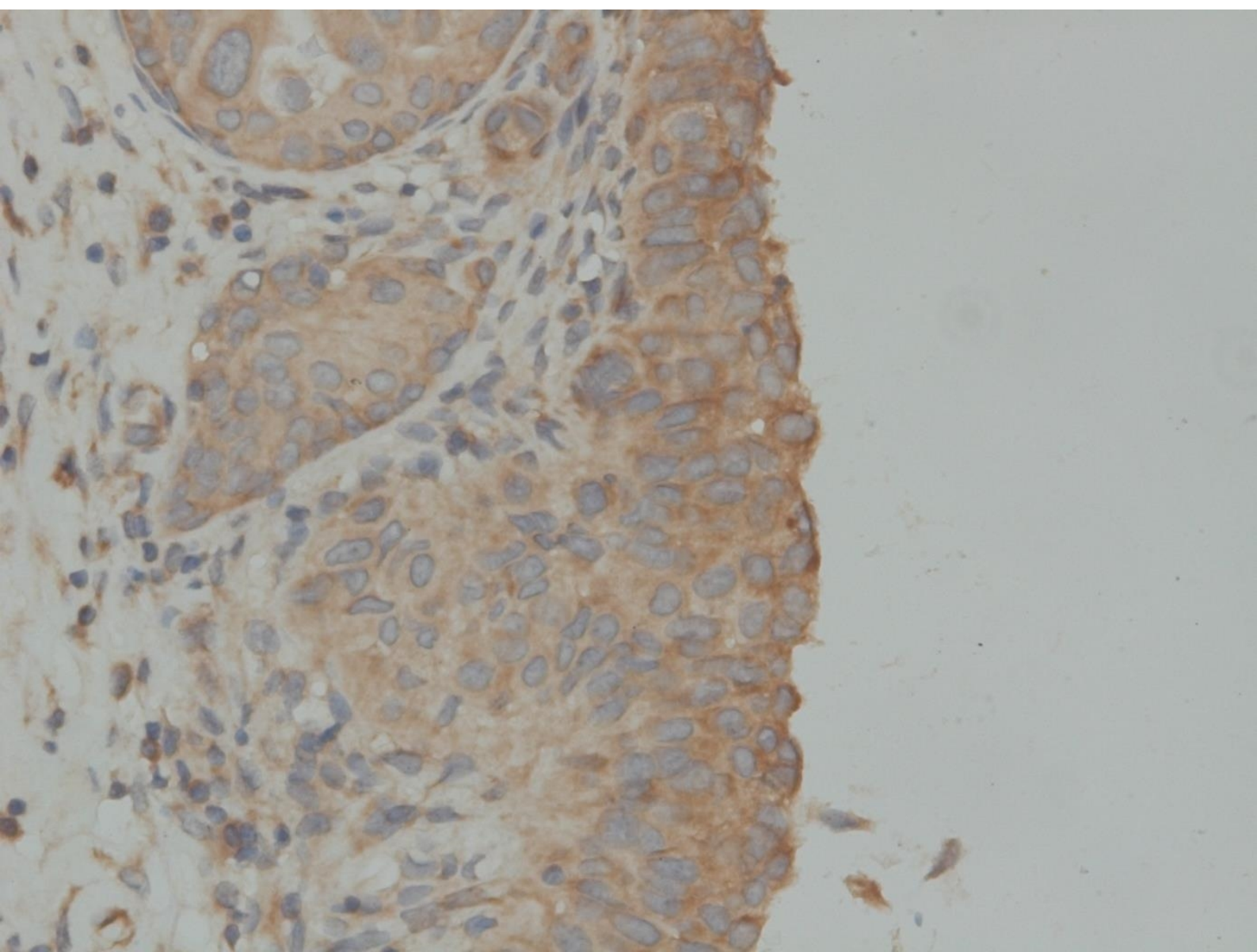

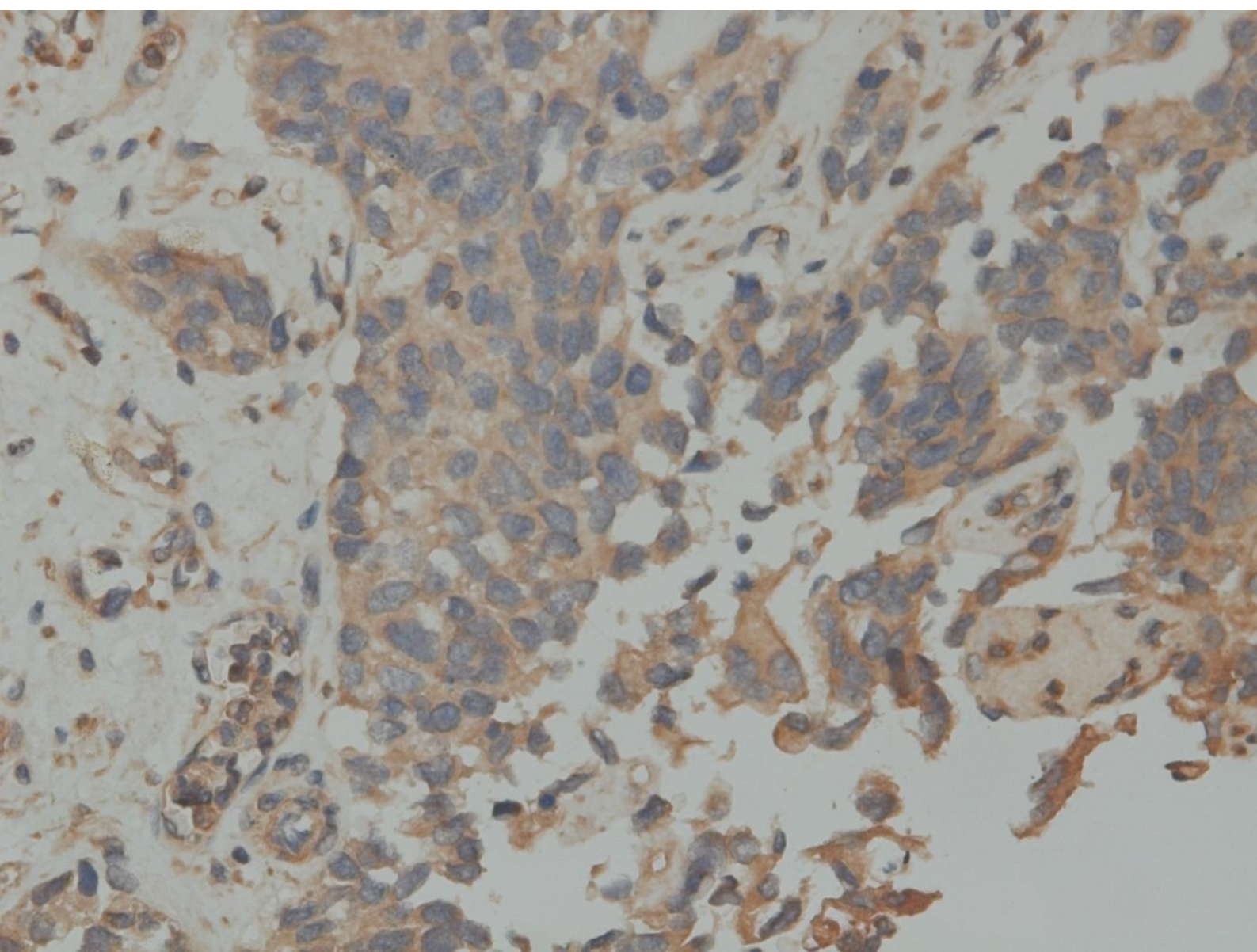

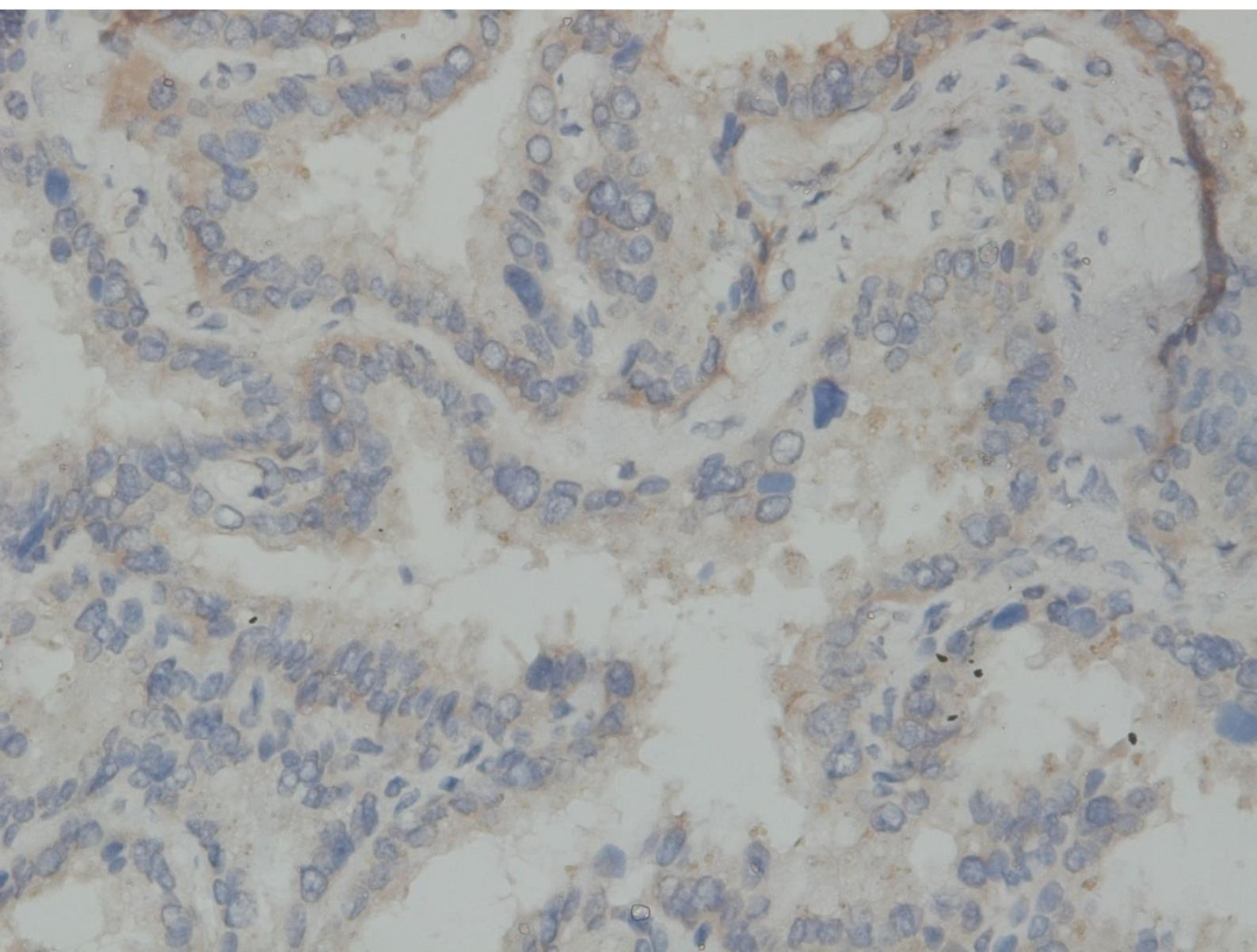

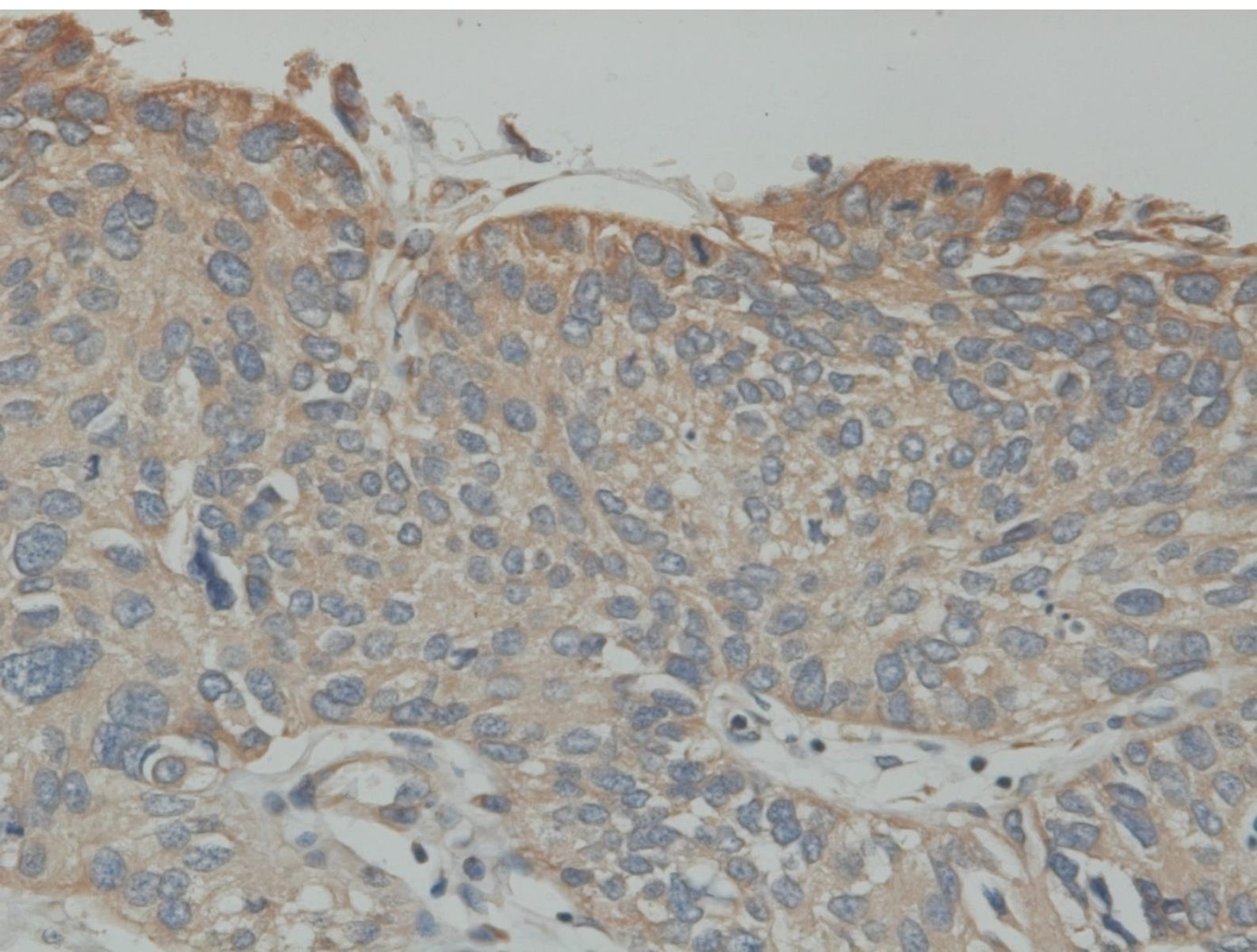

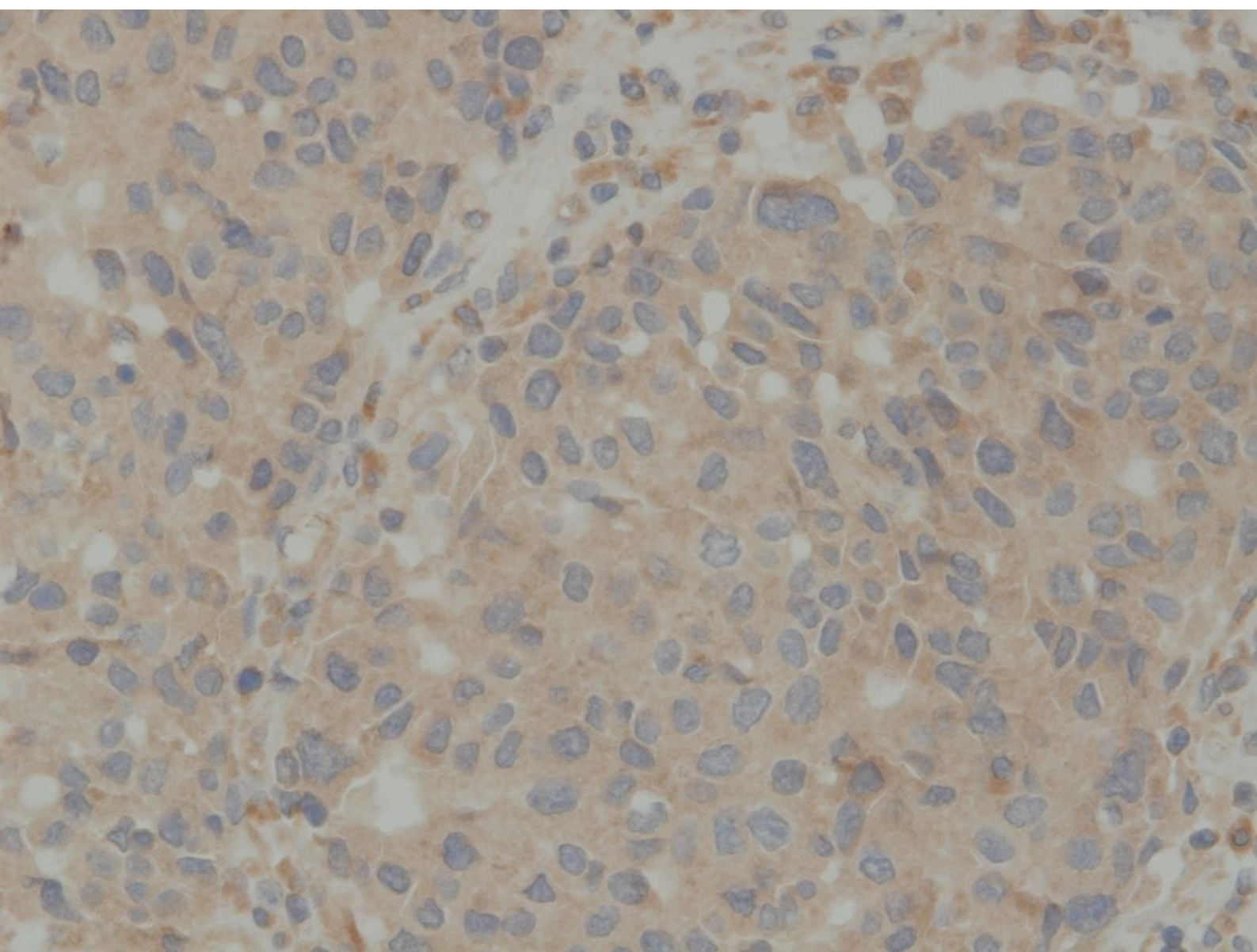

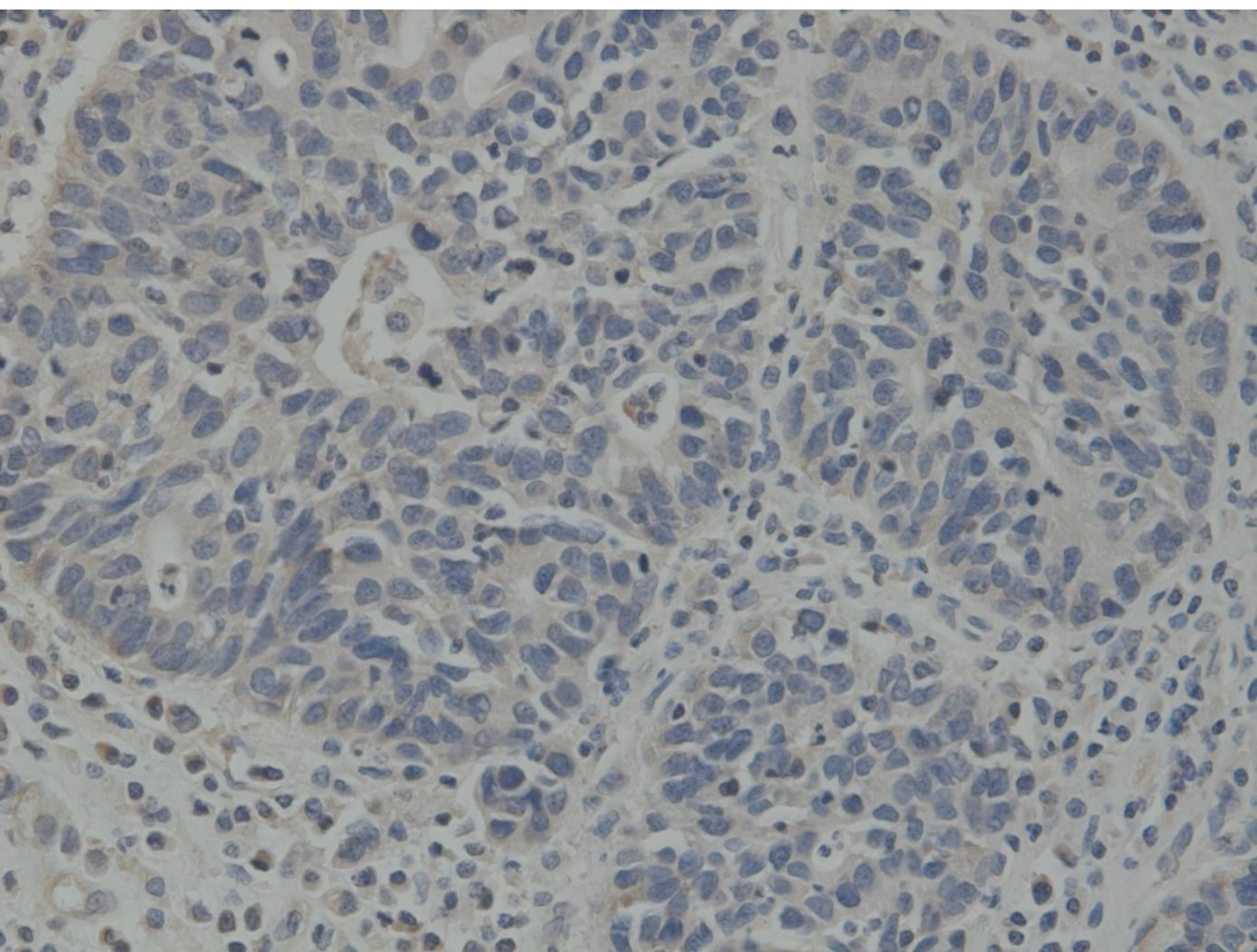

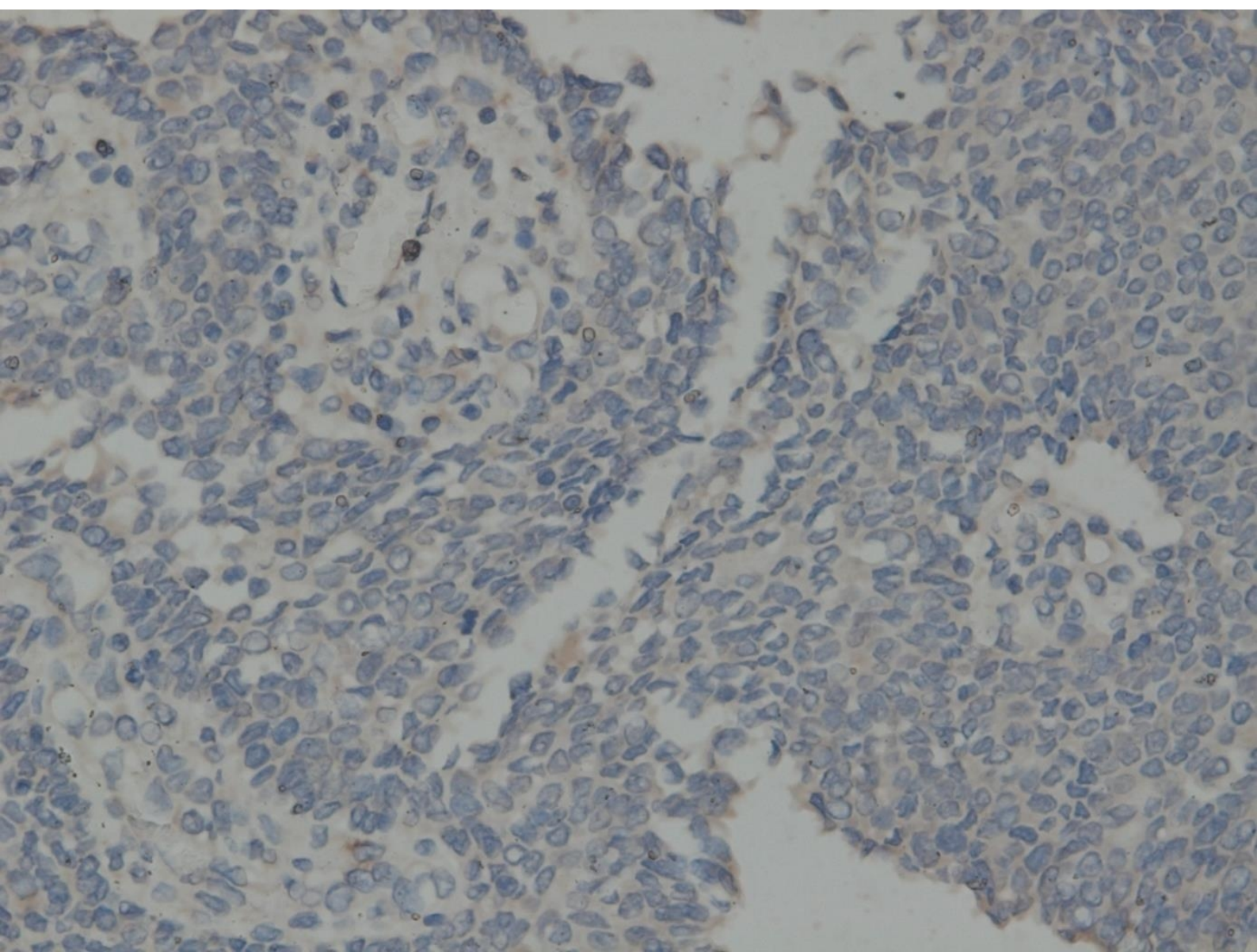

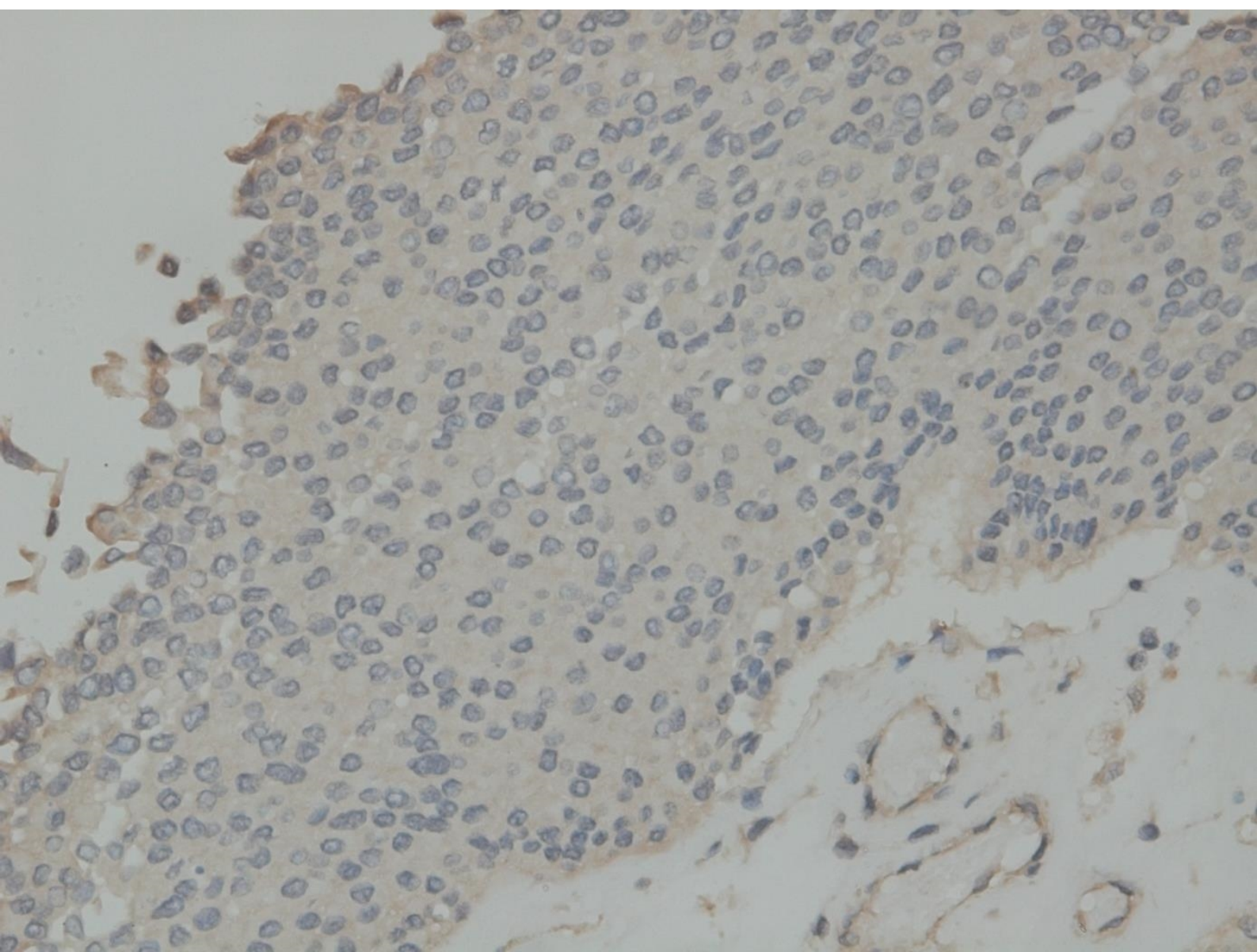

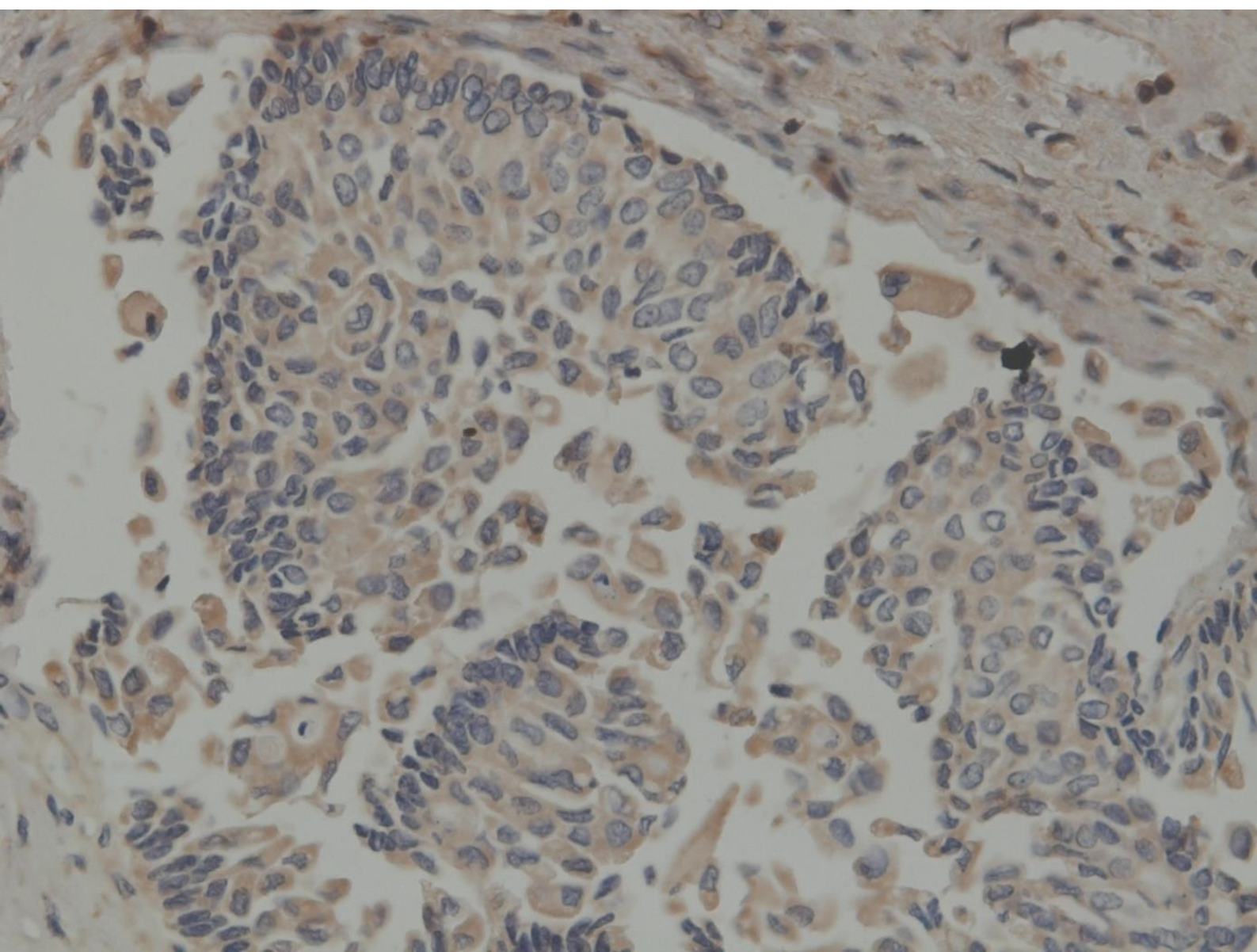

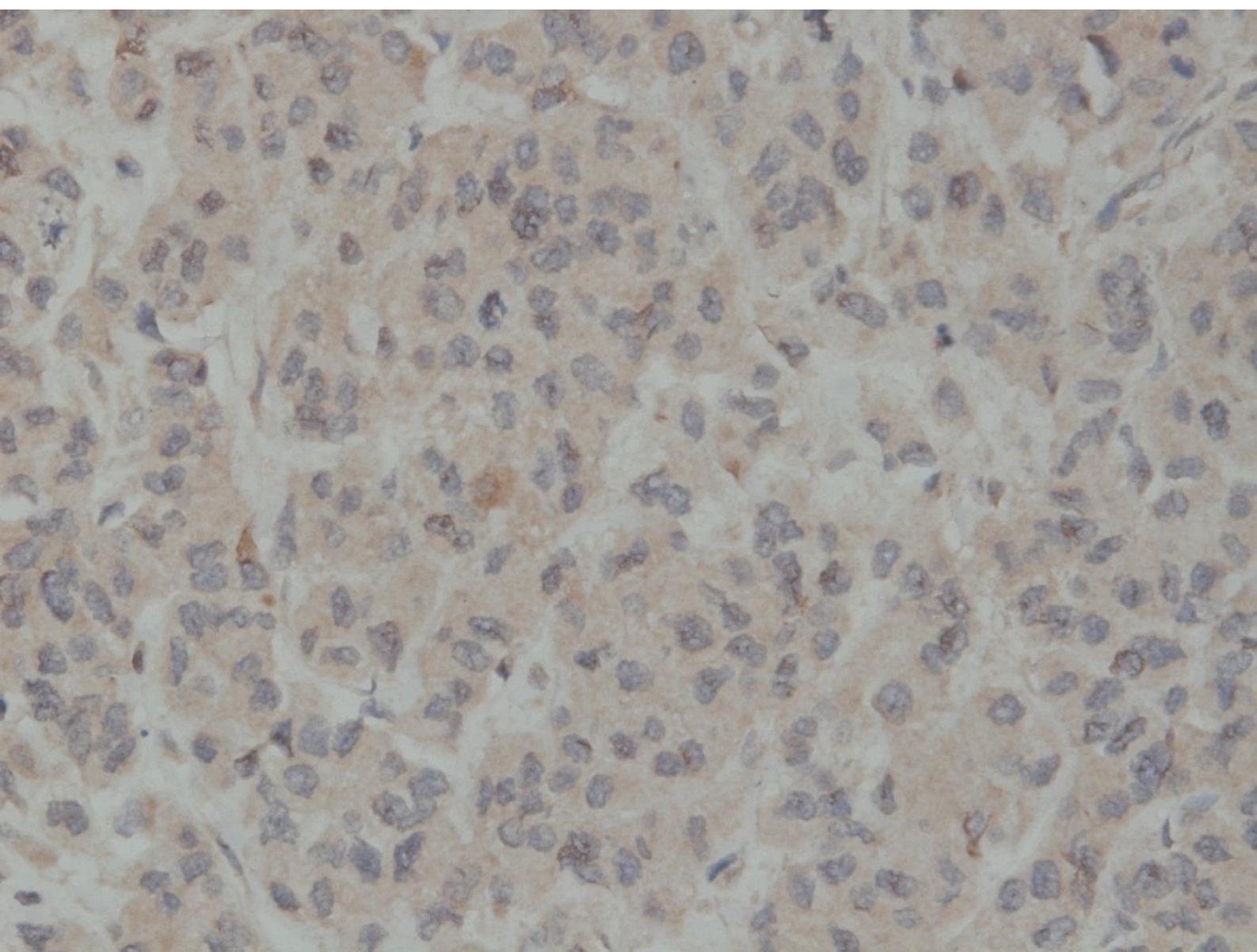

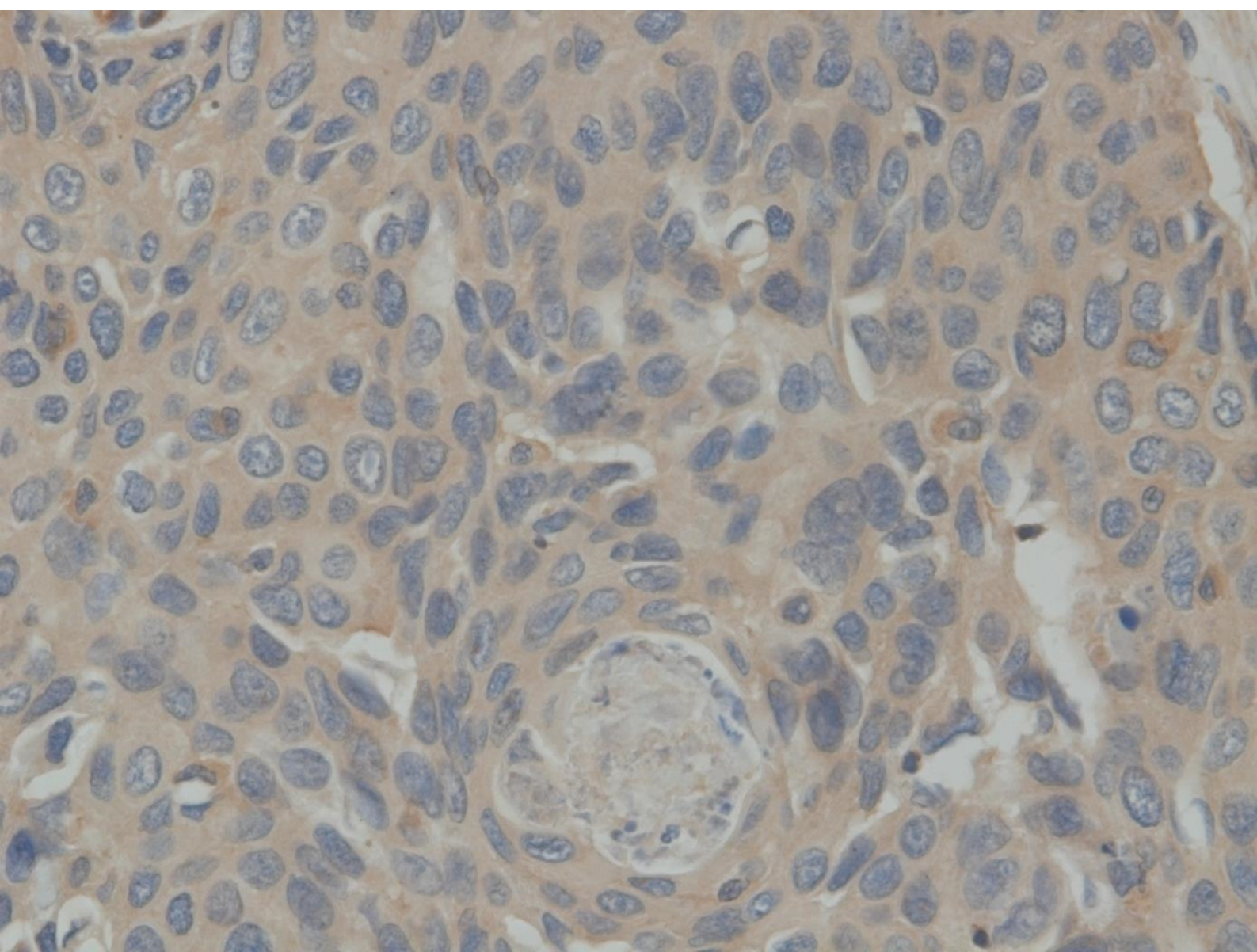

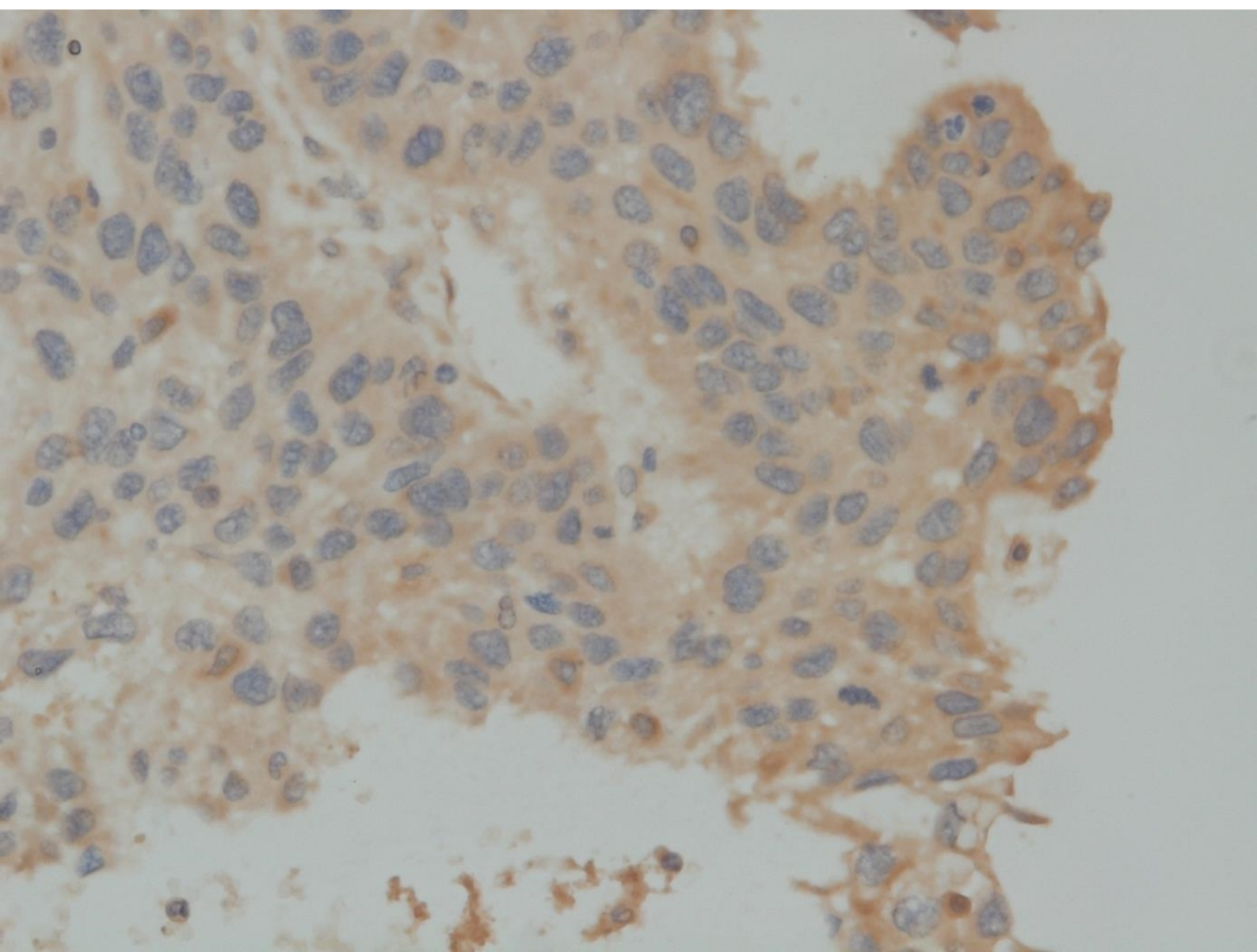

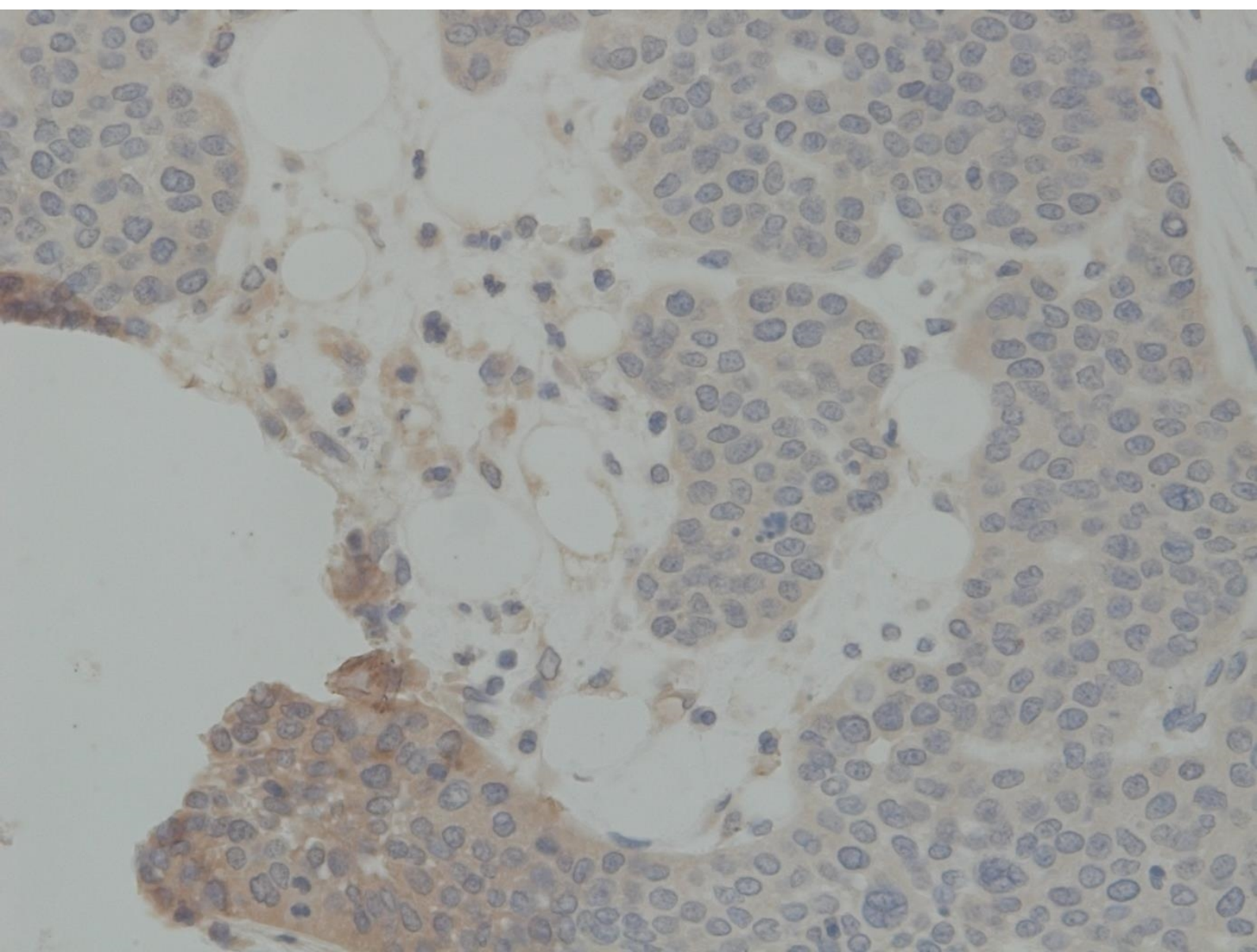

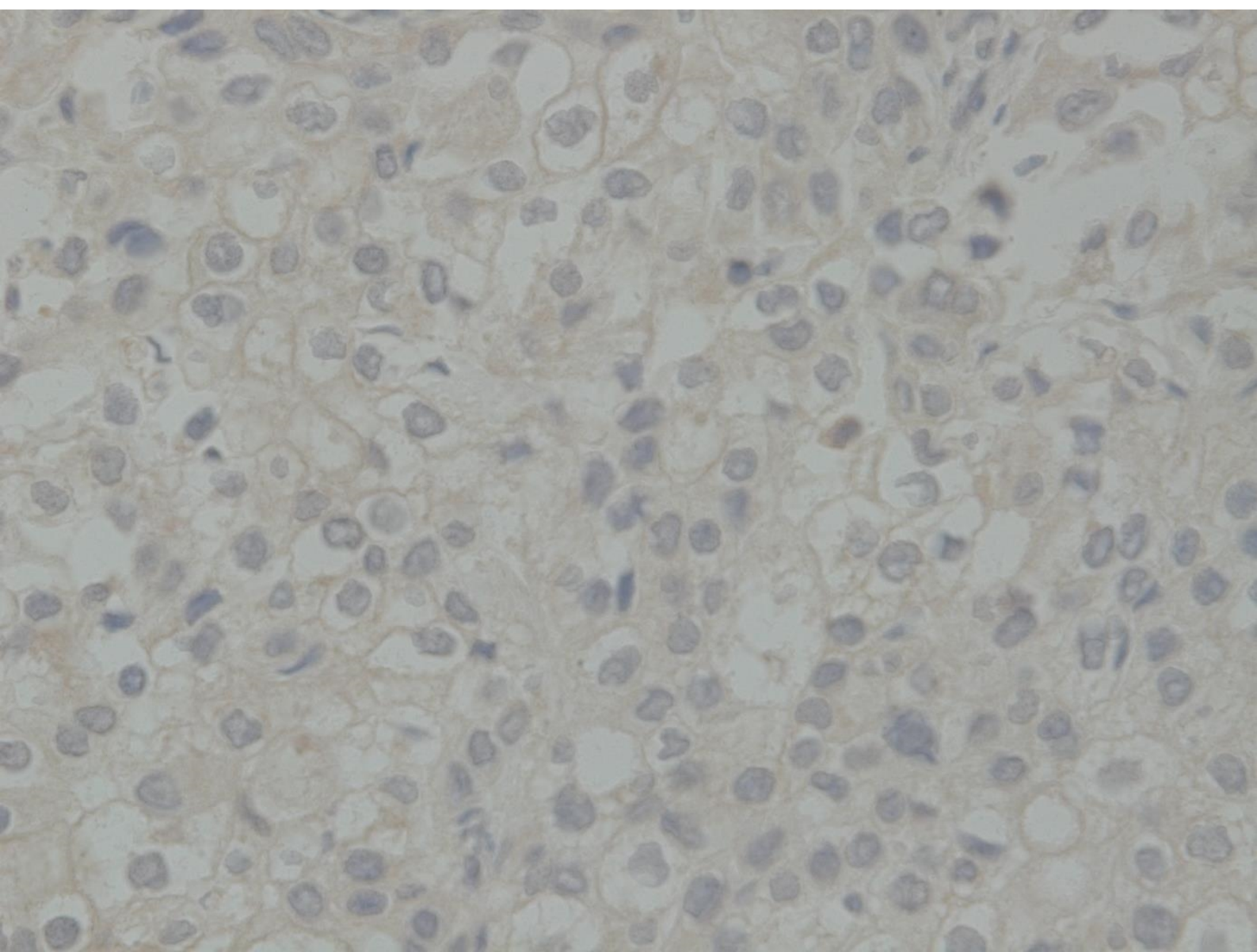

Supplement: Supplementary file 5 — Additional file 5. IHC 1: cancer tissues Immunohistochemical result of patients [file 12894_2023_1211_MOESM5_ESM.pdf]

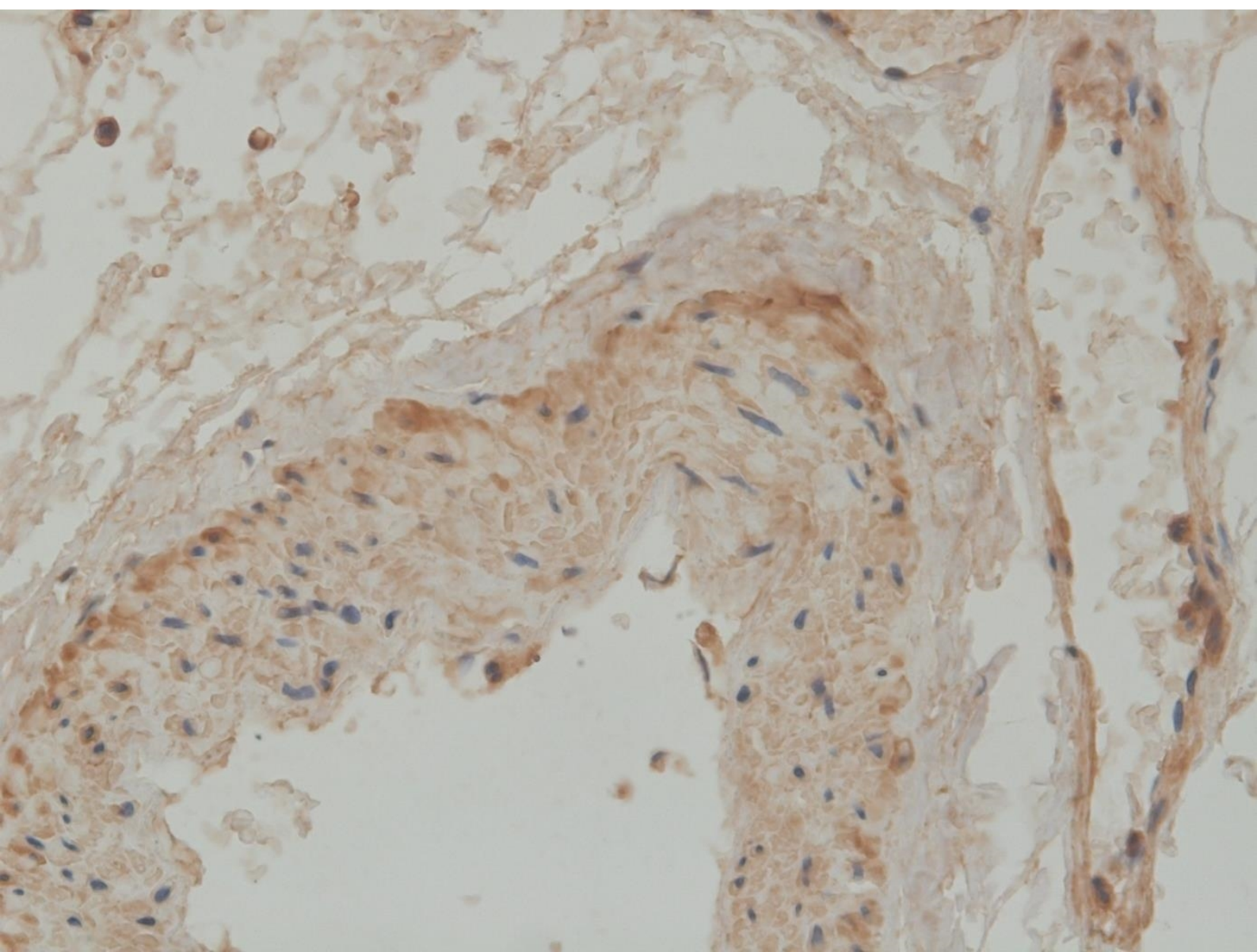

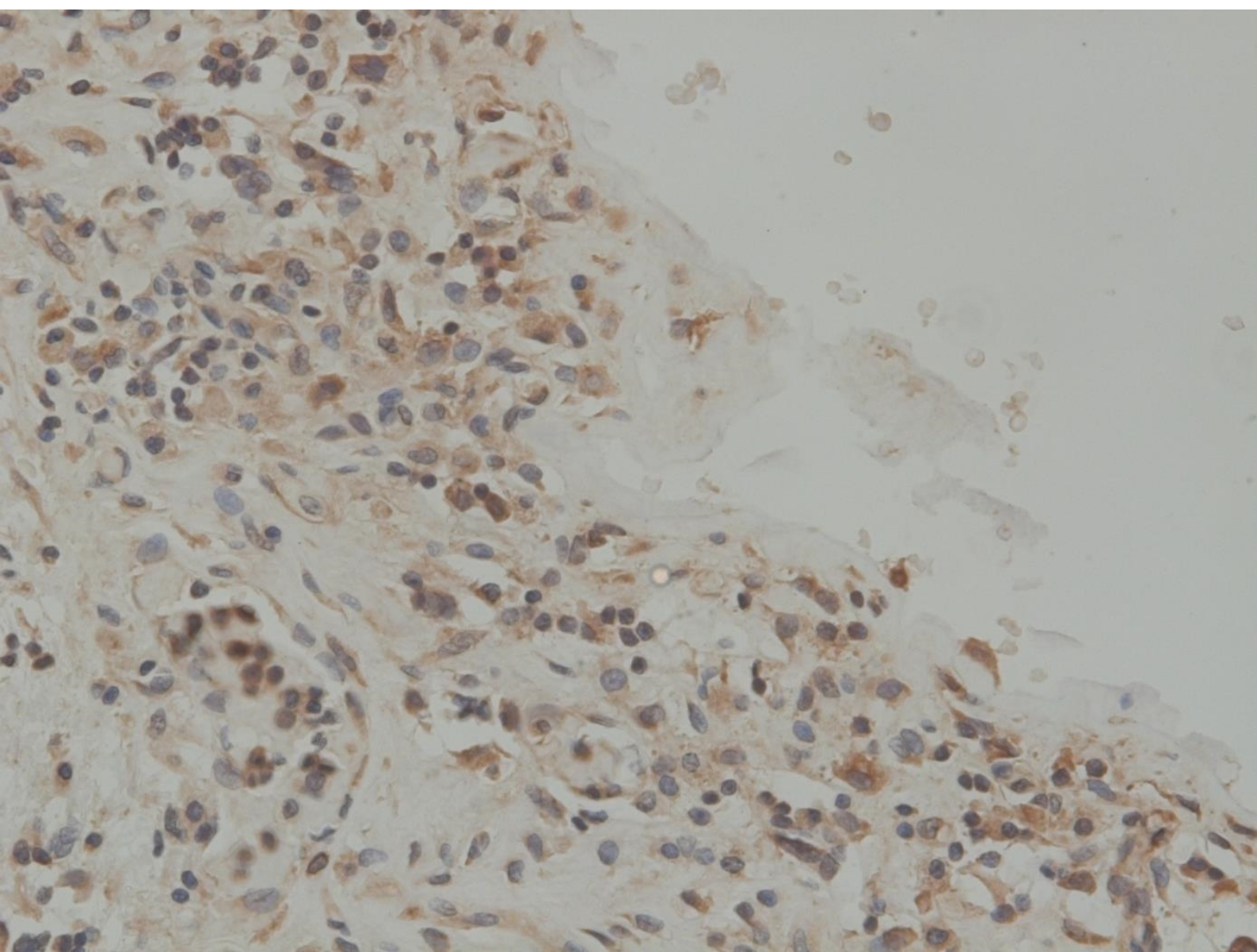

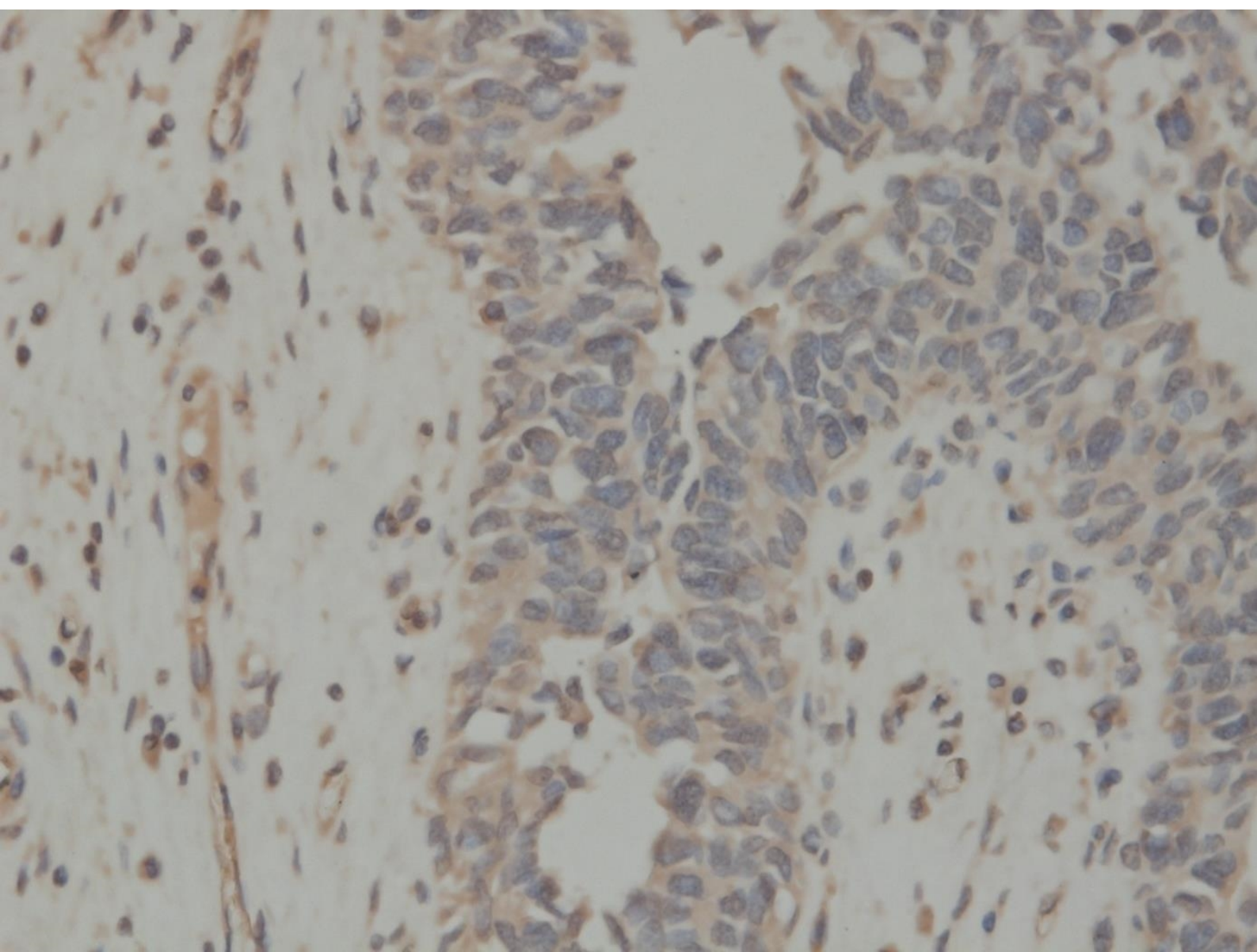

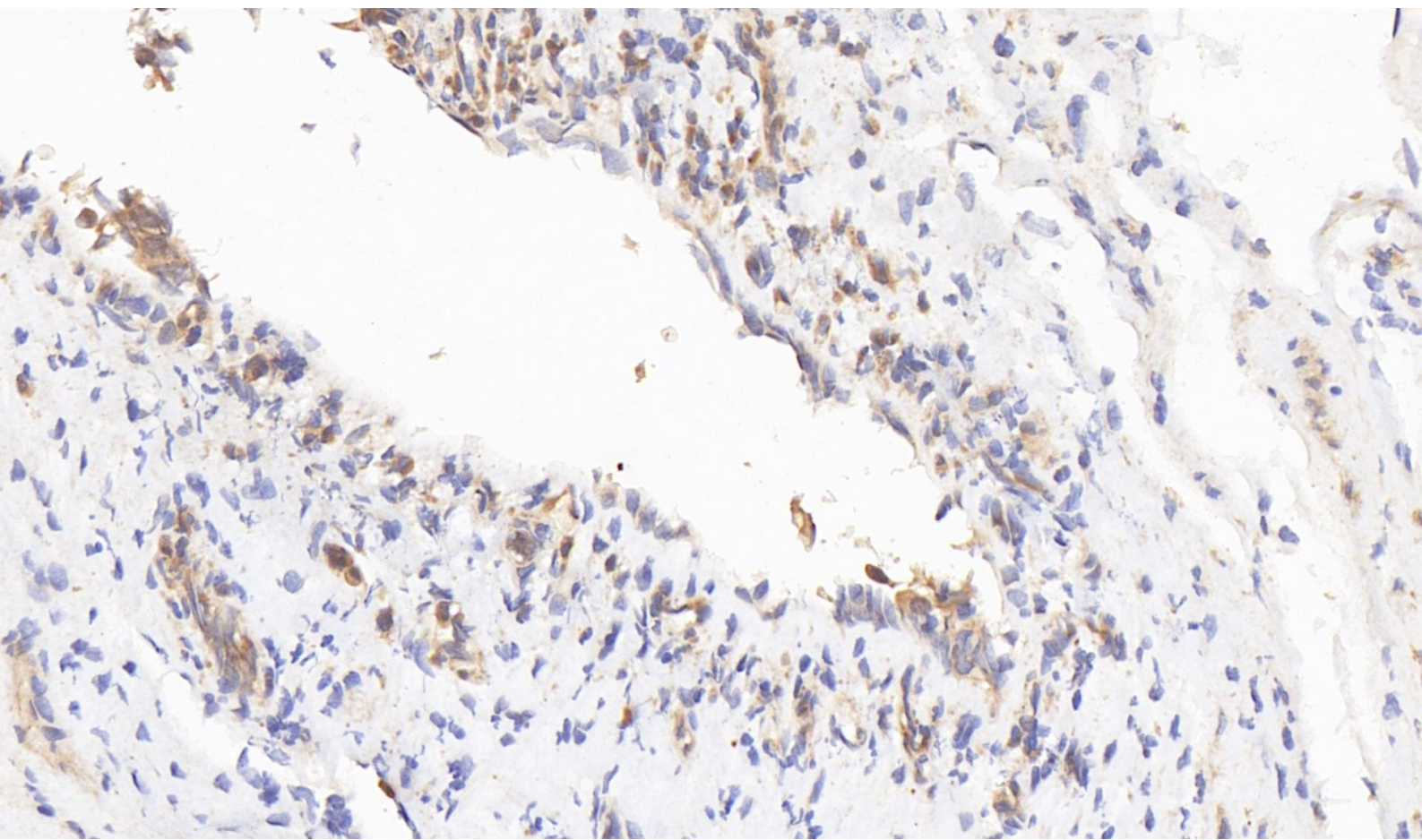

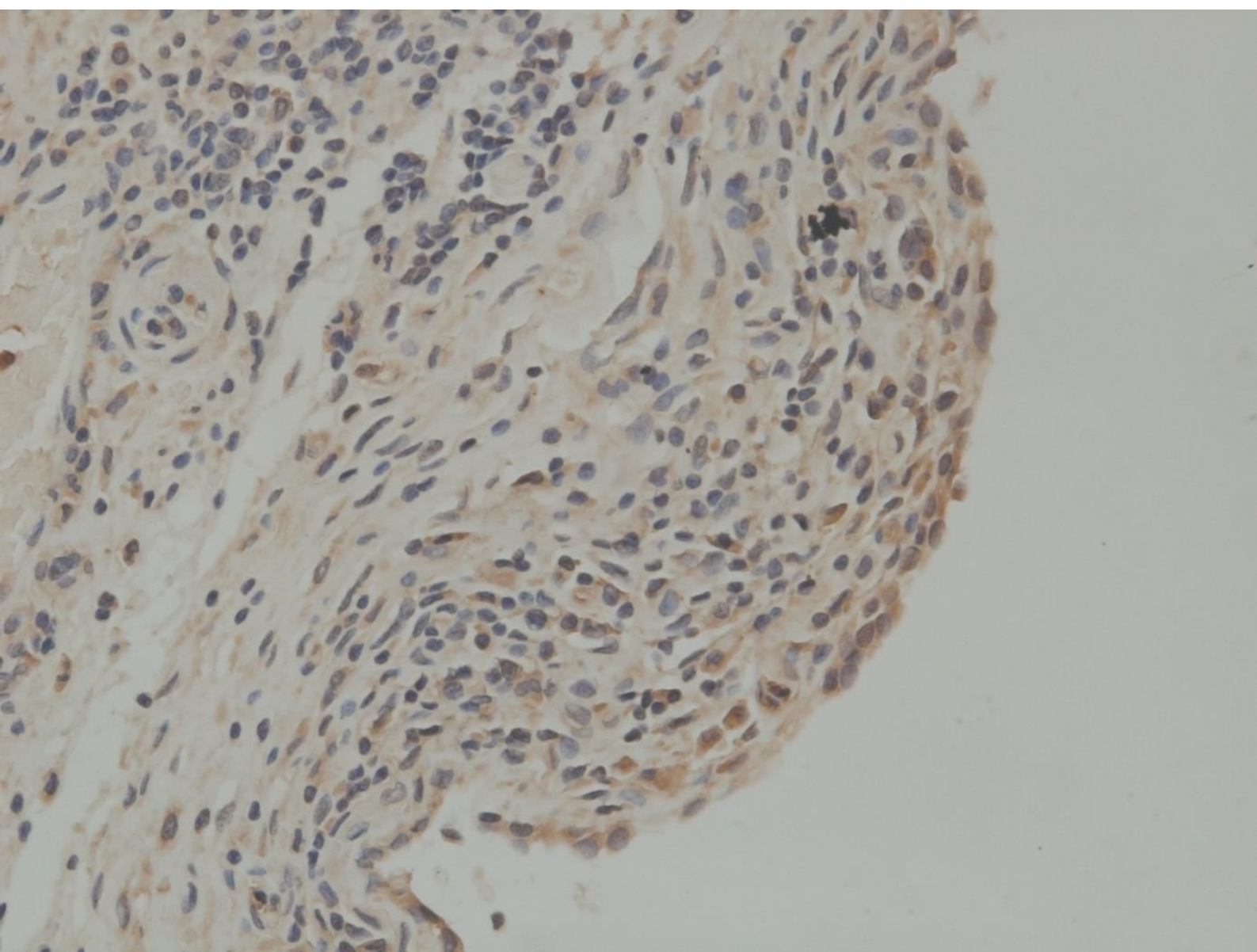

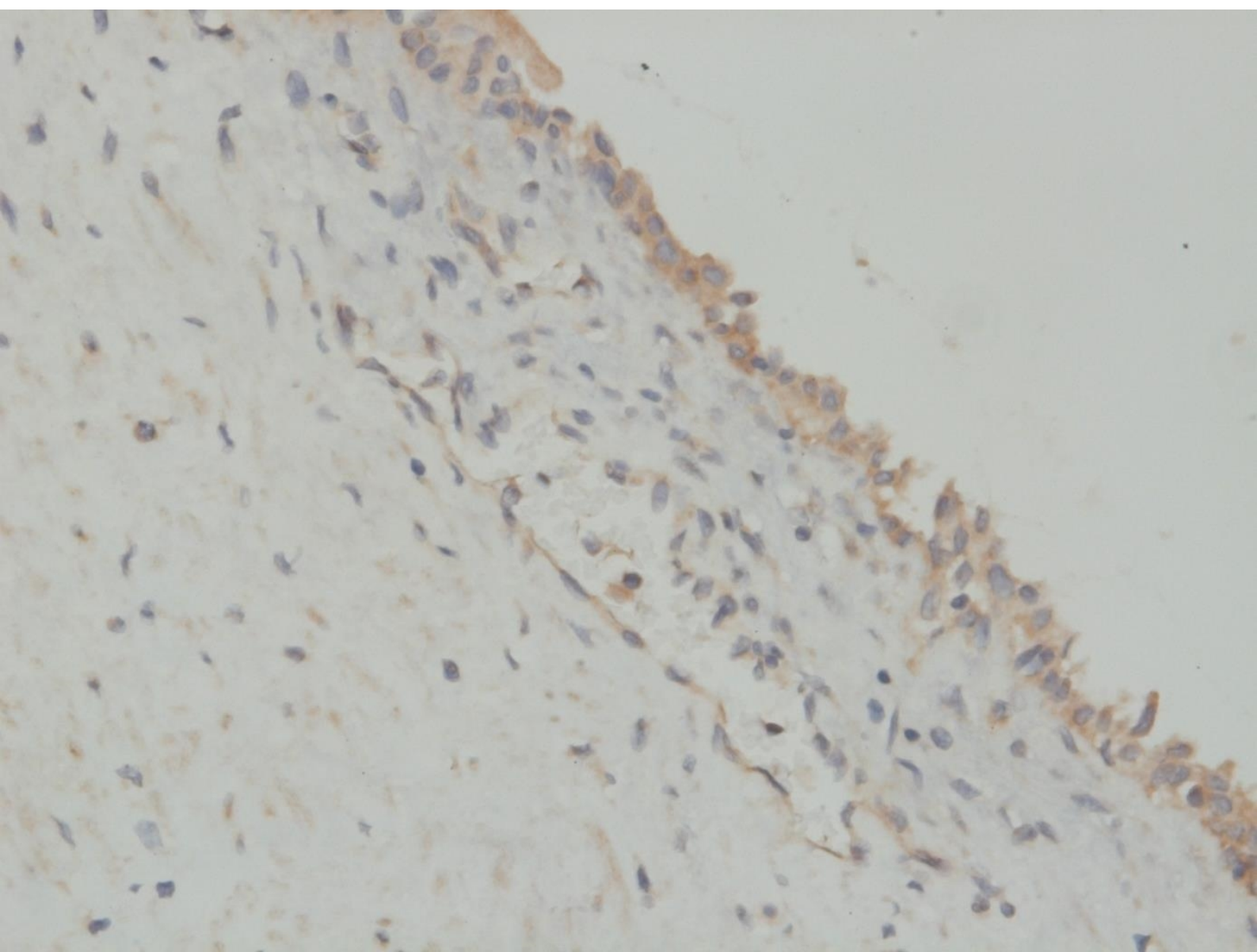

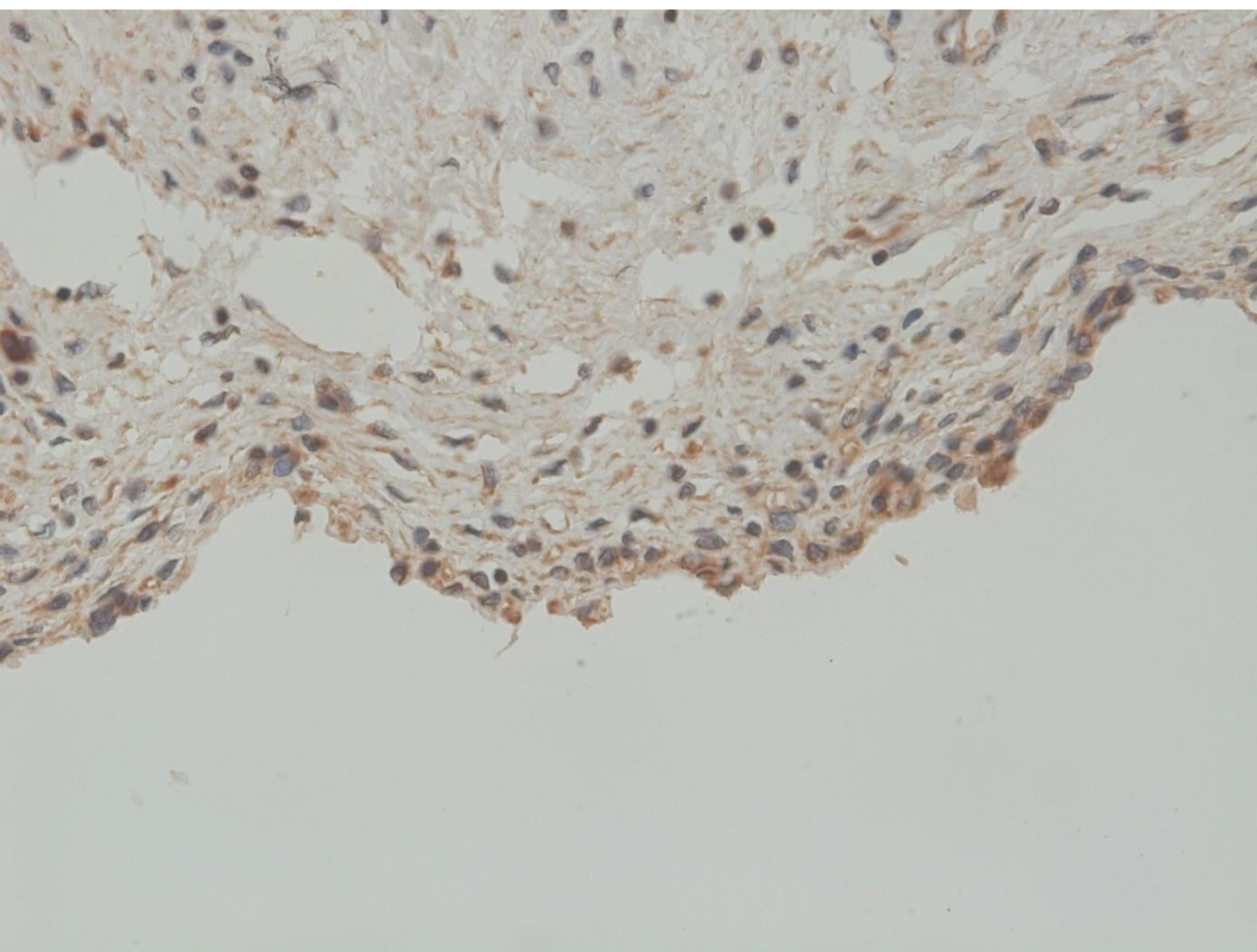

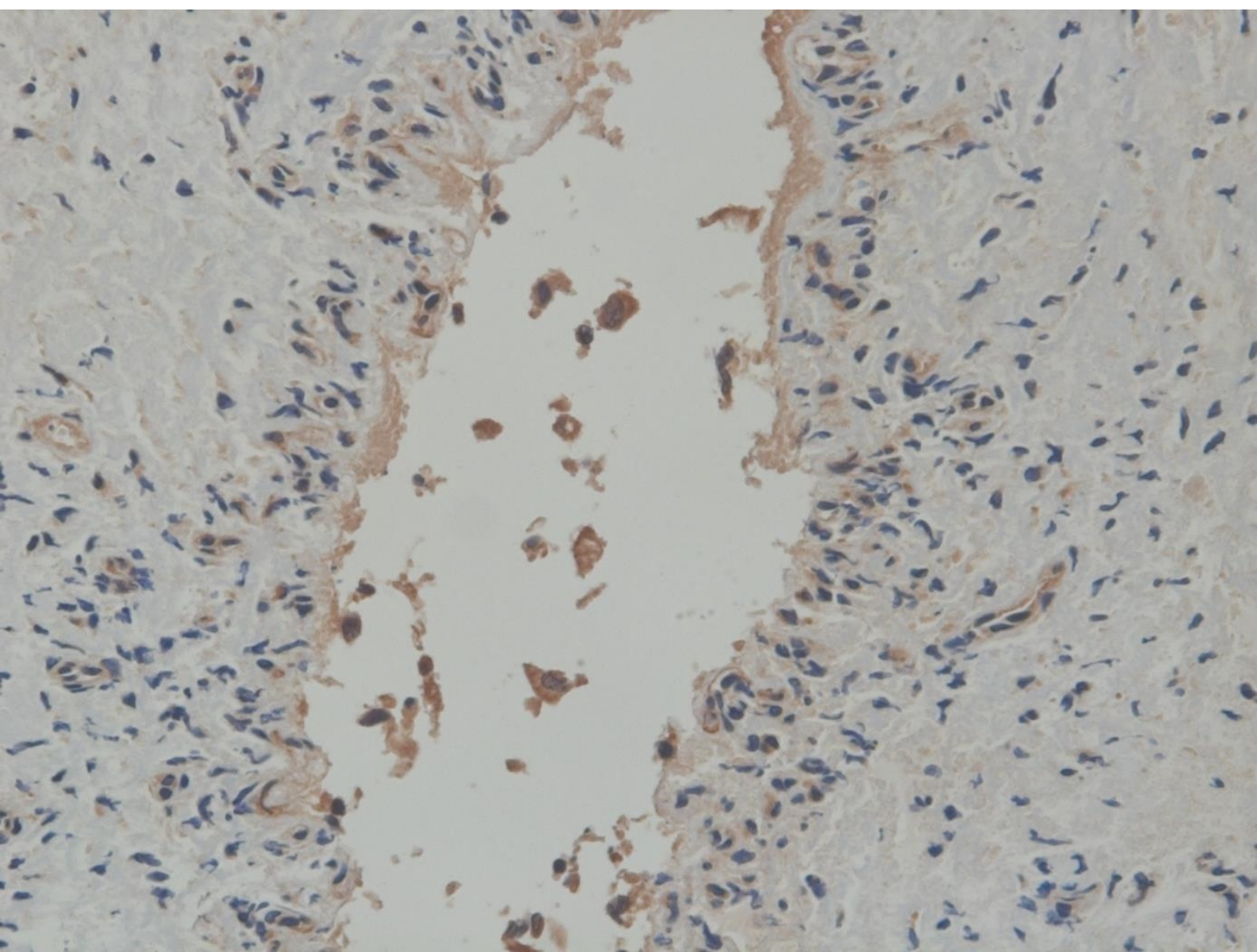

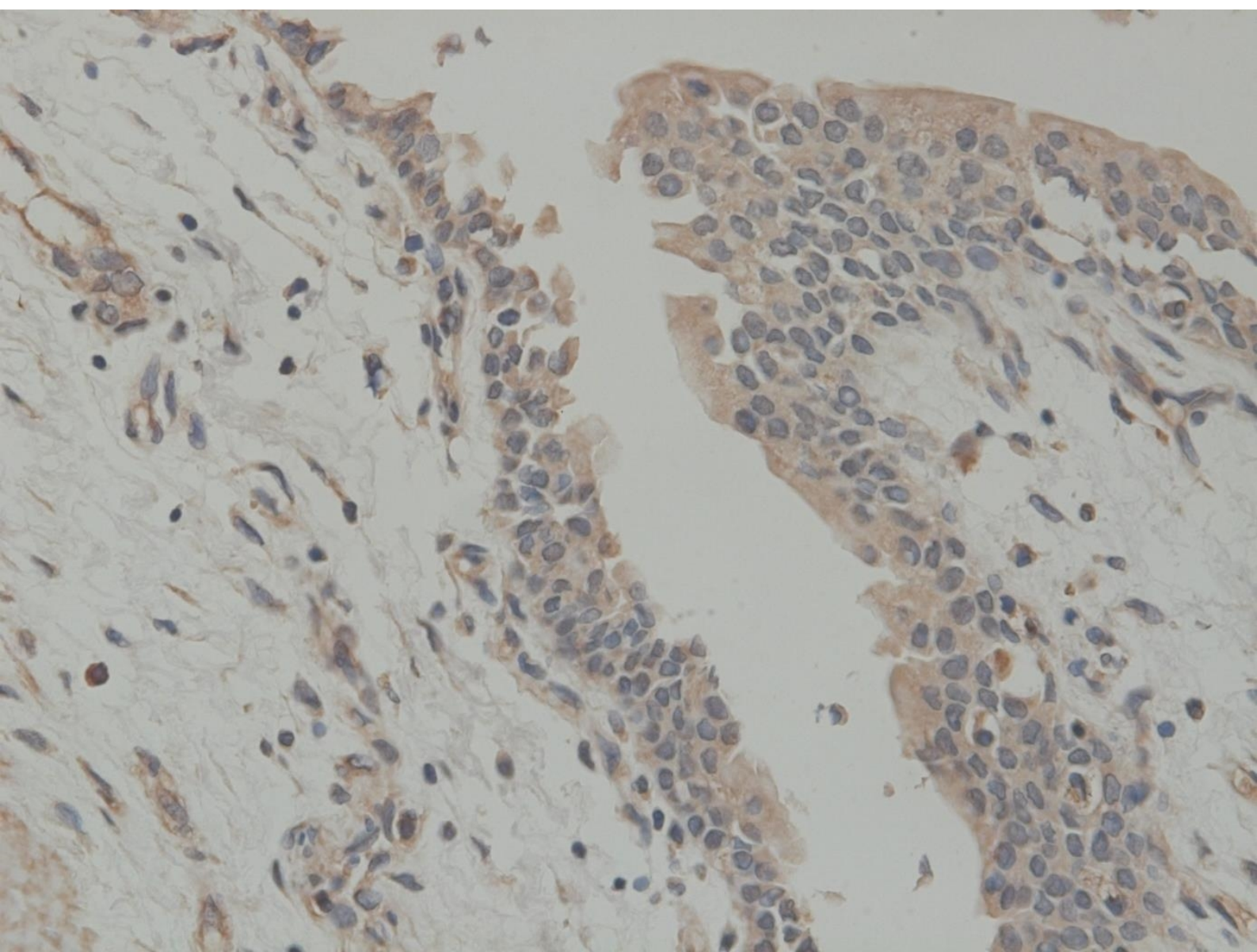

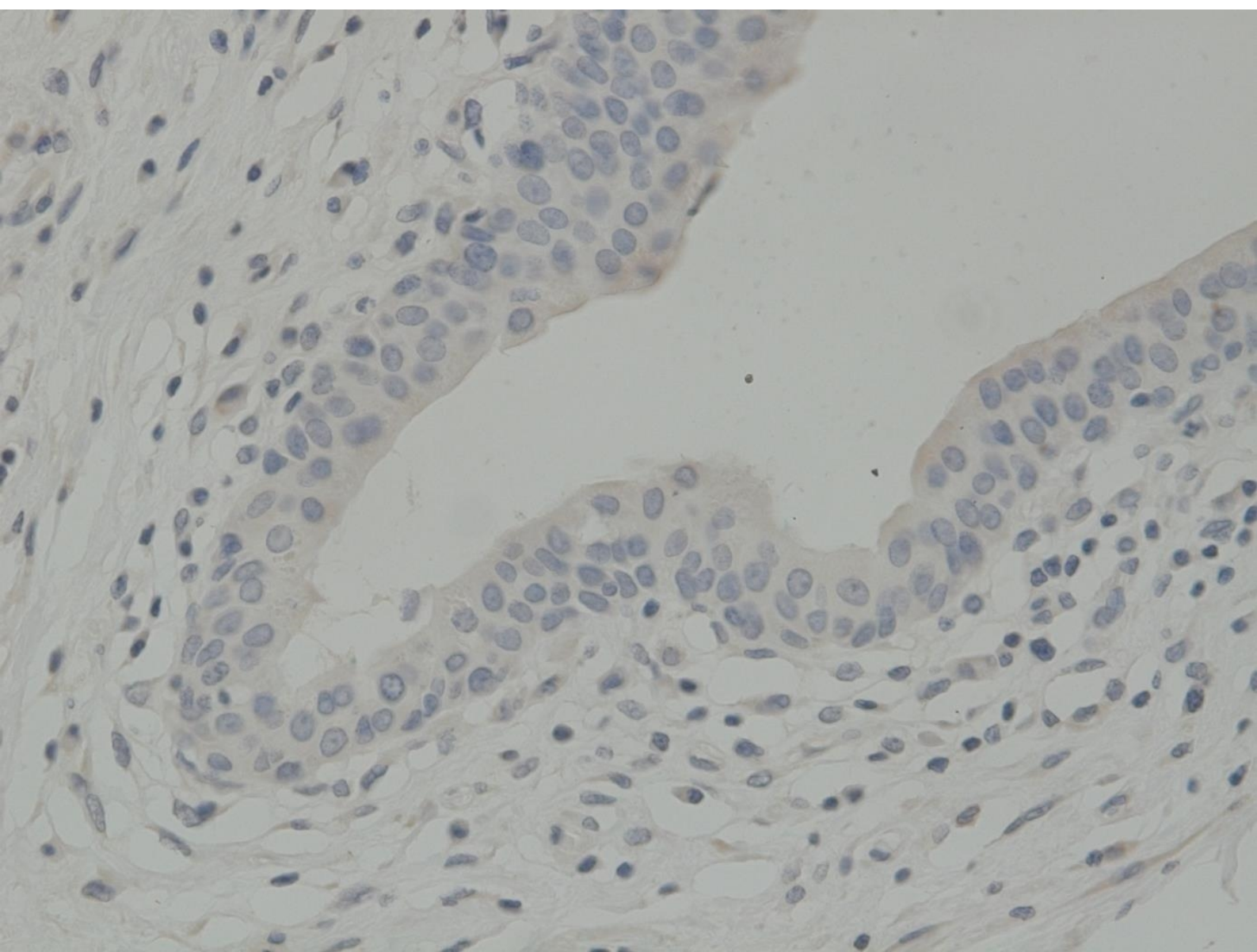

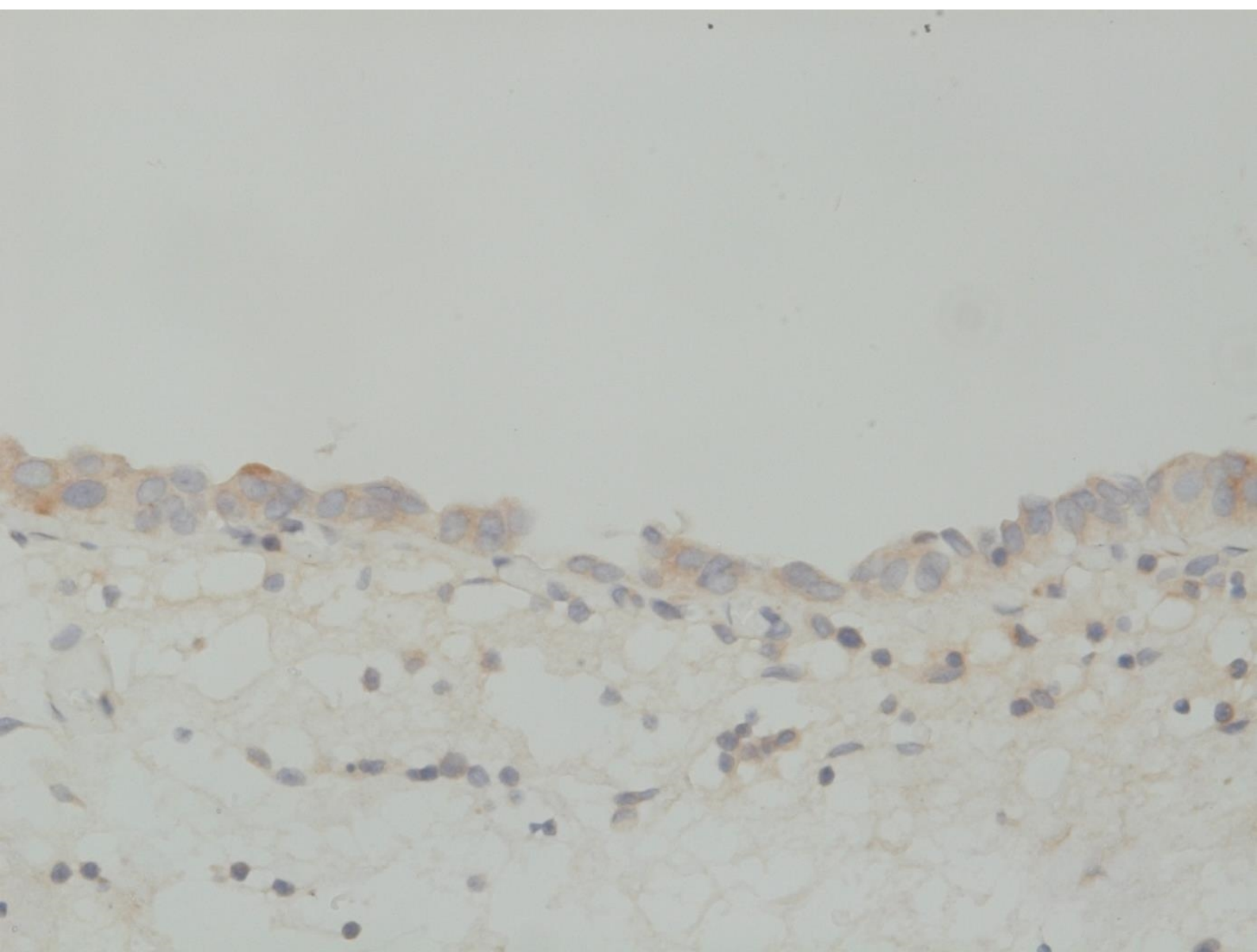

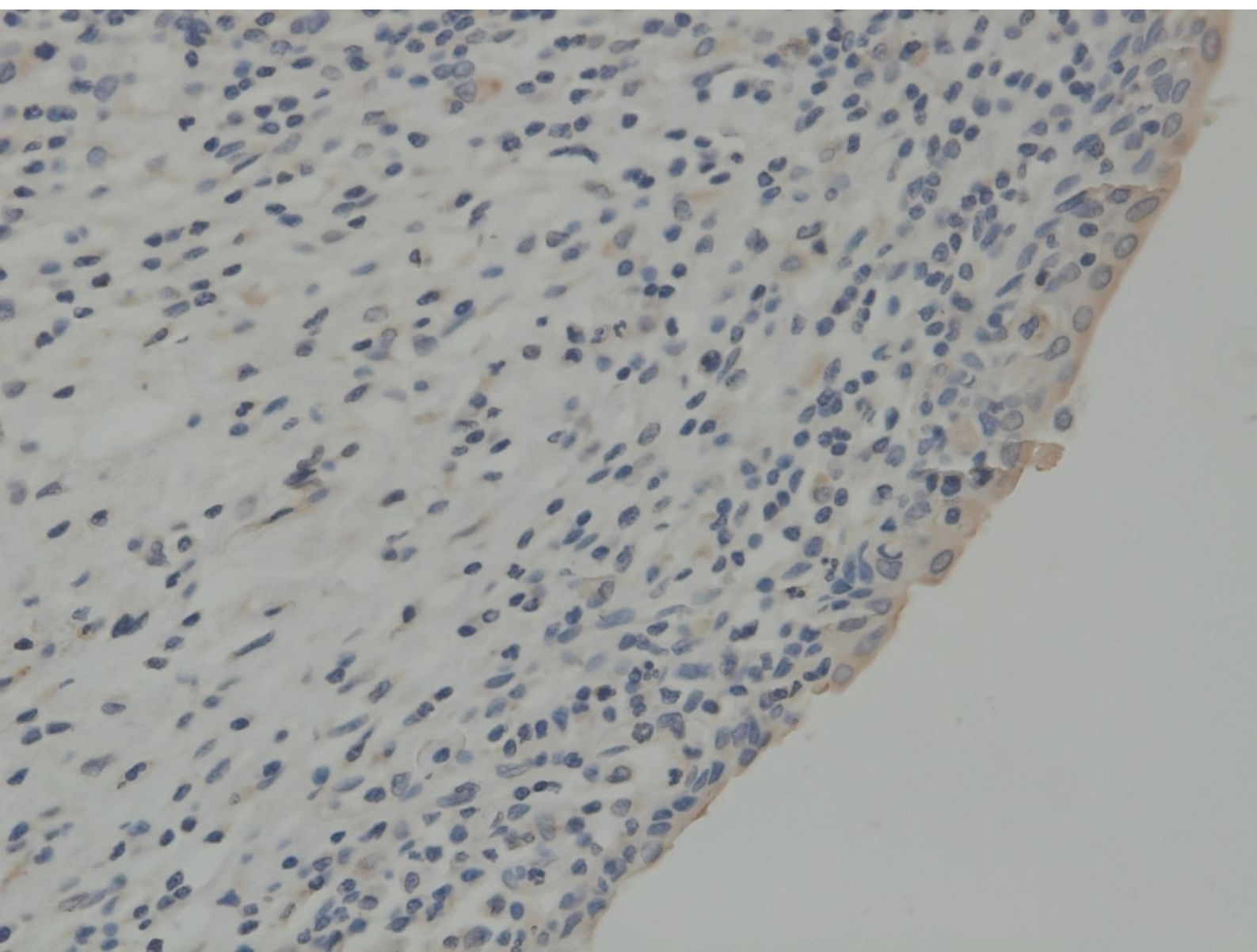

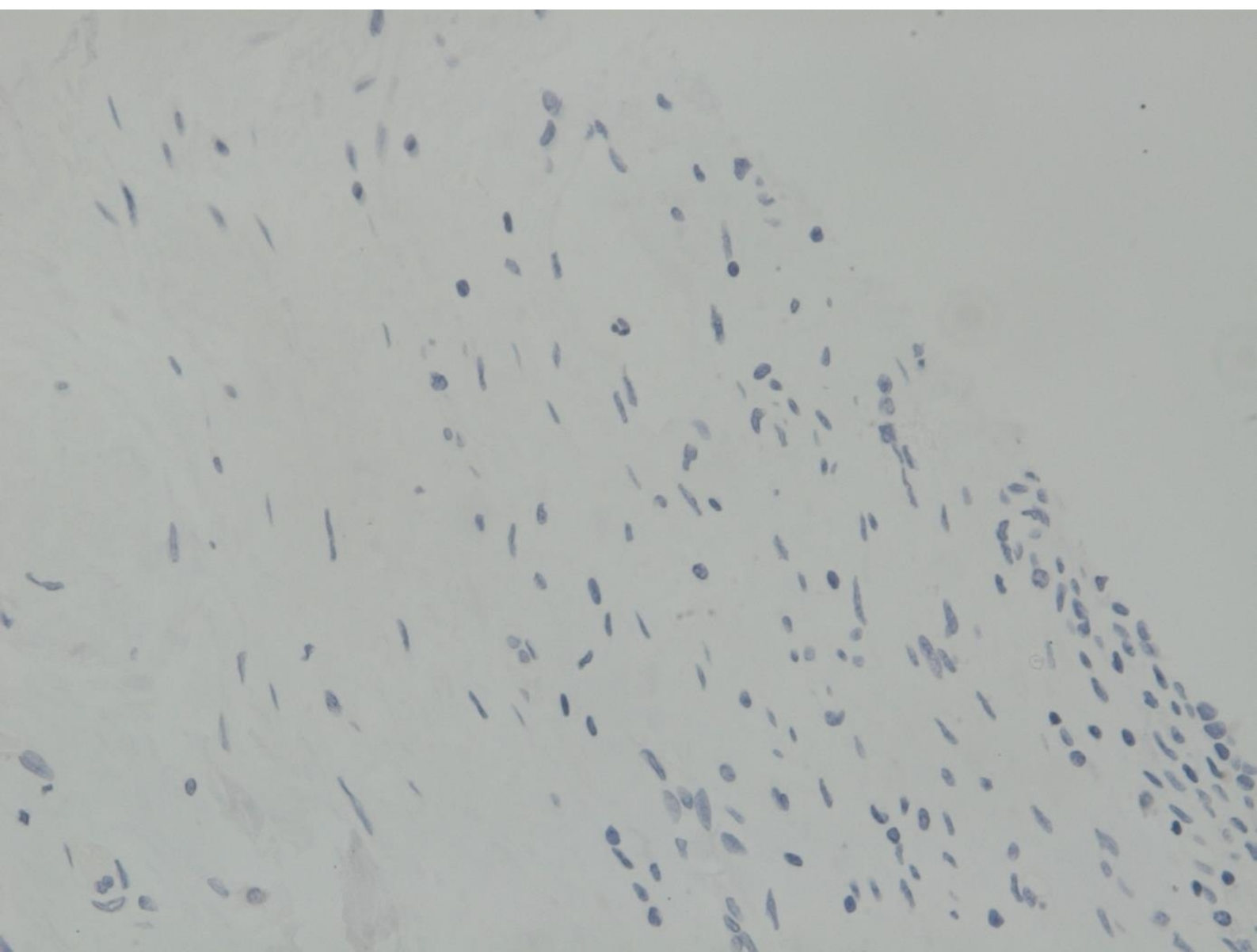

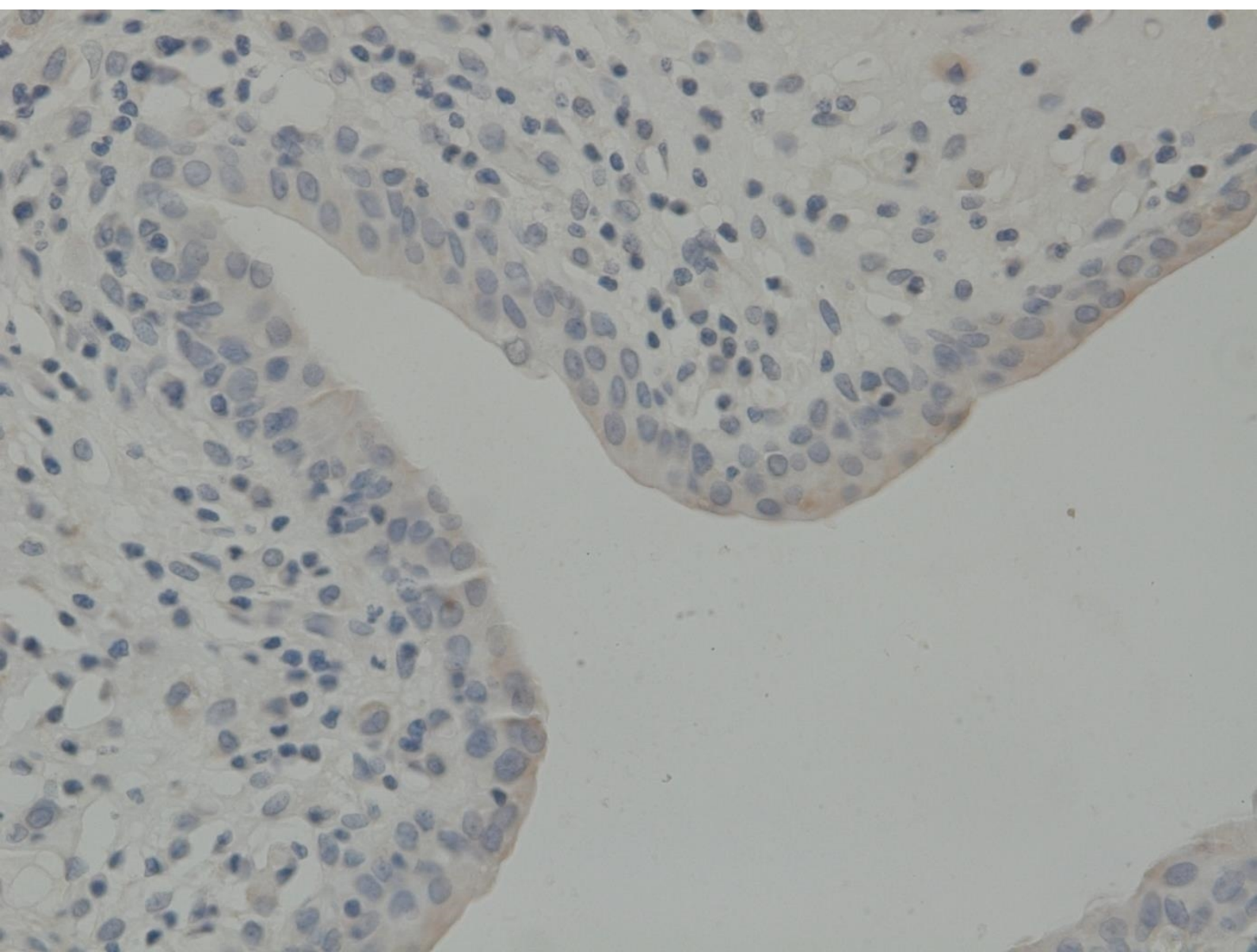

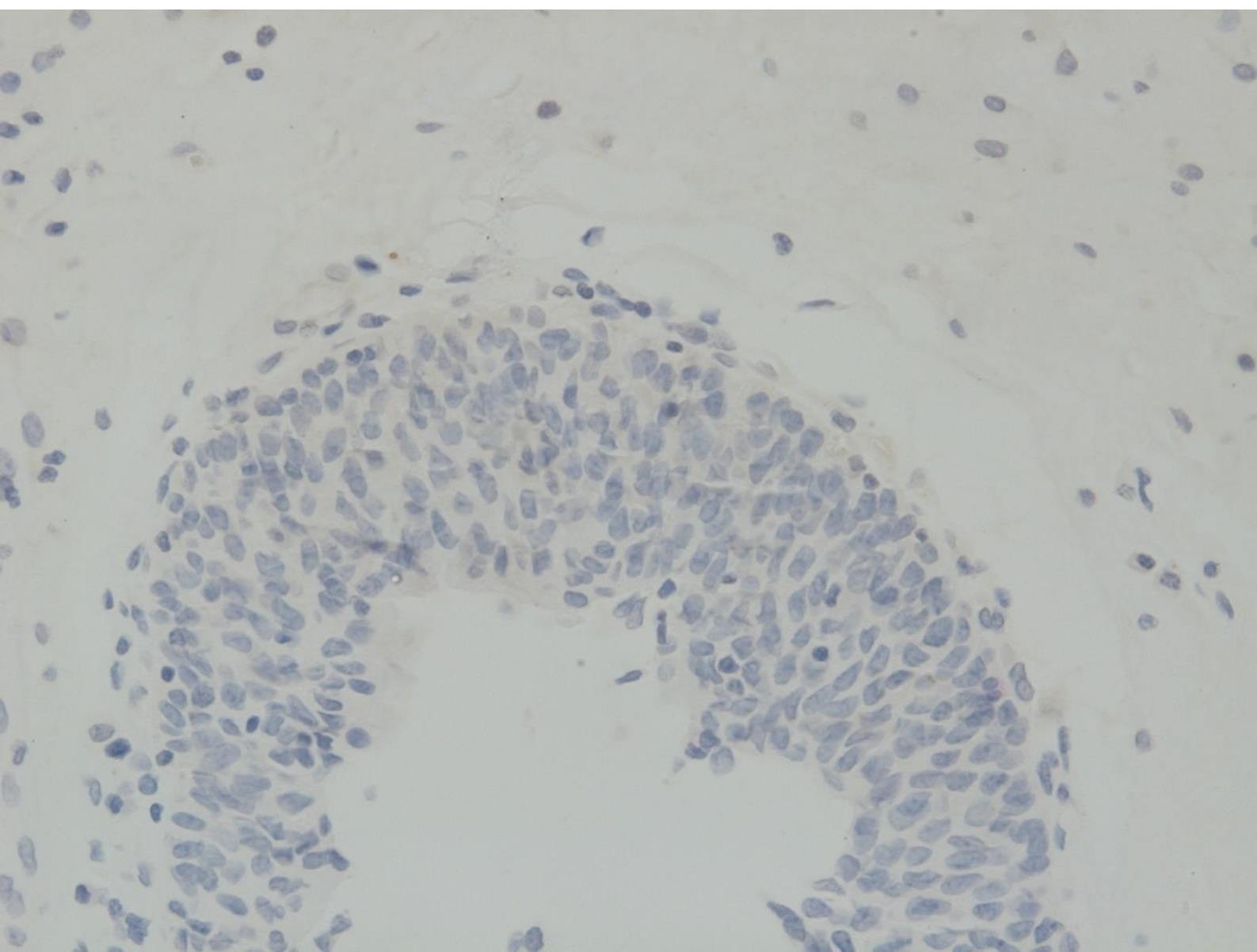

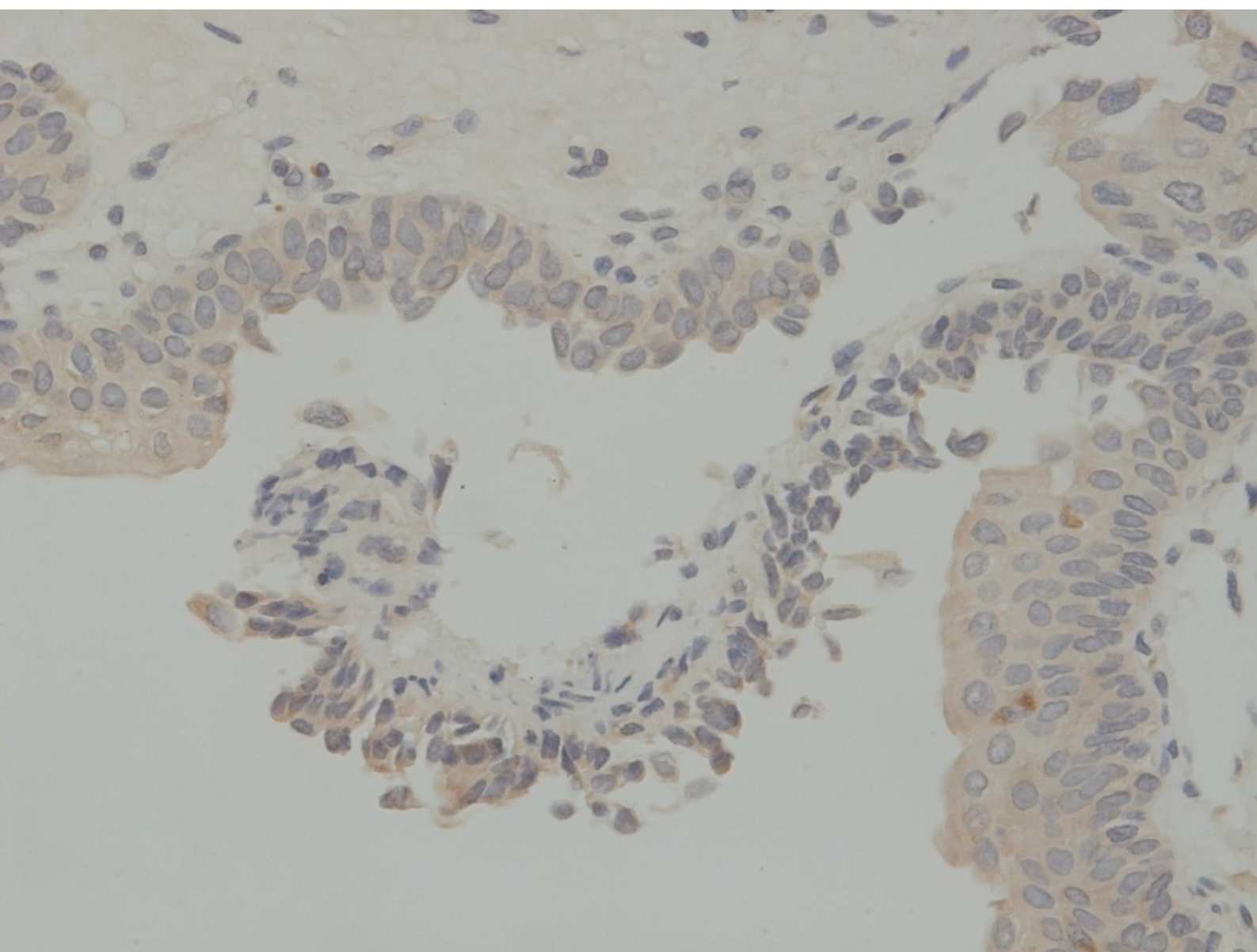

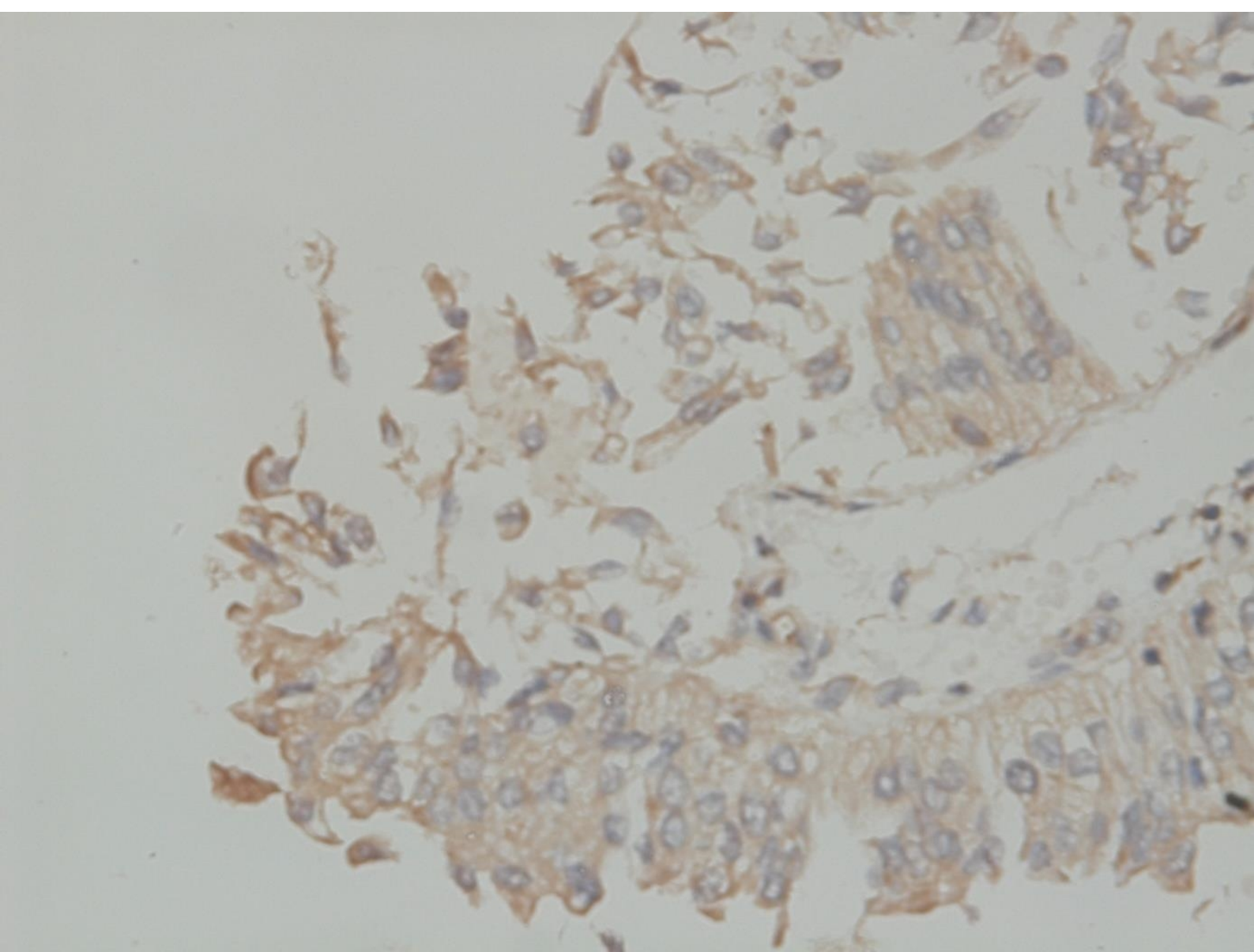

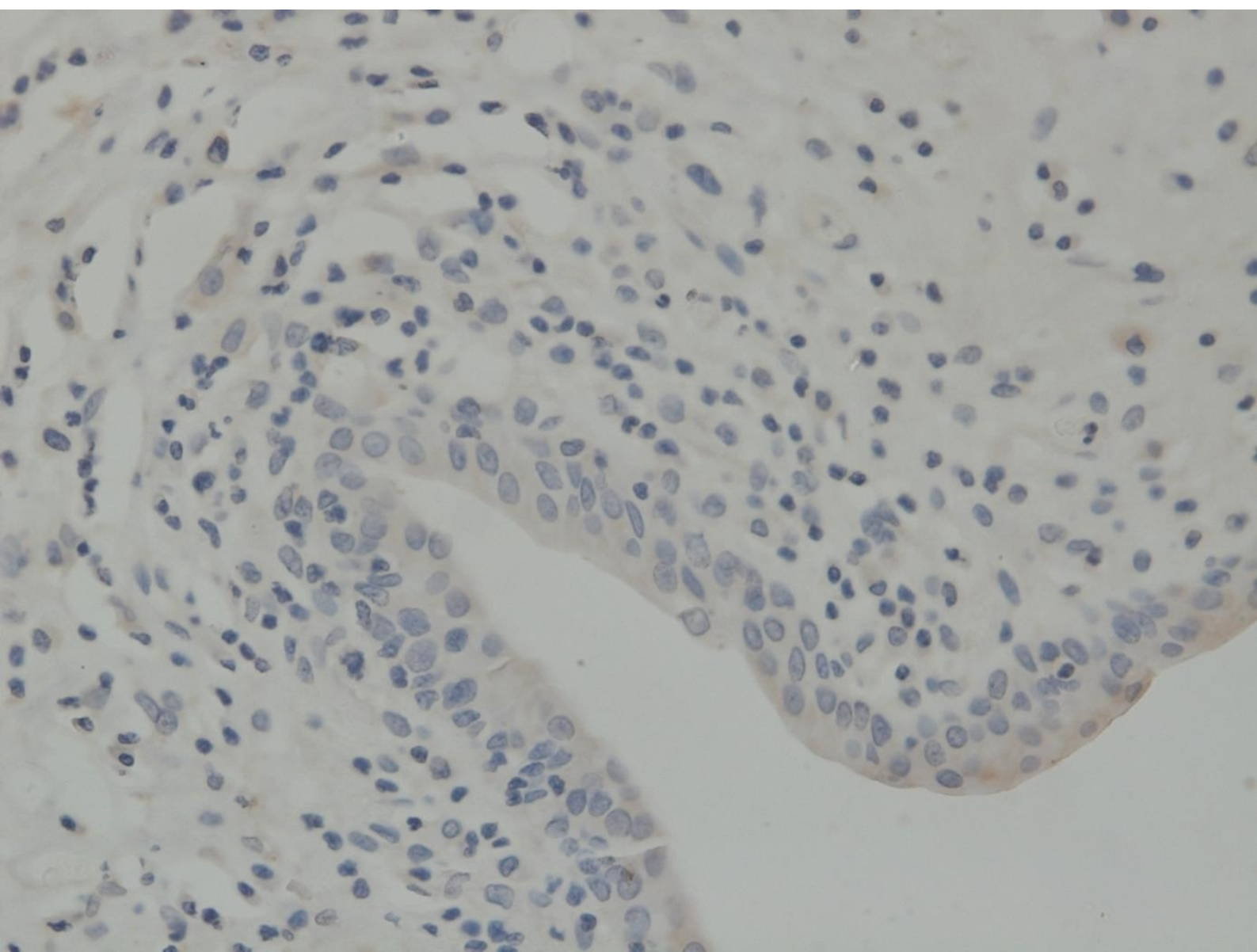

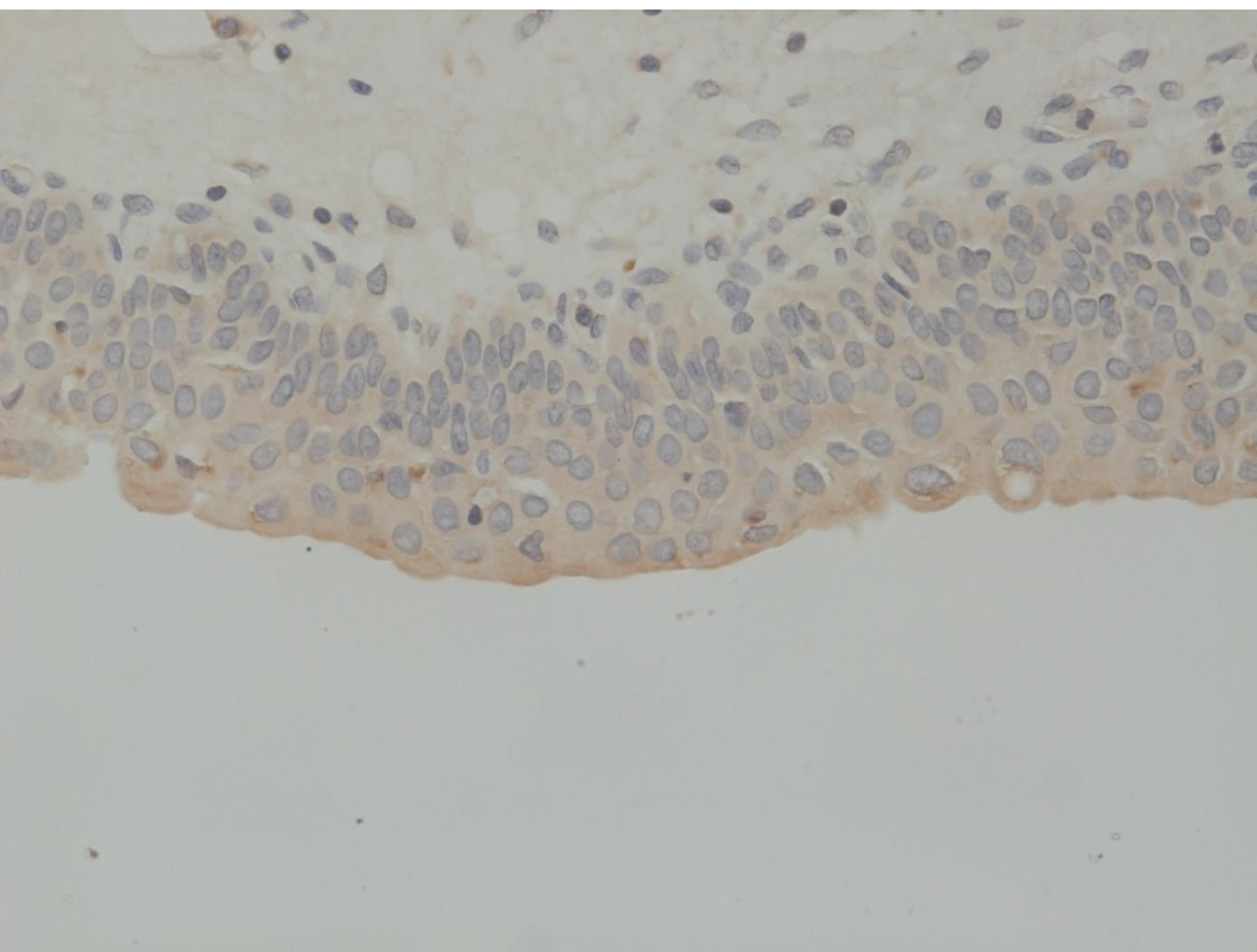

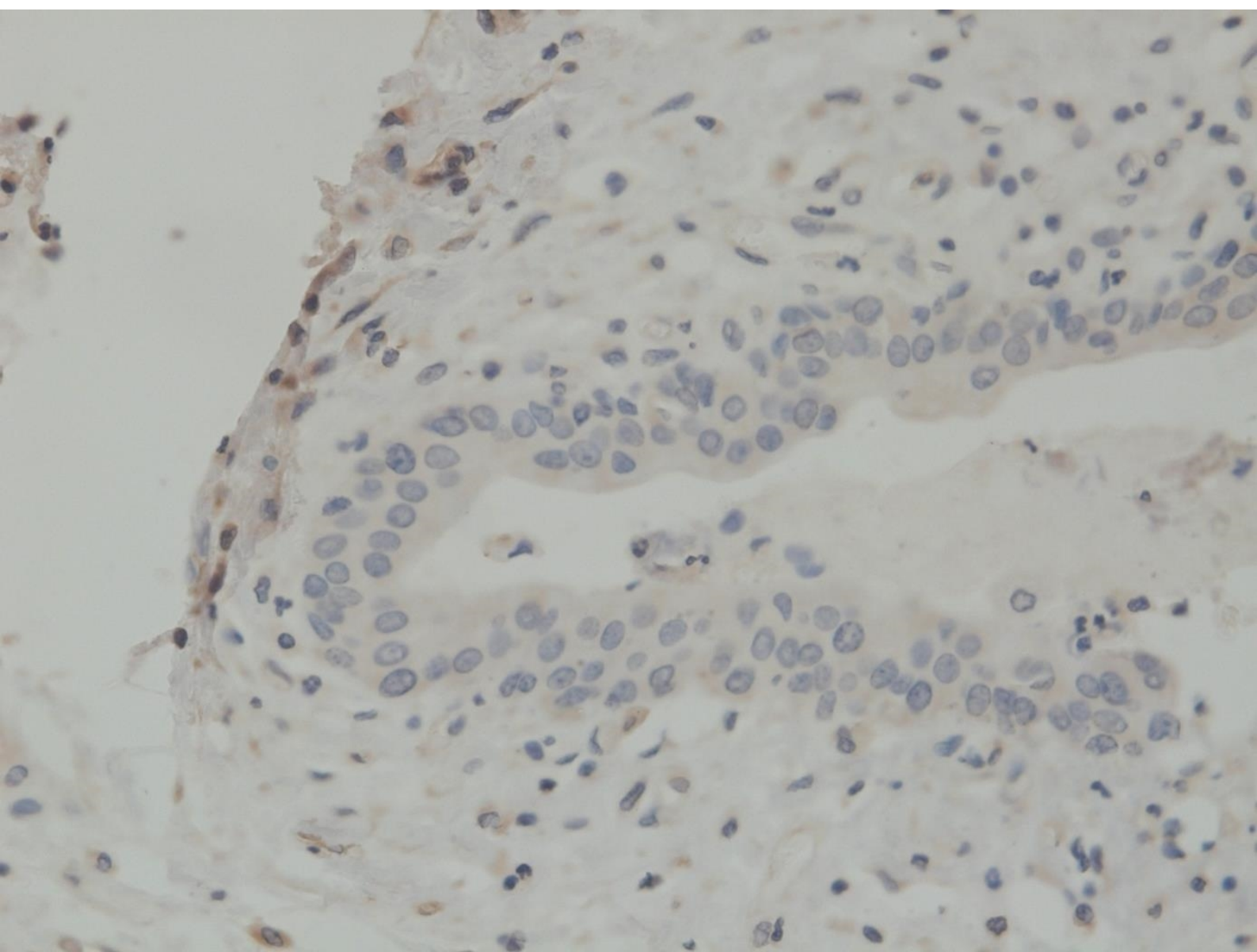

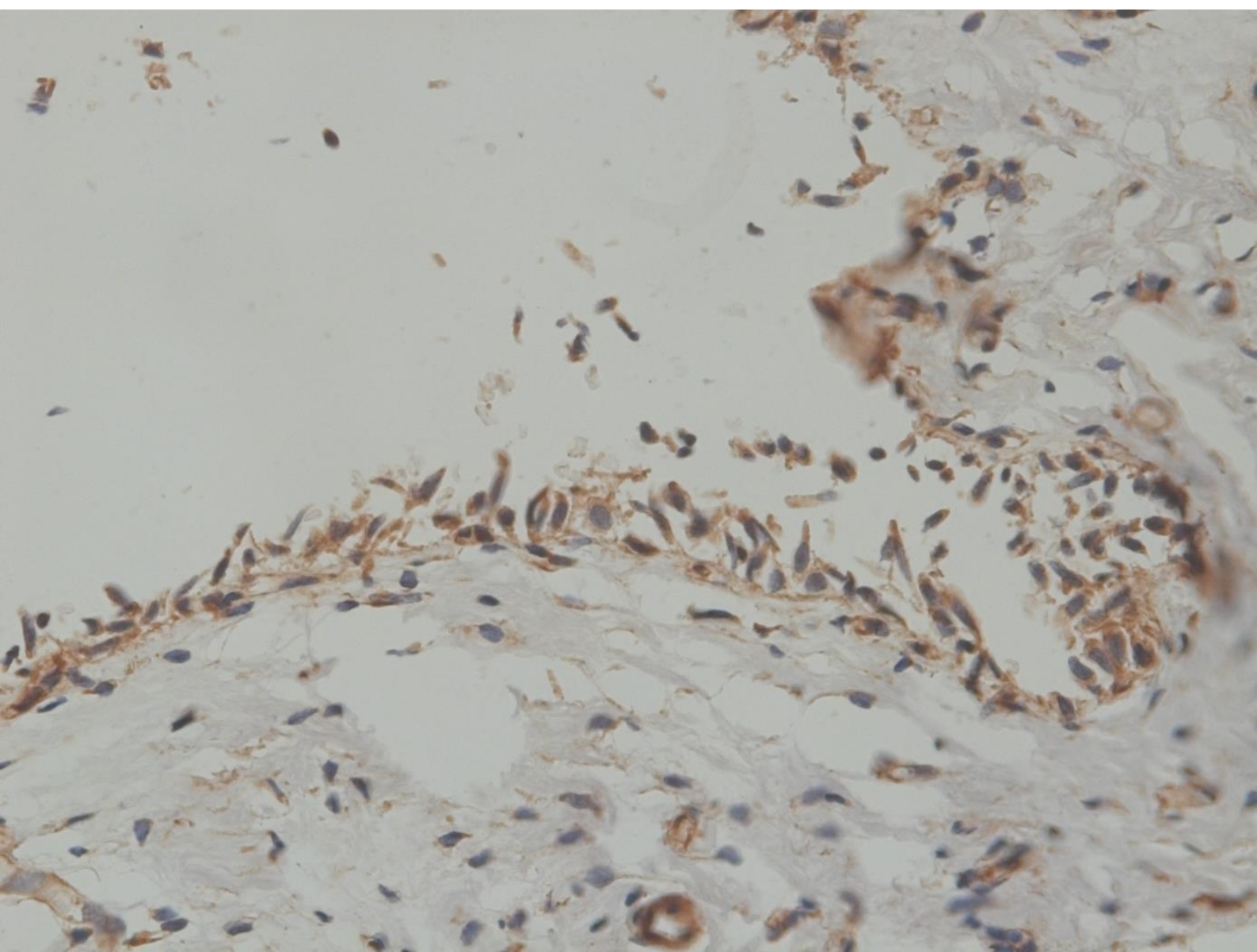

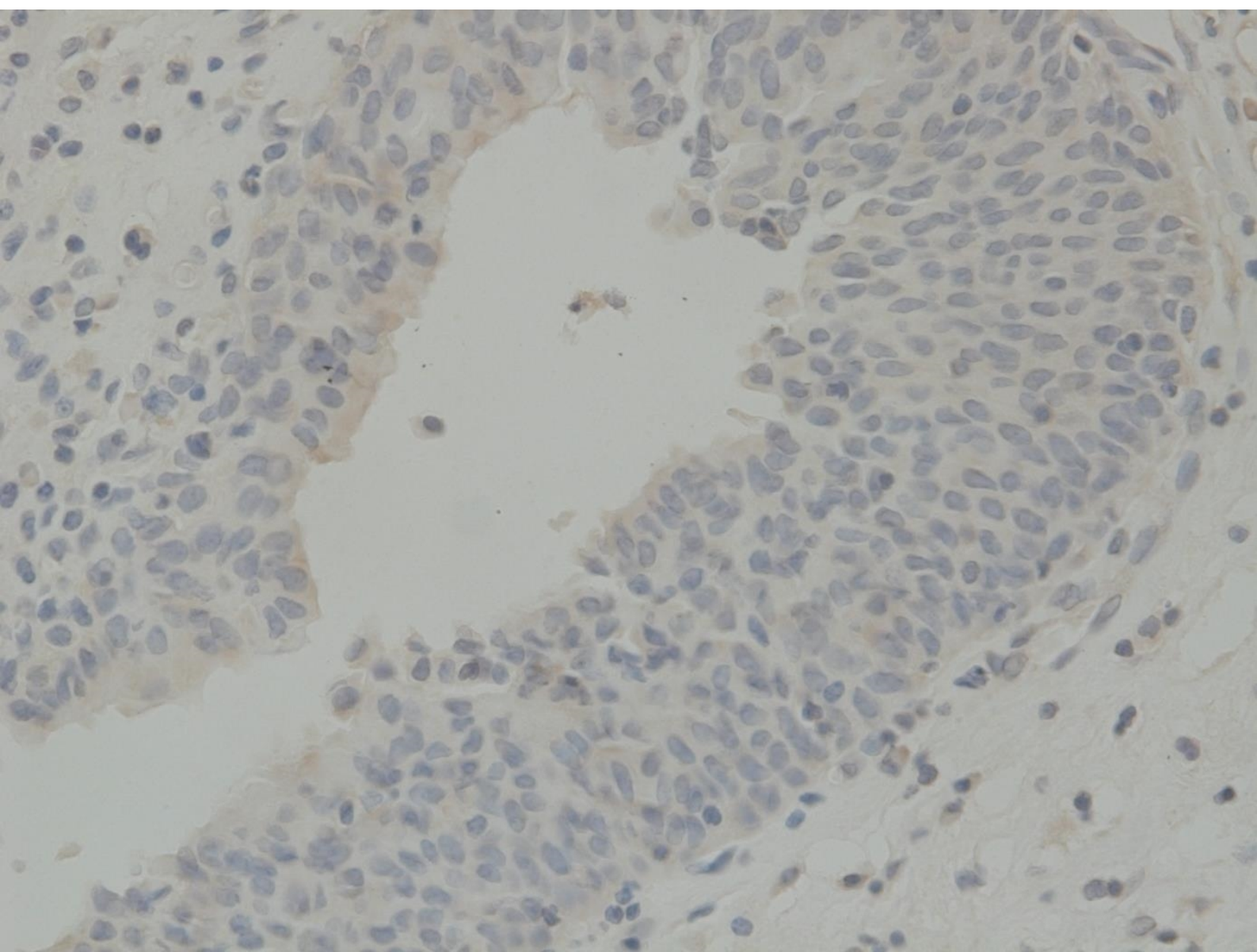

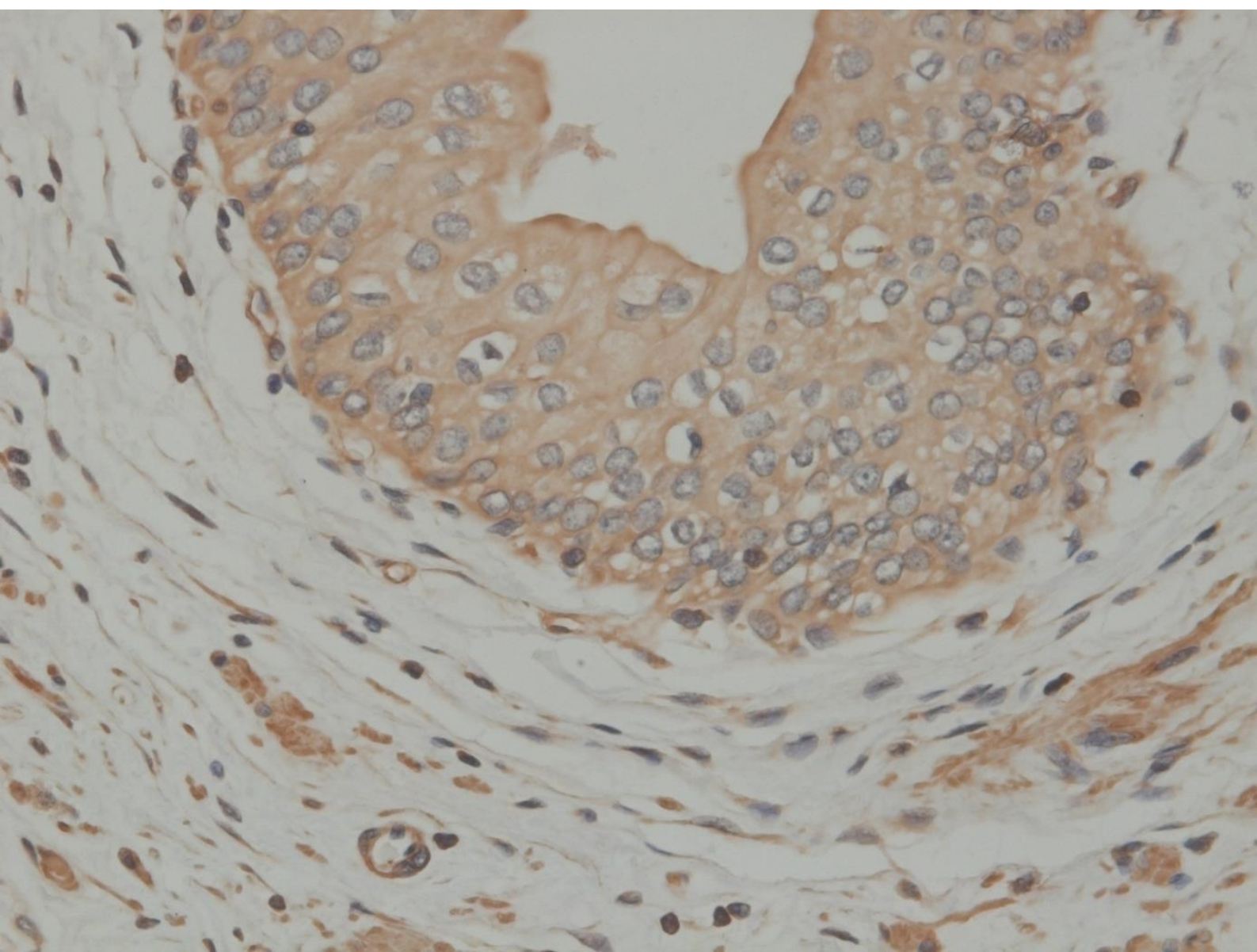

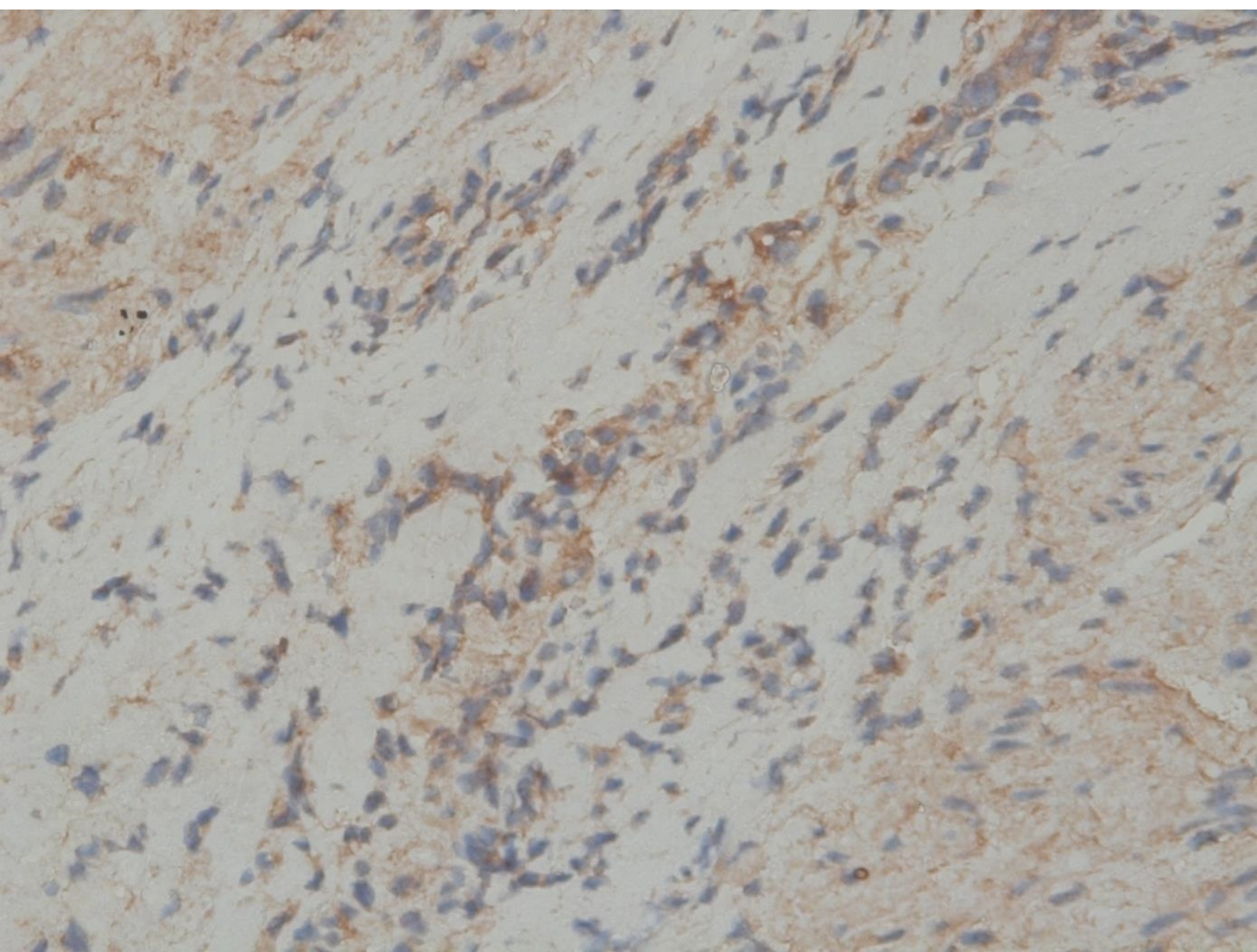

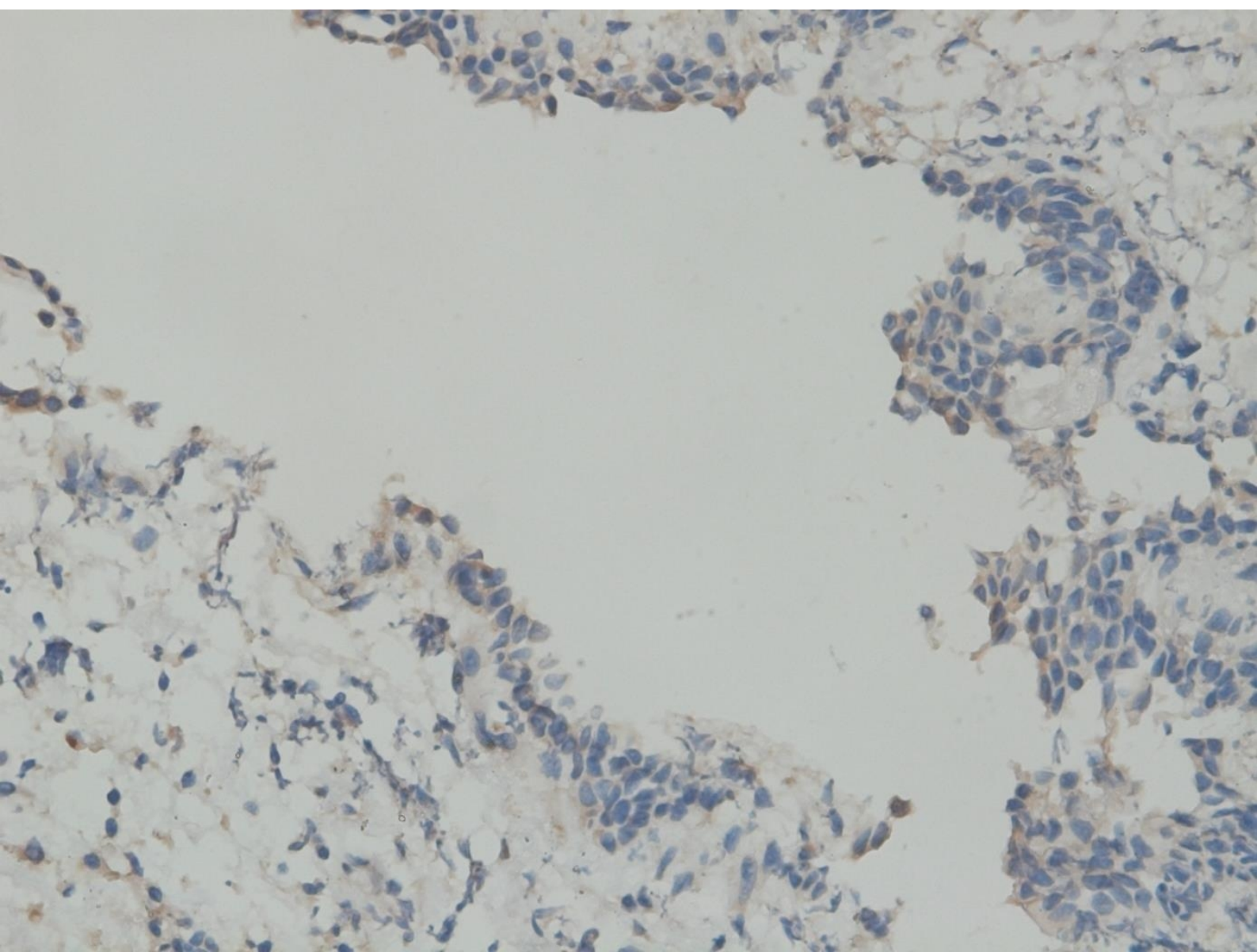

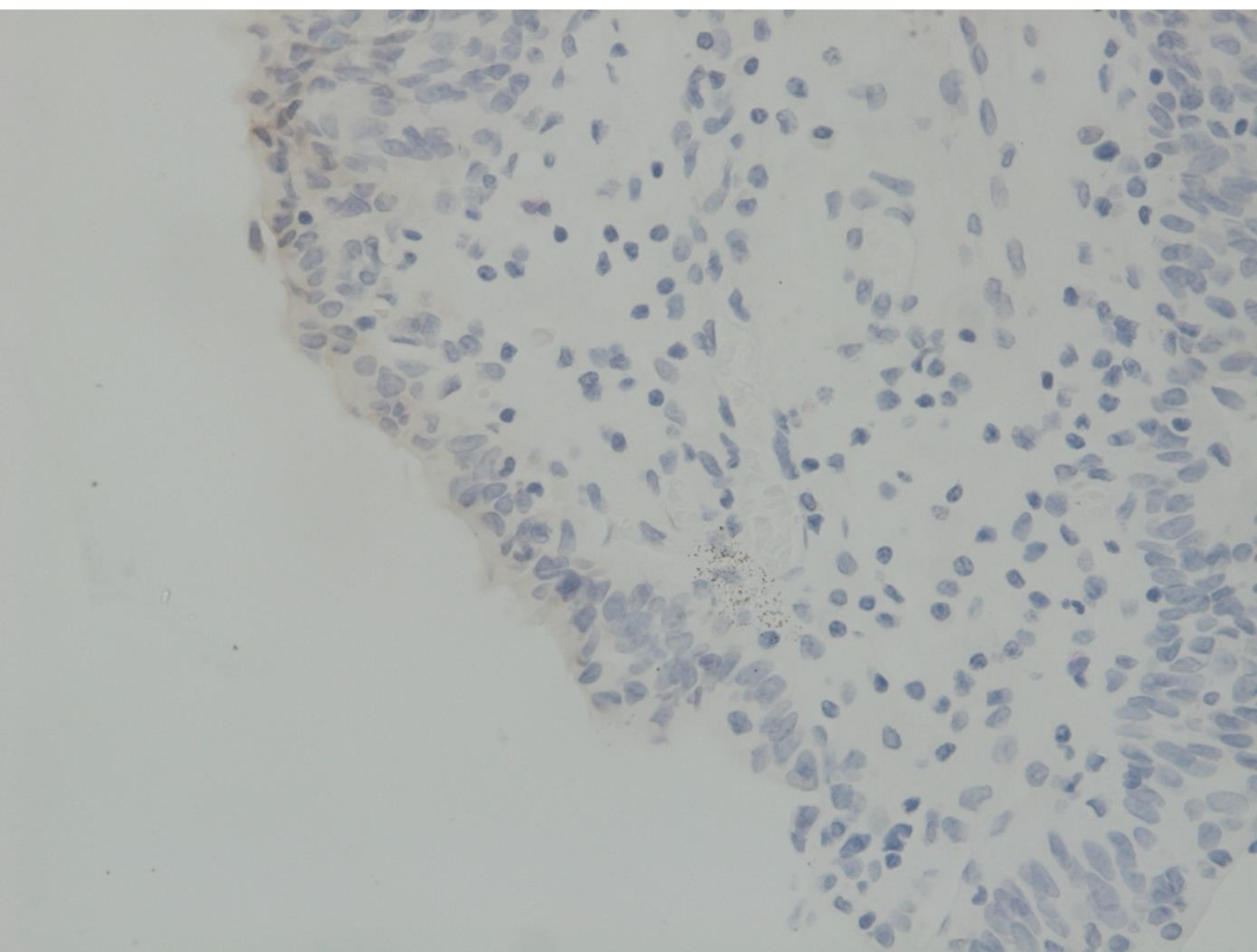

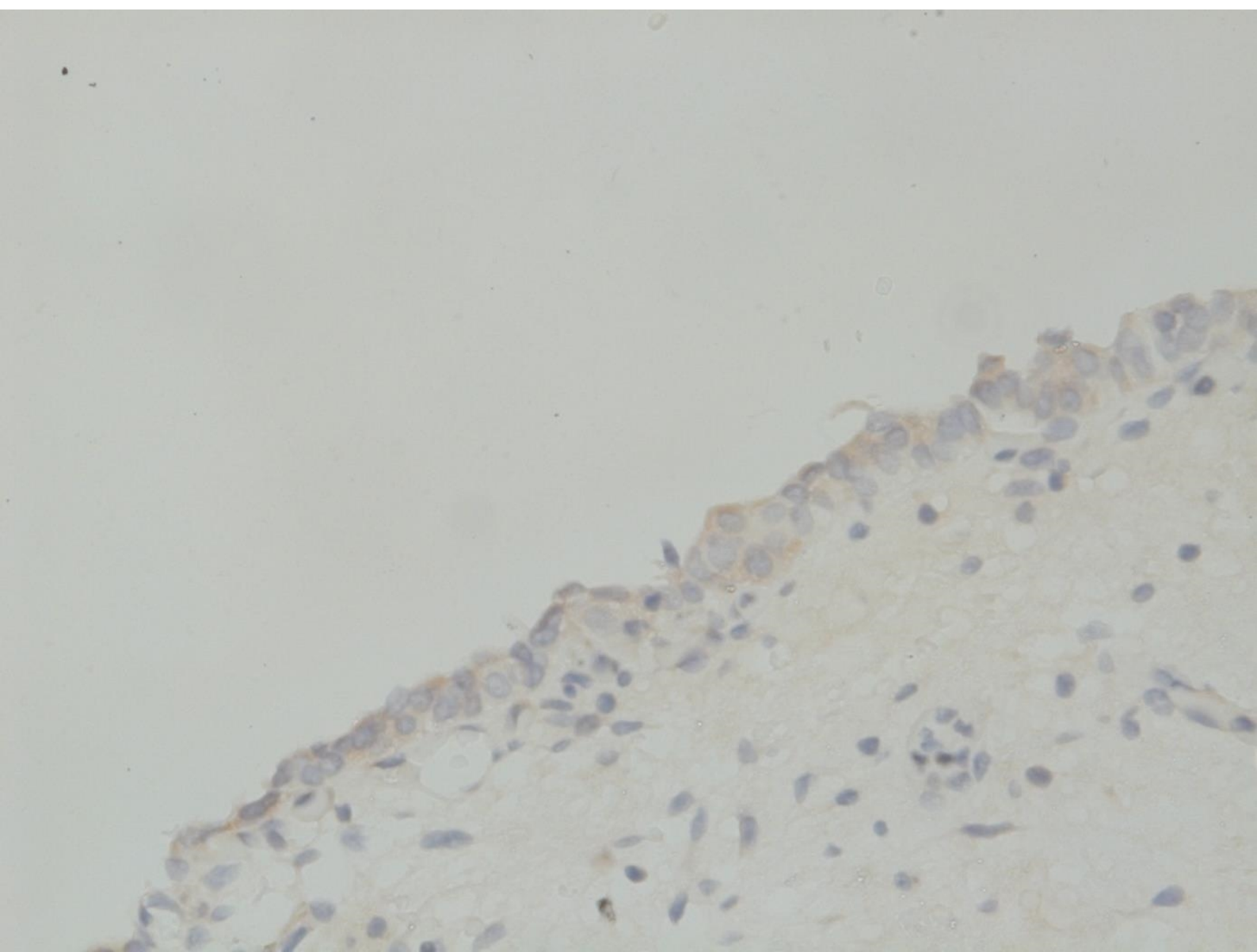

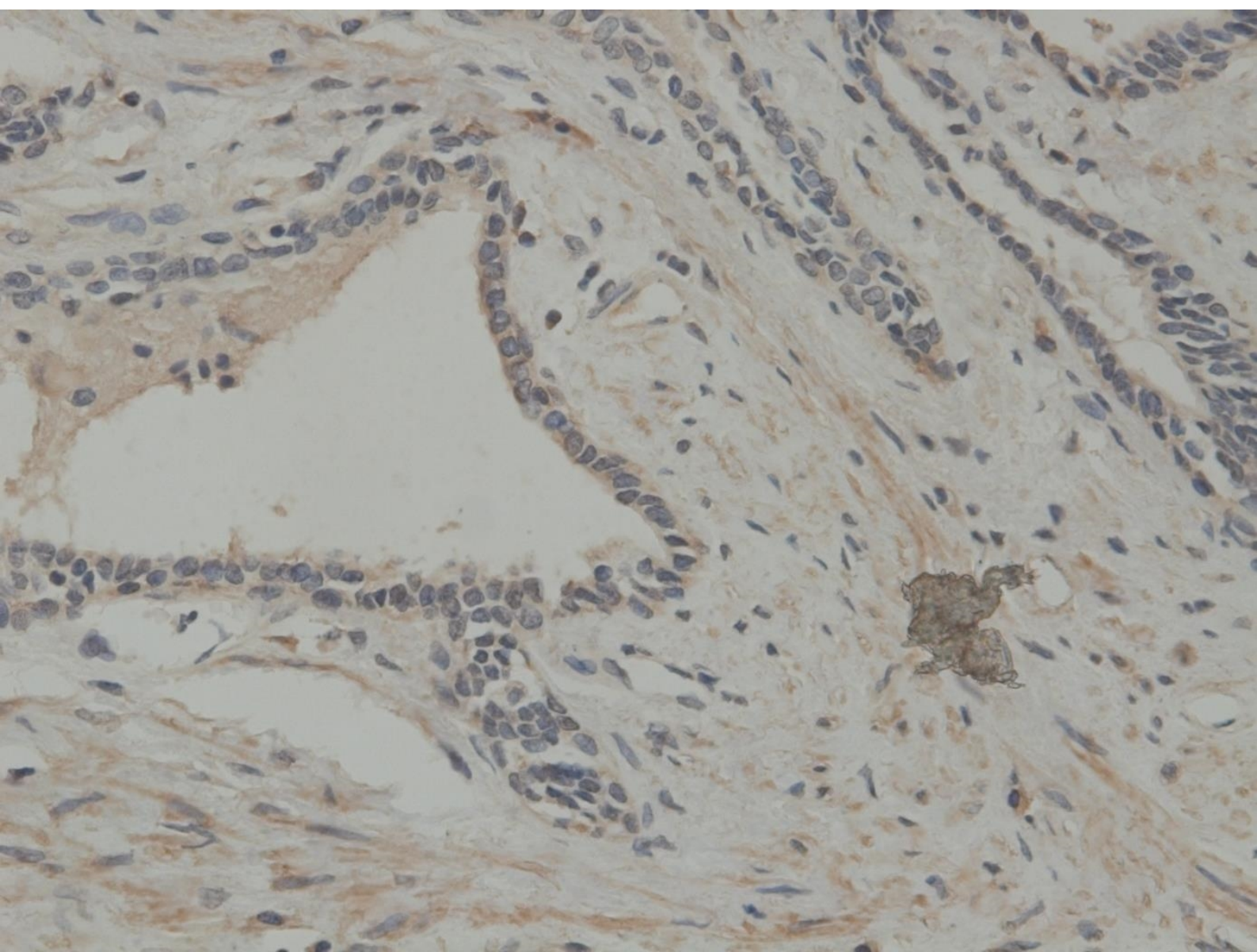

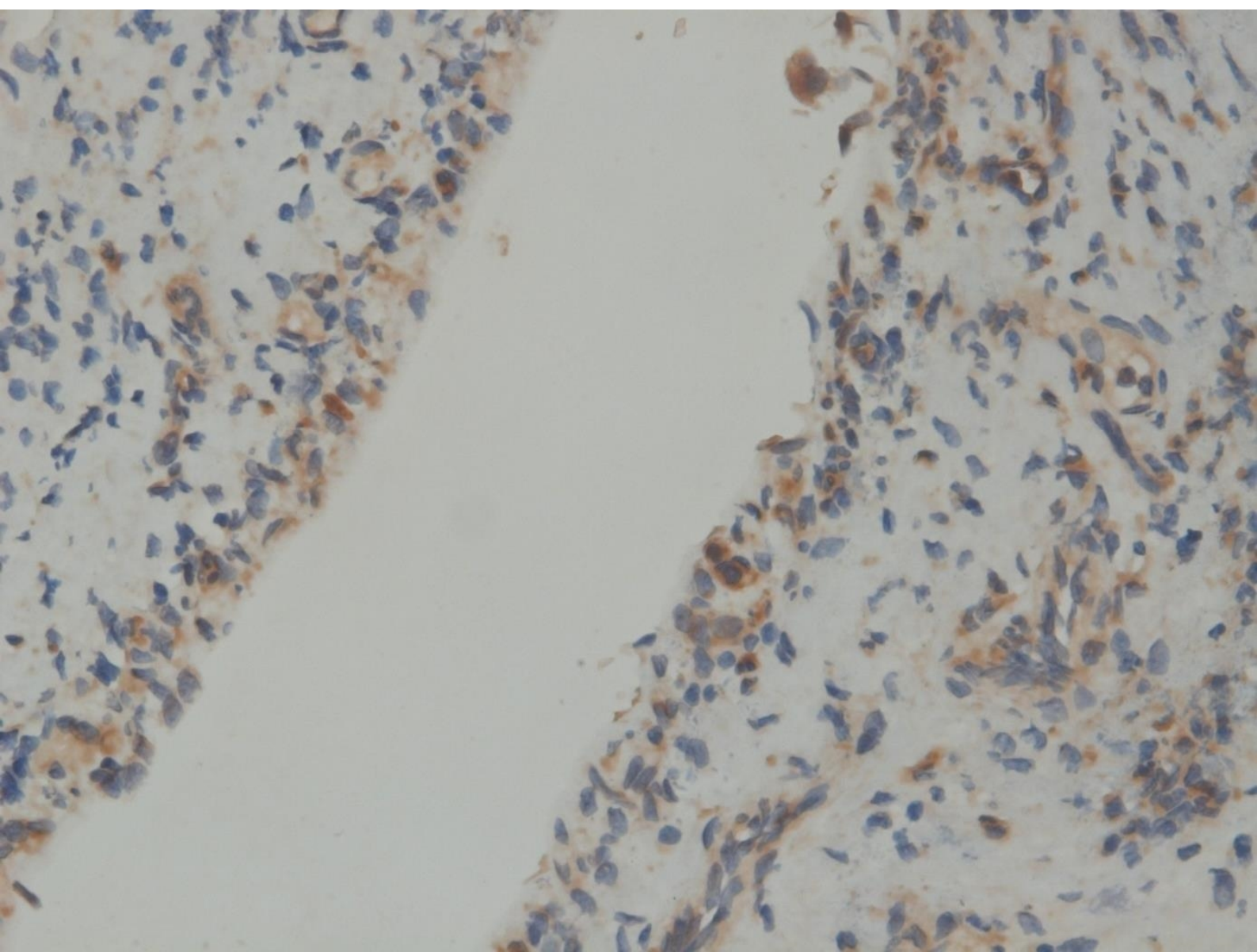

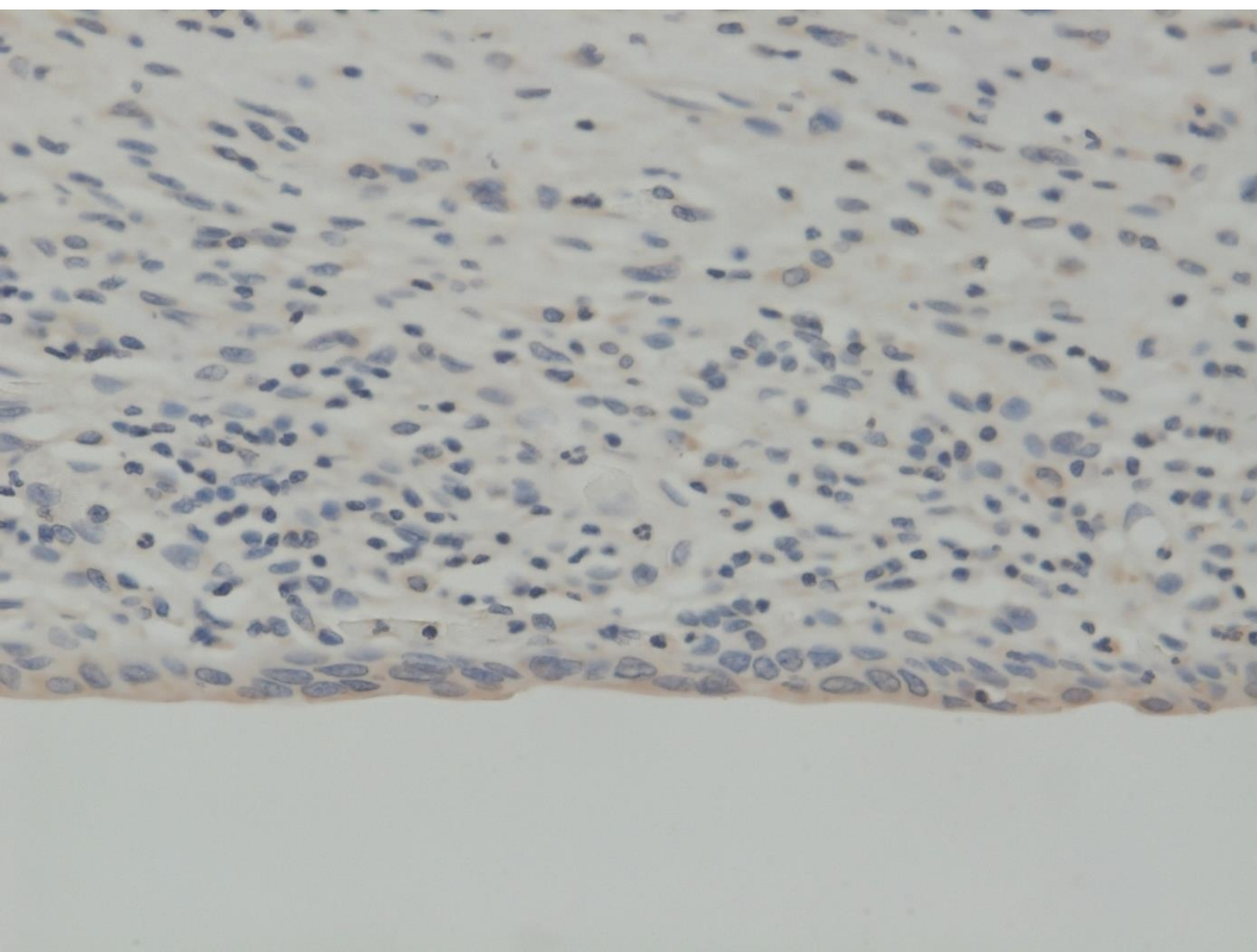

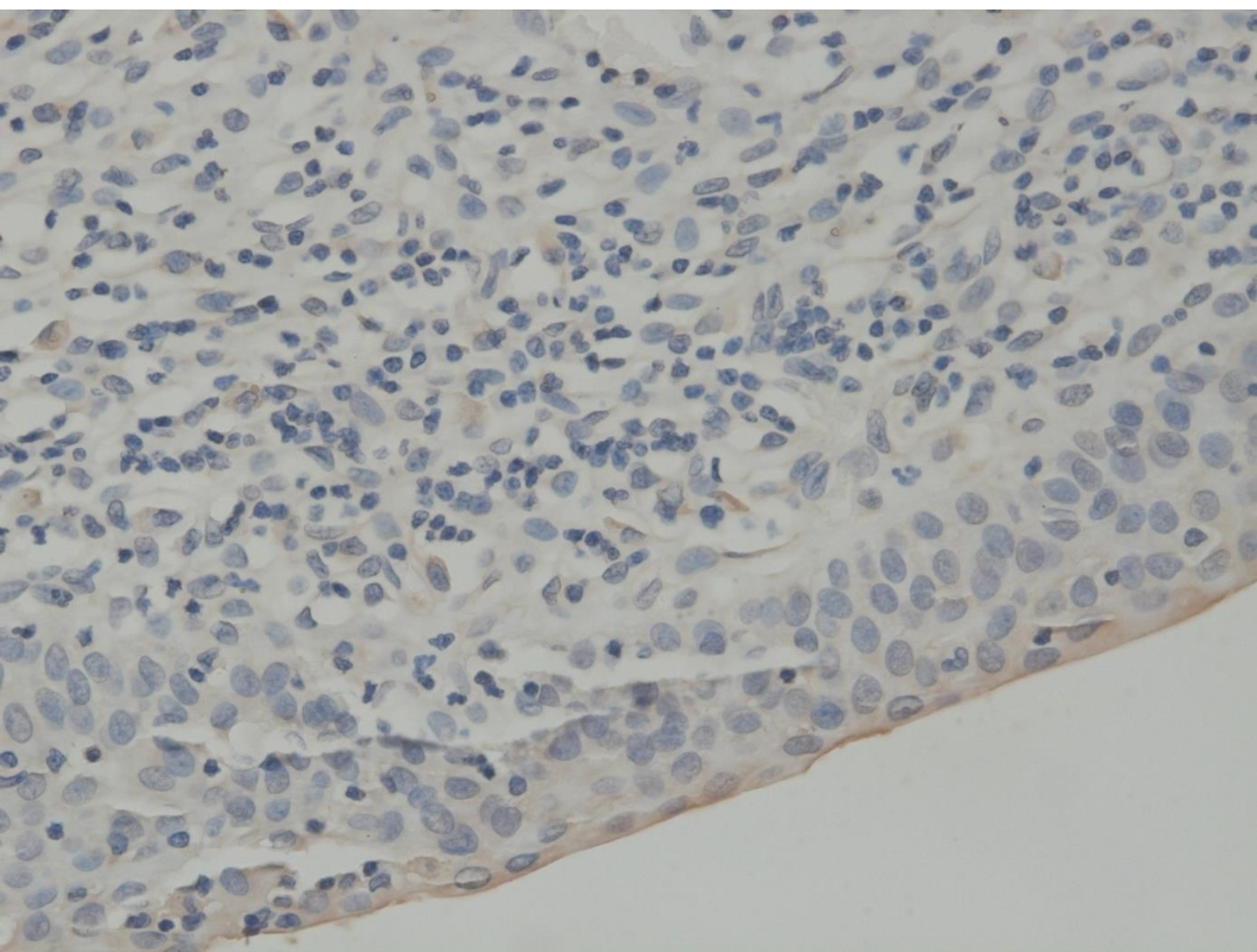

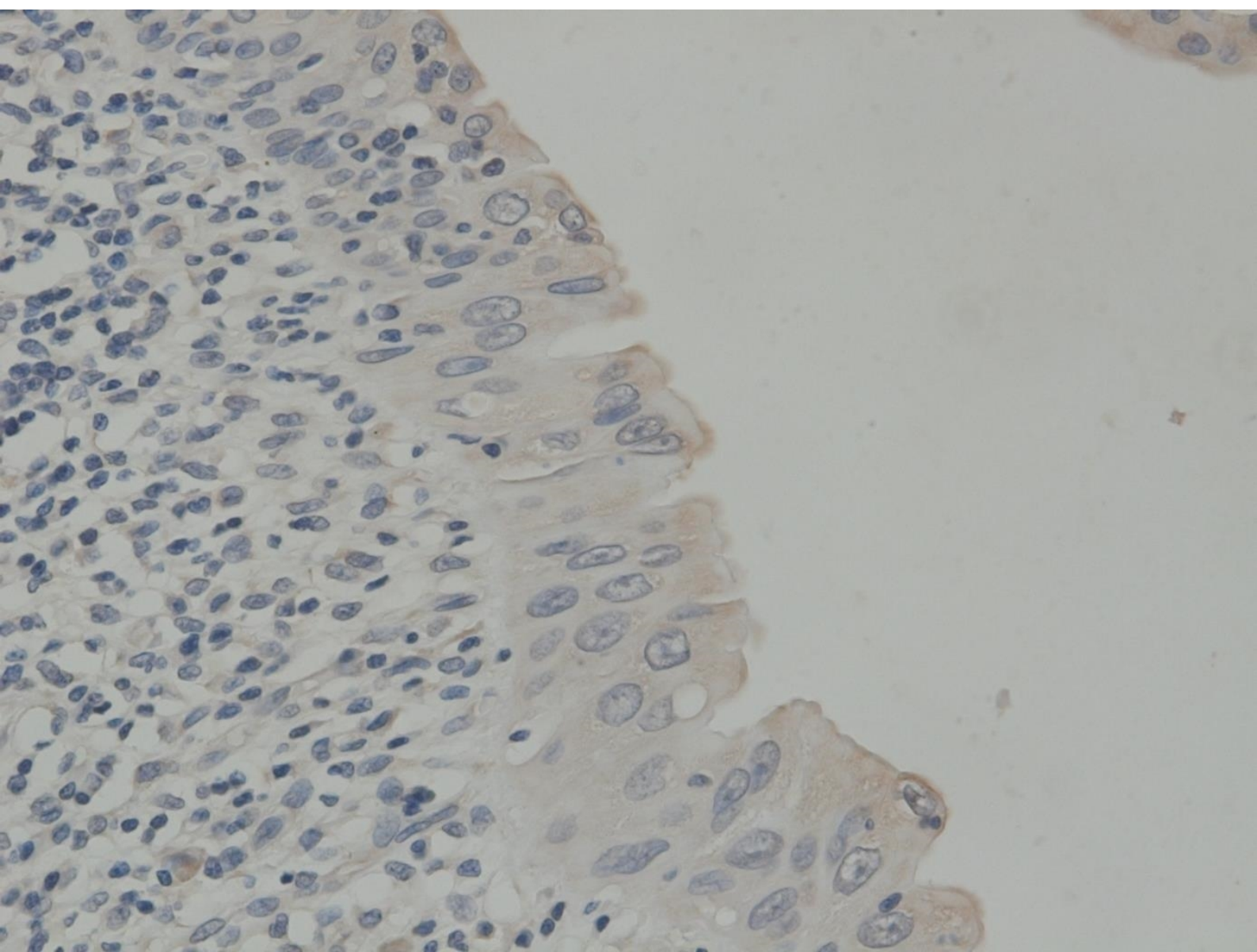

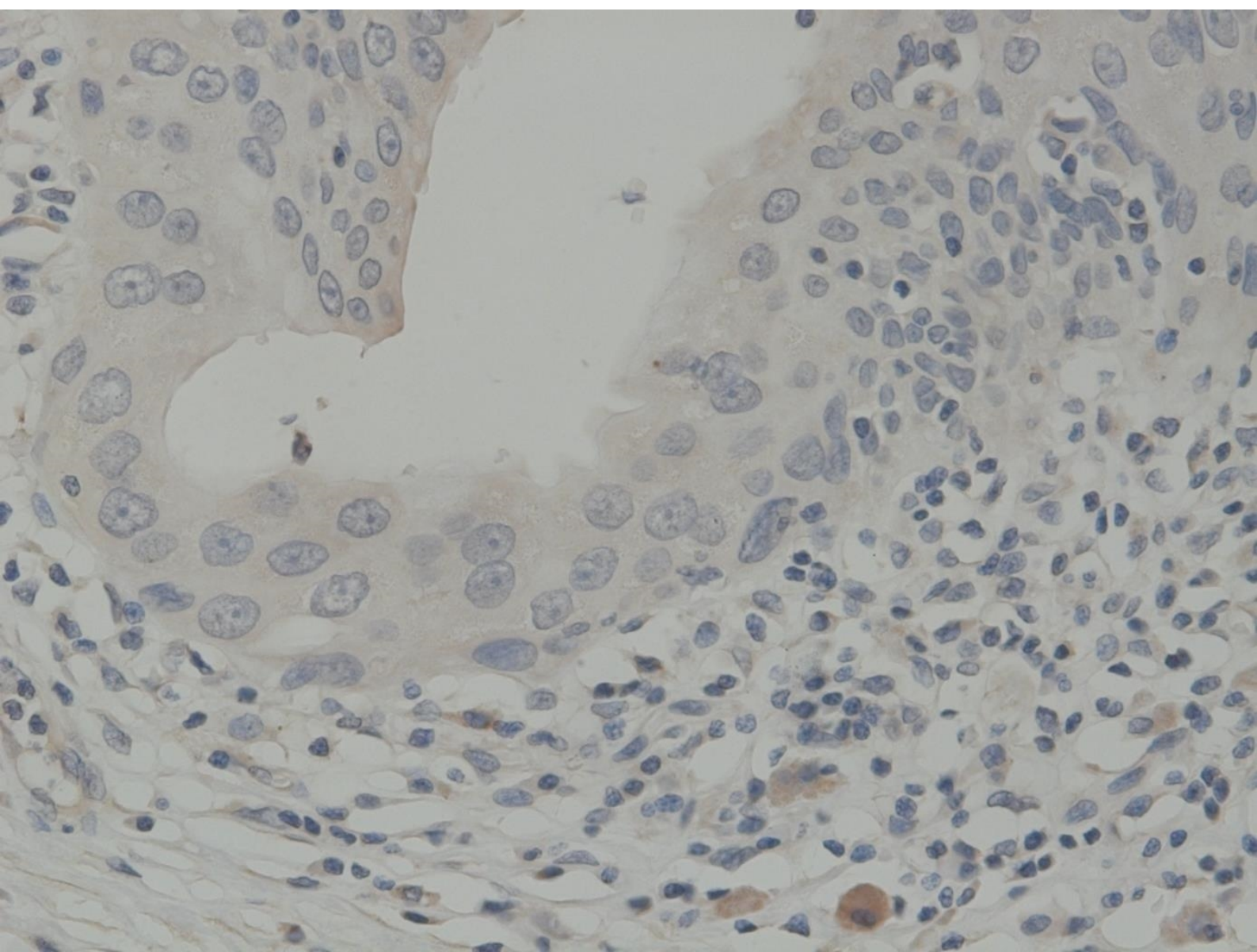

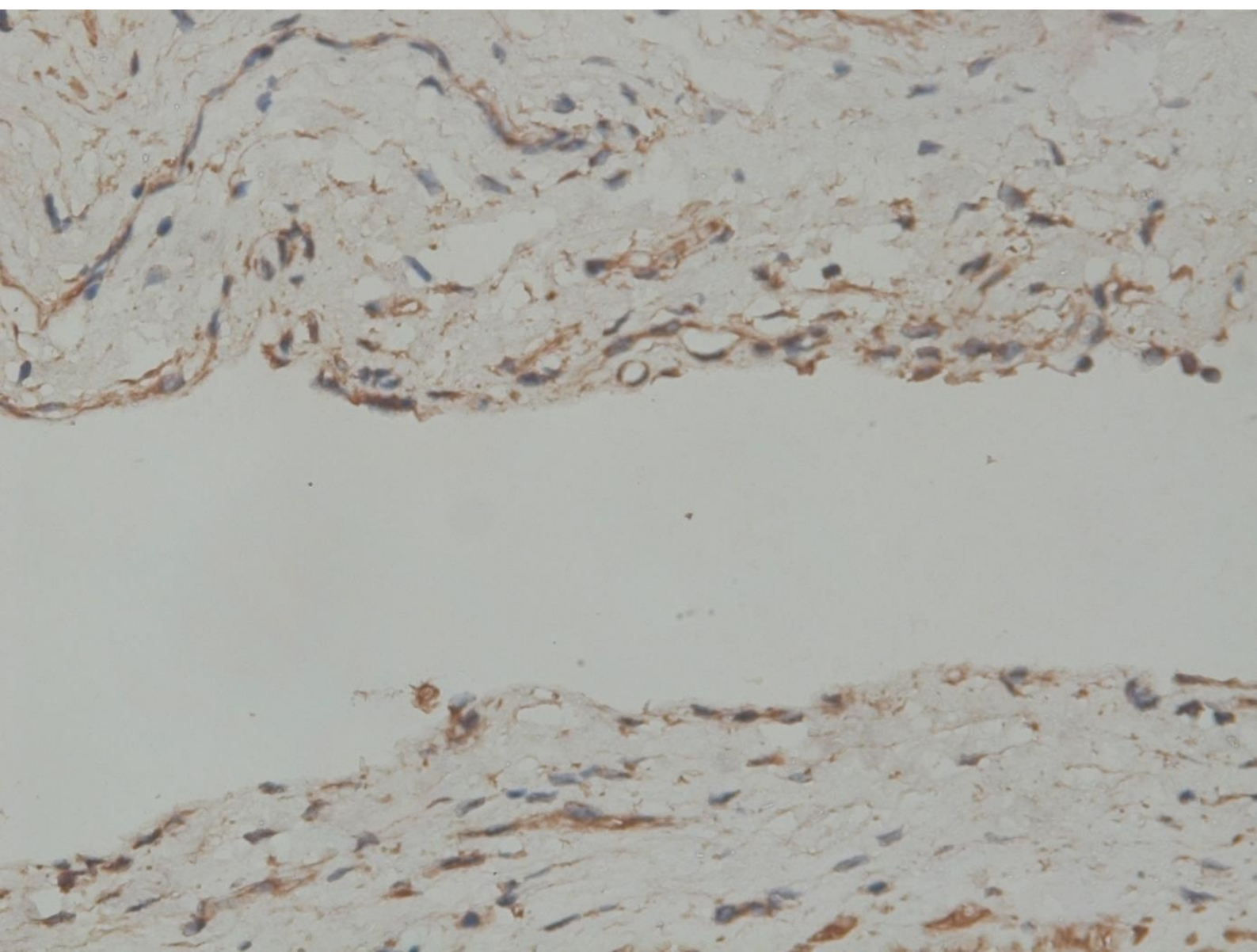

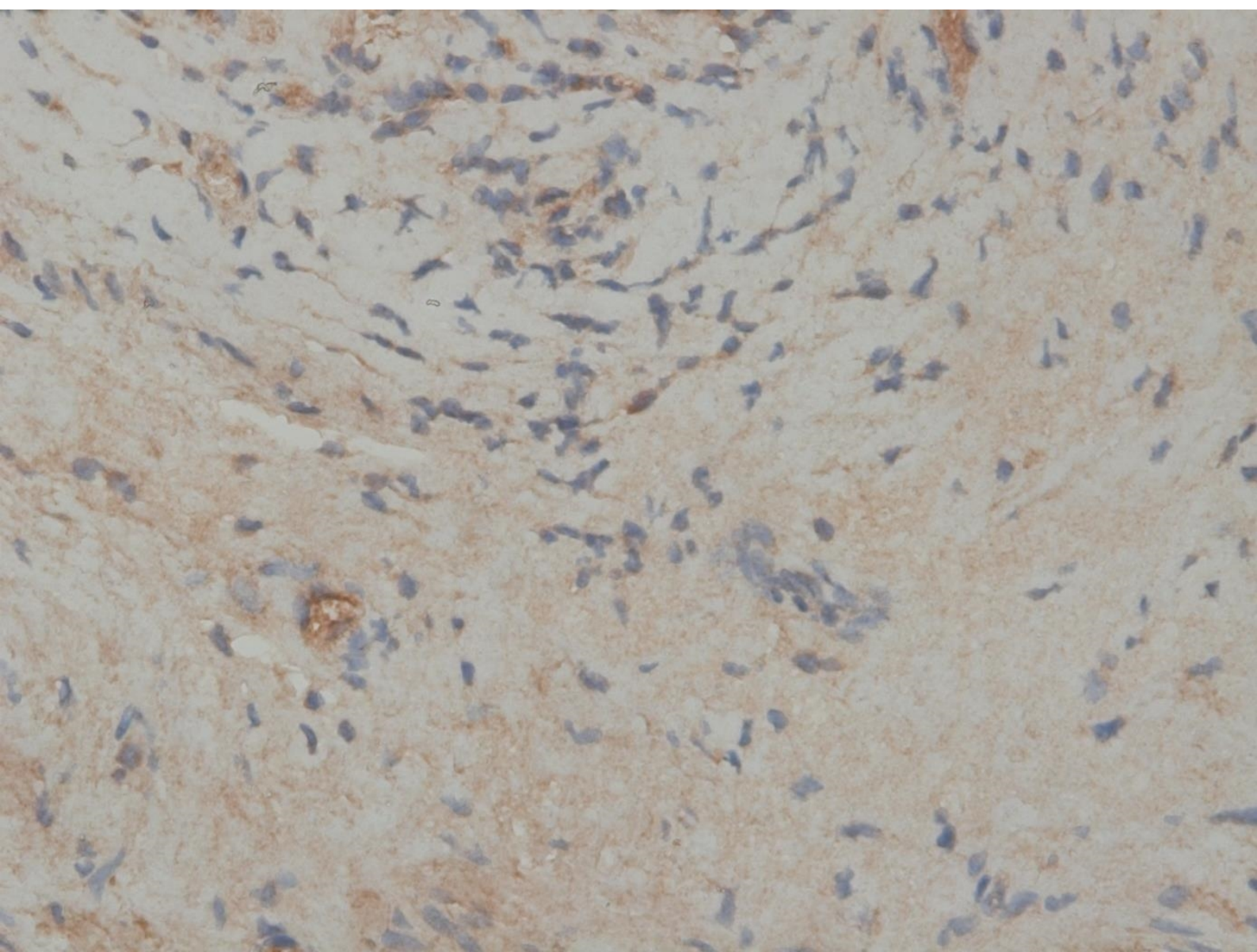

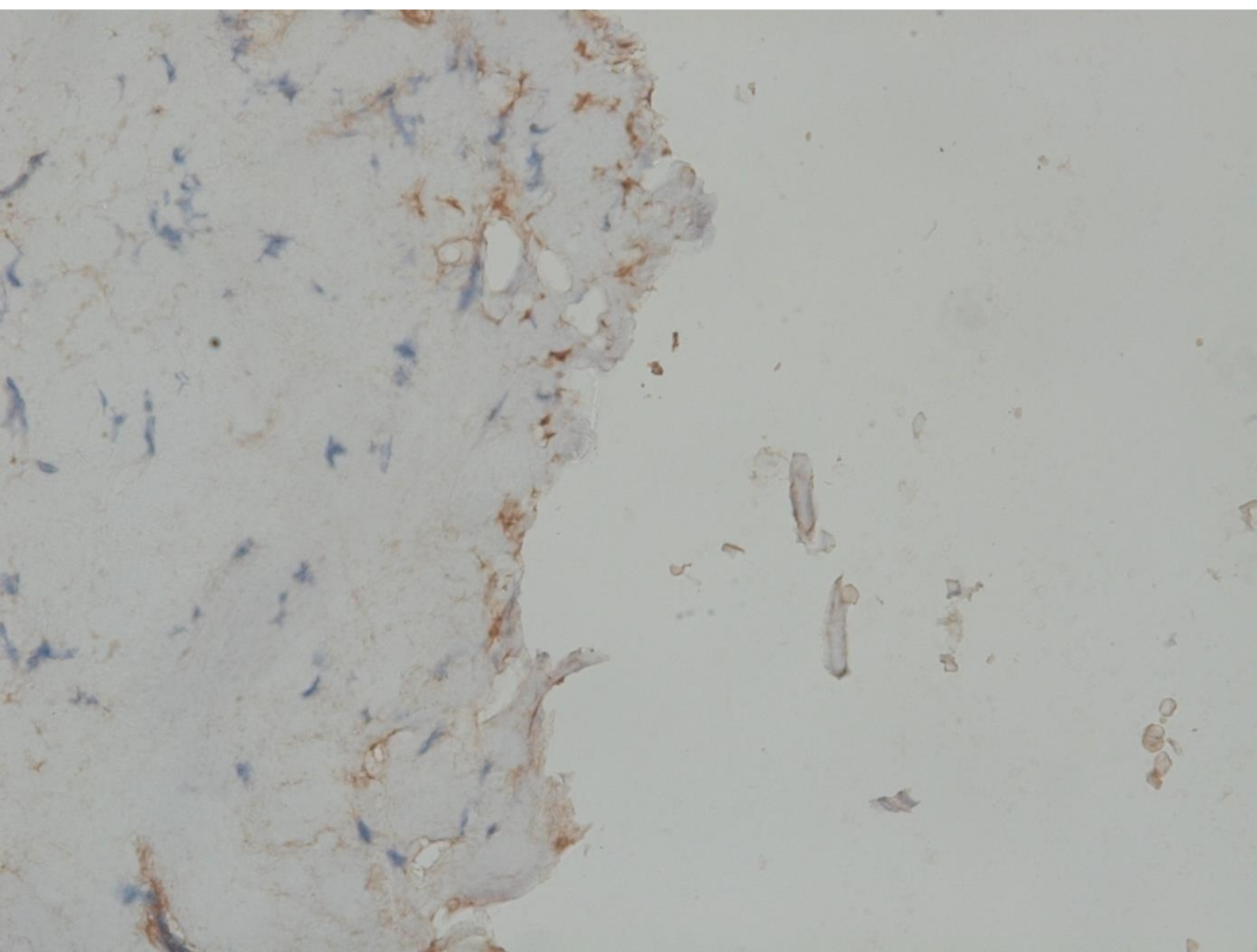

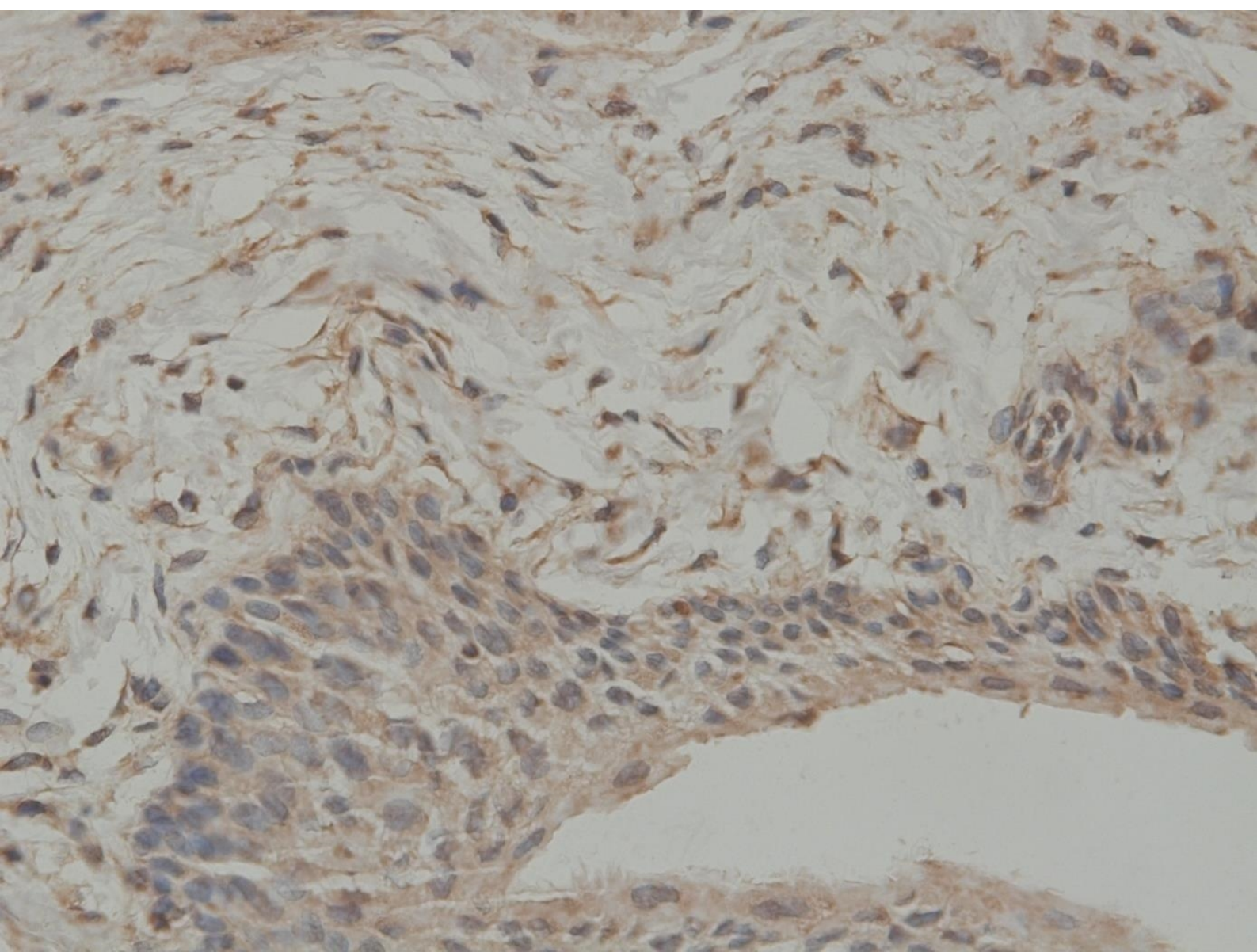

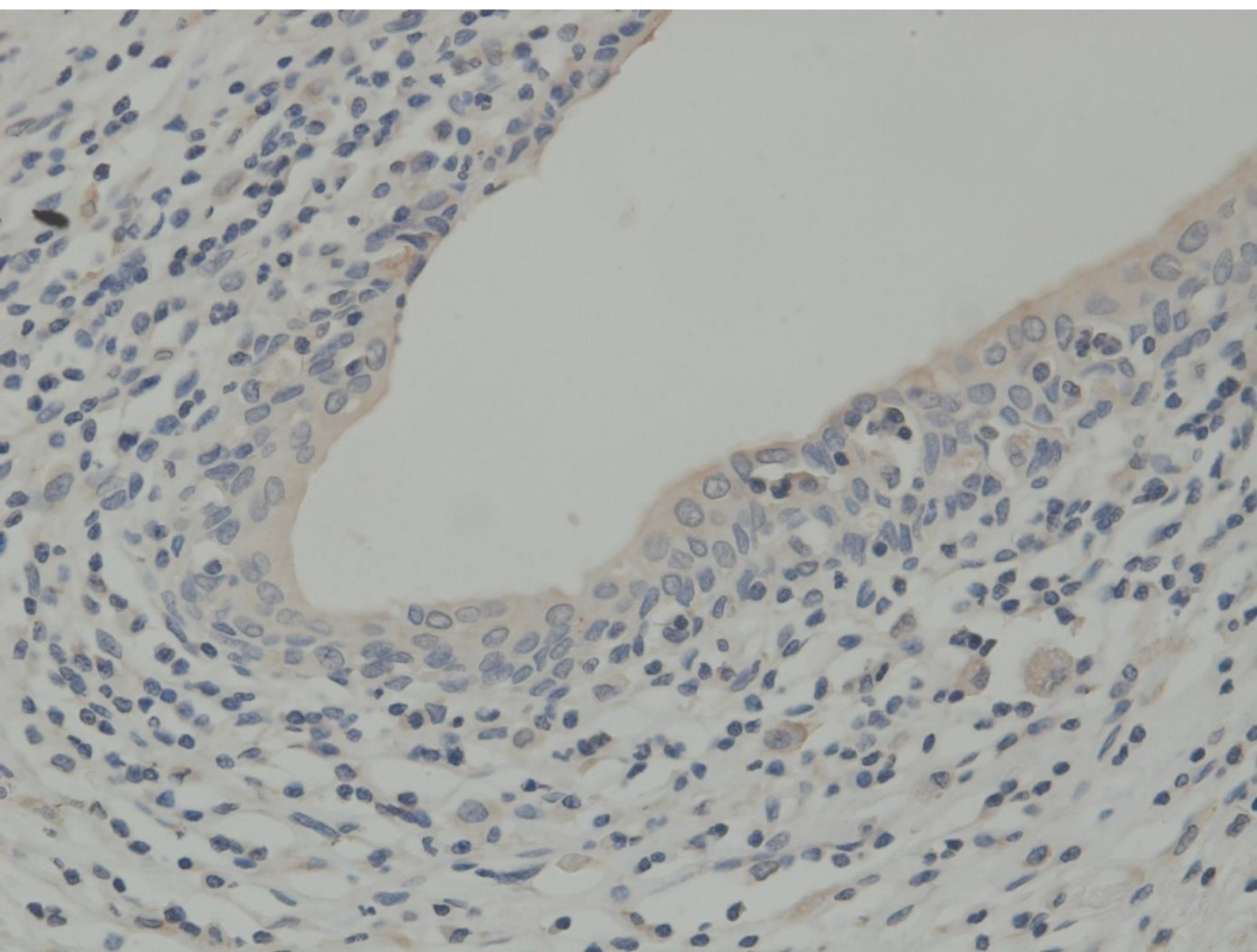

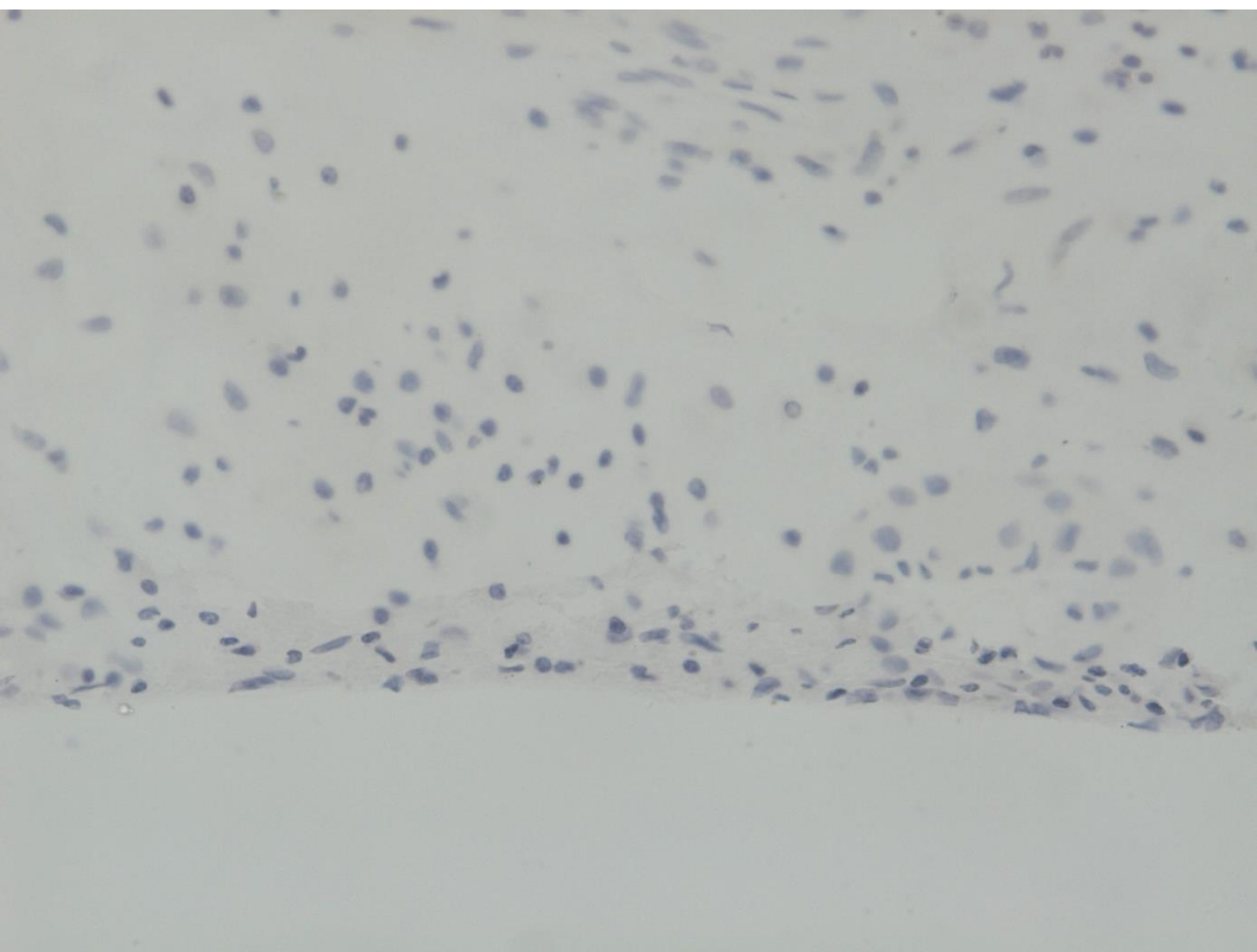

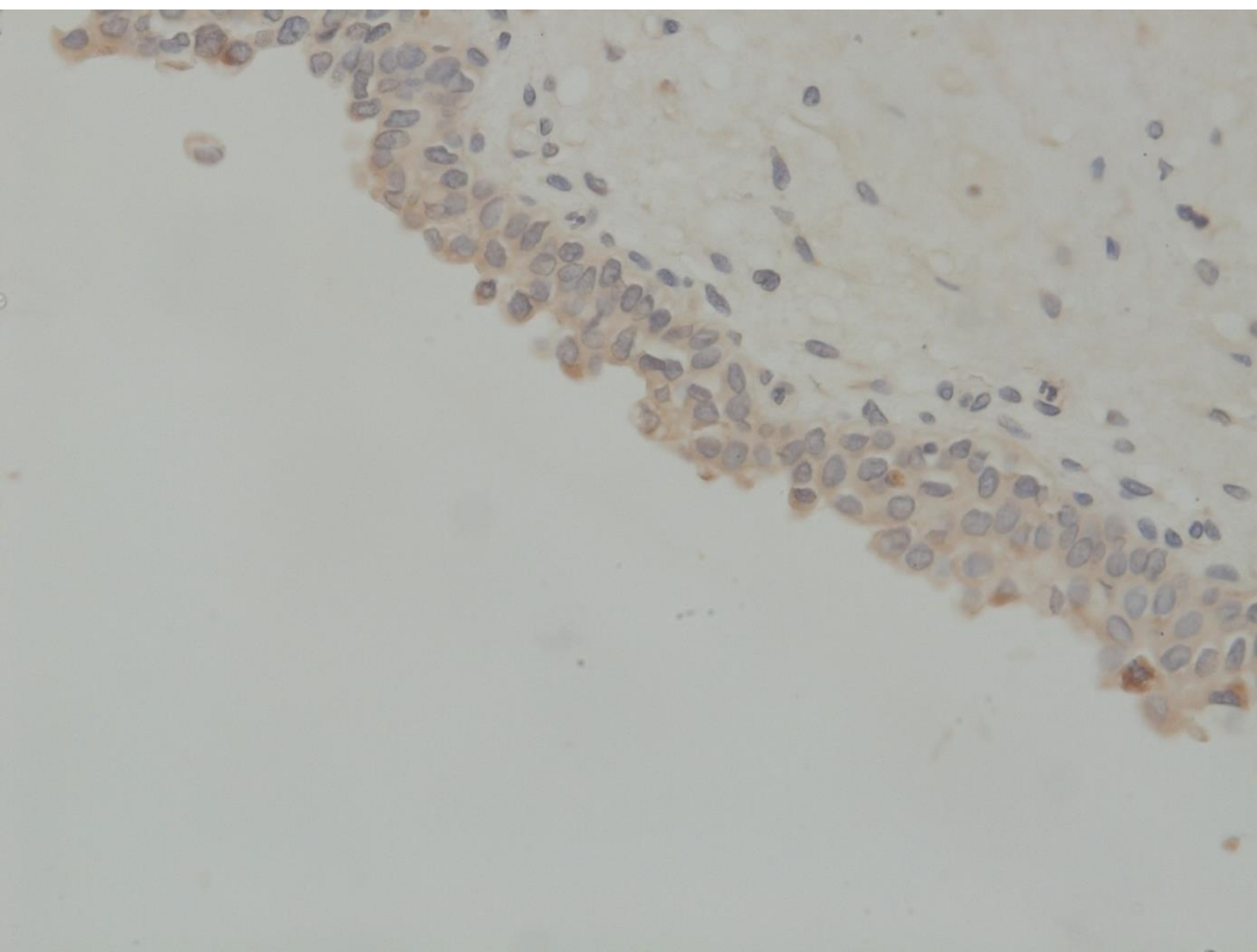

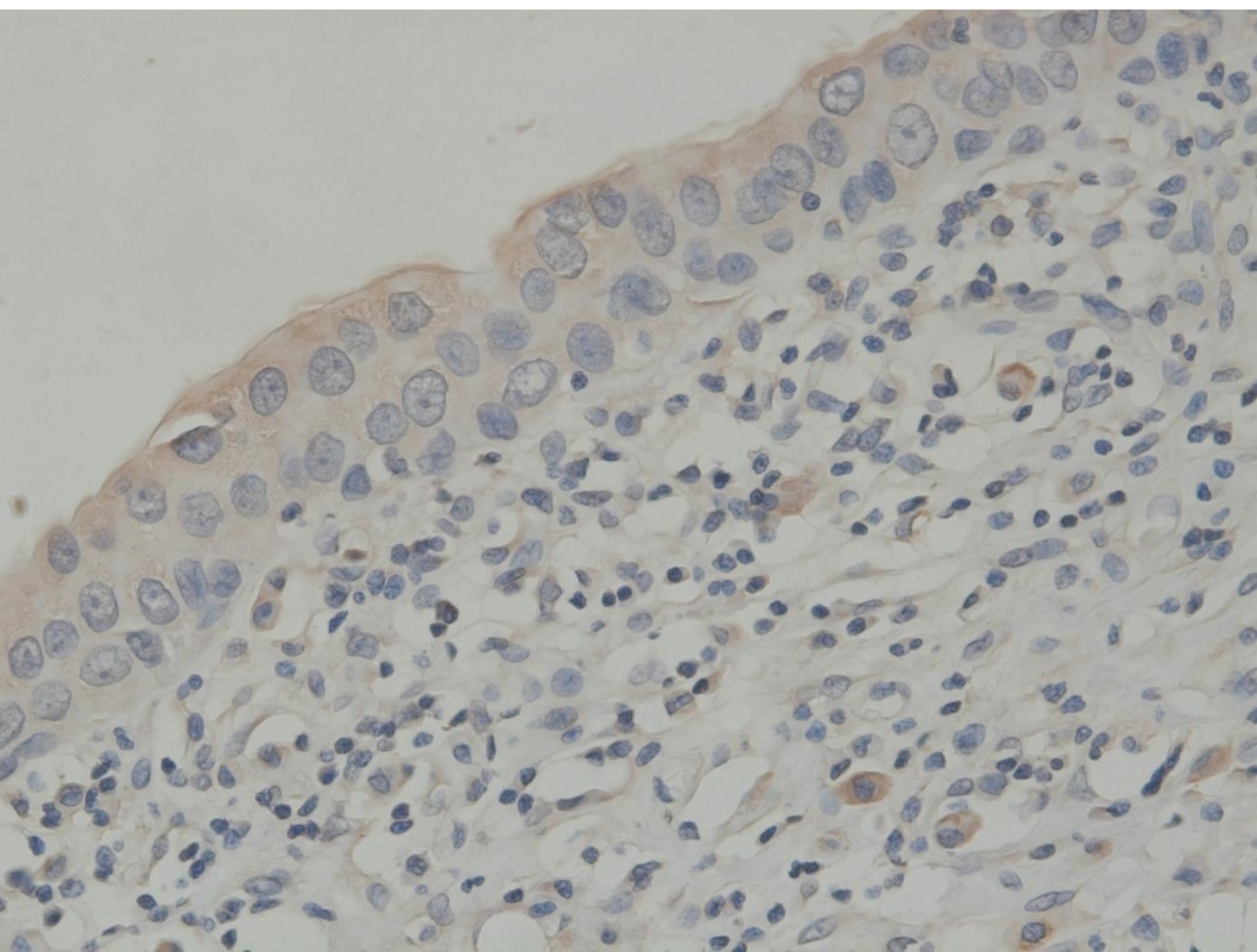

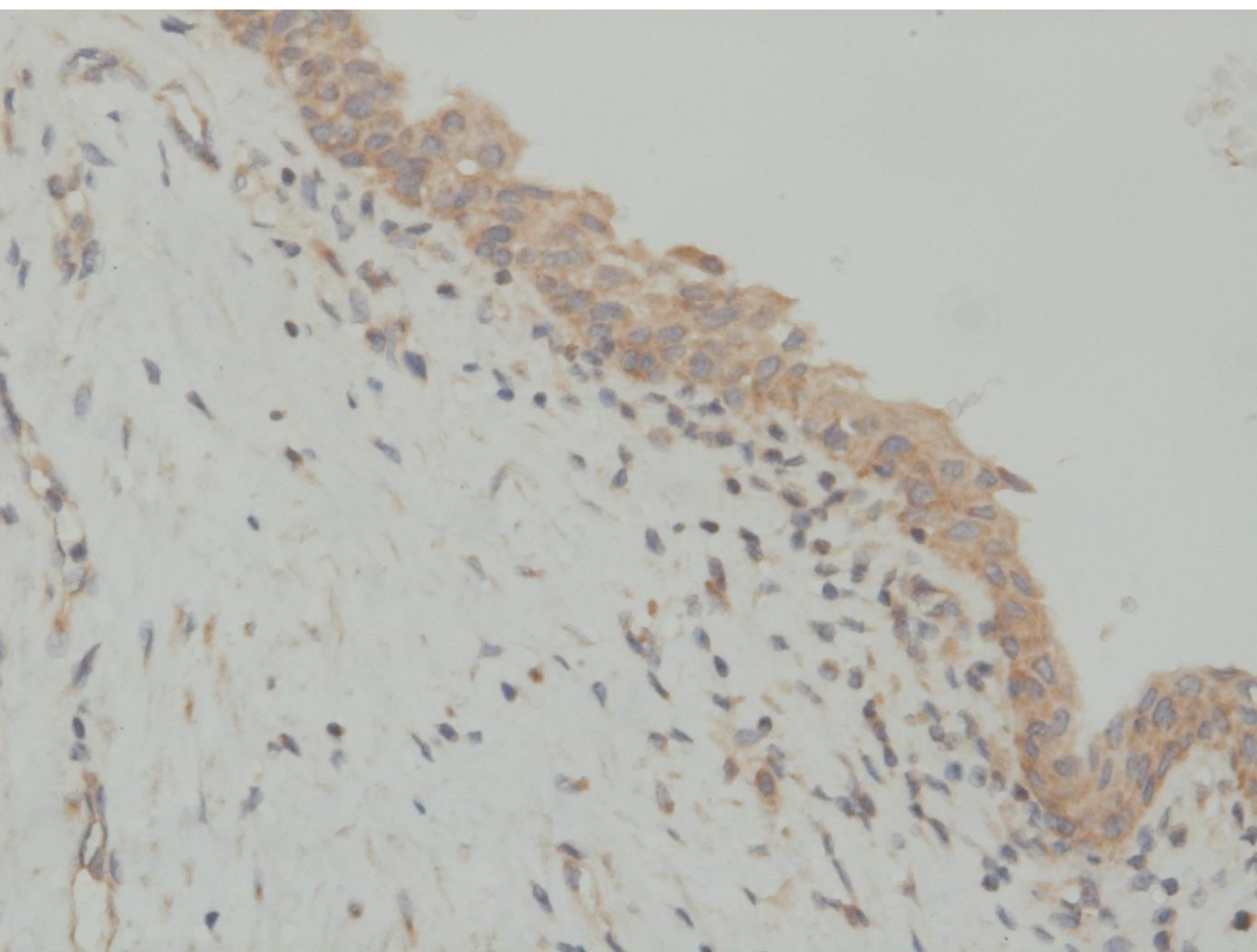

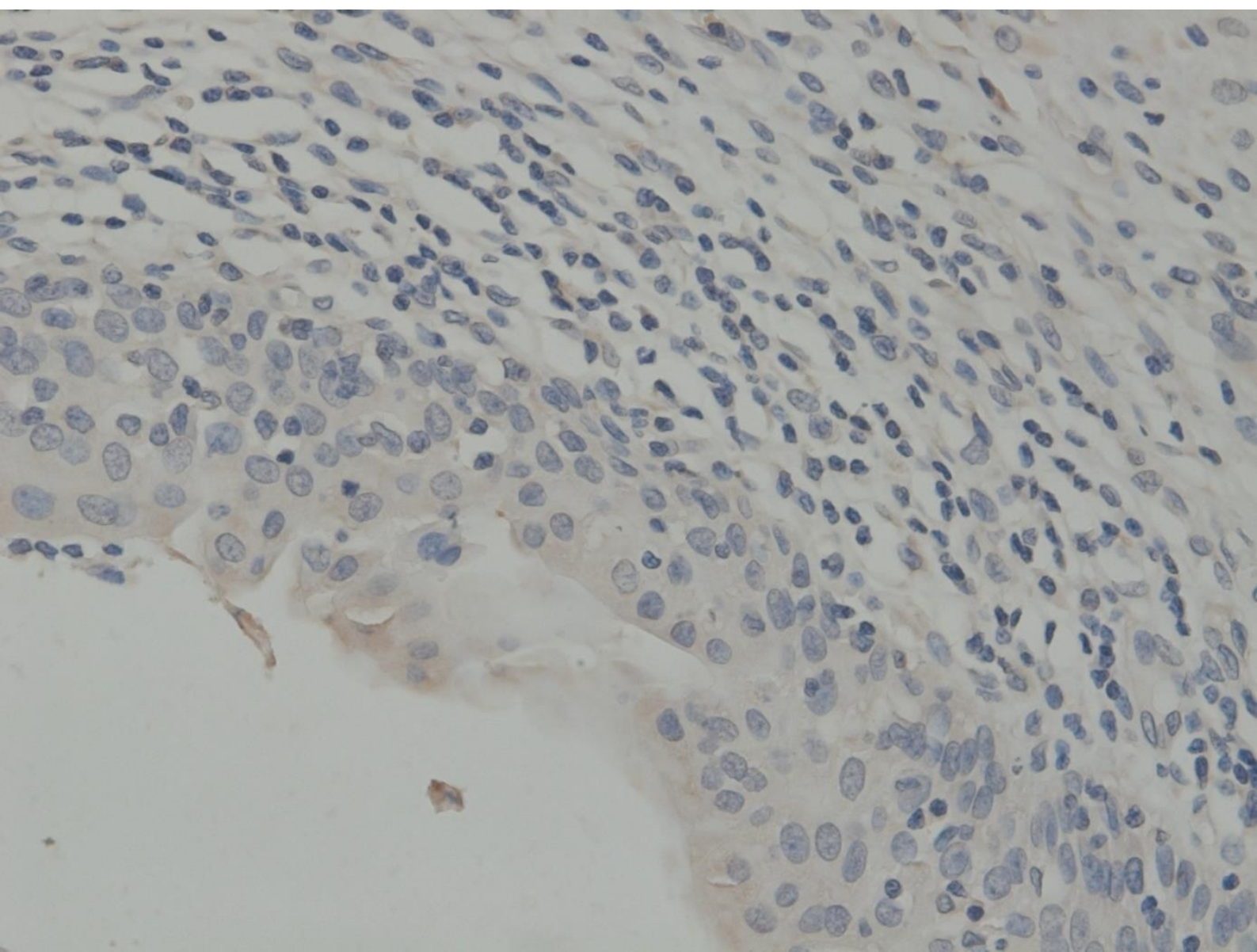

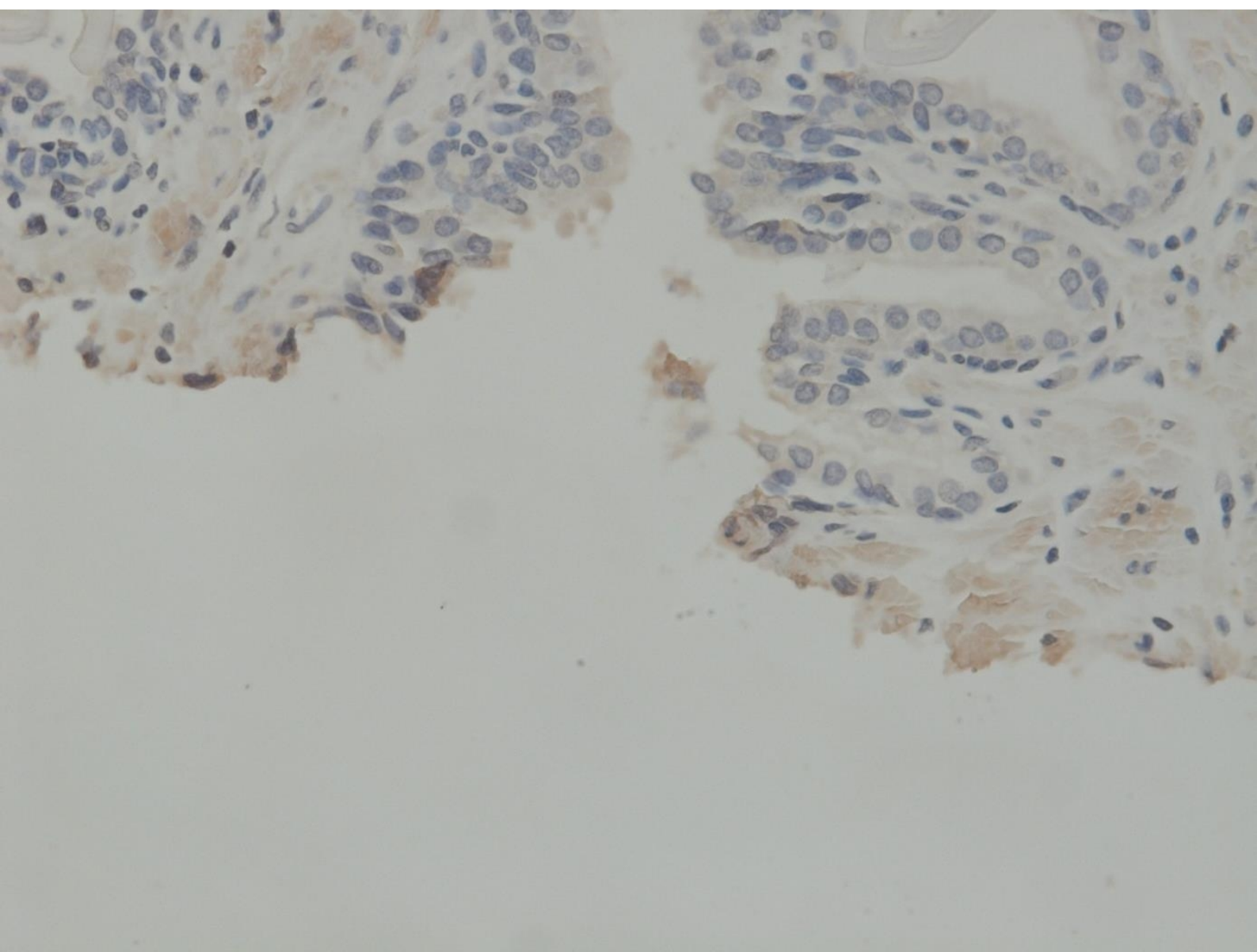

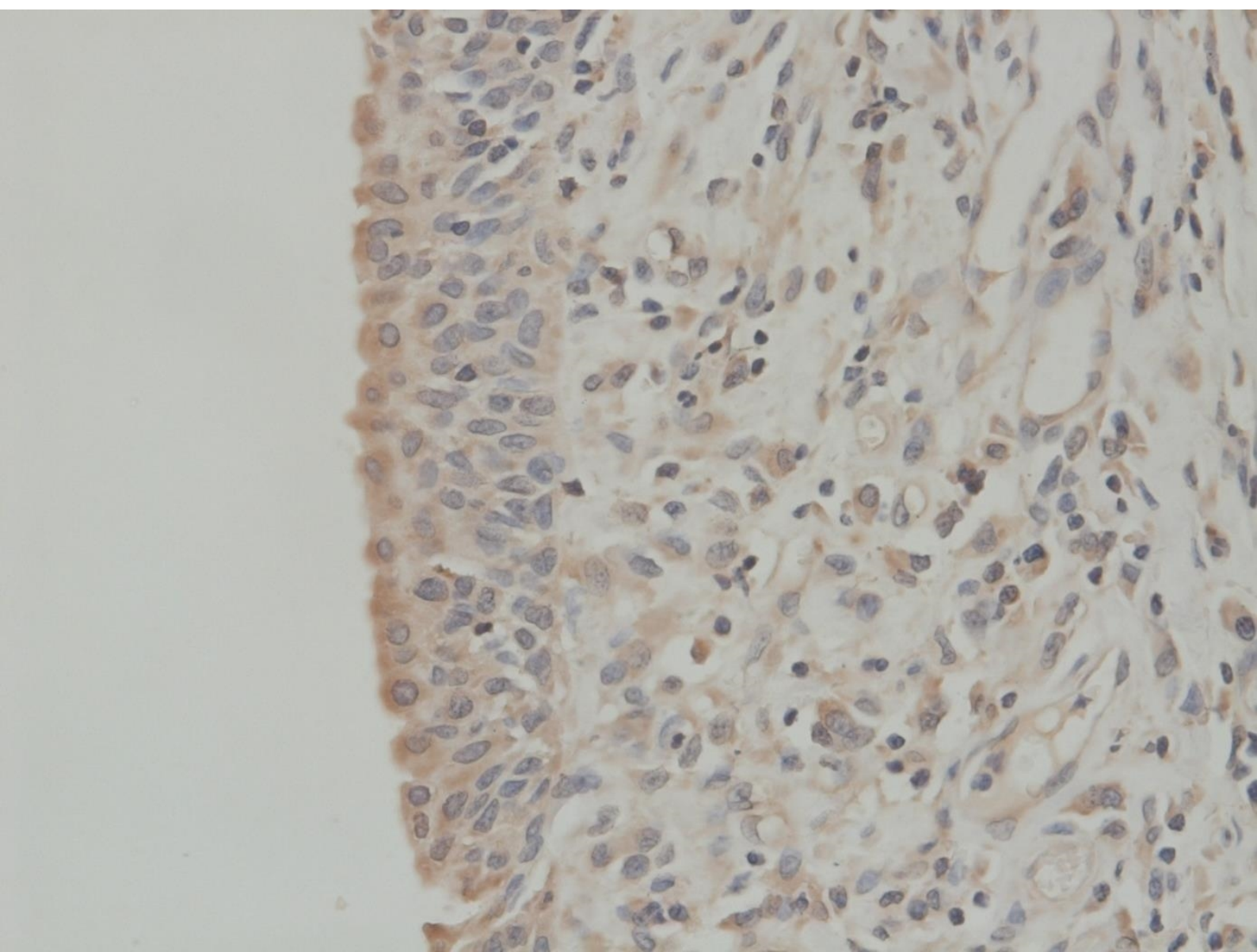

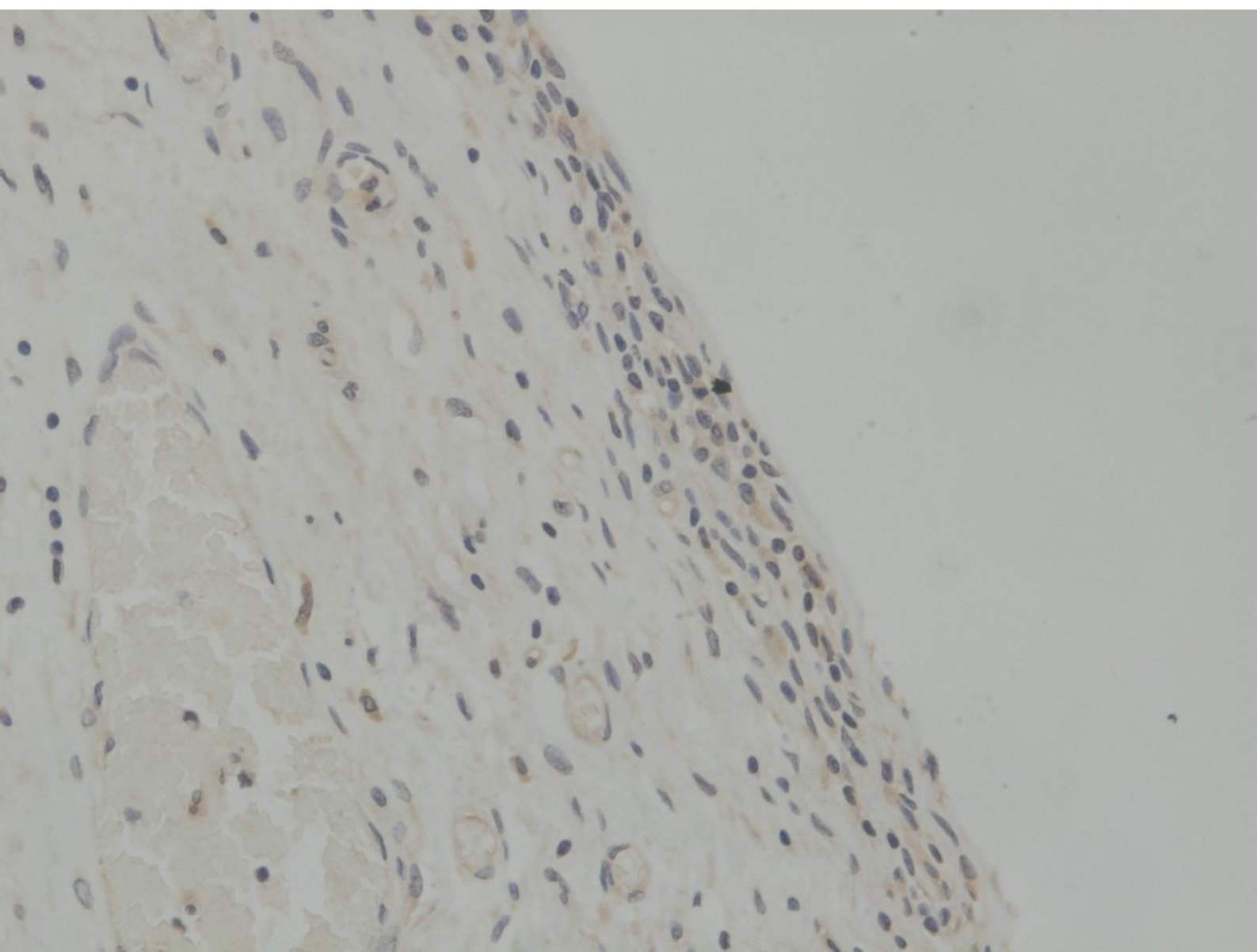

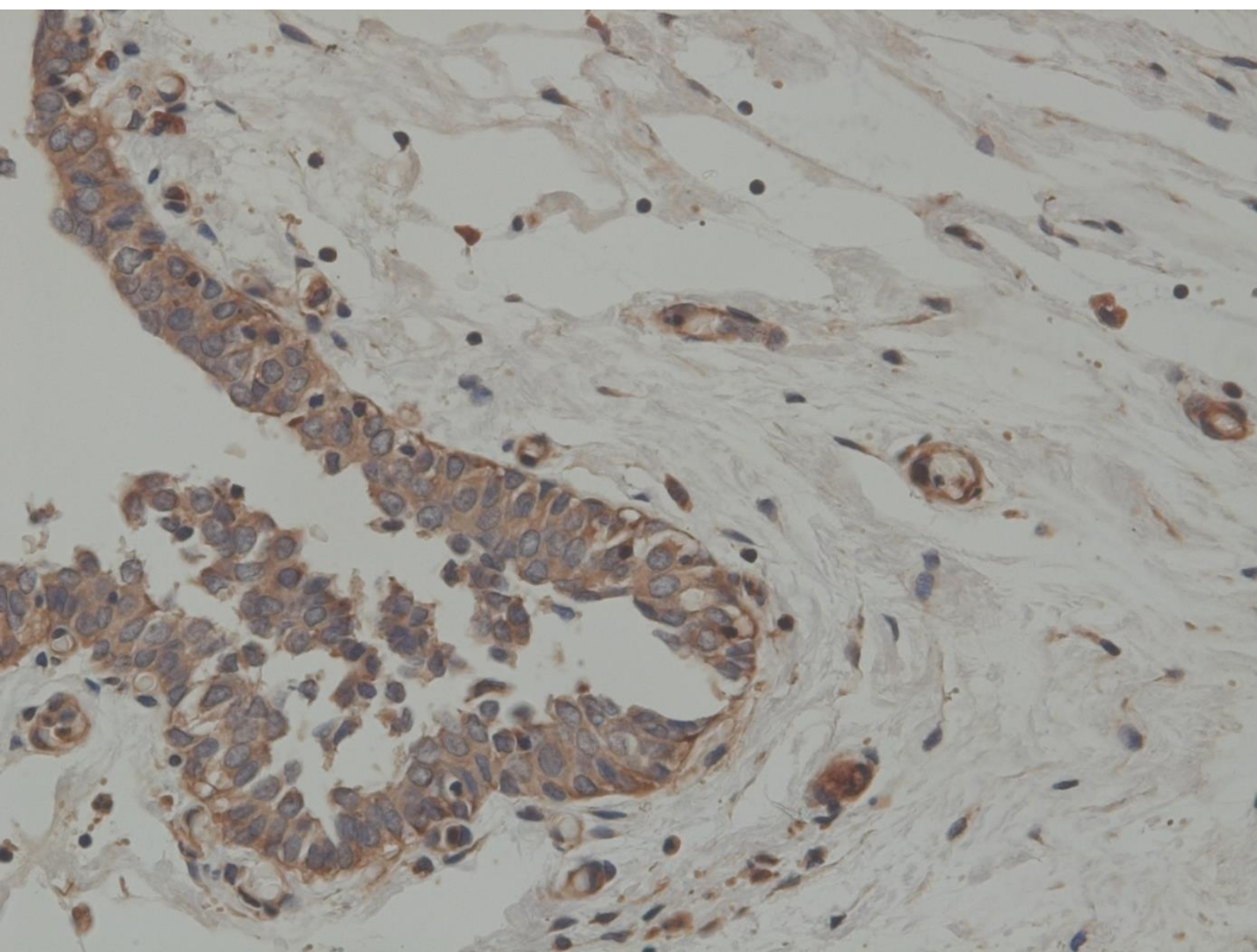

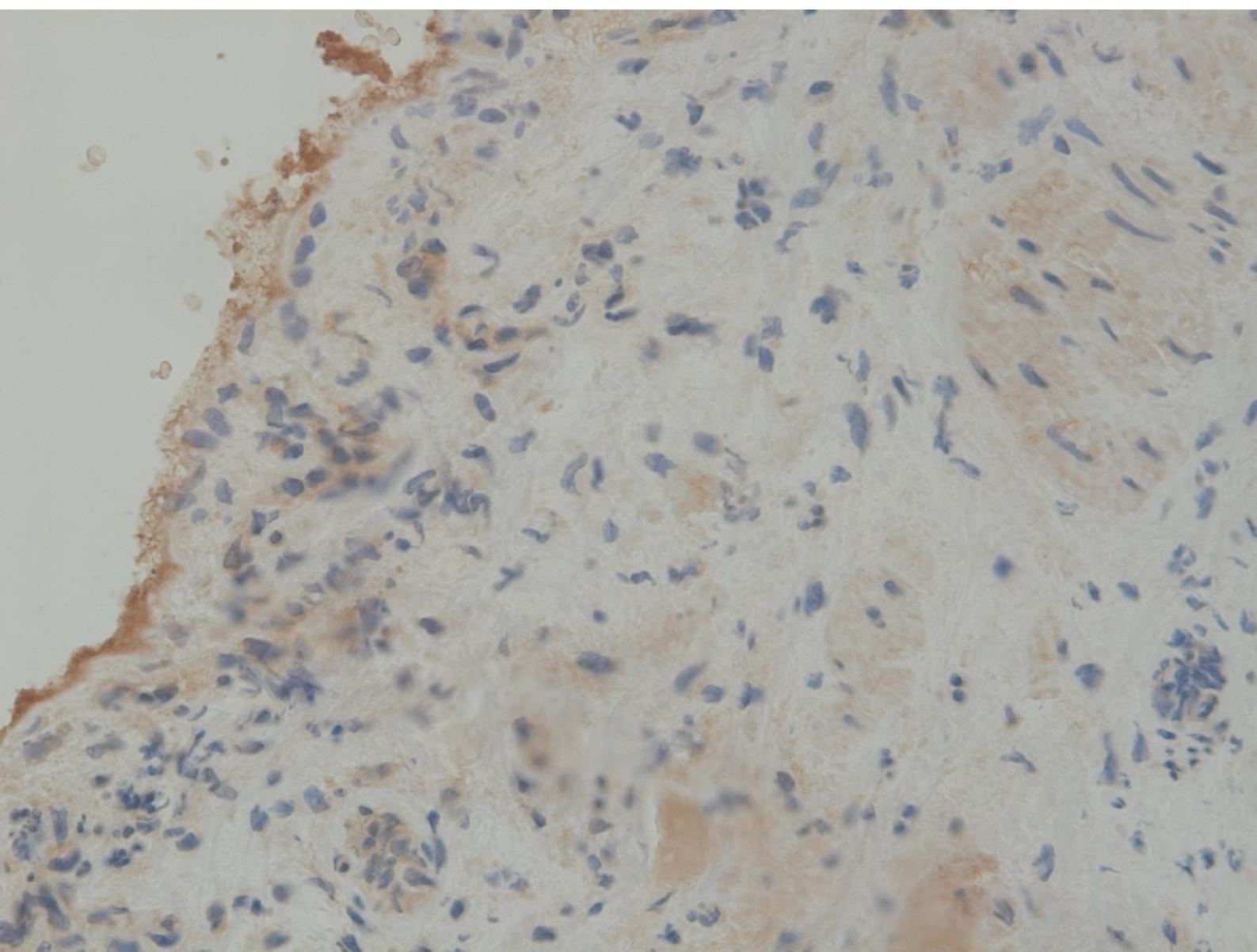

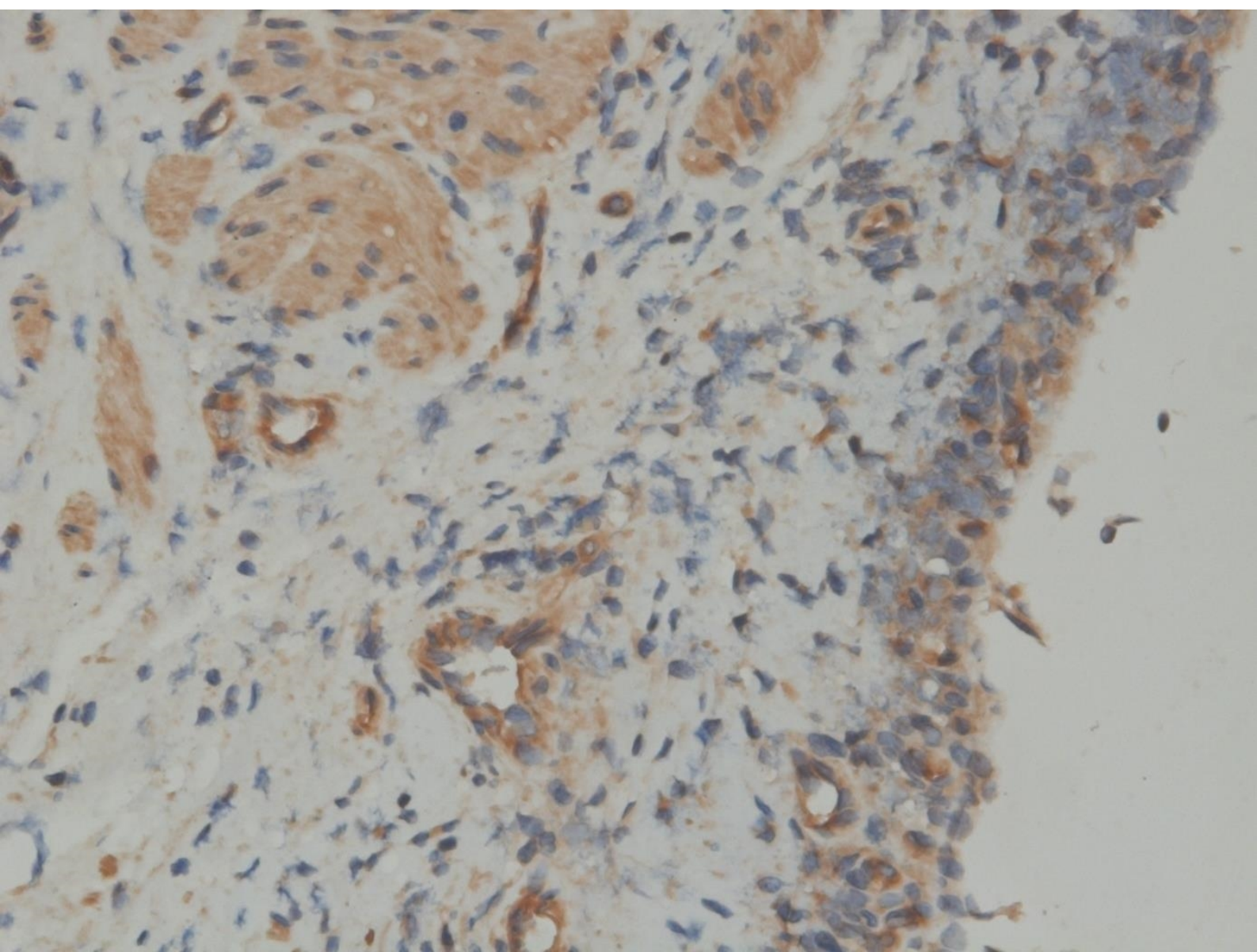

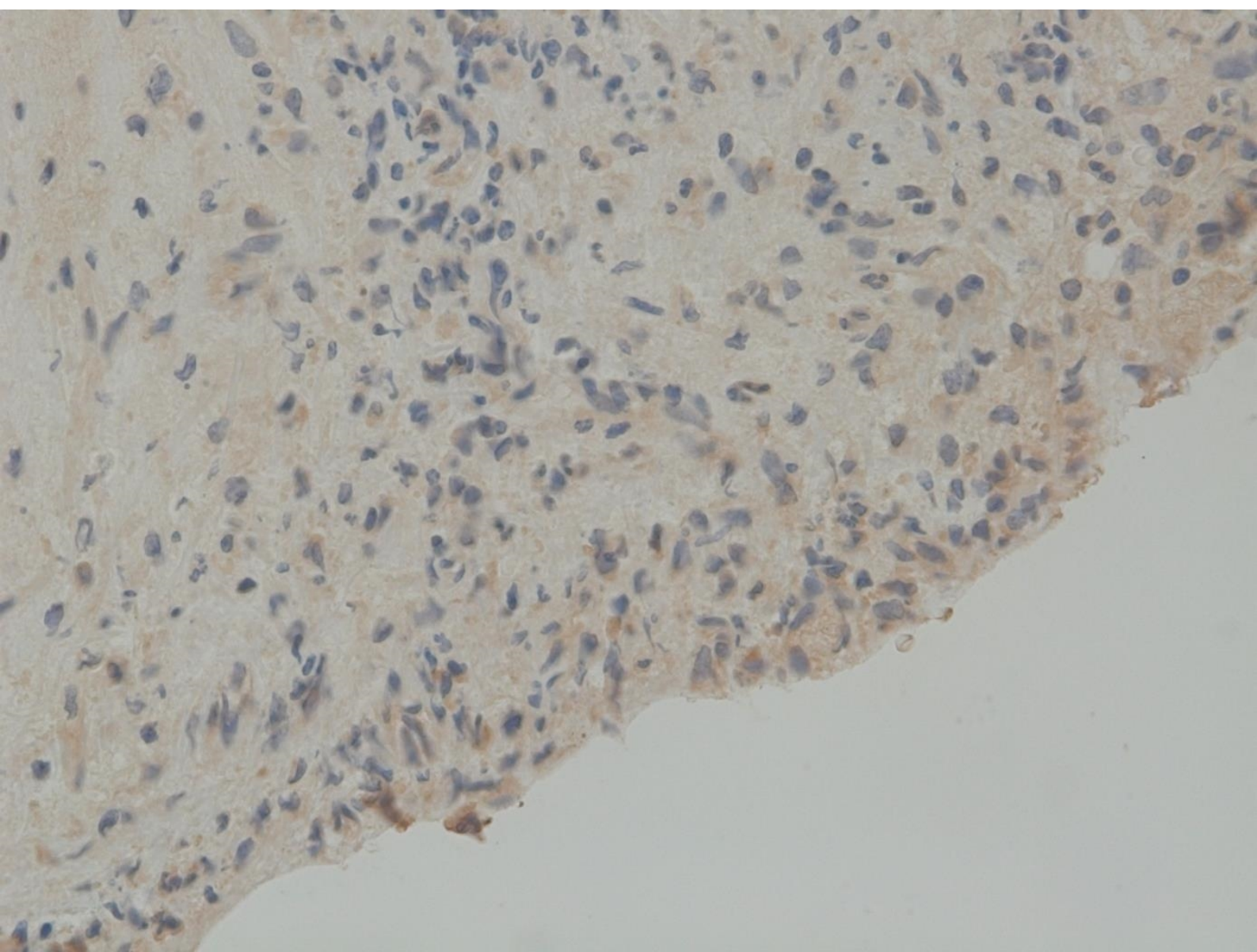

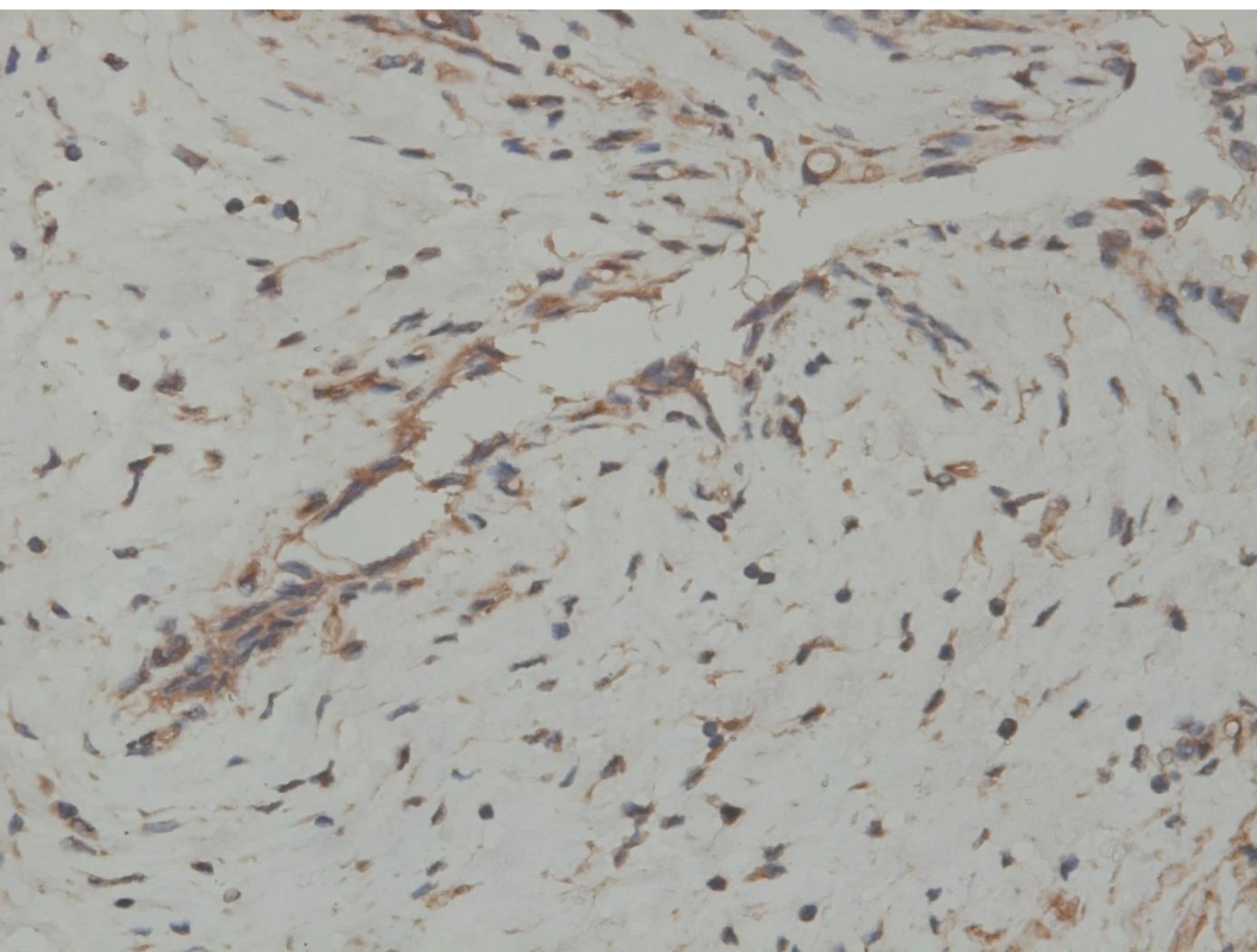

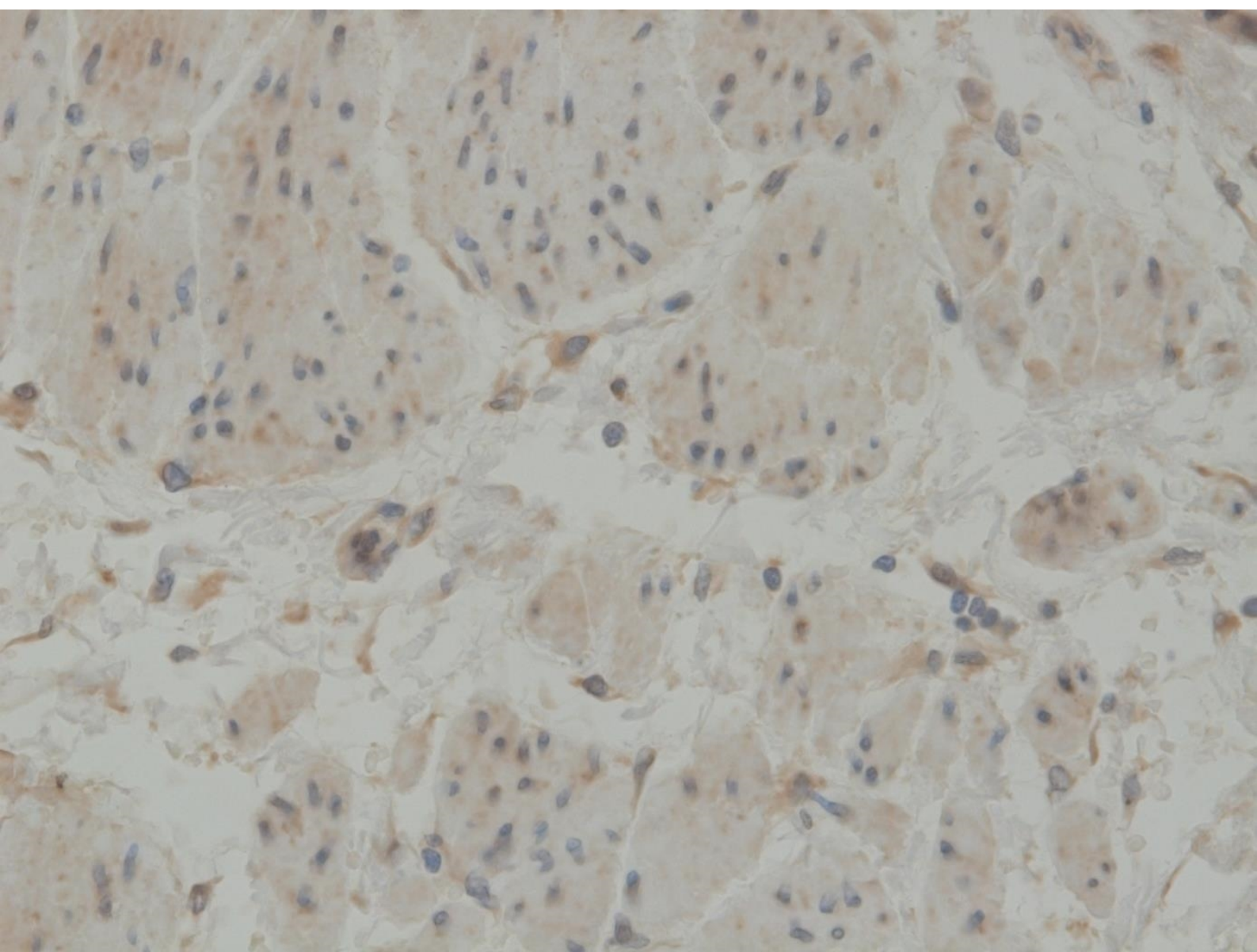

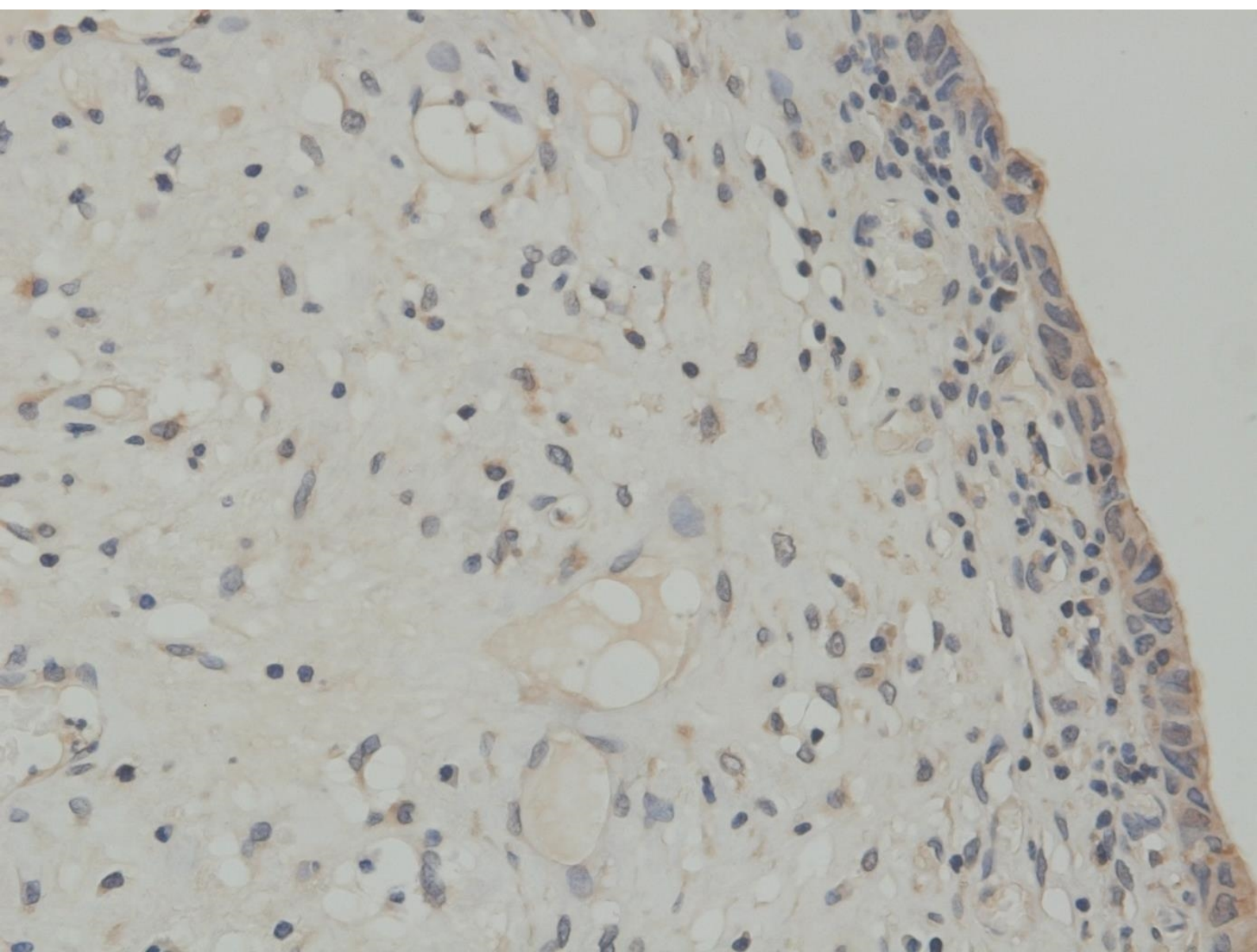

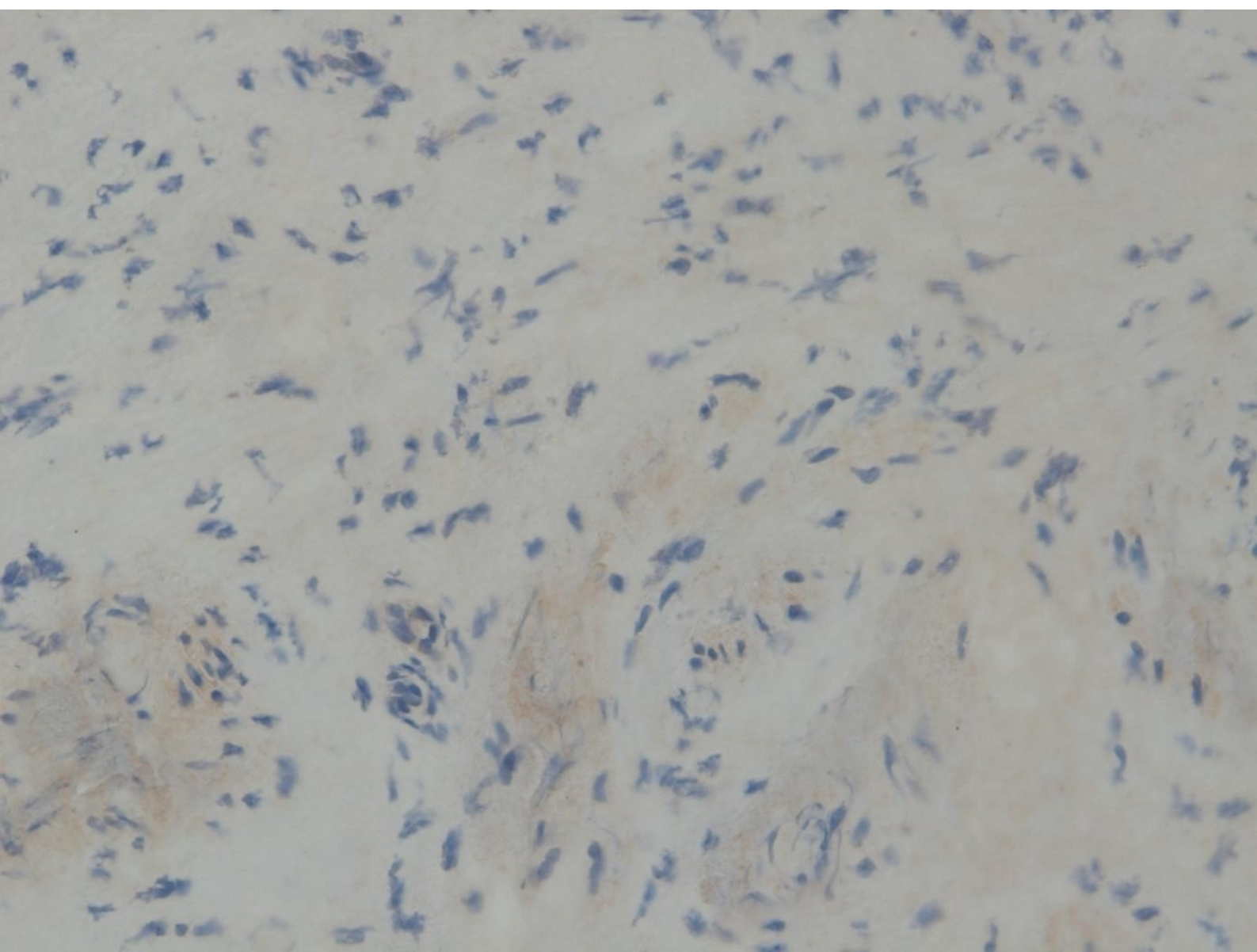

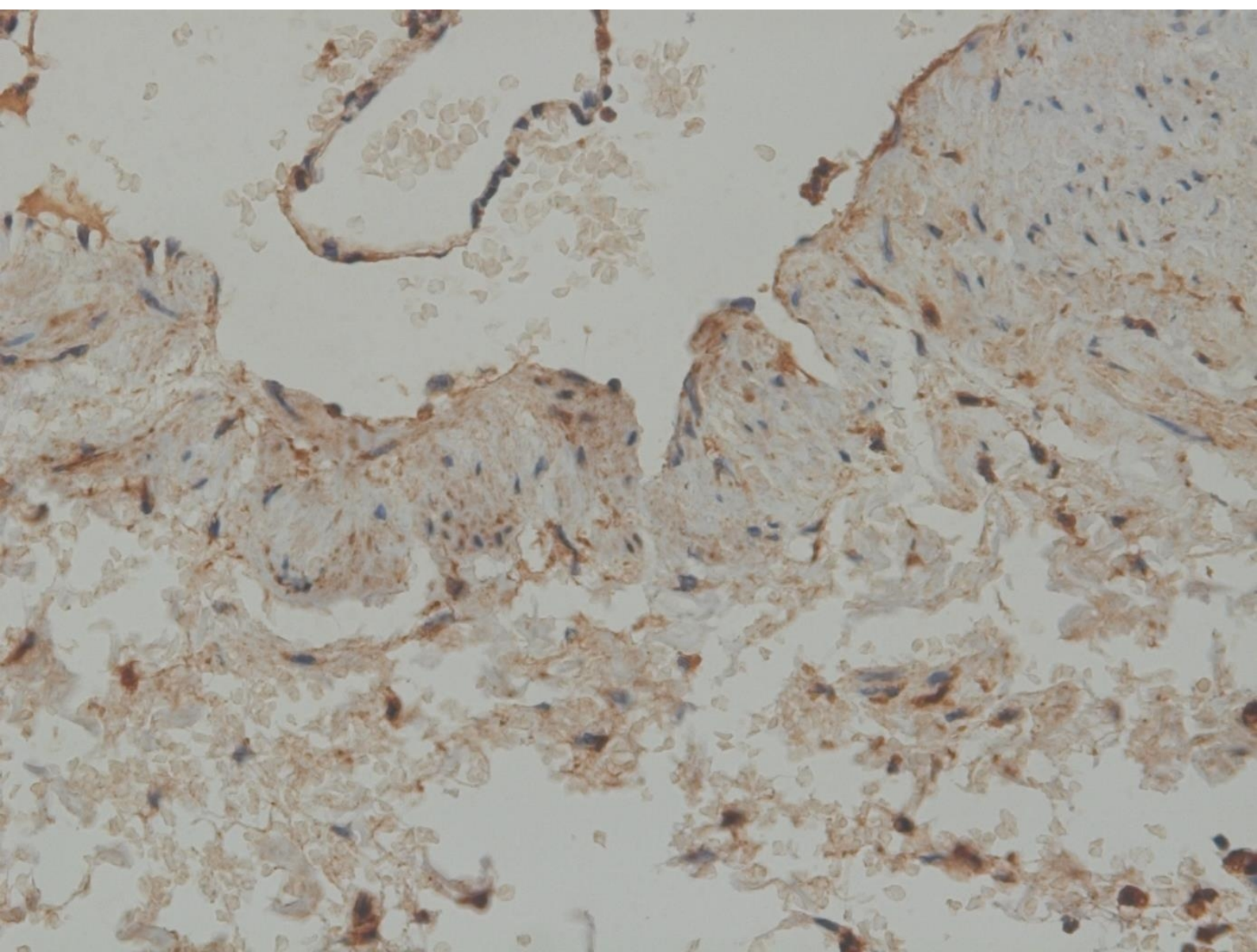

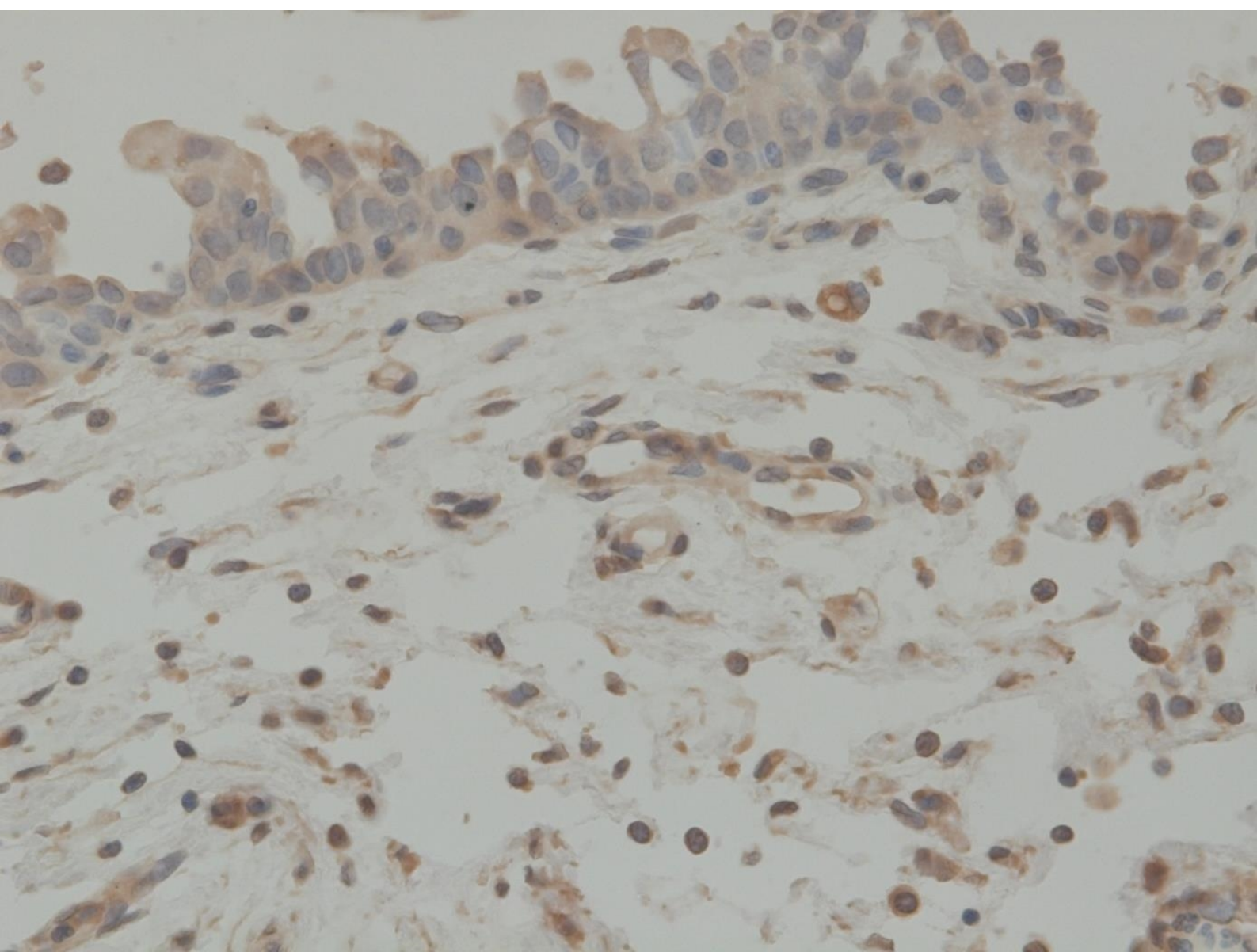

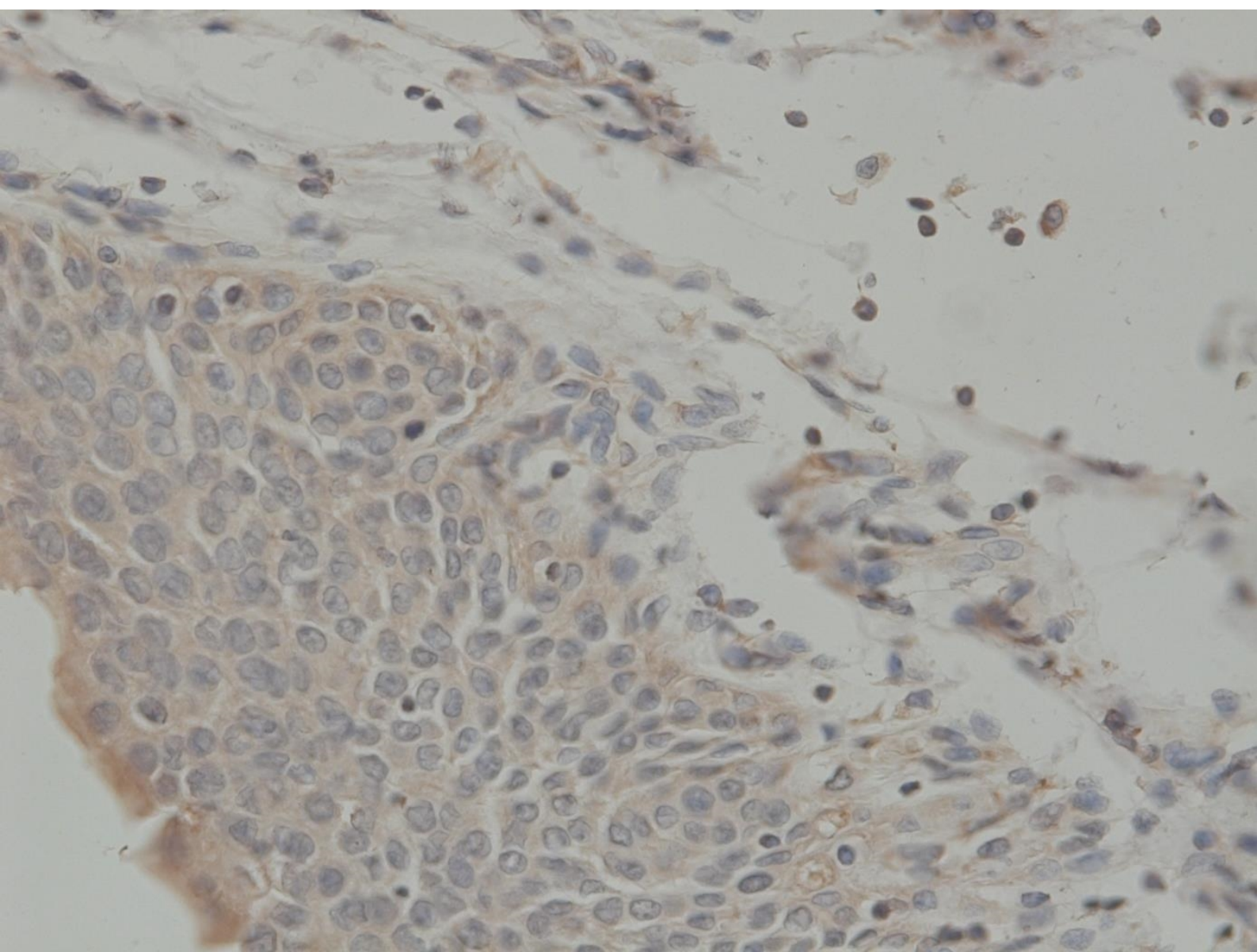

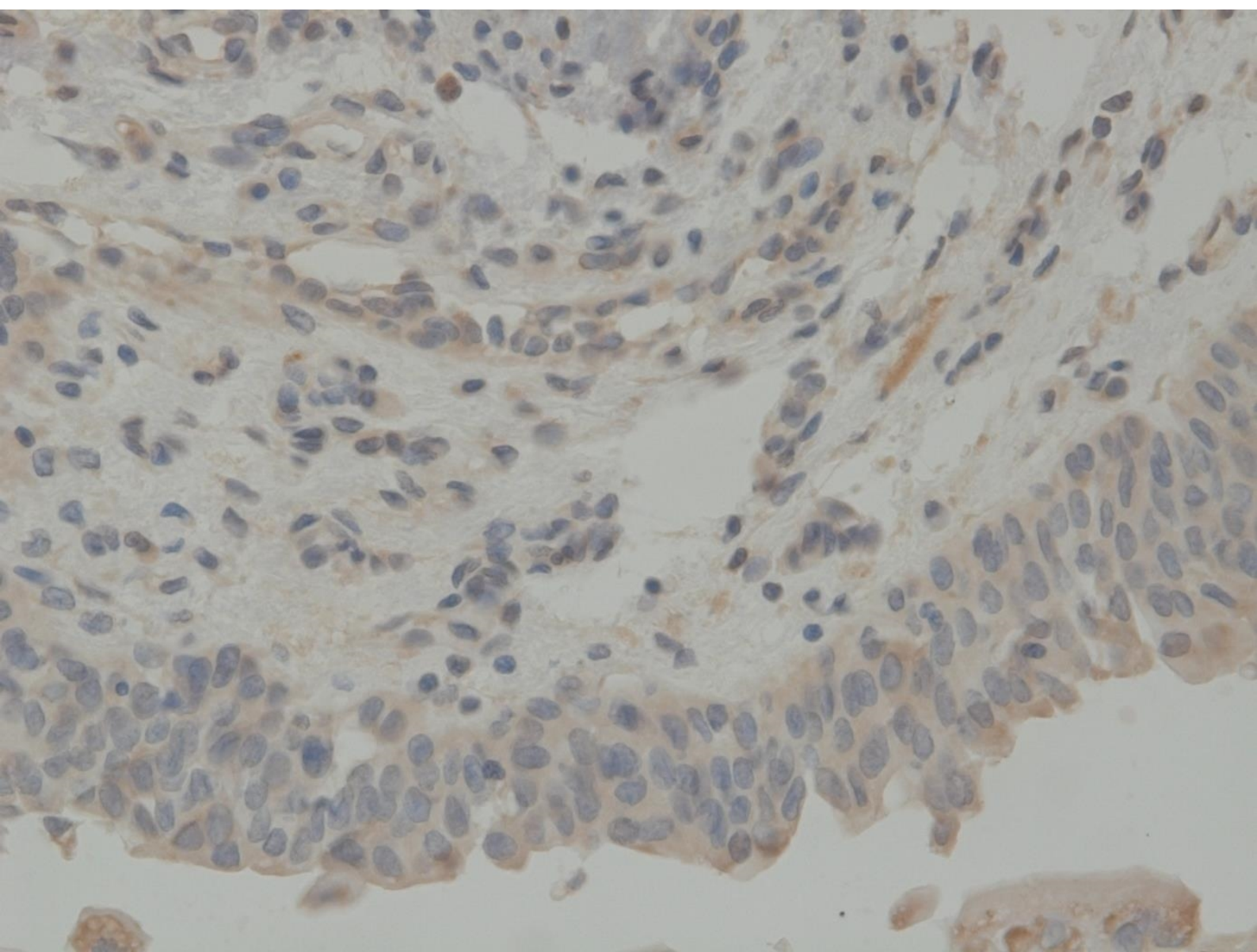

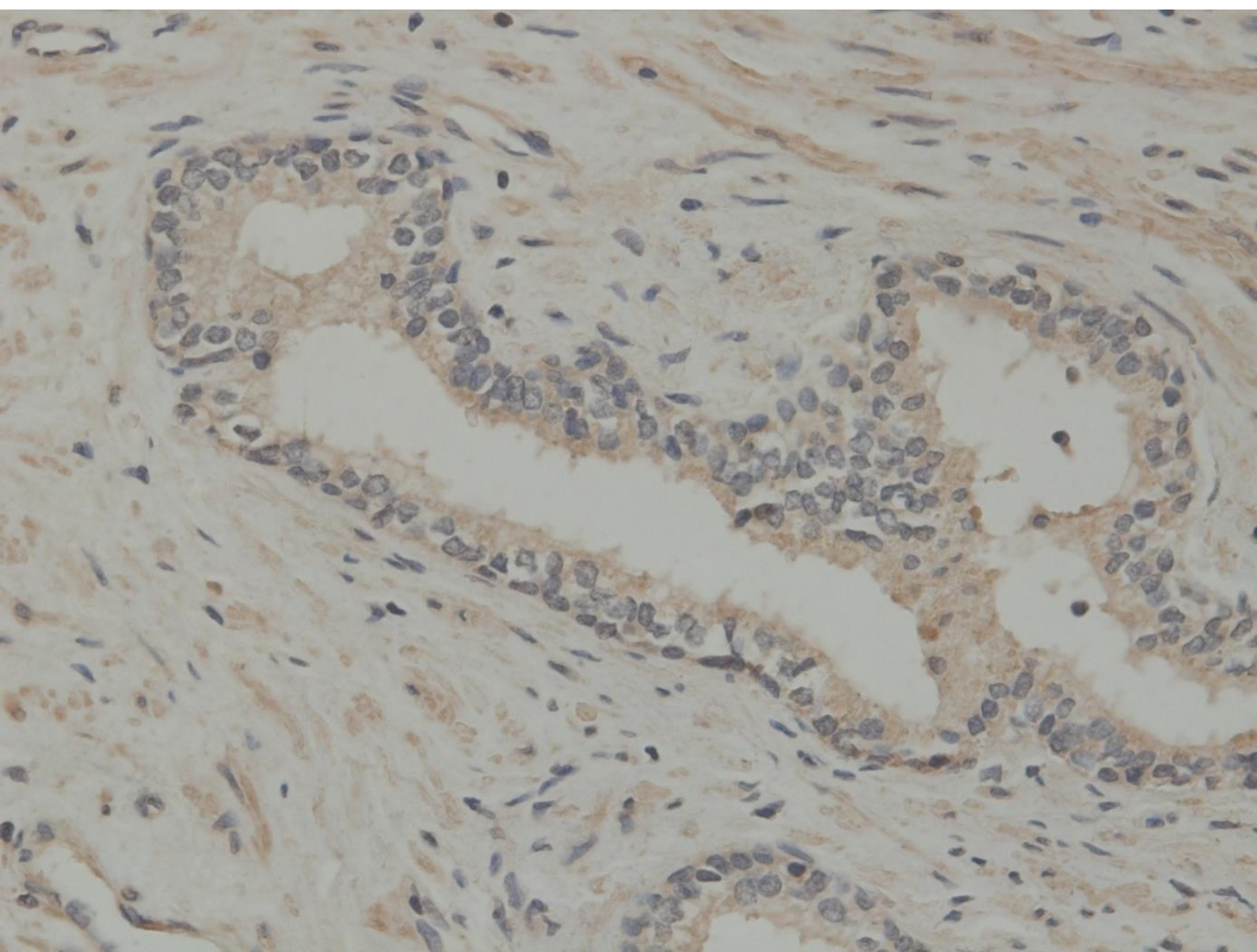

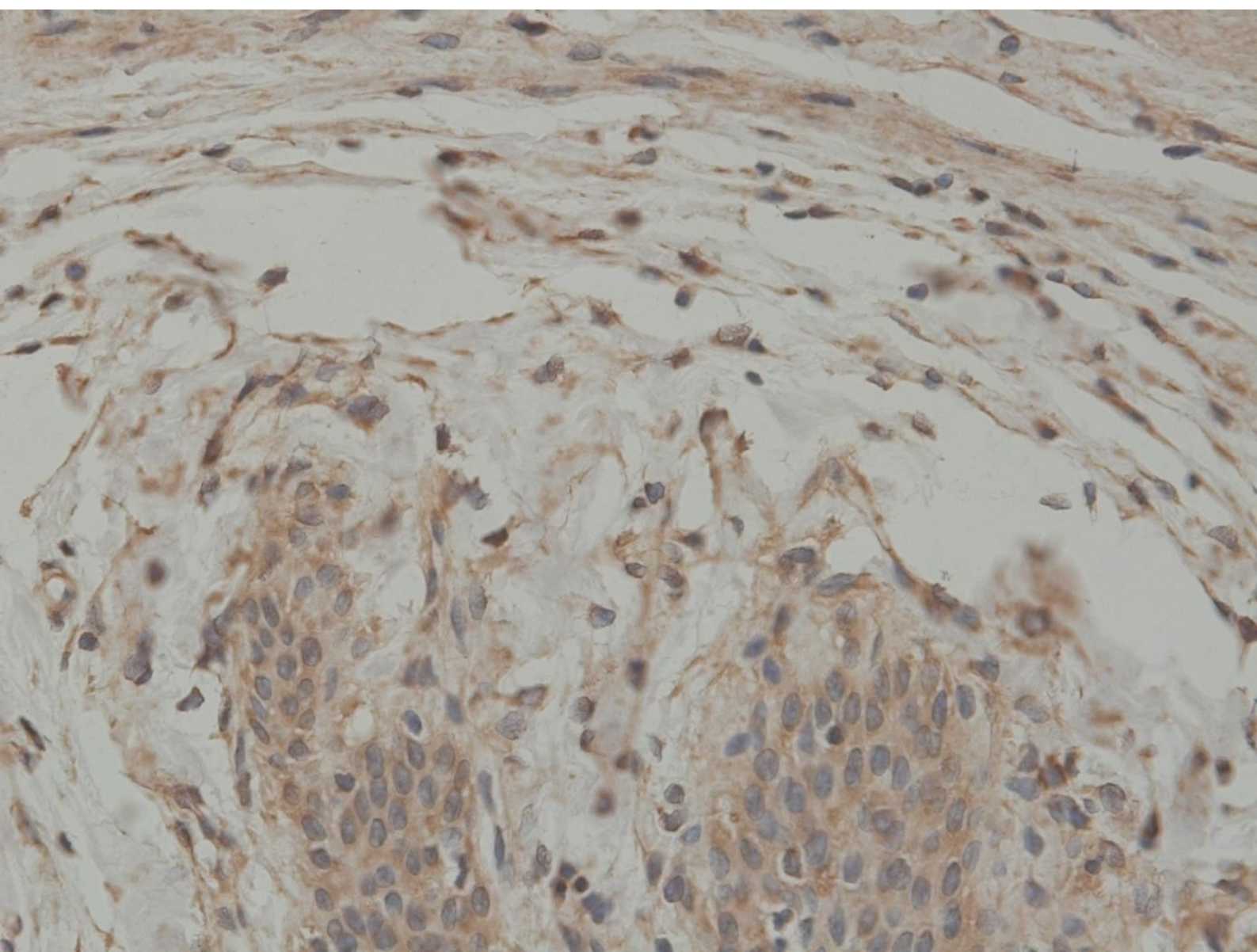

Supplement: Supplementary file 6 — Additional file 6. IHC 2: normal tissues Immunohistochemical result of patients [file 12894_2023_1211_MOESM6_ESM.pdf]
