# Supplementary material for: High expression of ABCF1 is an independent predictor of poor prognosis in bladder cancer
Source: BMC Urol. 2023 Mar 17;23:37. doi: 10.1186/s12894-023-01211-y (PMC10022215; doi:10.1186/s12894-023-01211-y)
Supplement: Supplementary file 4 — Additional file 4. PI: prognostic information of patients [file 12894_2023_1211_MOESM4_ESM.docx]

**Prognostic Information of Patients**

| Months after surgery | ABCF1-low | ABCF1-high |
| --- | --- | --- |
| 60 | 0 |  |
| 60 | 0 |  |
| 60 | 0 |  |
| 60 | 0 |  |
| 60 | 0 |  |
| 60 | 0 |  |
| 60 | 0 |  |
| 60 | 0 |  |
| 60 | 0 |  |
| 60 | 0 |  |
| 60 | 0 |  |
| 60 | 0 |  |
| 60 | 0 |  |
| 60 | 0 |  |
| 58 | 1 |  |
| 50 | 1 |  |
| 46 | 1 |  |
| 45 | 1 |  |
| 42 | 1 |  |
| 41 | 1 |  |
| 36 | 1 |  |
| 35 | 1 |  |
| 32 | 1 |  |
| 28 | 1 |  |
| 28 | 1 |  |
| 25 | 1 |  |
| 24 | 1 |  |
| 23 | 1 |  |
| 22 | 1 |  |
| 18 | 1 |  |
| 60 |  | 0 |
| 60 |  | 0 |
| 60 |  | 0 |
| 60 |  | 0 |
| 60 |  | 0 |
| 60 |  | 0 |
| 60 |  | 0 |
| 60 |  | 0 |
| 60 |  | 0 |
| 38 |  | 1 |
| 33 |  | 1 |
| 33 |  | 1 |
| 33 |  | 1 |
| 30 |  | 1 |
| 28 |  | 1 |
| 25 |  | 1 |
| 24 |  | 1 |
| 24 |  | 1 |
| 23 |  | 1 |
| 23 |  | 1 |
| 22 |  | 1 |
| 20 |  | 1 |
| 19 |  | 1 |
| 12 |  | 1 |
| 12 |  | 1 |
| 11 |  | 1 |
| 10 |  | 1 |
| 4 |  | 1 |
| 4 |  | 1 |
| 1 |  | 1 |

*ALIVE:0

*DEATH:1
